# Inductive Diastereoselective Rationale for a Catalytic Strategy to Build Densely Chiral Five-Membered Carbocycles

**DOI:** 10.1021/jacsau.6c00311

**Published:** 2026-05-21

**Authors:** Carmen M. Arenas-Baeza, Eva Rivera-Chao, Paula Dominguez-Molano, Andrea Chaves-Pouso, Jorge J. Carbó, Martín Fañanás-Mastral, Elena Fernández

**Affiliations:** † 16777 Universitat Rovira i Virgili, Departament de Química Física i Inorgànica, Tarragona 43007, Spain; ‡ Centro Singular de Investigación en Química Biolóxica e Materiais Moleculares(CiQUS). Departamento de Química Orgánica, 16780 Universidade de Santiago de Compostela, Santiago de Compostela 15782, Spain; § Oportunius, Galician Innovation Agency (GAIN), Santiago de Compostela 15702, Spain

**Keywords:** Cu-catalysis, cyclopentanes, multistereogenic centers, diastereoselectivity, DFT calculations

## Abstract

We report a streamlined synthetic strategy for accessing stereocongested five-membered carbocycles from enantioenriched branched borylated 1,4-dienes. The method enables precise control over diastereoselectivity and enantiospecificity in the construction of adjacent stereocenters within the newly formed cyclic frameworks. DFT calculations provide an understanding of the origin of diastereoselection along the Cu-catalyzed borylcupration step, allowing one to establish a stereochemical model that rationalizes the key ligand-substrate and intrasubstrate interactions governing selectivity. Moreover, the resulting stereocongested cyclopentenes can be further elaborated through stereoselective alkene activation, enabling the installation of up to four contiguous stereocenters and granting access to architecturally complex, multistereogenic cyclopentane frameworks.

## Graphical abstract

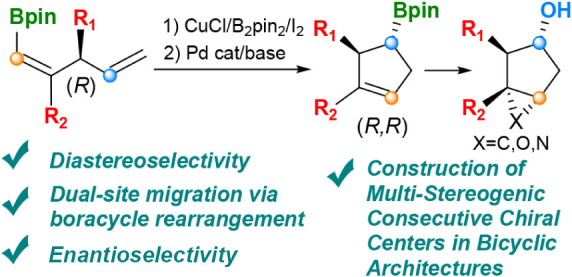

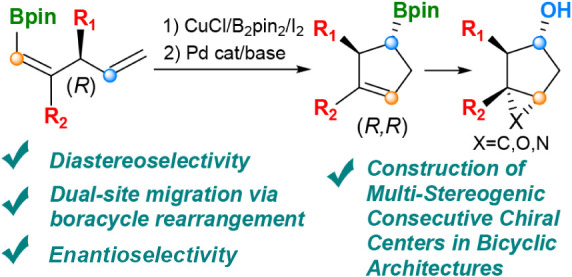

## Introduction

The access to highly substituted cyclopentanes and cyclopentenes, bearing congested arrays of stereocenters within the cyclic frameworks, is an incentive in modern synthetic chemistry. This is justified because that kind of stereocongested five-membered carbocycle is prevalently embedded in the structure of a wide range of bioactive molecules.^1–7^ Engineered cyclization methods that construct multistereogenic cyclopentene and cyclopentane frameworks, bearing a versatile C–B bond for downstream functionalization, are highly sought.^8^ Catalytic stereoselective borylative cyclization of unsaturated substrates allows efficient access to enantioenriched mixtures of five-membered carbocycles with contiguous stereocenters (strategy A, Scheme 1a).^9–18^ Alternatively, the stereoselective cyclization through boron electrophile-induced cyclic boron-ate formation, followed by ring contraction, also contributes to enriching the synthetic portfolio for stereocongested five-membered carbocycles (strategy B, Scheme 1b).^19,20^ Whereas in strategy A the pinacolboryl moiety is introduced regioselectivity through an asymmetric catalytic borylcupration, strategy B relies on a noncatalyzed carboboration in which the pinacolboryl fragment is already embedded in the chiral substrate, rendering the enantiospecific cyclization via a boron-ate intermediate.

**Scheme 1:**
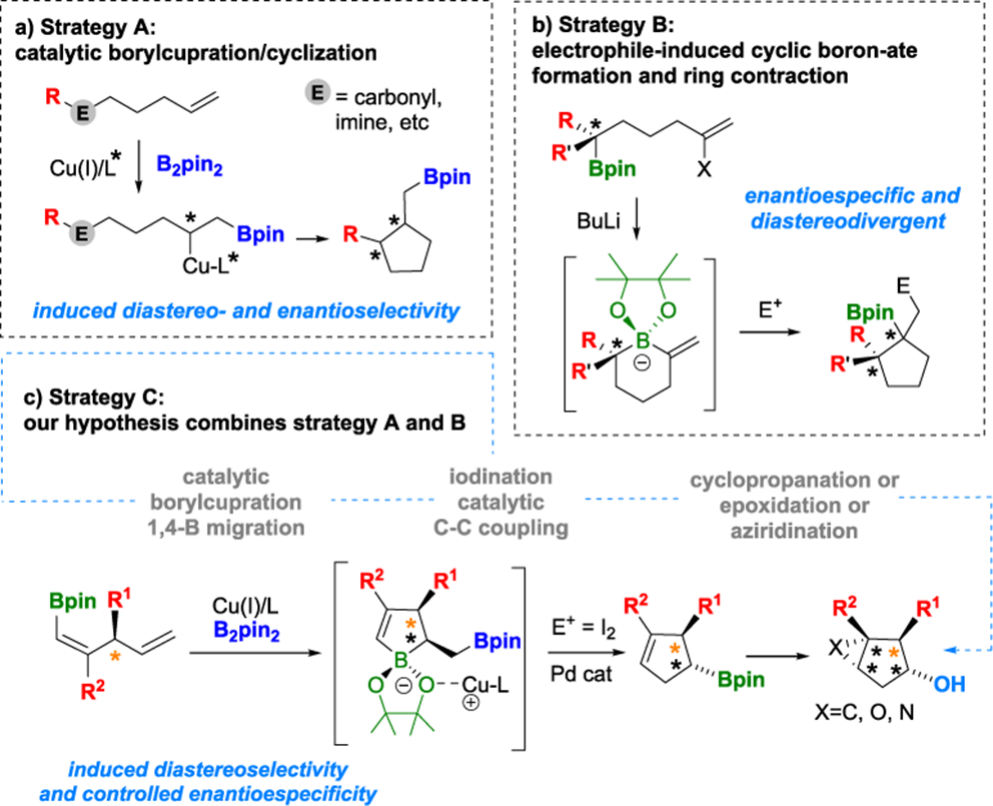
Cyclization Methods to Construct Multi-Stereogenic Cyclopentane Frameworks Bearing C–B Bonds

In both strategies A and B, however, diastereoselectivity is strongly dependent on the nature of the substrate structure, substitution pattern, solvent, and reaction conditions in general. We envisioned merging the key elements of both approaches into a new unified and operationally convenient strategy (strategy C) that enables precise diastereocontrol in the formation of contiguous stereocenters and, consequently, enantiospecific access to chiral cyclopentene scaffolds (Scheme 1c). These intriguing compounds might serve as a versatile platform to amplify the number of substituted stereocenters, up to four contiguous chiral centers, through assisted activation of the alkene motif, contributing to multistereogenic bicyclic molecular architectures.

To launch this study, we identified as suitable substrates, the enantioenriched borylated 1,4-dienes that can be efficiently prepared by conducting a straightforward copper-catalyzed asymmetric allylboration of alkynes.^21^ As reported by Fañanás–Mastral, this methodology employs a chiral sulfonate-bearing NHC/Cu catalyst to achieve the stereoselective construction of both the alkenyl boronate moiety and the bisallylic stereogenic center in a single step. The process proceeds from simple precursors such as an alkyne, B_2_pin_2_, and an allylic electrophile, and accommodates a broad range of electrophilic partners, including allylic bromides,^21a^ *gem*-dichlorides,^21b,c^ and phosphates.^21c^ Central to the present strategy is harnessing the ability of a single chiral center to direct the formation of three additional vicinal stereocenters within five-membered carbocycles, achieving complete diastereoselectivity and enantiospecificity.

## Results and Discussion

### Reaction Development

We explored the Cu-catalyzed borylcupration of the model skipped diene (±)-**1a** under the catalytic system comprising CuCl (10 mol %)/Xantphos (10 mol %), in the presence of B_2_pin_2_ (1.2 equiv) and KO^*t*^Bu as the base (Scheme 2). The reaction proceeded toward the chemo- and regioselective Cu-catalyzed borylcupration of the terminal alkene. The resulting pentenyl Cu­(I) intermediate undergoes a 1,4-B migration from a remote position along the alkyl chain, involving the migration of the C­(sp^2^)–Bpin unit and the formation of a C­(sp^3^)–Cu species through a transient boracycle intermediate. This remote Cu/Bpin migration has recently been observed in related achiral systems.^22^ The *in situ* sequence enables the concomitant migration of two distant sites across four carbon atoms, and subsequent reaction with I_2_ promotes boracycle rearrangement to furnish the electrophilic trapping product (±)-**2** in high yield and with remarkable diastereoselectivity (dr = 14:1) (Scheme 2). Overall, this process represents a stereoselective reaction cascade accomplished in a single operational step.

**Scheme 2:**
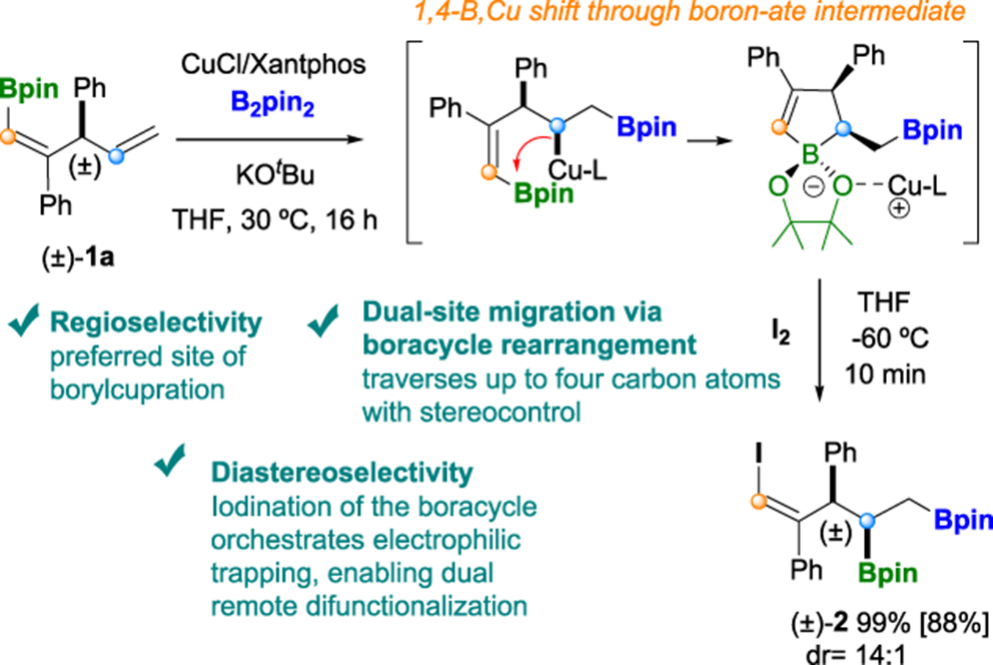
Diastereoselective Cu-Catalyzed Borylcupration/1,4-B Migrtion/Iodination of (*E*)-Skipped Diene 1a^a^

a Reaction conditions: borylcupration: (±)-**1a** (0.2 mmol), B_2_pin_2_ (1.2 equiv), CuCl (10 mol %), Xantphos (10 mol %), KO^*t*^Bu (1.2 equiv), THF (2 mL), 30 °C, 16 h; iodination: I_2_ (2 equiv), THF (2 mL), −60 °C, 10 min. NMR yields determined with naphthalene as the internal standard. Isolated yields in brackets.

Next, we investigated how the substituents R^1^ and R^2^ in borylated 1,4-dienes (±)-**1** influence diastereoselectivity during the Cu-catalyzed borylcupration (Scheme 3). When (*E*)-skipped diene (±)-**1b** (R^1^ = Ph, R^2^ = *o*-MeC_6_H_4_) was transformed into the desired alkenyl iodide product **3**, the diastereoselectivity slightly increased (dr = 16:1). Similarly, when R^2^ was *p*-BrC_6_H_4_ (substrate (±)-**1c**) or thiophenyl (substrate (±)-**1d**), the corresponding products (±)-**4** and (±)-**5** were obtained with lower diastereomeric ratios (dr = 8:1 and dr = 5:1, respectively). However, when the modification was introduced in R^1^ = *p*-CF_3_C_6_H_4_ (substrate (±)-**1e**), the alkenyl iodide product (±)-**6** could be isolated in 86% yield and with perfect diastereoselectivity (dr = 20:1).

**Scheme 3:**
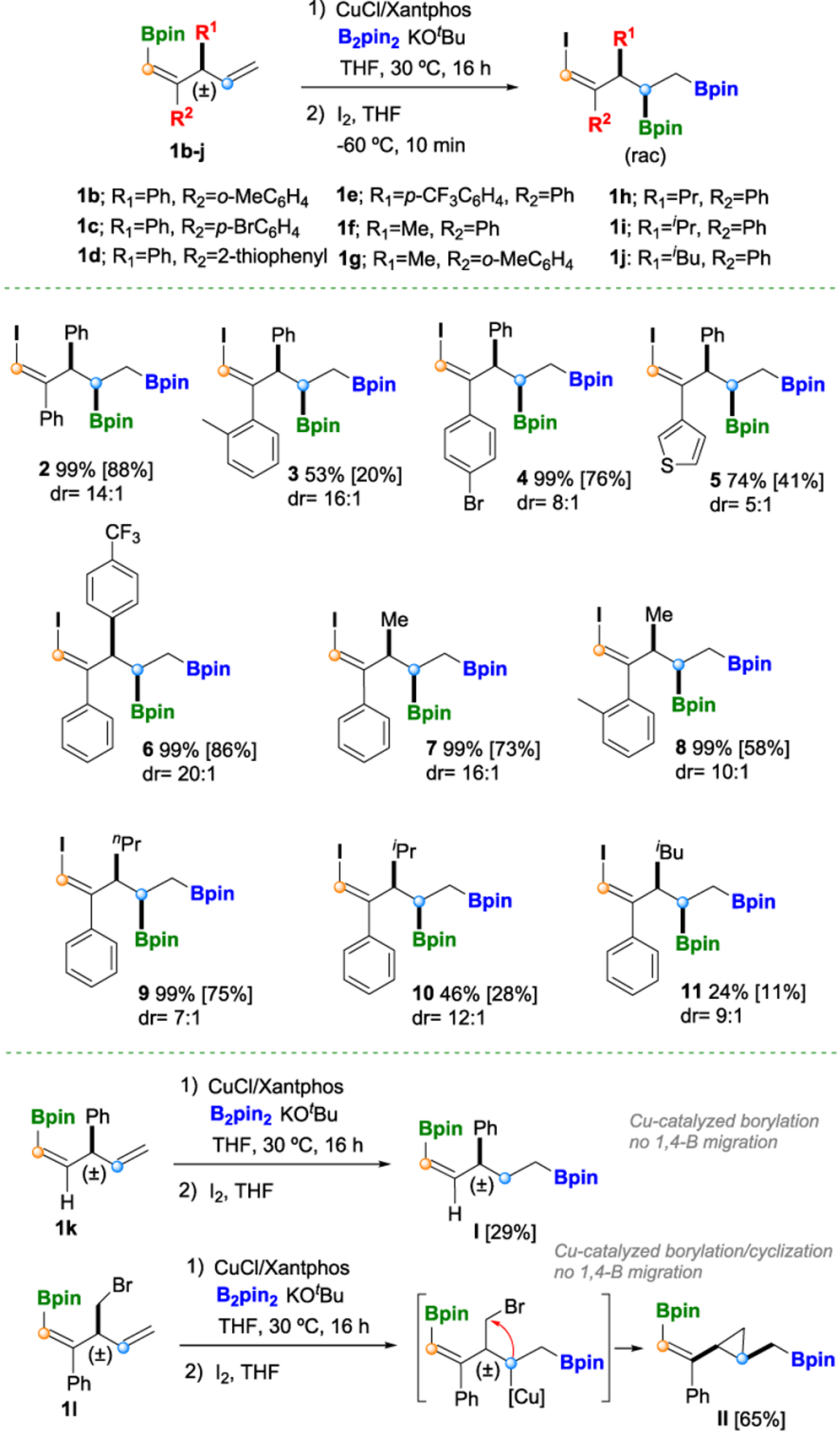
Cu-Catalyzed Borylcupration/1,4-B Migration/Iodination of (*E*)-Skipped Dienes (±)-1b-1j[Fn sch3-fn2]

a Reaction conditions: *borylcupration*: (±)-**1b**–**j** (0.2 mmol), B_2_pin_2_ (1.2 equiv), CuCl (10 mol %), Xantphos (10 mol %), KO^*t*^Bu (1.2 equiv), THF (2 mL), 30 °C, 16 h; *iodination*: I_2_ (2 equiv), THF (2 mL), −60 °C, 10 min. NMR yields determined with naphthalene as the internal standard. Isolated yields in brackets.

Much of the same behavior was observed when we used the methyl-substituted substrate (±)-**1f** (R^1^ = CH_3_), which led to product (±)-**7** in 73% yield and dr = 16:1. These results suggest that the modification on R^1^ (aryl or alkyl groups) has less influence on the reaction outcome and diastereoselectivity, whereas a subtle variation in R^2^ has a significant influence on the diastereoselectivity. To demonstrate this observation, we studied the Cu-catalyzed borylcupration/1,4-migration/iodination of (*E*)-skipped diene (±)-**1g** (R^1^ = Me, R^2^ = *o*-MeC_6_H_4_), and product (±)-**8** could be isolated in comparable yield but with lower diastereoselectivity (dr = 10:1) than product (±)-**7** (dr = 16:1). Evaluation of other alkyl R^1^ substituents ((±)-**1h**: R^1^ = ^*n*^Pr, (±)-**1i**: R^1^ = ^*i*^Pr and (±)-**1j**: R^1^ = ^*i*^Bu) denoted that the bulkier the alkyl group, the lower the yield, likely as a consequence of a more sterically hindered borylcupration sequence. By applying the Cu-catalyzed borylation of substrate (±)-**1k** (R^1^ = Ph, R^2^ = H), we observed exclusively the formation of the 1,5-diborylated 3-phenylpentene product **I**, likely generated via Cu-borylation without a 1,4-B migration sequence (Scheme 3). A distinct reactivity pattern was also observed in the Cu-catalyzed borylation of substrate (±)-**1l** (R^1^ = CH_2_Br, R^2^ = Ph), which afforded cyclopropyl bisboronate **II**, with exclusive *cis* stereoselectivity between the substituents (Scheme 3). This type of highly functionalized cyclopropane scaffold has only been accessed through closely related methodologies, in which cyclization prevails over any competing 1,4-B migration process.^22c^

To further validate our stereoselective strategy, palladium-catalyzed intramolecular cross-coupling was explored for products **2**–**10** (Scheme 4). When compound (±)-**2** reacted with a Pd­(OAc)_2_/Ruphos catalyst^23^ (Ruphos = dicyclohexyl­(2’,6’-diisopropoxy-[1,1’-biphenyl]-2-yl)­phosphine), cyclization proceeded with complete regioselectivity, engaging exclusively the terminal Bpin moiety. The reaction provided cyclopentene product (±)-**12**, which retains the original Bpin motif from substrate (±)-**1a**. The *trans* relative configuration observed in product (±)-**12** confirms the exclusive cyclization of the major diastereomer of (±)-**2**, thus amplifying the overall stereoselectivity and overcoming the notorious challenge associated with the catalytic stereoselective cyclization of pentylboranes.^9–18^ Encouraged by this result, we extended the synthetic protocol by varying the R^1^ and R^2^ substituents of the diborylated iodoalkenyl precursors. In all cases, cyclopentenes (±)-**13** to (±)-**20** were obtained exclusively as the *trans* diastereomers, demonstrating compatibility with a broad range of steric and electronic properties at R^1^ and R^2^. Oxidation of the C–Bpin bond furnished products (±)-**21** to (±)-**29** in quantitative yield (Scheme 4).

**Scheme 4:**
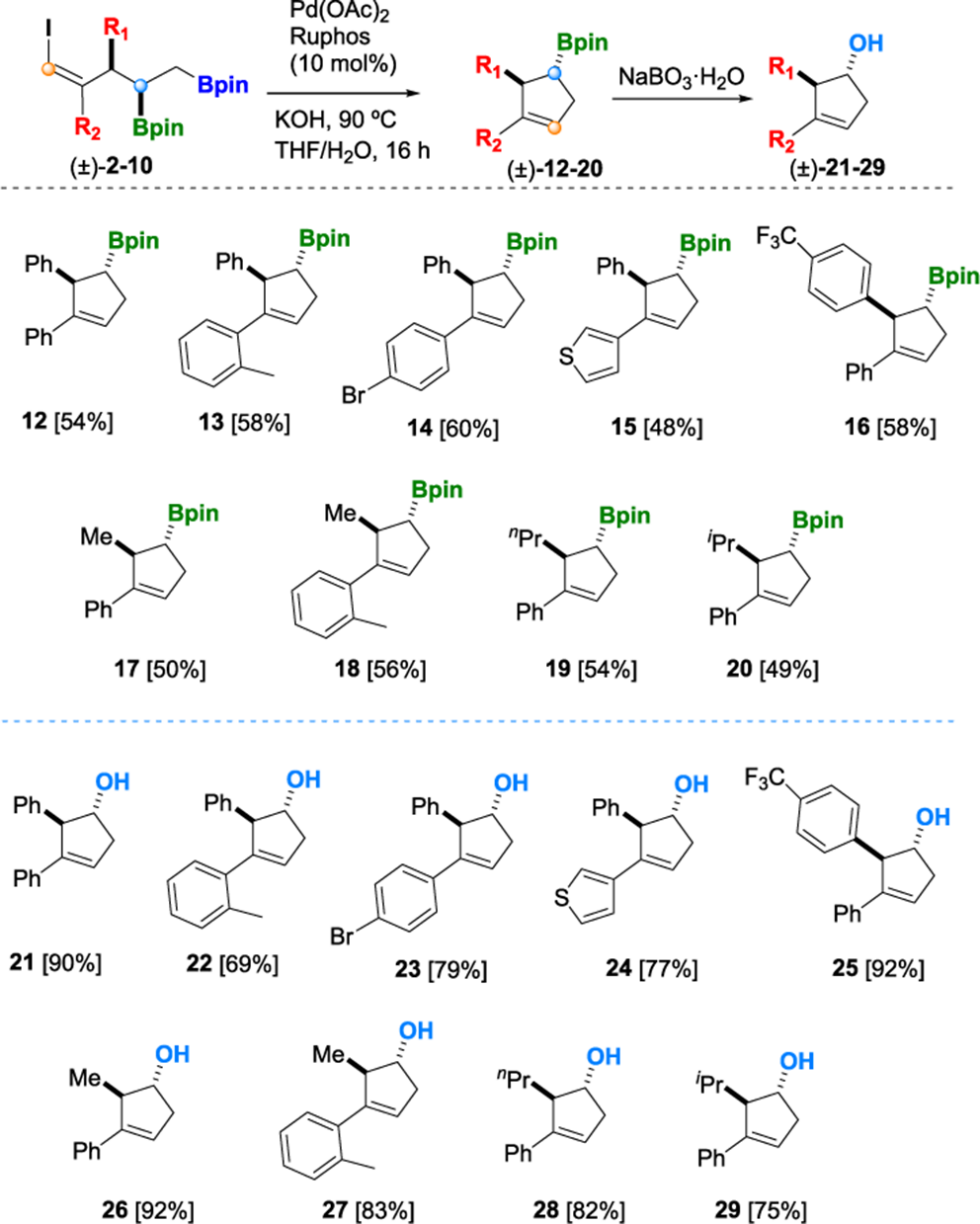
Pd-Catalyzed Intramolecular Regioselective Cross-Coupling Sequence[Fn sch4-fn3]

a Reaction conditions: *cross-coupling:* Pd­(OAc)_2_/Ruphos (Ruphos = dicyclohexyl­(2’,6’-diisopropoxy-[1,1’-biphenyl]-2-yl)­phosphine) (10 mol %), KOH (3 equiv), THF:H_2_O (2/0.2 mL), 90 °C, 16 h; *oxidation*: NaBO_3_·H_2_O (0.3 mmol), THF (2 mL). Isolated yields in brackets.

We next explored the asymmetric version of this synthetic route to assess whether the whole process could be enantiospecific. Toward this end, we proceeded to conduct the Cu-catalyzed borylcupration of (*R*)-**1a**, with concomitant 1,4-B migration/iodination and eventual Pd-catalyzed intramolecular coupling. As a result, we were able to isolate product (*R,R*)-**12** in 59% yield. Subsequent oxidation resulted in (1*R*,2*R*)-2,3-diphenylcyclopent-3-en-1-ol ((*R,R*)-**21**) which was obtained with 95:5 er, demonstrating that no erosion of chiral information occurred from the skipped diene (*R*)-**1a** (Scheme 5). The absolute configuration of the resulting cyclopentenol was determined by single-crystal X-ray diffraction analysis, which confirmed the *trans* distribution of the Ph and OH groups (Scheme 5).^24^

**Scheme 5:**
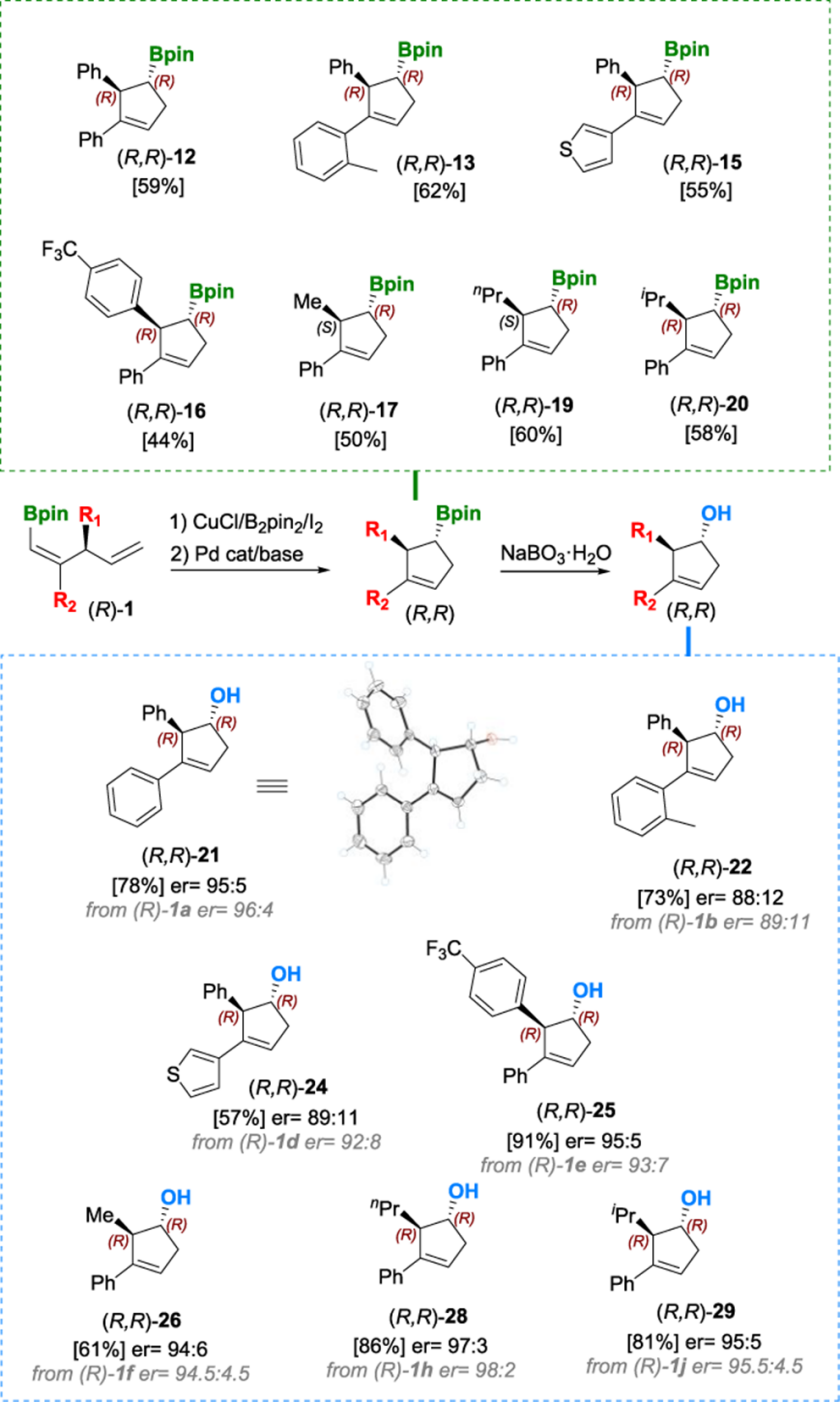
Diastereoselective and Enantiospecific Creation of Cyclopentenes Bearing Contiguous Stereocenters[Fn sch5-fn4]

a Reaction conditions: *borylcupration*: (*R*)-**1** (0.2 mmol), B_2_pin_2_ (1.2 equiv), CuCl (10 mol %), Xantphos (10 mol %), KO^*t*^Bu (1.2 equiv), THF (2 mL), 30 °C, 16 h; *iodination*: I_2_ (2 equiv), THF (2 mL), −60 °C, 10 min; *cross-coupling*: Pd­(OAc)_2_/Ruphos (10 mol %), KOH (3 equiv), THF:H_2_O (2/0.2 mL), 90 °C, 16 h; *oxidation:* NaBO_3_·H_2_O (0.3 mmol), THF (2 mL). Isolated yields in brackets.

The diphenyl-substituted cyclopentene (*R,R*)-**12** had been synthesized by Johnson and coworkers^25^ as part of a selected secondary transformation of highly functionalized cyclopentanols, prepared by stereodivergent Cu-catalyzed borylative cyclization of racemic β-oxo acid derivatives. Interestingly, Cho and coworkers applied the copper-catalyzed asymmetric allylic alkylation of allyl bromides with 1,1-diborylalkanes, followed by a ring-closing metathesis/oxidation pathway, to synthesize the opposite enantiomer (*S,S*)-**21**.^26^ Our methodology complements those of Johnson and Cho, providing an enantiospecific protocol that proved compatible with aryl and alkyl substituents on the cyclopentene frameworks. With evidence of the enantiospecificity of this transformation, we probed the compatibility of diverse steric and electronic parameters by varying the skipped diene substituents R^1^ and R^2^ (Scheme 5). A selection of enantioenriched borylated skipped dienes was converted into the corresponding cyclopentenyl boronates by applying our methodology, observing in all cases complete control over the diastereoselectivity and enantiospecificity of the reaction. The enantiomeric ratio was determined in the oxidized cyclopentenols, demonstrating that the chiral information from the skipped diene was fully preserved (Scheme 5, bottom).

### Computational Analysis of the Origin of Diastereoselectivity

To understand the origin of the diastereoselectivity observed in Cu-catalyzed borylcupration/1,4-B migration of chiral (*E*)-skipped dienes, we conducted DFT calculations^27^ using (*R*)-**1a** as the substrate. Scheme 6 shows the proposed reaction mechanism yielding the major diastereomer (see Supporting Information for more details), which is analogous to that previously characterized for achiral (*E*)-skipped dienes.^22a^ Following the *in situ* generation of the active catalyst Xantphos-Cu­(O^*t*^Bu) **I1**,^28^ the reaction proceeds via a mechanism consisting of: (1) the σ-bond metathesis of the Cu-alkoxide catalyst with B_2_pin_2_ to yield the Cu-boryl intermediate **I2**; (2) the regioselective borylcupration of the terminal double bond to yield the alkyl-Cu­(I) complex **I4** with borylation at the terminal carbon; (3) the intramolecular nucleophilic attack of the alkyl-Cu intermediate on the C­(sp^2^)-Bpin moiety, leading to the 5-membered boracycle **I5**; and (4) the electrophilic trapping of the boracycle, resulting in an overall 1,4-B migration. To identify this boracycle intermediate, we carried out the Cu-catalyzed borylation of **1a** and monitored the reaction by ^11^B NMR. The original signal for substrate **1a** was assigned at 31.3 ppm (CD_3_Cl as solvent). After 16 h under Cu/Xantphos and B_2_pin_2_ at 30 °C, a new signal appeared at a higher field, 21.1 ppm, which is consistent with the proposed boracycle intermediate involved in the 1,4-B migration. The overall computed free-energy barrier ((*R*)-**I4 →** (*R*)-**TS2**: 24.2 kcal·mol^–1^) is consistent with a reaction occurring at 30 °C (Scheme 6). More importantly, the early borylcupration step, through transition state (*R*)-**TS1**, is irreversible with a large reverse free-energy barrier ((*R*)-**I4 →** (*R*)-**TS1-R**) of 37.6 kcal·mol^–1^, and therefore, it corresponds to the diastereo-determining step.

**Scheme 6:**
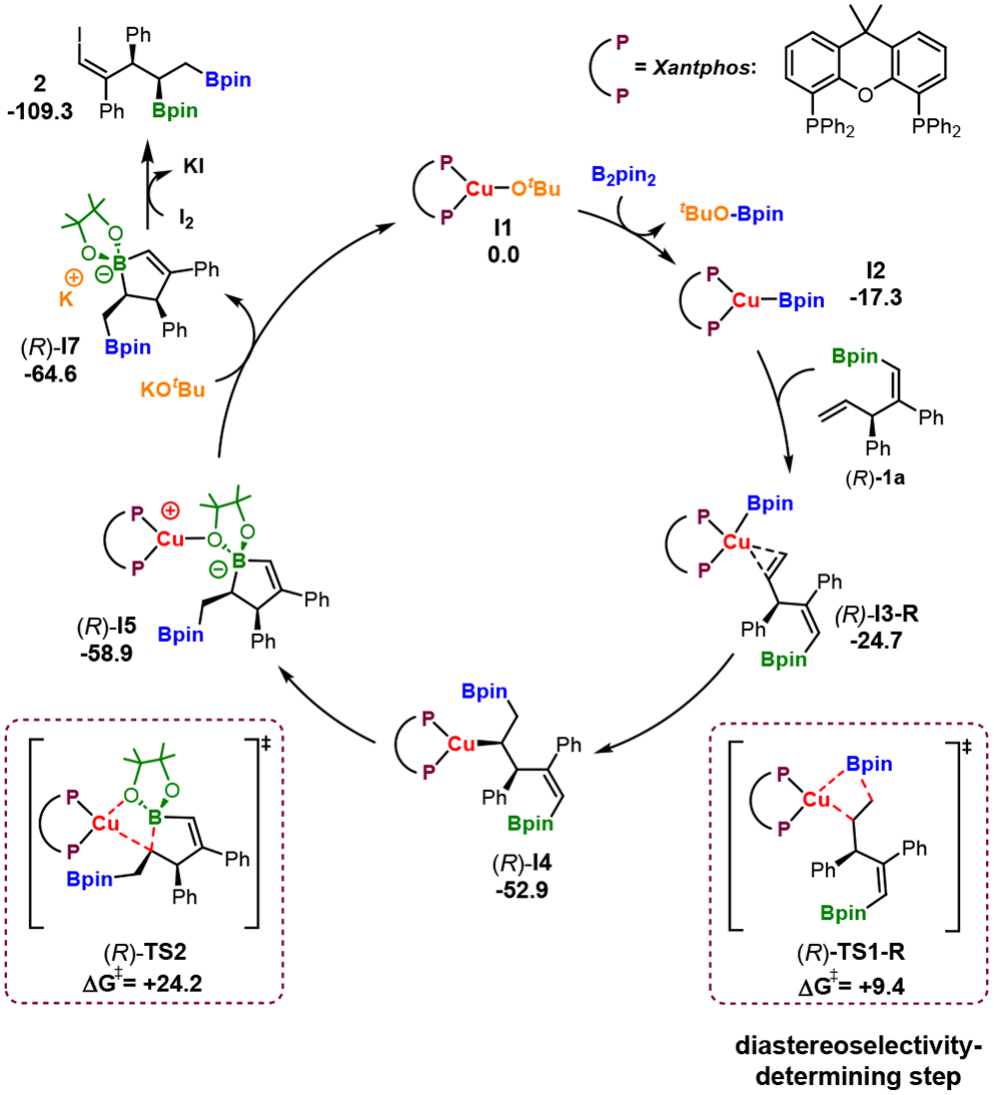
Proposed Reaction Mechanism For the Cu-Catalyzed Borylcuppration/1,4-B Migration/Iodination of (*R*)-1a[Fn sch6-fn5]

a Free energies in kcal·mol^–1^.

Next, we analyzed the differences between the two possible diastereomeric pathways leading to the major product (*R*,*R*)-**21** and the minor isomer (*R*,*S*)-**21**. Figure 1 shows the structures of the diastereoselectivity-determining transition states, their noncovalent interaction analysis (see Computational Details in Supporting Information), and the proposed stereochemical model. The free-energy difference between the corresponding diastereomeric transition states for borylcupration, (*R*)-**TS1**-**R** and (*R*)-**TS1**-**S**, is +1.2 kcal·mol^–1^ (see Figure 1) in agreement with the observations and very close to the value calculated from the experimental diastereomeric ratio (*dr* = 14:1 in Scheme 3) at 30 °C (+1.6 kcal·mol^–1^). Note that for each transition state, we performed conformational sampling, and the lowest-energy conformer was used for the analysis (see Supporting Information).

**Figure 1:**
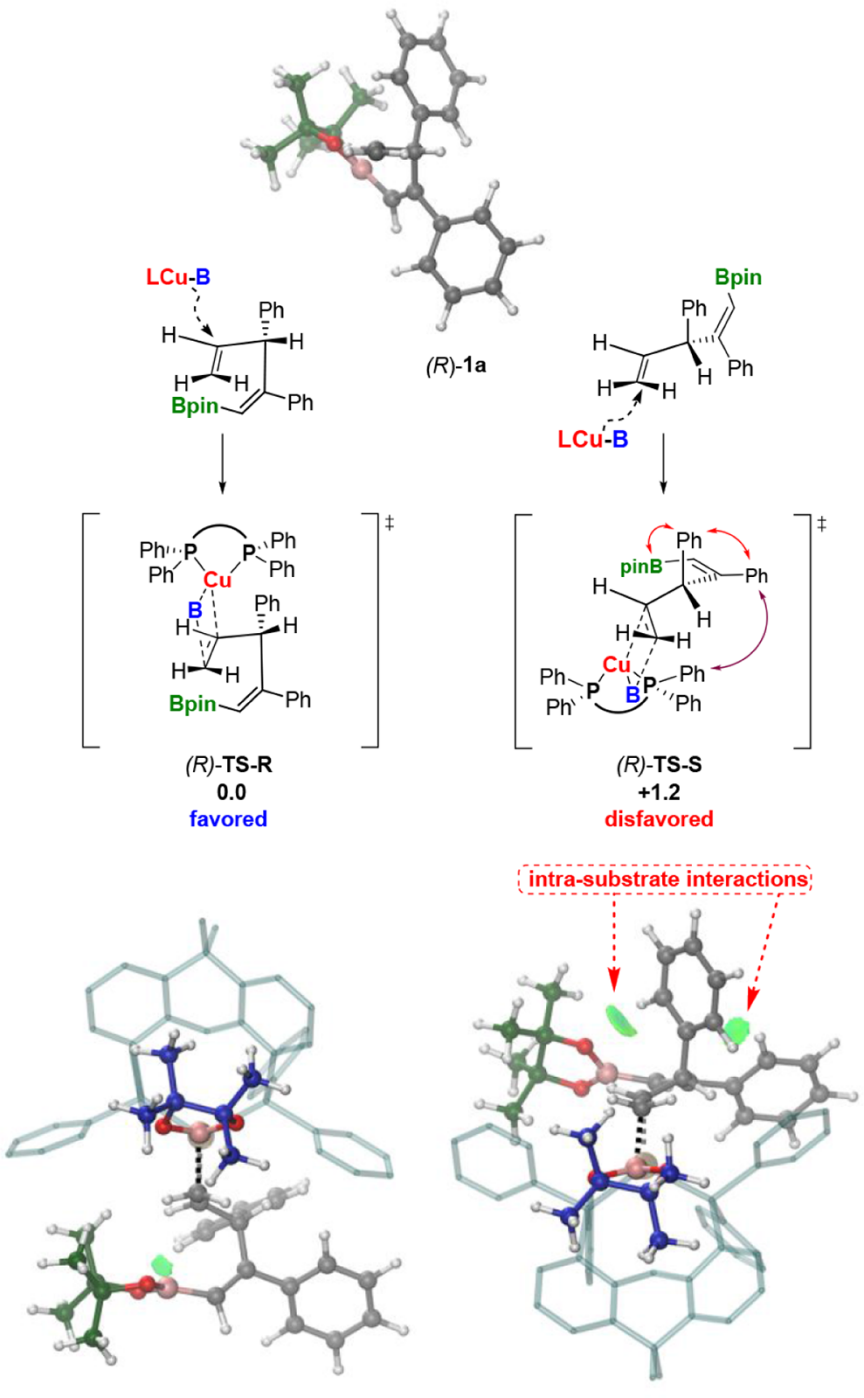
Proposed stereochemical model to explain diastereoselectivity based on transition states for the borylcupration step, and the molecular structures of transition states (*R*)-**TS1-R** and (*R*)-**TS1-S** along with noncovalent interaction analysis via the independent gradient model based on the Hirshfeld partition of molecular density IGMH. Relative free energies of transition states are in kcal·mol^–1^.

Figure 1 shows the initial conformation of the substrate (*R*)-**1a** and the possible approaches of the LCu-Bpin complex **I2** to the two enantiofaces of the carbon–carbon double bond, leading to transition states (*R*)-**TS1-R** and (*R*)-**TS1-S**. In each pathway, we observed a specific rotation of the allylic stereocenter bound to the terminal alkene moiety in order to minimize ligand-substrate steric interactions. To assess the effect of these rearrangements on both transition states, we evaluated the steric impact of substrate substituents on the metal center using the distance-weighted volume (*V*_W_) parameter,^29^ and no differences were appreciated (see Supporting Information). To further confirm these findings, we determined the diastereoselectivity induced by a model diphosphine, replacing each phenyl substituent with hydrogen while maintaining the backbone (XantPH_2_ model), which eliminated the nonbonding ligand-substrate interactions. Transitioning from the real-world Xantphos to the model XantPH_2_ ligand, the free-energy difference between the two diastereomeric transition states (*R*)-**TS1** remains almost identical, +1.2 and +1.3 kcal·mol^–1^, respectively. This indicates that ligand-substrate interactions do not directly determine the selectivity, but rather intrasubstrate interactions between the different substituents.

To minimize ligand-substrate interactions in the disfavored (*R*)-**TS1-S** structure, the system induces a conformational reorganization of the substrate that enhances the steric intrasubstrate interactions. As illustrated in Figure 1, the isodensity map derived from IGMH analysis identifies two main repulsive interactions (green-colored). These interactions correspond to an allylic 1,3-strain between the Bpin substituent on the double bond and the Ph-substituted allylic stereocenter, as well as a steric clash between the two Ph groups.^30^ Conversely, for the favored (*R*)-**TS1-R** structure, these interactions are only observed between the Ph group of the chiral center and the Bpin moiety to a smaller extent. In addition, geometry optimization of the substrate starting at the conformations in (*R*)-**TS1** structures led to two different isomers, the one derived from (*R*)-**TS1-S** being 0.8 kcal·mol^–1^ higher in energy, further proving that intrasubstrate interactions govern the diastereoselectivity. According to the stereochemical model, the substituent R^1^ (or R^2^) participates in repulsive interactions of both diastereoisomeric transitions states, disrupting the eventual linear structure-selectivity relationships. This, for example, explains why increasing the size of the substituent at R^1^ (Me (**1f**) < ^*n*^Pr (**1h**) < ^*i*^Pr (**1i**) < ^*t*^Bu (**1j**)) is not correlated to the observed diastereoselectivity (16:1, 7:1, 12:1, and 9:1, respectively, see Scheme 3). To sum up, the chiral center in the substrate governs its optimal and suboptimal conformation upon interaction with the Xantphos ligand at the borylcupration transition states (*R*)-**TS1-R** and (*R*)-**TS1-S**, respectively, ultimately governing the observed diastereoselectivity.

### Building Multistereogenic Chiral Bicyclic Architectures

The new enantioenriched cyclopentenols served as versatile structures to induce the stereoselective formation of new chiral sites. We first evaluated the formation of additional contiguous stereocenters under diastereofacial discrimination of alkene addition protocols. When (1*R*,2*R*)-2,3-diphenylcyclopent-3-en-1-ol ((*R,R*)-**21**) (er = 95:5) reacted with diiodomethane and a zinc complex, the Simmons–Smith cyclopropanation reaction was efficiently conducted. Under the optimized reaction conditions,^31^ the cyclopropanation proceeded in a diastereomeric manner, as zinc coordinates with the alcohol substituent and directs the cyclopropanation *cis* to the hydroxyl group (Scheme 7). Following this OH-directed synthetic strategy, bicyclo­(1*R*,2*R*,3*R*,5*S*)-1,2-diphenylbicyclo­[3.1.0]­hexan-3-ol (**30**), bearing four contiguous stereocenters, was isolated in 71% yield with 94:6 er (Scheme 7a). This method complements the current approaches for preparing analogues of Thujone, considered an inhibitor of the γ-aminobutyric acid A (GABAA) receptor.^32^

**Scheme 7:**
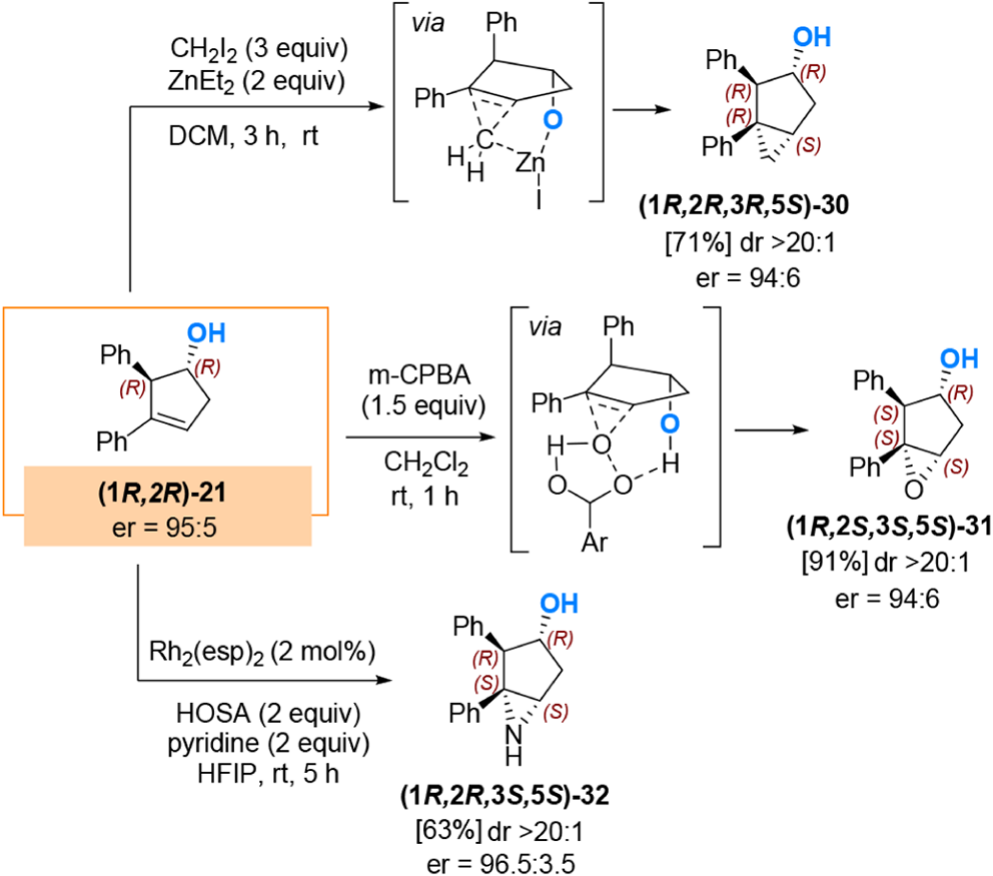
Synthesis of Bicyclic Molecular Architectures[Fn sch7-fn6]

a Reaction conditions: *cyclopropanation*: CH_2_I_2_ (3 equiv), ZnEt_2_ (2 equiv), CH_2_Cl_2_, rt, 3 h; *epoxidation*: *m*CPBA (1.5 equiv), CH_2_Cl_2_, rt, 1 h; *aziridination*: Rh_2_(esp)_2_ (2 mol%), HOSA (2 equiv), pyridine (2 equiv), HFIP, rt, 5 h. Yields of isolated products are shown in brackets.

Eventually, epoxidation of **21** employing *meta*-chloroperbenzoic acid (*m*CPBA) also occurred with complete diastereoselectivity, providing bicyclic compound **31** with excellent enantiospecificity (er = 94:6). The high diastereoselection observed likely arises from a strong hydrogen-bonding interaction between the alcohol moiety and the peroxyacid.^33^ Likewise, the rhodium-catalyzed N–H aziridination of product **21** employing hydroxylamine-*O*-sulfonic acid (HOSA) as the aminating reagent^34^ proceeded with total diastereoselectivity and enantiospecificity, rendering bicyclic product **32** in good yield as a single stereoisomer.

## Conclusions

In summary, we have developed a catalytic sequential protocol to prepare stereocongested five-membered carbocycles from enantioenriched branched borylated 1,4-dienes. Diastereoselectivity—and consequently enantiospecificity—in the formation of contiguous stereocenters within the new cyclic scaffolds is well controlled. DFT calculations provide insight into the origin of diastereoselection during the Cu-catalyzed borylcupration step, from which we derived a stereochemical model that rationalizes the ligand–substrate and intrasubstrate interactions governing selectivity. From stereocongested cyclopentenes, we further increased molecular complexity by stereoselective activation of the alkene motif through cyclopropanation, epoxidation, and aziridination, enabling the formation of up to four contiguous chiral centers and thus accessing multistereogenic bicyclic structures.

## Methods

### General Procedure for Cu-Catalyzed Borylcupration-1,4-B Migration/Iodination

The following reagents, CuCl (0.98 mg, 10 mol %, 0.01 mmol), bis­(pinacolato)­diboron (60.9 mg, 1.2 equiv, 0.24 mmol), and Xantphos (138.8 mg, 10 mol %, 0.01 mmol), were placed in a flamed Schlenk tube. The Schlenk tube was sealed with a screw cap containing a Teflon-coated rubber septum. The Schlenk tube was connected to a vacuum/nitrogen manifold through a needle, evacuated, and backfilled with nitrogen and THF (0.24 mL, 1 M). A solution of the base KO^*t*^Bu (26.9 mg, 1.2 equiv, 0.24 mmol) in THF (0.24 mL, 1 M) was added to the reaction medium through the rubber septum. Then, the borylated skipped diene (1 equiv, 0.2 mmol) in THF (0.2 mL, 1 M) was added dropwise at 30 °C and stirred for 16 h. Subsequently, iodine (101.5 mg, 2 equiv, 0.4 mmol) was dissolved in 2 mL of THF in a second oven-dried Schlenk tube. The reaction mixture was then kept at −60 °C while the iodine solution was passed through Teflon tubing. The cooling bath was removed, and the temperature was allowed to rise to room temperature. After 10 min at room temperature, a mixture of 10 mL of saturated sodium bisulfite was added with vigorous stirring and washed with Et_2_O (3 × 15 mL). The organic extracts were dried over anhydrous magnesium sulfate and then concentrated under vacuum. The NMR yield was calculated through comparison to an internal standard (naphthalene). The crude residue was purified by silica gel flash chromatography to obtain the desired product.

### Synthesis of Compound (*R,R*)-2

CuCl (0.98 mg, 10 mol %, 0.01 mmol), bis­(pinacolato)­diboron (60.9 mg, 1.2 equiv, 0.24 mmol), and Xantphos (138.8 mg, 10 mol %, 0.01 mmol) were placed in a flamed Schlenk tube. The Schlenk tube was sealed with a screw cap containing a Teflon-coated rubber septum. The Schlenk tube was connected to a vacuum/nitrogen manifold through a needle, evacuated, and backfilled with nitrogen and THF (0.24 mL, 1 M). A solution of KO^*t*^Bu (26.9 mg, 1.2 equiv, 0.24 mmol) in THF (0.24 mL, 1 M) was added to the reaction medium through the rubber septum. Then, a solution of (*R*)-(*E*)-2-(2,3-diphenylpenta-1,4-dien-1-yl)-4,4,5,5-tetramethyl-1,3,2-dioxaborolane (*R*)-**1a** (70 mg,1 equiv, 0.2 mmol) in THF (0.2 mL, 1 M) was added dropwise at 30 °C and stirred for 16 h. Subsequently, iodine (101.5 mg, 2 equiv, 0.4 mmol) was dissolved in 2 mL of THF in a second oven-dried Schlenk tube. The reaction mixture was then kept at −60 °C while the iodine solution was passed through Teflon tubing. The cooling bath was removed, and the temperature was allowed to rise to room temperature. After 10 min at room temperature, a mixture of 10 mL of saturated sodium bisulfite was added with vigorous stirring and washed with Et_2_O (3 × 15 mL). The organic extracts were dried over anhydrous magnesium sulfate and then concentrated under vacuum. The NMR yield was calculated through comparison to an internal standard (naphthalene). The crude residue was purified by silica gel flash chromatography to obtain the desired product (*R,R*)-**2** (108 mg, 90% yield).

### Synthesis of Cyclic Compound (*R,R*)-12

In a flamed Schlenk tube equipped with a magnetic stir bar, Pd­(OAc)_2_ (4.5 mg, 10 mol %), RuPhos (9.33 mg, 10 mol %), and the substrate (*R,R*)-**2** (120 mg, 0.2 mmol) were added in THF (2 mL). Under an argon atmosphere, KOH (33.6 mg, 0.6 mmol, 3 equiv) and deoxygenated water (0.2 mL) were added. The reaction mixture was stirred at 90 °C for 16 h. After that, the mixture was filtered, the organic extracts were concentrated under vacuum, and the NMR yield was calculated through comparison to an internal standard (naphthalene). The crude residue was purified by silica gel flash chromatography to obtain the desired product (*R,R*)-**12** (40 mg, 59% yield).

### Cyclopropanation to Synthesize (1*R*, *2R*, *3R*, *5S*)-30

To a solution of the oxidized cyclopentene product (*R*,*R*)-**12** (47 mg, 0.2 mmol, 1.0 equiv) in anhydrous DCM (2.0 mL, 0.1 M), ZnEt_2_ (1.0 M in hexane, 0.4 mL, 0.4 mmol, 2.0 equiv) was added at 0 °C. After stirring for 10 min, CH_2_I_2_ (161 mg, 0.6 mmol, 3.0 equiv) was added. The mixture was stirred at room temperature, and a white precipitate was gradually generated. After 3 h, the mixture was quenched with saturated aqueous NH_4_Cl (8 mL) and extracted with EtOAc (3 × 10 mL). The combined organic layers were dried with Na_2_SO_4_, filtered over Celite, and concentrated in vacuo. The residue was purified by flash column chromatography on silica gel (eluting with petroleum ether/ethyl acetate = 20/1) to give the corresponding product (1*R*, *2R*, *3R*, *5S*)-**30** (36 mg, 71% yield).

### Synthesis of (1*R*,2*S*,3*S*,5*S*)-31 by Epoxidation

(1*R*,2*R*)-**21** (0.55 mmol, 1.0 equiv) and *m*-CPBA (0.85 mmol, 1.5 equiv) were diluted in CH_2_Cl_2_ (5.5 mL, 0.1 M). The reaction mixture was stirred at room temperature for 1 h. After this time, the reaction was quenched with the addition of satd aq. NaHCO_3_ (2 mL). The reaction mixture was extracted with CH_2_Cl_2_ (2 × 2 mL). The combined organic layer was dried over Na_2_SO_4_, filtered, and concentrated under reduced pressure. The residue was purified by flash column chromatography on silica gel (eluting with *n*-hexane/ethyl acetate = 90/10) to give the product (1*R*,2*S*,3*S*,5*S*)-**31** (127 mg, 91% yield).

### Synthesis of (1*R*,2*R*,3*S*,5*S*)-32 by Rh-Catalyzed Aziridination

(1*R*,2*R*)**−21** (0.1 mmol, 1.0 equiv) was dissolved in HFIP (0.26 mL, 0.4 M) at room temperature. Pyridine (0.2 mmol, 2.0 equiv), HOSA (0.2 mmol, 2.0 equiv), and Rh_2_(esp)_2_ (Du Bois’ catalyst, 0.002 mmol, 2 mol %) were added sequentially, and the reaction was stirred for 6 h at room temperature. After this time, the reaction was quenched at rt with the addition of sat. aq. Na_2_CO_3_ (1 mL) and distilled water (2 mL). The reaction mixture was extracted with CH_2_Cl_2_ (2 × 4 mL). The combined organic layer was dried over Na_2_SO_4_, filtered, and concentrated under reduced pressure. The residue was purified by flash column chromatography (eluting with *n*-hexane/ethyl acetate = 60/40) to give the desired product (1*R*,2*R*,3*S*,5*S*)**−32** (16 mg, 63% yield).

## Supplementary Material



## Notes

Supporting Information contains general and specific procedures for the synthesis of substrates, reagents, and products, as well as full characterization through spectral data, HPLC analysis, and X-ray diffraction (PDF)
