# Supplementary material for: Inductive Diastereoselective Rationale for a Catalytic Strategy to Build Densely Chiral Five-Membered Carbocycles
Source: JACS Au. 2026 May 21;6(6):3274–81. doi: 10.1021/jacsau.6c00311 (PMC13291998; doi:10.1021/jacsau.6c00311)
Supplement: Supplementary file 1 [file au6c00311_si_001.pdf]

Supplementary Information

Inductive Diastereoselective Rationale for a Catalytic Strategy to Build Densely Chiral Five-Membered Carbocycles

Carmen M. Arenas-Baeza,[+a] Eva Rivera-Chao,[+b] Paula Dominguez-Molano,[a] Andrea Chaves-Pouso,[b] Jorge J. Carbó,[a]* Martín Fañanás-Mastral,[b,c]* Elena Fernández[a]*

[a]Universitat Rovira i Virgili, Departament de Química Física i Inorgànica, 43007 Tarragona, Spain. [b]Centro Singular de Investigación en Química Biolóxica e Materiais Moleculares (CiQUS). Departamento de Química Orgánica. Universidade de Santiago de Compostela, 15782 Santiago de Compostela, Spain. [c]Oportunius, Galician Innovation Agency (GAIN), 15702 Santiago de Compostela, Spain

E-mail: email:mariaelena.fernandez@urv.cat, martin.fananas@usc.es, j.carbo@urv.cat

[+] These authors contributed equally to this work

Contents

General information 2

General method for Cu-catalyzed allylboration of alkynes 3

General method for Cu-catalyzed borylcupration-1,4-B migration-iodination 4

Specific example Cu-catalyzed borylcupration-1,4-B migration-iodination of (*R*)-**1a** 4

General method for intramolecular Suzuki-Miyaura cross-coupling 5

Specific example for intramolecular Suzuki-Miyaura cross-coupling of (*R*,*R*)-**2** 5

General method for oxidation of C-B bond 5

General method for cyclopropanation reaction 6

General method for epoxidation reaction 6

General method for aziridination reaction 6

Characterization data for (*E*)-skipped dienes 7

Characterization data for products isolated from Cu-catalyzed borylcupration-1,4-B migration-iodination 13

Characterization data for cyclic products 19

^{1}H, ^{13}C, ^{11}B, ^{19}F spectra for products 28

^{11}B NMR experiment to demonstrate formation of boracycle intermediate 85

HPLC analysis 86

X-Ray Diffraction 100

Computational Details 115

References 145

General information

Solvents and reagents: Solvents and reagents were obtained from commercial suppliers and dried and/or purified (if needed) by standard procedures. Diboron reagents were purchased from Ally Chem and used without further purification. All reactions were conducted in an oven and flame-dried glassware under an inert atmosphere of argon, using Schlenk-type techniques. *Flash chromatography* was performed on standard silica gel (Merck Kieselgel 60 F254 400-630 mesh). *Thin-layer chromatography* was performed on Merck Kieselgel 60 F254, which was developed using standard visualizing agents: UV fluorescence (254 and 366 nm) or potassium permanganate/Δ. *NMR spectra* were recorded at a Varian Goku 400 or a Varian Mercury 400 spectrometer. ^{1}H NMR and $^{13}C\{^{1}H\}$ NMR chemical shifts (δ) are reported in ppm with the solvent resonance as the internal standard ($CHCl_3$: 7.26 ppm (^{1}H)) and ($CDCl_3$: 77.16 ppm (^{13}C). $^{11}B\{^{1}H\}$ NMR chemical shifts (δ) are reported in ppm relative to $(CH_3)_2O\cdots BF_3$. Data are reported as follows: chemical shift, multiplicity (s = singlet, d = doublet, t = triplet, q = quartet, hept = septuplet, br = broad, m = multiplet), coupling constants (Hz), and integration. *High-resolution mass spectra (HRMS)* were recorded using a 6210 Time of Flight (TOF) mass spectrometer from Agilent Technologies (Waldbronn, Germany) with an ESI interface and it was performed at the Unidade de Espectrometría de Masas e Proteómica (Universidade de Santiago de Compostela, Santiago de Compostela). GC-MS analyses were performed on an HP6890 gas chromatograph, and an Agilent Technologies 5973 Mass selective detector (Waldbronn, Germany) equipped with an achiral capillary column HP-5 (30m, 0.25mm i. d., 0.25μm thickness) using He as the carrier gas. Chiral HPLC was performed using Diacel or Chiralcel columns (4.6 x 250 mm) fitted with the respective guards (4 x 10 mm) and monitored by DAD (Diode Array Detector). $[\alpha]D_{20}$: Optical rotations were measured at 20 °C on a Jasco P-2000 polarimeter with a sodium lamp at 589 nm and a path of length of 1 dm. Solvent and concentration are specified in each case. Full sphere single crystal data collection were performed at 100 K on a Bruker Kappa Apex II DUO diffractometer equipped with a Cryostream 700 plus low temperature device.

General method for Cu-catalysed allylboration of alkynes[1]

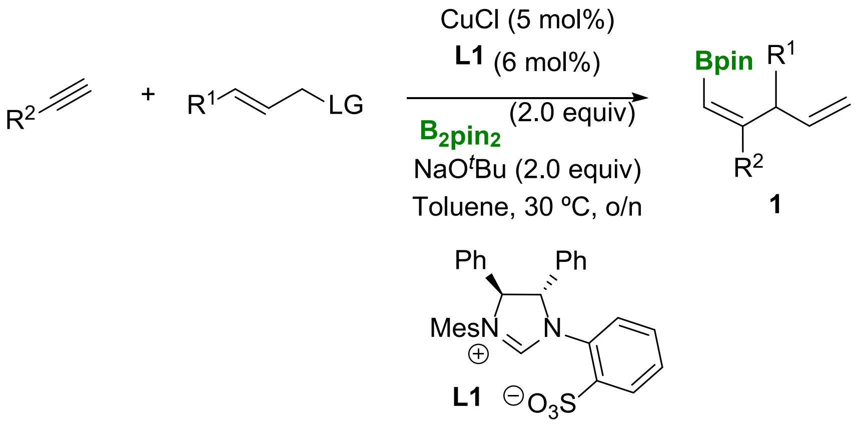

GP-1A. Cu-catalyzed allylboration of alkynes: CuCl (2.0 mg, 5 mol%, 0.02 mmol) and **L1** (enantioselctive version) or *rac*-**L1** (racemic version) (12 mg, 6 mol%, 0.024 mmol) were suspended in dry toluene (1 mL), stirred during 1 hour at r.t. and added to the reaction vial containing $B_2(pin)_2$ (200 mg, 2.0 equiv, 0.4 mmol) and NaO^{t}Bu (796 mg, 2.0 equiv, 0.8 mmol) in dry toluene (2.2 mL) to afford a red suspension. The corresponding alkyne (2.0 equiv, 0.8 mmol) and allylic substrate (1.0 equiv, 0.4 mmol) were added to the mixture which was stirred at 30 °C. The mixture was stirred for 16 h at 30 °C. After this time, the mixture was diluted in CH_2Cl_2 (10 mL) and washed with NH_4Cl (20 mL). Then the aqueous layer was extracted with CH_2Cl_2 (2x10 mL). The organic layer was dried, filtered and the solvents were evaporated under vacuum. The final product was purified by silica gel flash column chromatography.

Note: Alternatively, racemic products can be synthesized using IMesCuCl (8 mg, 5 mol%, 0.02 mmol) as catalyst.

GP-1B. Cu-catalyzed allylboration of alkynes (with slow addition of allylic substrate): CuCl (2.0 mg, 5 mol%, 0.02 mmol) and **L1**/*rac*-**L1** (12 mg, 6 mol%, 0.024 mmol) were suspended in dry toluene (1 mL), stirred during 1 hour at r.t. and added to the reaction vial containing $B_2(pin)_2$ (200 mg, 2.0 equiv, 0.4 mmol) and NaO^{t}Bu (796 mg, 2.0 equiv., 0.8 mmol) in dry toluene (1.2 mL) to afford a red suspension. The corresponding alkyne (2.0 equiv., 0.8 mmol) was added to the reaction mixture and stirred at 30 ºC. A solution of allylic substrate (1.0 equiv, 0.4 mmol) in dry toluene (1 mL) was added to the mixture over 5 h with a syringe pump. The mixture was stirred for additional 11 h at 30 °C. After this time, the mixture was diluted in CH_2Cl_2 (10 mL) and washed with NH_4Cl (20 mL). Then the aqueous layer was extracted with CH_2Cl_2 (2x10 mL). The organic layer was dried, filtered and the solvents were evaporated under vacuum. The final product was purified by silica gel flash column chromatography.

General procedure for Cu-catalyzed borylcupration-1,4-B migration -iodination[2]

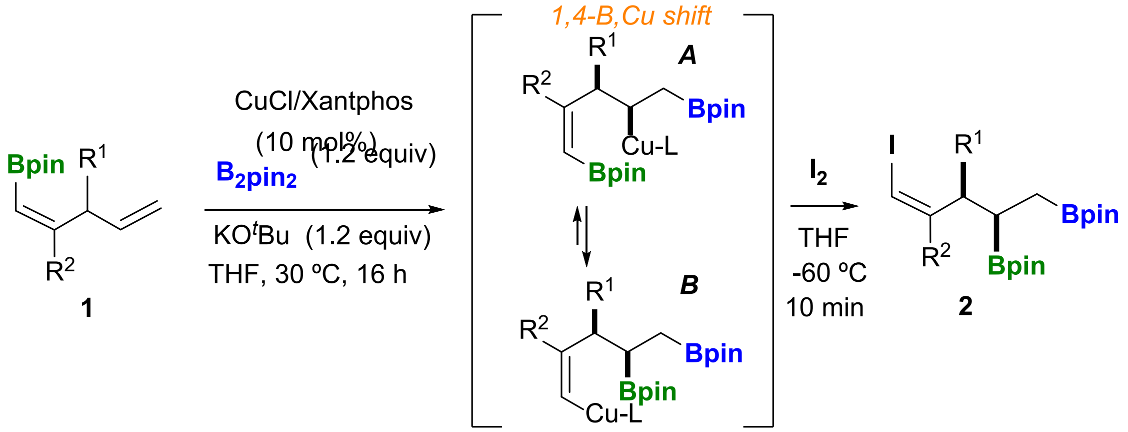

The following reagents, CuCl (0.98 mg, 10 mol%, 0.01 mmol), bis(pinacolato)diboron (60.9 mg, 1.2 equiv, 0.24 mmol), and Xantphos (138.8 mg, 10 mol%, 0.01 mmol) were placed in a flamed Schlenk-tube. The Schlenk-tube was sealed with a screw cap containing a Teflon-coated rubber septum. The Schlenk-tube was connected to a vacuum/nitrogen manifold through a needle, evacuated, and backfilled with nitrogen and THF (0.24 mL, 1M). A solution of the base KO*t*Bu (26.9 mg, 1.2 equiv, 0.24 mmol) in THF (0.24 mL, 1M) was added to the reaction media through the rubber septum. Then, the borylated (*E*)-skipped diene (1 equiv, 0.2 mmol) in THF (0.2 mL, 1M) was added dropwise at 30 °C and stirred for 16 hours. Subsequently, iodine (101.5 mg, 2 equiv, 0.4 mmol) was dissolved in 2 mL of THF in a second oven-dried Schlenk-tube. The reaction mixture was then kept at -60 °C while the iodine solution was passed through a Teflon tubing. The cooling bath was removed, and the temperature was allowed to rise to room temperature. After 10 minutes at room temperature, a mixture of 10 mL of saturated sodium bisulphite is added with vigorous stirring and washed with Et_2O (3 x 15 mL). The organic extracts were dried over anhydrous magnesium sulphate and then concentrated under vacuum. The NMR yield was calculated through comparison to an internal standard (naphthalene). The crude residue was purified by silica gel flash chromatography to obtain the desired product.

Specific example for Cu-catalysed borylcupration-1,4-B migration of (*R*)-**1a**

CuCl (0.98 mg, 10 mol%, 0.01 mmol), bis(pinacolato)diboron (60.9 mg, 1.2 equiv, 0.24 mmol), and Xantphos (138.8 mg, 10 mol%, 0.01 mmol) were placed in a flamed Schlenk-tube. The Schlenk-tube was sealed with a screw cap containing a Teflon-coated rubber septum. The Schlenk-tube was connected to a vacuum/nitrogen manifold through a needle, evacuated, and backfilled with nitrogen and THF (0.24 ml, 1M). A solution of KO*t*Bu (26.9 mg, 1.2 equiv, 0.24 mmol) in THF (0.24 ml, 1M) was added to the reaction media through the rubber septum. Then, a solution of (*R*)-(*E*)-2-(2,3-diphenylpenta-1,4-dien-1-yl)-4,4,5,5-tetramethyl-1,3,2-dioxaborolane (*R*)-**1a** (70 mg,1 equiv, 0.2 mmol) in THF (0.2 ml, 1M) was added dropwise at 30 °C and stirred for 16 hours. Subsequently, iodine (101.5 mg, 2 equiv, 0.4 mmol) was dissolved in 2 mL of THF in a second oven-dried Schlenk-tube. The reaction mixture was then kept at -60 °C while the iodine solution was passed through a Teflon tubing. The cooling bath was removed, and the

temperature was allowed to rise to room temperature. After 10 minutes at room temperature, a mixture of 10 mL of saturated sodium bisulfite is added with vigorous stirring and washed with Et_2O (3 x 15 mL). The organic extracts were dried over anhydrous magnesium sulphate and then concentrated under vacuum. The NMR yield was calculated through comparison to an internal standard (naphthalene). The crude residue was purified by silica gel flash chromatography to obtain the desired product (*R,R*)-**2** (106 mg, 88% yield).

General procedure for intramolecular Suzuki-Miyaura cross-coupling[3]

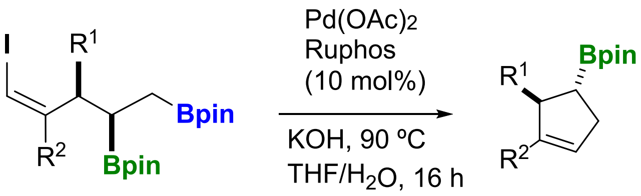

In a flamed Schlenk-tube equipped with a magnetic stir bar, $Pd(OAc)_2$ (4.5 mg, 10 mol%), RuPhos (9.33 mg, 10 mol%), and the substrate (0.2 mmol) were added in THF (2 mL). Under an argon atmosphere, KOH (33.6 mg, 0.6 mmol, 3 equiv) and deoxygenated water (0.2 mL) were added. The reaction mixture was stirred at 90 °C for 16 h. After that, the mixture was filtered, and the organic extracts were concentrated under vacuum, and the NMR yield was calculated through comparison to an internal standard (naphthalene). The crude residue was purified by silica gel flash chromatography to obtain the desired product.

Specific example for intramolecular Suzuki-Miyaura cross-coupling of (*R,R*)-**2**

In a flamed Schlenk-tube equipped with a magnetic stir bar, $Pd(OAc)_2$ (4.5 mg, 10 mol%), RuPhos (9.33 mg, 10 mol%), and the substrate (*R,R*)-**2** (120 mg, 0.2 mmol) were added in THF (2 mL). Under an argon atmosphere, KOH (33.6 mg, 0.6 mmol, 3 equiv) and deoxygenated water (0.2 mL) were added. The reaction mixture was stirred at 90 °C for 16 h. After that, the mixture was filtered, and the organic extracts were concentrated under vacuum, and the NMR yield was calculated through comparison to an internal standard (naphthalene). The crude residue was purified by silica gel flash chromatography to obtain the desired product (*R,R*)-**12** (40 mg, 59% yield).

General procedure for oxidation of C-B bond

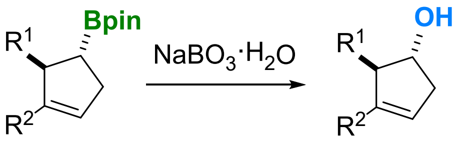

In an opened-air flask, charged with a magnetic stir bar, were added the corresponding cyclopentenes (0.1 mmol, 1 equiv), $NaBO_3 \cdot H_2O$ (0.3 mmol, 3 equiv), THF (2 mL) and distilled water (1 mL). The reaction was closed with a septum equipped with a needle to avoid

overpressures and was stirred for 16 h at room temperature. After this time, the mixture was extracted with Et_2O (3 x 15 mL), the organic layer was dried with anhydrous magnesium sulfate, filtered, and the solvents were evaporated. The crude residue was purified by silica gel chromatography to obtain the corresponding cyclopentenol product.

General procedure for the cyclopropanation reaction

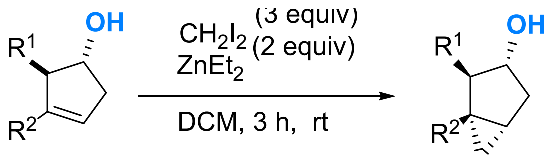

To a solution of the cyclopentenol product (0.2 mmol, 1.0 equiv) in anhydrous DCM (2.0 mL, 0.1 M) was added $ZnEt_2$ (1.0 M in hexane, 0.4 mL, 0.4 mmol, 2.0 equiv) at 0 °C. After stirring for 10 min, CH_2I_2 (161 mg, 0.6 mmol, 3.0 equiv) was added. The mixture was stirred at room temperature, and a white precipitate was gradually generated. After 3 h, the mixture was quenched with saturated aqueous NH_4Cl (8 mL) and extracted with EtOAc (3 × 10 mL). The combined organic layers were dried with Na_2SO_4, filtered over Celite, and concentrated in vacuo. The residue was purified by flash column chromatography on silica gel (eluting with petroleum ether/ethyl acetate = 20/1) to give the corresponding product.

General method for the epoxidation reaction

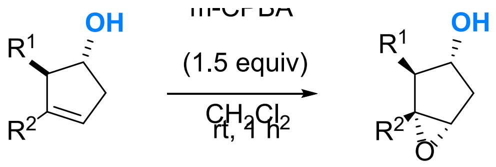

A solution of the cyclopentenol product (0.55 mmol, 1.0 equiv) and *m*-CPBA (0.85 mmol, 1.5 equiv) were diluted in CH_2Cl_2 (5.5 mL, 0.1 M). The reaction was stirred at room temperature for 1 h. After this time, the reaction was quenched with the addition of sat. aq. $NaHCO_3$ (2 mL). The reaction mixture was extracted with CH_2Cl_2 (2x 2 mL). The combined organic layer was dried over Na_2SO_4, filtered and concentrated under reduced pressure. The residue was purified by flash column chromatography on silica gel (eluting with *n*-hexane/ ethyl acetate = 90/10) to give the corresponding product.

General procedure for the aziridination reaction

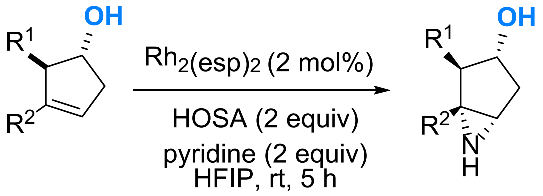

A solution of the cyclopentenol product (0.1 mmol, 1.0 equiv) was dissolved in HFIP (0.26 mL, 0.4 M) at room temperature. Pyridine (0.2 mmol, 2.0 equiv), HOSA (0.2 mmol, 2.0 equiv.) and $Rh_2(esp)_2$ (Du Bois' catalyst, 0.002 mmol, 2 mol%) were added sequentially and the reaction was stirred for 6 h at room temperature. After this time, the reaction was quenched at rt with the addition of sat. aq. Na_2CO_3 (1 mL) and distilled water (2 mL). The reaction mixture was extracted with CH_2Cl_2 (4 mL x 2). The combined organic layer was dried over Na_2SO_4, filtered and concentrated under reduced pressure. The residue was purified by flash column chromatography (eluting with *n*-hexane/ethyl acetate = 60/40) to give the corresponding product.

Characterization data for (*E*)-skipped dienes

(*E*)-2-(2,3-diphenylpenta-1,4-dien-1-yl)-4,4,5,5-tetramethyl-1,3,2-dioxaborolane ((±)-1a)

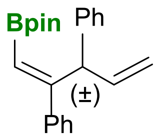

1a

Following GP-1A using IMesCuCl cinnamyl bromide and phenylacetylene, (±)-**1a** was was purified by flash chromatography using a mixture of *n*-hexane and dichloromethane (70:30) as eluent. The product was isolated in 52% yield as a white solid.

^{1}H NMR ($CDCl_3$, 300 MHz) δ 7.38 – 7.26 (m, 4H), 7.24 – 7.12 (m, 4H), 7.11 – 7.02 (m, 2H), 6.21 (ddd, *J* = 17.1, 10.2, 8.6 Hz, 1H), 5.70 (s, 1H), 5.59 (d, *J* = 8.6 Hz, 1H), 5.29 (dd, *J* = 17.1, 2.0 Hz, 1H), 5.20 (dd, *J* = 10.2, 2.0 Hz, 1H), 1.30 (s, 6H), 1.29 (s, 6H). Data in accordance with literature.[1]

(*R*,*E*)-2-(2,3-diphenylpenta-1,4-dien-1-yl)-4,4,5,5-tetramethyl-1,3,2-dioxaborolane ((*R*)-1a)

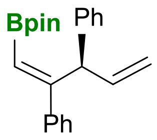

(*R*)-1a

Following GP-1B using cinnamyl diethyl phosphate[2] and phenylacetylene, (*R*)-**1a** was purified by flash chromatography using a mixture of *n*-hexane and dichloromethane (70:30) as eluent. The product was isolated in 45% yield as a white solid in a 96:4 enantiomeric ratio. Data in accordance with literature.[1]

Enantiomeric purity was determined by chiral uHPLC analysis [Chiralpak i-amylose-3, T_{oven}: 40 °C, flow: 1 mL/min; 1% IPA, 99% *n*-Hexane, minor enantiomer t_R = 3.74 min, major enantiomer t_R = 3.89 min].

(*E*)-4,4,5,5-Tetramethyl-2-(3-phenyl-2-(*o*-tolyl)penta-1,4-dien-1-yl)-1,3,2-dioxaborolane ((±)-1b)

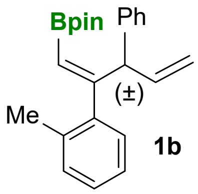

1b

Following GP-1A using cinnamyl bromide and 2-ethynyltoluene, (±)-**1b** was purified by flash chromatography using a mixture of *n*-hexane and dichloromethane (70:30) as eluent. The product was isolated in 62% yield as a white solid.

^{1}H NMR ($CDCl_3$, 300 MHz) δ 7.23 – 7.12 (m, 5H), 7.10 – 7.00 (m, 2H), 6.94 (t, J = 7.1 Hz, 1H), 6.61 (d, J = 7.6 Hz, 1H), 6.11 (dt, J = 17.1, 9.6 Hz, 1H), 5.49 (d, J = 9.2 Hz, 1H), 5.28 (d, J = 16.5 Hz, 2H), 5.22 (s, 1H), 5.13 (d, J = 10.0 Hz, 1H), 1.88 (s, 3H), 1.35 (s, 12H).

^{13}C NMR ($CDCl_3$, 75 MHz) δ 164.3, 142.6, 142.2, 139.0, 135.6, 130.0, 128.5, 128.3, 128.2, 126.8, 126.4, 124.4, 116.5, 83.4, 55.3, 25.1, 20.1.

^{11}B NMR ($CDCl_3$, 160 MHz) δ 29.9.

HRMS-(APCI) for $C_{24}H_{30}BO_2$ $[M+H]^+$: calculated: 361.2333; found: 361.2329.

(*R,E*)-4,4,5,5-tetramethyl-2-(3-phenyl-2-(o-tolyl)penta-1,4-dien-1-yl)-1,3,2-dioxaborolane ((*R*)-1b)

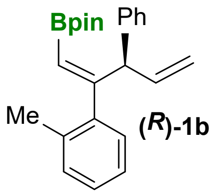

Following GP-1A using cinnamyl bromide and 2-ethynyltoluene, (*R*)-**1b** was purified by flash chromatography using a mixture of *n*-hexane and dichloromethane (70:30) as eluent. The product was isolated in 34% yield as a white solid.

Specific rotation: $[\alpha]_D^{21}$ +2.3 (*c*=0.70, $CHCl_3$) for enantiomerically enriched sample of 89:11 e.r.

Enantiomeric purity was determined by chiral uHPLC analysis [Chiralpak i-cellulose-3, T_{oven}: 40 °C, flow: 1 mL/min; 1% IPA, 99% *n*-Hexane, minor enantiomer t_R = 3.62 min, major enantiomer t_R = 4.07 min].

(*E*)-2-(2-(4-bromophenyl)-3-phenylpenta-1,4-dien-1-yl)-4,4,5,5-tetramethyl-1,3,2-dioxaborolane ((±)-1c)

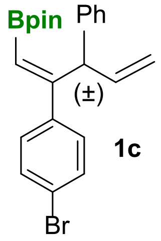

Following GP-1A using cinnamyl bromide and 4-bromophenylacetylene, (±)-**1c** was purified by flash chromatography using a mixture of hexane and dichloromethane (70:30) as eluent. The product was isolated in 40% yield as a white solid.

^{1}H NMR ($CDCl_3$, 300 MHz) δ 7.33 – 7.26 (m, 6H), 7.25 – 7.18 (m, 1H), 6.92 (d, *J* = 8.6 Hz, 2H), 6.16 (dt, *J* = 18.1, 9.3 Hz, 1H), 5.67 (s, 1H), 5.58 (d, *J* = 8.5 Hz, 1H), 5.29 (d, *J* = 17.2 Hz, 1H), 5.24 – 5.17 (m, 1H), 1.31 (s, 6H), 1.30 (s, 6H).

^{13}C NMR ($CDCl_3$, 75 MHz) δ 162.4, 142.4, 142.0, 138.6, 131.0, 129.8, 128.5, 128.0, 126.4, 121.7, 117.7, 83.5, 53.3, 25.1.

^{11}B NMR ($CDCl_3$, 160 MHz) δ 30.4.

HRMS-(APCI) for $C_{23}H_{27}BBrO_2$ $[M+H]^+$: calculated: 425.1282; found: 425.1294.

(*E*)-4,4,5,5-tetramethyl-2-(3-phenyl-2-(thiophen-3-yl)penta-1,4-dien-1-yl)-1,3,2-dioxaborolane ((±)-1d)

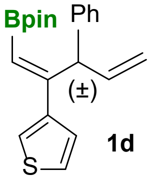

1d

Following GP-1A using cinnamyl bromide and 3-ethynylthiophene, (±)-**1d** was purified by flash chromatography using a mixture of hexane and dichloromethane (70:30) as eluent. The product was isolated in 56% yield as a colorless oil.

^{1}H NMR ($CDCl_3$, 300 MHz) δ 7.40 – 7.28 (m, 4H), 7.25 – 7.15 (m, 1H), 7.09 (dd, *J* = 5.1, 2.9 Hz, 1H), 6.98 (dd, *J* = 5.0, 1.4 Hz, 1H), 6.88 (dd, *J* = 3.0, 1.4 Hz, 1H), 6.29 (ddd, *J* = 17.2, 10.1, 8.5 Hz, 1H), 5.87 (s, 1H), 5.63 (d, *J* = 8.5 Hz, 1H), 5.30 (ddd, *J* = 17.1, 1.9, 1.1 Hz, 1H), 5.22 (dd, *J* = 10.2, 1.9 Hz, 1H), 1.29 (s, 6H), 1.28 (s, 6H).

^{13}C NMR ($CDCl_3$, 75 MHz) δ 157.6, 142.9, 138.8, 128.4, 127.9, 127.7, 126.2, 124.4, 123.5, 117.8, 83.3, 53.0, 25.0, 25.0.

^{11}B NMR ($CDCl_3$, 160 MHz) δ 30.1.

HRMS-(APCI) for $C_{21}H_{26}BO_2S$ $[M+H]^+$: calculated: 353.1741; found: 353.1753.

(*R*,*E*)-4,4,5,5-tetramethyl-2-(3-phenyl-2-(thiophen-3-yl)penta-1,4-dien-1-yl)-1,3,2-dioxaborolane ((*R*)-1d)

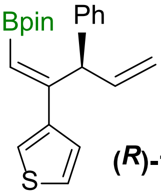

(*R*)-1d

Following GP-1A using cinnamyl bromide and 3-ethynylthiophene, (*R*)-**1d** was purified by flash chromatography using a mixture of hexane and dichloromethane (70:30) as eluent. The product was isolated in 48% yield as a colorless oil.

Specific rotation: $[\alpha]_D^{21}$ +12.6 (*c*=0.52, $CHCl_3$) for enantiomerically enriched sample of 92:8 e.r..

Enantiomeric purity was determined by chiral uHPLC analysis [Chiralpak i-celulose-5, Toven: 40 °C, flow: 1 mL/min; 1% IPA, 99% *n*-Hexane, minor enantiomer tR = 3.59 min, major enantiomer tR = 3.94 min].

(*E*)-4,4,5,5-tetramethyl-2-(2-phenyl-3-(4-(trifluoromethyl)phenyl)penta-1,4-dien-1-yl)-1,3,2-dioxaborolane ((±)-1e)

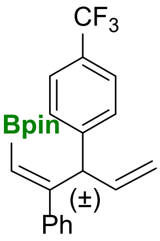

1e

Following GP-1B using cinnamyl bromide and phenylacetylene, (±)-**1e** was purified by flash chromatography using a mixture of *n*-hexane and dichloromethane (70:30) as eluent. The product was isolated in 51% yield as a colorless oil.

^{1}H NMR ($CDCl_3$, 300 MHz) δ 7.53 (d, *J* = 8.3 Hz, 2H), 7.43 (d, *J* = 8.5 Hz, 2H), 7.24 – 7.12 (m, 3H), 7.12 – 7.00 (m, 2H), 6.19 (ddd, *J* = 17.0, 10.0, 8.6 Hz, 1H), 5.74 (s, 1H), 5.57 (d, *J* = 8.6 Hz, 1H), 5.38 – 5.16 (m, 2H), 1.27 (s, 6H), 1.26 (s, 6H). Data in accordance with literature.[1]

(*R*,*E*)-4,4,5,5-tetramethyl-2-(2-phenyl-3-(4-(trifluoromethyl)phenyl)penta-1,4-dien-1-yl)-1,3,2-dioxaborolane ((*R*)-1e)

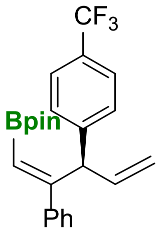

(*R*)-1e

Following GP-1B using cinnamyl bromide and phenylacetylene, (*R*)-**1e** was purified by flash chromatography using a mixture of *n*-hexane and dichloromethane (70:30) as eluent. The product was isolated in 53% yield as a colorless oil in a 93:7 enantiomeric ratio. Data in accordance with literature.[1] Enantiomeric purity was determined by chiral uHPLC analysis [Chiralpak i-amylose-3, T_{oven}: 40 °C, flow: 1 mL/min; 1% IPA, 99% *n*-Hexane, minor enantiomer t_R = 3.23 min, major enantiomer t_R = 3.47 min].

(*E*)-4,4,5,5-tetramethyl-2-(3-methyl-2-phenylpenta-1,4-dien-1-yl)-1,3,2-dioxaborolane ((±)-1f)

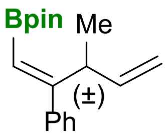

1f

Following GP-1A using crotyl bromide and phenylacetylene, (±)-**1f** was purified by flash chromatography using a mixture of *n*-hexane and dichloromethane (70:30) as eluent. The product was isolated in 75% yield as a colorless oil.

^{1}H NMR ($CDCl_3$, 300 MHz) δ 7.31 – 7.22 (m, 5H), 6.04 (ddd, J = 16.8, 10.3, 6.8 Hz, 1H), 5.43 (s, 1H), 5.12 (d, J = 16.8 Hz, 1H), 5.05 (d, J = 10.3 Hz, 1H), 4.20 (p, J = 6.8 Hz, 1H), 1.31 (s, 12H), 1.14 (d, J = 6.8 Hz, 3H). Data in accordance with literature.[1]

(*S*,*E*)-4,4,5,5-tetramethyl-2-(3-methyl-2-phenylpenta-1,4-dien-1-yl)-1,3,2-dioxaborolane ((*S*)-1f)

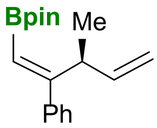

(*S*)-1f

Following GP-1A using crotyl bromide and phenylacetylene, (*S*)-**1f** was purified by flash chromatography using a mixture of *n*-hexane and dichloromethane (70:30) as eluent. The product was isolated in 62% yield as a colorless oil in a 94.5:4.5 enantiomeric ratio. Data in accordance with literature.[1]

Enantiomeric purity was determined by chiral uHPLC analysis [Chiralpak i-amylose-3, T_{oven}: 40 °C, flow: 1 mL/min; 1% IPA, 99% *n*-Hexane, minor enantiomer t_R = 3.34 min, major enantiomer t_R = 3.52 min].

(*E*)-4,4,5,5-tetramethyl-2-(3-methyl-2-(*o*-tolyl)penta-1,4-dien-1-yl)-1,3,2-dioxaborolane ((±)-1g)

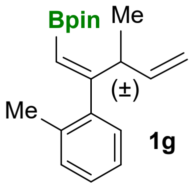

1g

Following GP-1A using crotyl bromide and 2-ethynyltoluene, (±)-**1g** was purified by flash chromatography using a mixture of hexane and dichloromethane (70:30) as eluent. The product was isolated in 41% yield as a colorless oil.

^{1}H NMR ($CDCl_3$, 300 MHz) δ 7.19 – 7.11 (m, 2H), 7.13 – 7.01 (m, 1H), 7.02 – 6.96 (m, 1H), 5.84 (ddd, *J* = 17.3, 10.2, 7.2 Hz, 1H), 5.16 (s, 1H), 5.04 (dt, *J* = 17.2, 1.6 Hz, 1H),

4.94 (dt, J = 10.2, 1.5 Hz, 1H), 4.12 (p, J = 7.1 Hz, 1H), 2.23 (s, 3H), 1.32 (s, 12H), 1.07 (d, J = 7.0 Hz, 3H).

^{13}C NMR ($CDCl_3$, 75 MHz) δ 166.5, 143.2, 142.8, 134.9, 130.1, 128.1, 126.6, 124.5, 113.4, 83.1, 43.6, 25.0, 25.0, 22.8, 18.8.

^{11}B NMR ($CDCl_3$, 160 MHz) δ 30.1.

HRMS-(APCI) for $C_{19}H_{28}BO_2$ $[M+H]^+$: calculated: 299.2177; found: 299.2171.

(*E*)-4,4,5,5-tetramethyl-2-(2-phenyl-3-vinylhex-1-en-1-yl)-1,3,2-dioxaborolane ((±)-**1h)**

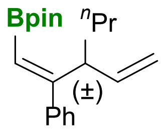

1h

Following GP-1A using (*E*)-diethyl hex-2-en-1-yl phosphate[2] and phenylacetylene, (±)-**1h** was purified by flash chromatography using a mixture of *n*-hexane and dichloromethane (70:30) as eluent. The product was isolated in 59% yield as a colorless oil.

^{1}H NMR ($CDCl_3$, 300 MHz) δ 7.37 – 7.17 (m, 5H), 5.96 (ddd, J = 17.2, 10.3, 6.9 Hz, 1H), 5.43 (s, 1H), 5.16 (dt, J = 17.3, 1.7 Hz, 1H), 5.05 (ddd, J = 10.3, 1.9, 1.4 Hz, 1H), 4.07 (q, J = 7.3 Hz, 1H), 1.53 – 1.41 (m, 2H), 1.30 (s, 12H), 1.27 – 1.21 (m, 2H), 0.85 (t, J = 7.3 Hz, 3H).

^{13}C NMR ($CDCl_3$, 75 MHz) δ 165.9, 143.9, 142.3, 127.8, 127.7, 127.2, 114.5, 83.1, 47.9, 35.0, 25.0, 20.7, 14.0.

^{11}B NMR ($CDCl_3$, 160 MHz) δ 30.1.

HRMS-(APCI) for $C_{20}H_{30}BO_2$ $[M+H]^+$: calculated: 313.2333; found: 313.2338.

(*S*,*E*)-4,4,5,5-tetramethyl-2-(2-phenyl-3-vinylhex-1-en-1-yl)-1,3,2-dioxaborolane ((*S*)-1h)

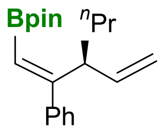

(*S*)-1h

Following GP-1A using (*E*)-diethyl hex-2-en-1-yl phosphate[2] and phenylacetylene, (*S*)-**1h** was purified by flash chromatography using a mixture of *n*-hexane and dichloromethane (70:30) as eluent. The product was isolated in 69% yield as a colorless oil.

Specific rotation: $[\alpha]_D^{21}$ -3.8 (c=0.77, $CHCl_3$) for enantiomerically enriched sample of 98:2 e.r.

Enantiomeric purity was determined by chiral uHPLC analysis [Chiralpak i-amylose-3, T_{oven}: 40 °C, flow: 1 mL/min; 1% IPA, 99% *n*-Hexane, minor enantiomer t_R = 3.17 min, major enantiomer t_R = 3.30 min].

(*E*)-2-(3-isopropyl-2-phenylpenta-1,4-dien-1-yl)-4,4,5,5-tetramethyl-1,3,2-dioxaborolane ((±)-**1i)**

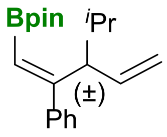

1i

Following GP-1A using (*E*)-diethyl (4-methylpent-2-en-1-yl) phosphate[2] and phenylacetylene, (±)-**1i** was purified by flash chromatography using a mixture of *n*-hexane and dichloromethane (70:30) as eluent. The product was isolated in 65% yield as a colorless oil.

^{1}H NMR ($CDCl_3$, 300 MHz) δ 7.36 – 7.22 (m, 5H), 5.96 (ddd, *J* = 17.0, 10.1, 8.8 Hz, 1H), 5.44 (s, 1H), 5.21 (ddd, *J* = 17.1, 2.2, 1.0 Hz, 1H), 5.06 (ddd, *J* = 10.1, 2.2, 0.8 Hz, 1H), 3.66 (t, *J* = 9.7 Hz, 1H), 1.88 – 1.66 (m, 1H), 1.32 (s, 12H), 0.92 (d, *J* = 4.0 Hz, 3H), 0.89 (d, *J* = 4.0 Hz, 3H).

^{13}C NMR ($CDCl_3$, 75 MHz) δ 165.7, 144.4, 141.3, 127.8, 127.1, 115.8, 83.1, 57.1, 30.0, 25.1, 24.9, 21.5, 21.3.

^{11}B NMR ($CDCl_3$, 160 MHz) δ 30.3.

HRMS-(APCI) for $C_{20}H_{30}BO_2$ $[M+H]^+$: calculated: 313.2333; found: 313.2325.

(*S*,*E*)-2-(3-isopropyl-2-phenylpenta-1,4-dien-1-yl)-4,4,5,5-tetramethyl-1,3,2-dioxaborolane ((*S*)-1i)

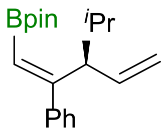

(*S*)-1i

Following GP-1A using (*E*)-diethyl (4-methylpent-2-en-1-yl) phosphate[2] and phenylacetylene, (*S*)-**1i** was purified by flash chromatography using a mixture of *n*-hexane and dichloromethane (70:30) as eluent. The product was isolated in 58% yield as a colorless oil.

Specific rotation: $[\alpha]_D^{21}$ -11.9 (*c*=0.70, $CHCl_3$) for enantiomerically enriched sample of 95.5:4.5 e.r.

Enantiomeric purity was determined by chiral uHPLC analysis [Chiralpak i-celulose-5, Toven: 40 °C, flow: 1 mL/min; 0.5% IPA, 99.5% *n*-Hexane, minor enantiomer tR = 3.80 min, major enantiomer tR = 4.06 min].

(*E*)-4,4,5,5-tetramethyl-2-(5-methyl-2-phenyl-3-vinylhex-1-en-1-yl)-1,3,2-dioxaborolane ((±)-1j)

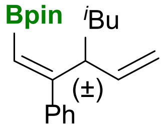

1j

Following GP-1A using (*E*)-diethyl (5-methylhex-2-en-1-yl) phosphate[2] and phenylacetylene, (±)-**1j** was purified by flash chromatography using a mixture of *n*-hexane and dichloromethane (70:30) as eluent. The product was isolated in74% yield as a colorless oil.

^{1}H NMR ($CDCl_3$, 300 MHz) δ 7.35 – 7.22 (m, 5H), 5.98 (ddd, *J* = 17.2, 10.2, 7.0 Hz, 1H), 5.44 (s, 1H), 5.21 (dt, *J* = 17.3, 1.7 Hz, 1H), 5.08 (dt, *J* = 10.3, 1.6 Hz, 1H), 4.24 (q, *J* = 7.9 Hz, 1H), 1.52 (hept, *J* = 6.7 Hz, 1H), 1.39 – 1.30 (m, 14H), 0.90 (d, *J* = 6.5 Hz, 3H), 0.82 (d, *J* = 6.5 Hz, 3H).

^{13}C NMR ($CDCl_3$, 75 MHz) δ 166.4, 143.8, 142.4, 127.8, 127.6, 127.1, 114.5, 83.1, 46.0, 41.9, 25.6, 25.0, 25.0, 22.8, 22.6.

^{11}B NMR ($CDCl_3$, 160 MHz) δ 30.3.

HRMS-(APCI) for $C_{21}H_{32}BO_2$ $[M+H]^+$: calculated: 327.2490; found: 327.2483.

Characterization data for products isolated from Cu-catalyzed borylcupration-1,4-B migration-iodination

(*E*)-2,2'-(5-iodo-3,4-diphenylpent-4-ene-1,2-diyl)bis(4,4,5,5-tetramethyl-1,3,2-dioxaborolane) ((±)-2)

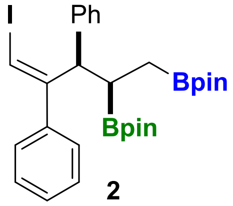

The product (±)-**2** was purified by flash chromatography using a mixture of petroleum ether and diethyl ether (100:10) as eluent. It was isolated as a colorless oil (106 mg, 88%) as a mixture of both diastereoisomers (14:1). Product (*R*)-**2** (108 mg, 90%).

Major diastereoisomer: ^1H NMR ($CDCl_3$, 400 MHz) δ 7.22 – 7.15 (m, 1H), 7.15 – 7.08 (m, 5H), 7.06 (m, 2H), 6.71 – 6.65 (m, 2H), 6.21 (s, 1H), 4.32 (d, *J* = 12.1 Hz, 1H), 1.78 – 1.65 (m, 1H), 1.26 (s, 6H), 1.25 (s, 6H), 1.10 (m, 2H), 0.93 (s, 6H), 0.87 (s, 6H). *Minor diastereoisomer*: ^1H NMR ($CDCl_3$, 400 MHz) δ 7.22 – 7.15 (m, 1H), 7.15 – 7.08 (m, 5H), 7.06 (m, 2H), 6.71 – 6.65 (m, 2H), 6.03 (s, 1H), 4.46 (d, *J* = 12.3 Hz, 1H), 1.78 – 1.65 (m, 1H), 1.26 (s, 6H), 1.25 (s, 6H), 1.10 (m, 2H), 0.93 (s, 6H), 0.87 (s, 6H).

Major diastereoisomer: ^{13}C NMR ($CDCl_3$, 100 MHz) δ 154.0, 141.5, 139.5, 129.3, 129.1, 127.5, 127.2, 127.2, 126.5, 83.1, 82.8, 81.8, 56.5, 25.0, 24.8, 24.5, 24.3.

^{11}B NMR ($CDCl_3$, 128.3 MHz) δ 32.9.

HRMS-(ESI+) for $C_{29}H_{40}B_2IO_4$ $[M+H]^+$: calculated: 601.2154; found: 601.2152.

(*E*)-2,2'-(5-iodo-3-phenyl-4-(o-tolyl)pent-4-ene-1,2-diyl)bis(4,4,5,5-tetramethyl-1,3,2-dioxaborolane) ((±)-3)

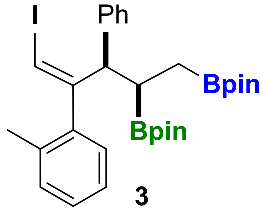

The product (±)-**3** was purified by flash chromatography using a mixture of petroleum ether and diethyl ether (100:10) as eluent. It was isolated as a colorless oil (25 mg, 20%) as a mixture of both diastereoisomers (16:1). Product (*R*)-**3** (20 mg, 16%).

Major diastereoisomer: ^1H NMR ($CDCl_3$, 400 MHz) δ 7.16 – 6.97 (m, 5H), 6.97 – 6.84 (m, 4H), 6.13 (s, 1H), 4.29 (d, *J* = 12.1 Hz, 1H), 1.85 (m, 1H), 1.27 (s, 12H), 1.26 (s, 3H), 1.20 – 1.17 (m, 2H), 0.86 (s, 6H), 0.84 (s, 6H). *Minor diastereoisomer*: ^1H NMR ($CDCl_3$, 400 MHz) δ 7.16 – 6.97 (m, 5H), 6.97 – 6.84 (m, 4H), 5.95 (s, 1H), 4.52 – 4.40 (m, 1H), 1.85 (m, 1H), 1.44 – 1.38 (m, 1H), 1.27 (s, 12H), 1.26 (s, 3H), 1.20 – 1.17 (m, 2H), 0.86 (s, 6H), 0.84 (s, 6H).

Major diastereoisomer:^{13}C NMR ($CDCl_3$, 100 MHz) δ 151.3, 141.2, 138.3, 137.8, 129.8, 129.2, 127.6, 127.3, 126.5, 124.5, 83.1, 82.8, 81.9, 57.4, 25.0, 24.8, 24.5, 24.3, 18.6.

^{11}B NMR ($CDCl_3$, 128.3 MHz) δ 33.8.

HRMS-(ESI+) for $C_{30}H_{42}B_2IO_4$ $[M+H]^+$: calculated: 615.2308; found: 615.2309

(*E*)-2,2'-(4-(4-bromophenyl)-5-iodo-3-phenylpent-4-ene-1,2-diyl)bis(4,4,5,5-tetramethyl-1,3,2-dioxaborolane) ((±)-4)

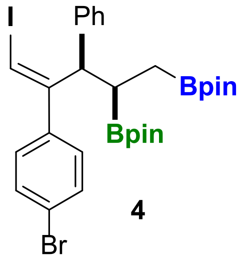

4

The product (±)-**4** was purified by flash chromatography using a mixture of petroleum ether and diethyl ether (100:10) as eluent. It was isolated as a colorless oil (103 mg, 76%) as a mixture of both diastereoisomers (8:1).

Major diastereoisomer: ^{1}H NMR ($CDCl_3$, 400 MHz) δ 7.39 (dd, *J* = 8.0, 1.5 Hz, 2H), 7.27 (m, 3H), 7.23 – 7.15 (m, 2H), 6.71 – 6.66 (m, 2H), 6.36 (s, 1H), 4.45 (d, *J* = 12.1 Hz, 1H), 1.81 – 1.70 (m, 1H), 1.39 (s, 6H), 1.39 (s, 6H), 1.39 (m, 2H), 1.07 (s, 6H), 1.01 (s, 6H). *Minor diastereoisomer:* ^{1}H NMR ($CDCl_3$, 400 MHz) δ 7.46 – 7.41 (m, 2H), 7.27 (m, 3H), 7.13 – 7.08 (m, 2H), 6.93 (d, *J* = 8.4 Hz, 2H), 6.17 (s, 1H), 4.59 (d, *J* = 12.3 Hz, 1H), 1.81 – 1.70 (m, 1H), 1.49 (s, 6H), 1.44 (s, 6H), 1.32 (s, 6H), 1.32 (s, 6H), 1.32 (m, 2H).

Major diastereoisomer:^{13}C NMR ($CDCl_3$, 100 MHz) δ 153.0, 141.2, 138.4, 131.0, 130.4, 129.0, 127.6, 126.7, 121.6, 83.1, 82.9, 82.2, 56.3, 25.0, 24.8, 24.5, 24.3.

^{11}B NMR ($CDCl_3$, 128.3 MHz) δ 33.2.

HRMS-(ESI+) for $C_{29}H_{39}B_2BrIO_4$ $[M+H]^+$: calculated: 679.1271; found: 679.1223.

(*E*)-2,2'-(5-iodo-3-phenyl-4-(thiophen-3-yl)pent-4-ene-1,2-diyl)bis(4,4,5,5-tetramethyl-1,3,2-dioxaborolane) ((±)-5)

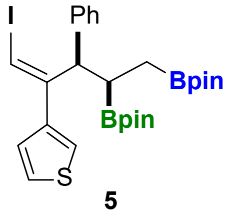

5

The product (±)-**5** was purified by flash chromatography using a mixture of petroleum ether and diethyl ether (100:10) as eluent. It was isolated as a colorless oil (50 mg, 41%) as a mixture of both diastereoisomers (5:1).

Major diastereoisomer: ^{1}H NMR ($CDCl_3$, 400 MHz) δ 7.20 – 7.10 (m, 5H), 7.10 – 7.05 (m, 1H), 6.78 (dd, *J* = 3.0, 1.3 Hz, 1H), 6.57 (dd, *J* = 4.9, 1.3 Hz, 1H), 6.33 (s, 1H), 4.31 (d, *J* = 12.1 Hz, 1H), 1.75 (td, *J* = 12.4, 3.1 Hz, 1H), 1.25 (s, 6H), 1.24 (s, 6H), 1.04 (m, 2H), 0.95 (s, 6H), 0.91 (s, 6H). *Minor diastereoisomer*: ^{1}H NMR ($CDCl_3$, 400 MHz) δ 7.20 – 7.10 (m, 5H), 7.10 – 7.05 (m, 1H), 6.78 (dd, *J* = 3.0, 1.3 Hz, 1H), 6.57 (dd, *J* = 4.9, 1.3 Hz, 1H), 6.14 (s, 1H), 4.39 (d, *J* = 12.1 Hz, 1H), 1.75 (td, *J* = 12.4, 3.1 Hz, 1H), 1.33 (s, 6H), 1.28 (s, 6H), 1.19 (s, 12H), 1.04 (m, 2H).

Major diastereoisomer ^{13}C NMR ($CDCl_3$, 100 MHz) δ 149.5, 141.8, 139.1, 128.9, 128.6, 128.5, 127.9, 127.6, 126.5, 123.9, 123.8, 123.1, 83.1, 82.9, 82.1, 82.0, 55.9, 25.5, 25.0, 24.9, 24.8, 24.8, 24.6, 24.5, 24.4.

^{11}B NMR ($CDCl_3$, 128.3 MHz) δ 33.7.

HRMS-(ESI+) for $C_{27}H_{37}B_2IO_4S$ $[M+H]^+$: calculated: 607.1730; found: 607.1686.

(*E*)-2,2'-(5-iodo-4-phenyl-3-(4-(trifluoromethyl)phenyl)pent-4-ene-1,2-diyl)bis(4,4,5,5-tetramethyl-1,3,2-dioxaborolane) ((±)-6)

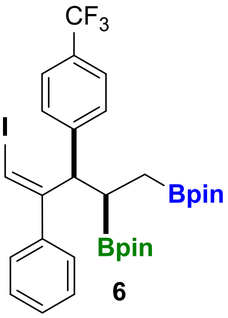

The product (±)-**6** was purified by flash chromatography using a mixture of petroleum ether and diethyl ether (100:10) as eluent. It was isolated as a colorless oil (104 mg, 86%) as a mixture of both diastereoisomers (20:1). Product (*R*)-**6** (84 mg, 63%).

Major diastereoisomer: ^{1}H NMR ($CDCl_3$, 400 MHz) δ 7.31 (m, 2H), 7.20 – 7.01 (m, 5H), 6.65 – 6.58 (m, 2H), 6.21 (s, 1H), 4.31 (d, *J* = 12.2 Hz, 1H), 1.68 (m, 1H), 1.19 (s, 6H), 1.19 (m, 1H), 1.18 (s, 6H), 1.04 (m, 1H), 0.83 (s, 6H), 0.80 (s, 6H). *Minor diastereoisomer*: ^{1}H NMR ($CDCl_3$, 400 MHz) δ 7.31 (m, 2H), 7.20 – 7.01 (m, 5H), 6.65 – 6.58 (m, 2H), 6.10 (s, 1H), 4.56 (d, *J* = 12.3 Hz, 1H) 1.68 (m, 1H), 1.19 (s, 6H), 1.19 (m, 1H), 1.18 (s, 6H), 1.04 (m, 1H), 0.83 (s, 6H), 0.80 (s, 6H).

Major diastereoisomer ^{13}C NMR ($CDCl_3$, 100 MHz) δ 153.0, 145.8, 145.8, 139.0, 129.4, 129.3, 127.5, 124.4, 124.4, 83.2, 83.0, 82.9, 56.4, 25.0, 24.8, 24.4, 24.3. *Quaternary C heterocoupled with F could not be seen.

^{11}B NMR ($CDCl_3$, 128.3 MHz) δ 33.2.

^{19}F NMR ($CDCl_3$, 376.5 MHz): δ -62.3.

HRMS-(ESI+) for $C_{30}H_{38}B_2F_3IO_4$ $[M+H]^+$: calculated: 669.2042; found: 669.1993.

(*E*)-2,2'-(5-iodo-3-methyl-4-phenylpent-4-ene-1,2-diyl)bis(4,4,5,5-tetramethyl-1,3,2-dioxaborolane) ((±)-7)

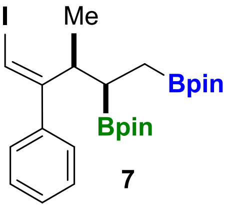

The product (±)-**7** was purified by flash chromatography using a mixture of petroleum ether and diethyl ether (100:10) as eluent. It was isolated as a colorless oil (79 mg, 73%) as a mixture of both diastereoisomers (16:1). Product (*R*)-**7** (54 mg, 50%).

Major diastereoisomer: ^{1}H NMR ($CDCl_3$, 400 MHz) δ 7.32 – 7.19 (m, 3H), 7.16 – 7.08 (m, 2H), 6.21 (s, 1H), 3.07 (m, 1H), 1.26 (s, 12H), 1.22 (s, 6H), 1.21 (s, 6H), 1.15 (m, 1H), 1.04 (d, *J* = 6.9 Hz, 3H), 1.02 – 0.95 (m, 2H). *Minor diastereoisomer*: ^{1}H NMR ($CDCl_3$, 400 MHz) δ 7.32 – 7.19 (m, 3H), 7.16 – 7.08 (m, 2H), 6.05 (s, 1H), 3.07 (m, 1H), 1.26 (s, 12H), 1.22 (s, 6H), 1.21 (s, 6H), 1.15 (m, 1H), 1.04 (d, *J* = 6.9 Hz, 3H), 1.02 – 0.95 (m, 2H).

^{13}C NMR (CDCl$_3$, 100 MHz) δ 155.9, 140.4, 128.5, 127.7, 127.2, 83.0, 82.9, 81.1, 45.1, 25.0, 25.0, 24.8, 24.7, 19.1.

^{11}B NMR (CDCl$_3$, 128.3 MHz) δ 34.9.

HRMS-(ESI+) for $C_{24}H_{37}B_2IO_4$ [M+H]$^+$: calculated: 539.2005; found: 539.1968.

(*E*)-2,2'-(5-iodo-3-methyl-4-(o-tolyl)pent-4-ene-1,2-diyl)bis(4,4,5,5-tetramethyl-1,3,2-dioxaborolane) ((±)-8)

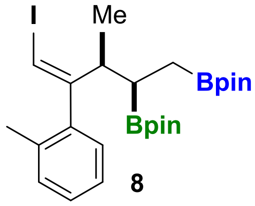

8

The product (±)-**8** was purified by flash chromatography using a mixture of petroleum ether and diethyl ether (100:10) as eluent. It was isolated as a colorless oil (64 mg, 58%) as a mixture of both diastereoisomers (10:1).

Major diastereoisomer: ^{1}H NMR (CDCl$_3$, 400 MHz) δ 7.18 – 7.08 (m, 2H), 7.05 (m, 1H), 6.95 (dd, *J* = 7.4, 1.3 Hz, 1H), 6.09 (s, 1H), 3.11 – 2.99 (m, 1H), 2.23 (s, 3H), 1.24 (s, 6H), 1.24 (s, 6H), 1.24 (m, 2H), 1.24 (s, 6H), 1.23 (s, 6H), 1.05 (m, 1H), 0.92 (d, *J* = 6.9 Hz, 3H). *Minor diastereoisomer*: ^{1}H NMR (CDCl$_3$, 400 MHz) δ 7.18 – 7.08 (m, 2H), 7.05 (m, 1H), 6.95 (dd, *J* = 7.4, 1.3 Hz, 1H), 5.93 (s, 1H), 3.11 – 2.99 (m, 1H), 2.23 (s, 3H), 1.24 (s, 6H), 1.24 (s, 6H), 1.24 (m, 2H), 1.24 (s, 6H), 1.23 (s, 6H), 1.05 (m, 1H), 0.92 (d, *J* = 6.9 Hz, 3H).

^{13}C NMR (CDCl$_3$, 100 MHz) δ 153.3, 139.2, 136.4, 130.3, 128.9, 127.1, 124.9, 83.0, 83.0, 81.5, 45.8, 25.0, 25.0, 24.9, 24.7, 20.4, 18.2.

^{11}B NMR (CDCl$_3$, 128.3 MHz) δ 34.5.

HRMS-(ESI+) for $C_{25}H_{39}B_2IO_4$ [M+H]$^+$: calculated: 553.2159; found: 553.2124.

(*E*)-2,2'-(3-(2-iodo-1-phenylvinyl)hexane-1,2-diyl)bis(4,4,5,5-tetramethyl-1,3,2-dioxaborolane) ((±)-9)

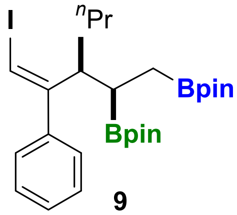

9

The product (±)-**9** was purified by flash chromatography using a mixture of petroleum ether and diethyl ether (100:10) as eluent. It was isolated as a colorless oil (85 mg, 75%) as a mixture of both diastereoisomers (7:1). Product (*R*)-**9** (67 mg, 60%).

Major diastereoisomer: ^{1}H NMR (CDCl$_3$, 400 MHz) δ 7.27 – 7.20 (m, 3H), 7.10 (m, 2H), 6.34 (s, 1H), 3.08 – 2.96 (m, 1H), 1.39 – 1.28 (m, 4H), 1.25 (s, 12H), 1.25 (m, 1H), 1.22 (s, 6H), 1.21 (s, 6H), 1.10 – 0.96 (m, 2H), 0.88 (t, *J* = 7.0 Hz, 3H). *Minor diastereoisomer*: ^{1}H NMR (CDCl$_3$, 400 MHz) δ 7.27 – 7.20 (m, 3H), 7.10 (m, 2H), 6.19 (s, 1H), 3.08 – 2.96 (m, 1H), 1.39 – 1.28 (m, 4H), 1.25 (s, 12H), 1.25 (m, 1H), 1.22 (s, 6H), 1.21 (s, 6H), 1.10 – 0.96 (m, 2H), 0.88 (t, *J* = 7.0 Hz, 3H).

^{13}C NMR ($CDCl_3$, 100 MHz) δ 153.8, 128.9, 128.4, 127.8, 127.2, 83.0, 82.9, 50.5, 35.4, 25.0, 25.0, 24.8, 24.7, 24.7, 20.8, 14.4.

^{11}B NMR ($CDCl_3$, 128.3 MHz) δ 34.7.

HRMS-(ESI+) for $C_{26}H_{42}B_2IO_4$ $[M+H]^+$: calculated: 567.2280; found: 567.2336.

(*E*)-2,2'-(5-iodo-3-isopropyl-4-phenylpent-4-ene-1,2-diyl)bis(4,4,5,5-tetramethyl-1,3,2-dioxaborolane) ((±)-10)

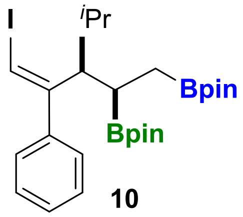

10

The product (±)-**10** was purified by flash chromatography using a mixture of petroleum ether and diethyl ether (100:10) as eluent. It was isolated as a colorless oil (32 mg, 28%) as a mixture of both diastereoisomers (12:1).

Major diastereoisomer: ^{1}H NMR ($CDCl_3$, 400 MHz) δ 7.29 – 7.17 (m, 5H), 6.39 (s, 1H), 3.05 – 3.00 (dd, *J* = 5.6, 5 Hz, 1H), 1.87 – 1.79 (m, 1H), 1.70 – 1.59 (m, 1H), 1.27 (s, 6H), 1.27 (m, 1H), 1.25 (s, 6H), 1.23 (s, 6H), 1.22 (s, 6H), 0.98 (d, *J* = 6.9 Hz, 3H), 0.82 (m, 2H), 0.69 (d, *J* = 6.8 Hz, 3H). *Minor diastereoisomer*: ^{1}H NMR ($CDCl_3$, 400 MHz) δ 7.29 – 7.17 (m, 5H), 6.16 (s, 1H), 3.05 – 3.00 (m, 1H), 1.87 – 1.79 (m, 1H), 1.70 – 1.59 (m, 1H), 1.27 (s, 6H), 1.27 (m, 1H), 1.25 (s, 6H), 1.23 (s, 6H), 1.22 (s, 6H), 0.98 (d, *J* = 6.9 Hz, 3H), 0.82 (m, 2H), 0.69 (d, *J* = 6.8 Hz, 3H).

^{13}C NMR ($CDCl_3$, 100 MHz) δ 153.3, 129.1, 128.4, 127.9, 127.2, 83.0, 82.9, 56.1, 31.0, 25.0, 25.0, 24.9, 24.7, 21.4, 19.7.

^{11}B NMR ($CDCl_3$, 128.3 MHz) δ 33.9.

HRMS-(ESI+) for $C_{26}H_{42}B_2IO_4$ $[M+H]^+$: calculated: 567.2280; found: 567.2277.

(*E*)-2,2'-(3-(2-iodo-1-phenylvinyl)-5-methylhexane-1,2-diyl)bis(4,4,5,5-tetramethyl-1,3,2-dioxaborolane) ((±)-11)

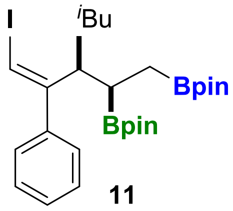

11

The product (±)-**11** was purified by flash chromatography using a mixture of petroleum ether and diethyl ether (100:10) as eluent. It was isolated as a colorless oil (13 mg, 11%) as a mixture of both diastereoisomers (9:1).

Major diastereoisomer: ^{1}H NMR ($CDCl_3$, 400 MHz) δ 7.29 – 7.20 (m, 3H), 7.12 – 7.03 (m, 2H), 6.33 (s, 1H), 3.08 (dd, *J* = 5.1, 4.9 Hz, 1H), 1.54 (m, 1H), 1.25 (s, 12H), 1.25 (m, 3H), 1.23 (s, 6H), 1.23 (s, 6H), 1.17 – 1.03 (m, 2H), 1.00 (d, *J* = 6.5 Hz, 3H), 0.77 (d, *J* = 6.6 Hz, 3H). *Minor diastereoisomer*: ^{1}H NMR ($CDCl_3$, 400 MHz) δ 7.29 – 7.20 (m, 3H), 7.12 – 7.03 (m, 2H), 6.17 (s, 1H), 3.08 (m, 1H), 1.54 (m, 1H), 1.25 (s, 12H), 1.25 (m, 2H), 1.23 (s, 6H), 1.23 (s, 6H), 1.17 – 1.03 (m, 2H), 1.00 (d, *J* = 6.5 Hz, 3H), 0.77 (d, *J* = 6.6 Hz, 3H).

^{13}C NMR ($CDCl_3$, 100 MHz) δ 154.0, 129.1, 128.5, 127.9, 127.2, 83.0, 83.0, 48.5, 42.5, 26.1, 25.2, 25.0, 24.8, 24.7, 24.1, 22.6.

^{11}B NMR ($CDCl_3$, 128.3 MHz) δ 34.9.

HRMS-(ESI+) for $C_{27}H_{44}B_2IO_4$ $[M+H]^+$: calculated: 581.2436; found: 581.2494.

(*Z*)-2,2'-(3-phenylpent-1-ene-1,5-diyl)bis(4,4,5,5-tetramethyl-1,3,2-dioxaborolane) (I)

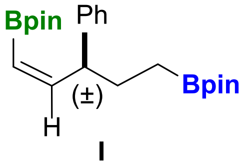

I

The product was purified by flash chromatography using a mixture of petroleum ether and diethyl ether (100:10) as eluent. It was isolated as a colorless oil (23 mg, 29%).

^{1}H NMR ($CDCl_3$, 400 MHz) δ 7.25 – 7.20 (m, 2H), 7.18 – 7.11 (m, 3H), 6.58 (dd, *J* = 18.0, 7.0 Hz, 1H), 5.35 (dd, *J* = 18.0, 1.0 Hz, 1H), 2.79 – 2.52 (m, 3H), 1.24 (s, 12H), 1.20 (s, 6H), 1.20 (s, 6H), 0.93 – 0.80 (m, 2H).

^{13}C NMR ($CDCl_3$, 100 MHz) δ 158.8, 140.5, 129.4, 128.0, 125.8, 83.0, 82.9, 43.1, 43.0, 29.7, 24.9, 24.8, 24.8, 24.7.

^{11}B NMR ($CDCl_3$, 128.3 MHz) δ 34.20, 30.25.

HRMS-(ESI+) for $C_{23}H_{36}B_2O_4$ $[M+H]^+$: calculated: 399.2457; found: 399.2460.

(*E*)-4,4,5,5-tetramethyl-2-(2-phenyl-2-(2-((4,4,5,5-tetramethyl-1,3,2-dioxaborolan-2-yl)methyl)cyclopropyl)vinyl)-1,3,2-dioxaborolane (II)

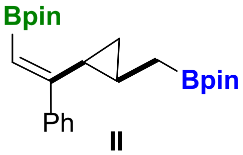

II

The product was purified by flash chromatography using a mixture of petroleum ether and diethyl ether (100:10) as eluent. It was isolated as a colorless oil (55 mg, 65%).

^{1}H NMR ($CDCl_3$, 400 MHz) δ 7.25 – 7.15 (m, 5H), 5.35 (s, 1H), 2.18 – 2.09 (m, 1H), 1.31 (s, 12H), 1.23 (s, 12H), 0.92 – 0.78 (m, 2H), 0.63 (m, 3H).

^{13}C NMR ($CDCl_3$, 100 MHz) δ 164.7, 143.1, 127.5, 126.8, 83.0, 82.9, 25.1, 25.0, 24.9, 24.9, 24.8, 15.6, 15.2.

^{11}B NMR ($CDCl_3$, 128.3 MHz) δ 33.90, 30.22.

HRMS-(ESI+) for $C_{24}H_{37}B_2O_4$ $[M+H]^+$: calculated: 411.2873; found: 411.2872.

Characterization data for cyclic products

2-(2,3-diphenylcyclopent-3-en-1-yl)-4,4,5,5-tetramethyl-1,3,2-dioxaborolane (12)

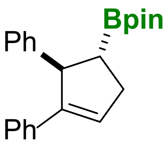

12

The product **12** was purified by flash chromatography using as eluent a mixture of petroleum ether/diethyl ether (100:5). The product was isolated as a colorless oil (37 mg, 54%). Product (*R*,*R*)-**12** (40 mg, 59%).

^{1}H NMR ($CDCl_3$, 400 MHz) δ 7.39 – 7.28 (m, 3H), 7.25 – 7.09 (m, 7H), 6.42 (m, 1H), 4.53 (m, 1H), 2.85 (m, 1H), 2.64 (m, 1H), 1.83 – 1.70 (m, 1H), 1.33 (s, 6H), 1.32 (s, 6H).

^{13}C NMR ($CDCl_3$, 100 MHz) δ 146.3, 144.7, 136.3, 129.2, 128.4, 128.0, 127.6, 126.5, 126.4, 125.7, 83.3, 54.3, 34.3, 24.9, 24.7.

^{11}B NMR ($CDCl_3$, 128.3 MHz) δ 35.1.

HRMS-(ESI+) for $C_{23}H_{28}BO_2$ $[M+H]^+$: calculated: 347.2175; found: 347.2177.

4,4,5,5-tetramethyl-2-(2-phenyl-3-(o-tolyl)cyclopent-3-en-1-yl)-1,3,2-dioxaborolane (13)

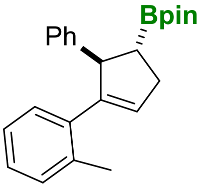

13

The product was purified by flash chromatography using a mixture of petroleum ether and diethyl ether (100:5) as eluent. It was isolated as a colorless oil (42 mg, 58%). Product (*R*,*R*)-**13** (45 mg, 62%).

^{1}H NMR ($CDCl_3$, 400 MHz) δ 7.17 – 7.09 (m, 4H), 7.07 – 6.92 (m, 5H), 5.92 (m, 1H), 4.43 (m, 1H), 2.83 (m, 1H), 2.59 (m, 1H), 2.36 (s, 3H), 1.74 (m, 1H), 1.27 (s, 6H), 1.26 (s, 6H).

^{13}C NMR ($CDCl_3$, 100 MHz) δ 146.1, 145.6, 137.3, 135.5, 131.4, 130.2, 128.5, 128.1, 127.8, 126.3, 125.7, 125.2, 83.3, 57.0, 34.9, 24.9, 24.7, 21.0.

^{11}B NMR ($CDCl_3$, 128.3 MHz) δ 35.9.

HRMS-(ESI+) for $C_{24}H_{30}BO_2$ $[M+H]^+$: calculated: 361.2333; found: 361.2332.

2-(3-(4-bromophenyl)-2-phenylcyclopent-3-en-1-yl)-4,4,5,5-tetramethyl-1,3,2-dioxaborolane (14)

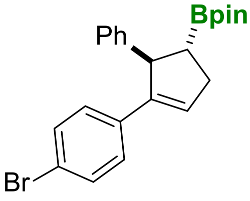

14

The product was purified by flash chromatography using a mixture of petroleum ether and diethyl ether (100:5) as eluent. It was isolated as a colorless oil (51 mg, 60%).

^{1}H NMR ($CDCl_3$, 400 MHz) δ 7.30 – 7.23 (m, 2H), 7.23 – 7.08 (m, 7H), 6.36 (m, 1H), 4.46 – 4.37 (m, 1H), 2.77 (m, 1H), 2.56 (m, 1H), 1.70 (m, 1H), 1.27 (s, 6H), 1.26 (s, 6H).

^{13}C NMR ($CDCl_3$, 100 MHz) δ 145.9, 143.8, 135.2, 131.1, 130.0, 128.5, 128.0, 127.5, 125.9, 120.3, 83.4, 54.3, 34.4, 25.0, 24.7.

^{11}B NMR ($CDCl_3$, 128.3 MHz) δ 35.6.

HRMS-(ESI+) for $C_{23}H_{26}BBrO_2$ $[M+H]^+$: calculated: 425.1282; found: 425.1260.

4,4,5,5-tetramethyl-2-(2-phenyl-3-(thiophen-3-yl)cyclopent-3-en-1-yl)-1,3,2-dioxaborolane (15)

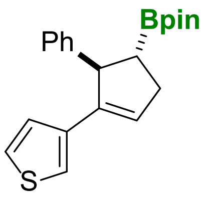

15

The product was purified by flash chromatography using a mixture of petroleum ether and diethyl ether (100:5) as eluent. It was isolated as a colorless oil (34 mg, 48%).

^1H NMR ($CDCl_3$, 400 MHz) δ 7.25 – 7.20 (m, 4H), 7.18 – 7.06 (m, 3H), 6.67 (dd, J = 3.0, 1.2 Hz, 1H), 6.24 (m, 1H), 4.31 (m, 1H), 2.80 (m, 1H), 2.55 (m, 1H), 1.68 (m, 1H), 1.25 (s, 6H), 1.25 (s, 6H).

^{13}C NMR ($CDCl_3$, 100 MHz) δ 146.6, 139.9, 137.9, 128.4, 128.2, 127.6, 126.5, 125.9, 124.6, 121.0, 83.3, 55.2, 34.3, 24.9, 24.7.

^{11}B NMR ($CDCl_3$, 128.3 MHz) δ 33.9.

HRMS-(ESI+) for $C_{21}H_{25}BO_2S$ $[M+H]^+$: calculated: 353.1743; found: 353.1723.

4,4,5,5-tetramethyl-2-(3-phenyl-2-(4-(trifluoromethyl)phenyl)cyclopent-3-en-1-yl)-1,3,2-dioxaborolane (16)

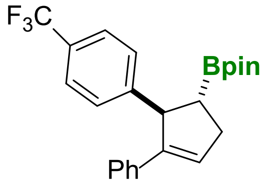

16

The product was purified by flash chromatography using a mixture of petroleum ether and diethyl ether (100:5) as eluent. It was isolated as a colorless oil (48 mg, 58%). Product (*R*,*R*)-**16** (36 mg, 44%).

^1H NMR ($CDCl_3$, 400 MHz) δ 7.37 (d, J = 8.0 Hz, 2H), 7.22 (d, J = 8.1 Hz, 2H), 7.19 – 7.13 (m, 2H), 7.13 – 6.98 (m, 3H), 6.31 (m, 1H), 4.45 (m, 1H), 2.71 (m, 1H), 2.53 (m, 1H), 1.58 (m, 1H), 1.19 (s, 6H), 1.19 (s, 6H).

^{13}C NMR ($CDCl_3$, 100 MHz) δ 150.5, 144.1, 135.8, 129.9, 128.2, 127.9, 126.8, 126.4, 125.4, 125.4, 125.4, 125.3, 83.5, 54.0, 34.4, 24.9, 24.7. *Quaternary C heterocoupled with F could not be seen.

^{11}B NMR ($CDCl_3$, 128.3 MHz) δ 34.8.

^{19}F NMR ($CDCl_3$, 376.5 MHz): δ -62.2.

HRMS-(ESI+) for $C_{24}H_{26}BF_3O_2$ $[M+H]^+$: calculated: 415.2051; found: 415.2030.

4,4,5,5-tetramethyl-2-(2-methyl-3-phenylcyclopent-3-en-1-yl)-1,3,2-dioxaborolane (17)

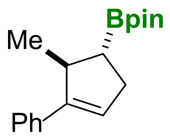

17

The product was purified by flash chromatography using a mixture of petroleum ether and diethyl ether (100:5) as eluent. It was isolated as a colorless oil (28 mg, 50%). Product (*R*,*R*)-**17** (30 mg, 55%).

^{1}H NMR ($CDCl_3$, 400 MHz) δ 7.40 – 7.34 (m, 2H), 7.34 – 7.26 (m, 3H), 7.24 – 7.16 (m, 1H), 5.93 (m, 1H), 3.30 (m, 1H), 2.63 (m, 1H), 1.47 – 1.34 (m, 1H), 1.27 (s, 12H), 1.13 (d, *J* = 6.8 Hz, 3H).

^{13}C NMR ($CDCl_3$, 100 MHz) δ 148.1, 137.1, 128.2, 127.0, 126.5, 126.4, 83.1, 42.6, 33.8, 24.8, 20.9.

^{11}B NMR ($CDCl_3$, 128.3 MHz) δ 36.1.

HRMS-(ESI+) for $C_{18}H_{26}BO_2$ [M+H]$^+$: calculated: 285.2023; found: 285.2006.

4,4,5,5-tetramethyl-2-(2-methyl-3-(o-tolyl)cyclopent-3-en-1-yl)-1,3,2-dioxaborolane (18)

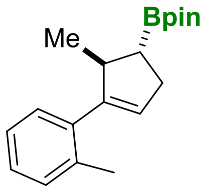

18

The product was purified by flash chromatography using a mixture of petroleum ether and diethyl ether (100:5) as eluent. It was isolated as a colorless oil (33 mg, 56%).

^{1}H NMR ($CDCl_3$, 400 MHz) δ 7.20 – 7.04 (m, 4H), 5.61 (m, 1H), 3.15 (m, 1H), 2.61 (m, 1H), 2.47 (m, 1H), 2.30 (s, 3H), 1.28 (s, 12H), 1.27 – 1.20 (m, 1H), 0.95 (d, *J* = 6.8 Hz, 3H).

^{13}C NMR ($CDCl_3$, 100 MHz) δ 148.6, 138.1, 135.7, 130.0, 128.7, 128.6, 126.4, 125.2, 83.0, 45.5, 34.2, 24.8, 20.5, 20.3.

^{11}B NMR ($CDCl_3$, 128.3 MHz) δ 34.9.

HRMS-(ESI+) for $C_{19}H_{28}BO_2$ [M+H]$^+$: calculated: 299.3120; found: 299.3112.

4,4,5,5-tetramethyl-2-(3-phenyl-2-propylcyclopent-3-en-1-yl)-1,3,2-dioxaborolane (19)

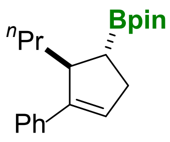

19

The product was purified by flash chromatography using a mixture of petroleum ether and diethyl ether (100:5) as eluent. It was isolated as a colorless oil (34 mg, 54%). Product (*R*,*R*)-**19** (38 mg, 60%).

^{1}H NMR ($CDCl_3$, 400 MHz) δ 7.39 – 7.34 (m, 2H), 7.30 (m, 2H), 7.23 – 7.17 (m, 1H), 5.92 (m, 1H), 3.36 – 3.27 (m, 1H), 2.65 (m, 1H), 2.48 (m, 1H), 1.66 (m, 1H), 1.49 (m, 1H), 1.36 – 1.29 (m, 2H), 1.27 (s, 12H), 1.23 – 1.12 (m, 1H), 0.86 (t, *J* = 7.3 Hz, 3H).

^{13}C NMR ($CDCl_3$, 100 MHz) δ 147.1, 137.3, 128.2, 126.9, 126.5, 126.4, 83.1, 47.8, 37.3, 34.3, 24.7, 24.7, 20.5, 14.4.

^{11}B NMR ($CDCl_3$, 128.3 MHz) δ 34.8.

HRMS-(ESI+) for $C_{20}H_{30}BO_2$ [M+H]$^+$: calculated: 313.2311; found: 313.2355.

2-(2-isopropyl-3-phenylcyclopent-3-en-1-yl)-4,4,5,5-tetramethyl-1,3,2-dioxaborolane (20)

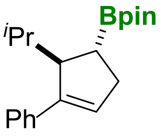

20

The product was purified by flash chromatography using a mixture of petroleum ether and diethyl ether (100:5) as eluent. It was isolated as a colorless oil (31 mg, 49%).

^{1}H NMR ($CDCl_3$, 400 MHz) δ 7.42 – 7.32 (m, 2H), 7.31 (m, 2H), 7.24 – 7.15 (m, 1H), 5.91 (m, 1H), 3.41 (m, 1H), 2.60 (m, 1H), 2.47 (m, 1H), 1.66 – 1.56 (m, 2H), 1.26 (s, 12H), 0.92 (d, *J* = 6.9 Hz, 3H), 0.59 (d, *J* = 6.8 Hz, 3H).

^{13}C NMR ($CDCl_3$, 100 MHz) δ 146.3, 137.6, 128.1, 127.7, 126.6, 126.5, 83.0, 53.8, 34.7, 28.5, 24.7, 24.7, 21.5, 15.5.

^{11}B NMR ($CDCl_3$, 128.3 MHz) δ 35.2.

HRMS-(ESI+) for $C_{20}H_{30}BO_2$ [M+H]$^+$: calculated: 313.2311; found: 313.2323.

(1*R,2*R**)-2,3-diphenylcyclopent-3-en-1-ol (21)**

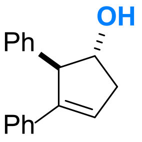

21

The product **21** was purified by flash chromatography using as eluent a mixture of petroleum ether/ethyl acetate (50:50). The product was isolated as a colorless oil (43 mg, 90%).

^{1}H NMR ($CDCl_3$, 400 MHz) δ 7.36 – 7.26 (m, 3H), 7.25 – 7.12 (m, 7H), 6.43 (m, 1H), 4.31 (m, 1H), 4.20 (s, 1H), 2.97 (m, 1H), 2.49 (m, 1H), 1.26 (s, 1H).

^{13}C NMR ($CDCl_3$, 100 MHz) δ 142.7, 140.8, 135.4, 128.8, 128.3, 127.5, 127.2, 126.7, 126.3, 125.2, 80.9, 62.1, 41.2.

HRMS-(ESI+) for $C_{17}H_{17}O$ [M+H]$^+$: calculated: 237.1262; found: 237.1286.

(1*R*,2*R*)-2,3-diphenylcyclopent-3-en-1-ol (*R,R*)-**21**

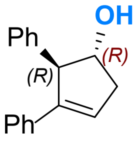

(*R,R*)-**21**

The product (*R,R*)-**21** was purified by flash chromatography using as eluent a mixture of petroleum ether/ethyl acetate (50:50). The product was isolated as a colorless oil (37mg, 78%).

e.r= 95:5 Optical rotation: $[\alpha]_D^{25}$ -219.0 (c=1.7, $CHCl_3$).

(1*R,2*R**)-2-phenyl-3-(o-tolyl)cyclopent-3-en-1-ol (22)**

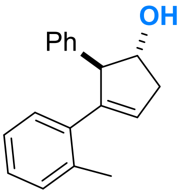

22

The product **22** was purified by flash chromatography using a mixture of petroleum ether and ethyl acetate (50:50) as eluent. It was isolated as a colorless oil (35 mg, 69%).

^{1}H NMR ($CDCl_3$, 400 MHz) δ 7.24 – 7.19 (m, 2H), 7.17 – 7.09 (m, 3H), 7.08 – 6.99 (m, 4H), 5.94 (m, 1H), 4.38 (m, 1H), 4.17 (m, 1H), 3.01 (m, 1H), 2.55 (m, 1H), 2.37 (s, 3H), 1.26 (s, 1H).

^{13}C NMR ($CDCl_3$, 100 MHz) δ 144.1, 141.2, 136.5, 135.7, 130.4, 128.7, 128.6, 127.8, 126.8, 126.6, 125.4, 81.1, 64.1, 41.2, 30.3, 21.2.

HRMS-(ESI+) for $C_{18}H_{18}O$ $[M+H]^+$: calculated: 251.1430; found: 251.1441.

(1*R*,2*R*)-2-phenyl-3-(o-tolyl)cyclopent-3-en-1-ol (*R,R*)-**(22)**

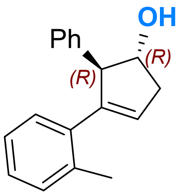

(*R,R*)-**22**

The product (*R,R*)-**21** was purified by flash chromatography using as eluent a mixture of petroleum ether/ethyl acetate (50:50). The product was isolated as a colorless oil (37mg, 73%).

e.r= 88:12 Optical rotation: $[\alpha]_D^{25}$ -46.3 (c=1.0, $CHCl_3$).

(1*R,2*R**)-3-(4-bromophenyl)-2-phenylcyclopent-3-en-1-ol (23)**

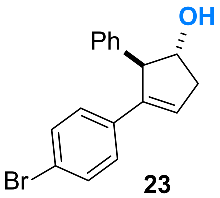

23

The product **23** was purified by flash chromatography using a mixture of petroleum ether and ethyl acetate (50:50) as eluent. It was isolated as a colorless oil (48 mg, 79%).

^{1}H NMR ($CDCl_3$, 400 MHz) δ 7.35 – 7.30 (m, 2H), 7.30 – 7.23 (m, 2H), 7.22 – 7.13 (m, 5H), 6.45 – 6.39 (m, 1H), 4.31 (m, 1H), 4.15 (m, 1H), 3.00 – 2.89 (m, 1H), 2.54 – 2.44 (m, 1H), 2.00 (s, 1H).

^{13}C NMR ($CDCl_3$, 100 MHz) δ 141.8, 140.5, 134.4, 131.4, 128.9, 127.9, 127.5, 126.9, 126.0, 121.0, 80.9, 62.0, 41.3.

HRMS-(ESI+) for $C_{17}H_{16}BrO$ $[M+H]^+$: calculated: 315.0363; found: 315.0395

(1*R,2*R**)-2-phenyl-3-(thiophen-3-yl)cyclopent-3-en-1-ol (24)**

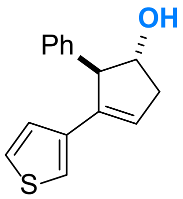

24

The product was purified by flash chromatography using a mixture of petroleum ether and ethyl acetate (50:50) as eluent. It was isolated as a colorless oil (37 mg, 77%).

^{1}H NMR ($CDCl_3$, 400 MHz) δ 7.25 (s, 3H), 7.25 – 7.15 (m, 4H), 6.82 (dd, *J* = 3.0, 1.3 Hz, 1H), 6.25 (m, 1H), 4.30 (m, 1H), 4.08 (s, 1H), 2.97 (m, 1H), 2.47 (m, 1H), 1.23 (s, 1H).

^{13}C NMR ($CDCl_3$, 100 MHz) δ 140.9, 138.4, 137.5, 128.8, 127.6, 126.8, 126.0, 125.3, 124.4, 121.5, 80.9, 63.0, 41.2.

HRMS-(ESI+) for $C_{15}H_{15}OS$ $[M+H]^+$: calculated: 243.0826; found: 243.0850.

(1*R*,2*R*)-2-phenyl-3-(thiophen-3-yl)cyclopent-3-en-1-ol (*R,R*)-**(24)**

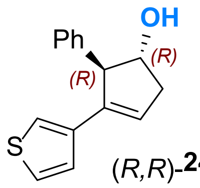

(*R,R*)-**24**

The product (*R,R*)-**24** was purified by flash chromatography using a mixture of petroleum ether and ethyl acetate (50:50) as eluent. It was isolated as a colorless oil (57%).

e.r= 89:11 Optical rotation: $[\alpha]_D^{25}$ -130.3 (c=0.1, $CHCl_3$)

(1*R,2*R**)-3-phenyl-2-(4-(trifluoromethyl)phenyl)cyclopent-3-en-1-ol (25)**

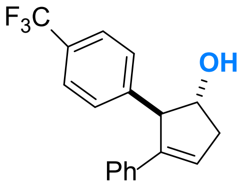

25

The product **25** was purified by flash chromatography using a mixture of petroleum ether and ethyl acetate (50:50) as eluent. It was isolated as a colorless oil (56 mg, 92%).

^1H NMR ($CDCl_3$, 400 MHz) δ 7.47 (d, *J* = 8.1 Hz, 2H), 7.33 – 7.22 (m, 4H), 7.22 – 7.04 (m, 3H), 6.40 (m, 1H), 4.27 – 4.20 (m, 2H), 2.91 (m, 1H), 2.54 – 2.39 (m, 1H), 2.06 (s, 1H).

^{13}C NMR ($CDCl_3$, 100 MHz) δ 145.1, 142.2, 135.0, 128.4, 127.9, 127.5, 126.2, 125.8, 125.8, 125.8, 125.7, 80.7, 61.7, 41.3. *Quaternary C heterocoupled with F could not be seen.

^{19}F NMR ($CDCl_3$, 376.5 MHz): δ -62.39.

HRMS-(ESI+) for $C_{18}H_{16}F_3O$ $[M+H]^+$: calculated: 305.1127; found: 305.1169.

(1*R*,2*R*)-3-phenyl-2-(4-(trifluoromethyl)phenyl)cyclopent-3-en-1-ol (*R,R*)-**(25)**

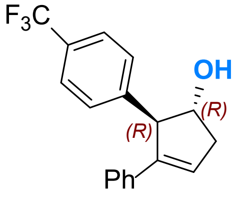

(*R,R*)-**25**

The product (*R,R*)-**25** was purified by flash chromatography using a mixture of petroleum ether and ethyl acetate (50:50) as eluent. It was isolated as a colorless oil (55 mg, 91%).

e.r= 95:5 Optical rotation: $[\alpha]_D^{25}$ -69.4 (c=1.0, $CHCl_3$)

(1*R,2*R**)-2-methyl-3-phenylcyclopent-3-en-1-ol (26)**

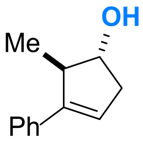

26

The product was purified by flash chromatography using a mixture of petroleum ether and ethyl acetate (50:50) as eluent. It was isolated as a colorless oil (32 mg, 92%).

^1H NMR ($CDCl_3$, 400 MHz) δ 7.39 – 7.32 (m, 2H), 7.29 – 7.21 (m, 2H), 7.19 – 7.12 (m, 1H), 5.96 (m, 1H), 4.11 – 4.05 (m, 1H), 3.06 – 2.94 (m, 1H), 2.82 (m, 1H), 2.43 – 2.27 (m, 1H), 1.03 (d, *J* = 7.1 Hz, 3H).

^{13}C NMR ($CDCl_3$, 100 MHz) δ 145.9, 135.6, 128.4, 127.2, 126.1, 122.0, 79.2, 49.6, 41.1, 16.9.

HRMS-(ESI+) for $C_{12}H_{15}O$ $[M+H]^+$: calculated: 175.1108; found: 175.1126.

(1*R*,2*R*)-2-methyl-3-phenylcyclopent-3-en-1-ol (*R,R*)-**(26)**

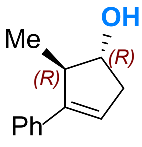

(*R,R*)-**26**

The product (*R,R*)-**26** was purified by flash chromatography using a mixture of petroleum ether and ethyl acetate (50:50) as eluent. It was isolated as a colorless oil (21 mg, 61%).

e.r= 94:6 Optical rotation: $[\alpha]_D^{25}$ -73.4 (c=0.8, $CHCl_3$).

(1*R,2*R**)-2-methyl-3-(o-tolyl)cyclopent-3-en-1-ol (27)**

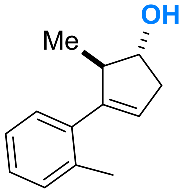

27

The product was purified by flash chromatography using a mixture of petroleum ether and ethyl acetate (50:50) as eluent. It was isolated as a colorless oil (31 mg, 83%).

^1H NMR ($CDCl_3$, 400 MHz) δ 7.21 – 7.04 (m, 4H), 5.56 (m, 1H), 4.16 (m, 1H), 3.03 – 2.78 (m, 2H), 2.48 – 2.36 (m, 1H), 2.32 (s, 3H), 1.74 (s, 1H), 0.97 (d, *J* = 7.2 Hz, 3H).

^{13}C NMR ($CDCl_3$, 100 MHz) δ 146.9, 137.2, 135.9, 130.2, 128.7, 126.9, 125.4, 124.8, 79.7, 52.0, 41.1, 20.5, 16.6.

HRMS-(ESI+) for $C_{13}H_{17}O$ $[M+H]^+$: calculated: 189.1265; found: 189.1283.

(1*R,2*R**)-3-phenyl-2-propylcyclopent-3-en-1-ol (28)**

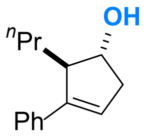

28

The product was purified by flash chromatography using a mixture of petroleum ether and ethyl acetate (50:50) as eluent. It was isolated as a colorless oil (33 mg, 82%).

^1H NMR ($CDCl_3$, 400 MHz) δ 7.45 – 7.40 (m, 2H), 7.33 (m, 2H), 7.26 – 7.21 (m, 1H), 6.04 (m, 1H), 4.29 (m, 1H), 3.01 (m, 1H), 2.89 (m, 1H), 2.43 (m, 1H), 1.74 (s, 1H), 1.58 – 1.48 (m, 1H), 1.48 – 1.41 (m, 1H), 1.40 – 1.31 (m, 1H), 1.24 – 1.15 (m, 1H), 0.91 (t, *J* = 7.2 Hz, 3H).

^{13}C NMR ($CDCl_3$, 100 MHz) δ 144.8, 135.8, 128.4, 127.2, 126.1, 122.5, 76.8, 55.3, 41.9, 33.2, 20.9, 14.3.

HRMS-(ESI+) for $C_{14}H_{19}O$ $[M+H]^+$: calculated: 203.1416; found: 203.1444.

(1*R*,2*R*)-3-phenyl-2-propylcyclopent-3-en-1-ol (28)

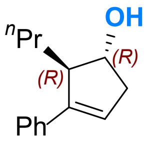

(*R*,*R*)-**28**

The product (*R,R*)-**28** was purified by flash chromatography using a mixture of petroleum ether and ethyl acetate (50:50) as eluent. It was isolated as a colorless oil (35 mg, 86%).

e.r= 97:3 Optical rotation: $[\alpha]_D^{25}$ -20.3 (c=0.6, $CHCl_3$).

(1*R,2*R**)-2-isopropyl-3-phenylcyclopent-3-en-1-ol (29)**

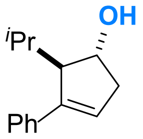

29

The product was purified by flash chromatography using a mixture of petroleum ether and ethyl acetate (50:50) as eluent. It was isolated as a colorless oil (30 mg, 75%).

^{1}H NMR ($CDCl_3$, 400 MHz) δ 7.44 – 7.37 (m, 2H), 7.32 (m, 2H), 7.25 – 7.20 (m, 1H), 6.02 (m 1H), 4.40 (m, 1H), 3.03 (m, 1H), 2.81 (m, 1H), 2.42 (m, 1H), 1.96 (m, 1H), 1.61 (s, 1H), 1.07 (d, *J* = 6.9 Hz, 3H), 0.66 (d, *J* = 7.0 Hz, 3H).

^{13}C NMR ($CDCl_3$, 100 MHz) δ 143.9, 136.5, 128.4, 127.1, 126.4, 123.9, 73.1, 62.0, 43.5, 28.1, 21.4, 17.1.

HRMS-(ESI+) for $C_{20}H_{30}BO_2$ $[M+H]^+$: calculated: 203.1416; found: 203.1435.

(1*R*,2*R*)-2-isopropyl-3-phenylcyclopent-3-en-1-ol (*R,R*)-**(29)**

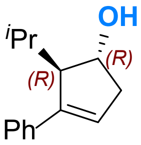

(*R*,*R*)-**29**

The product was purified by flash chromatography using a mixture of petroleum ether and ethyl acetate (50:50) as eluent. It was isolated as a colorless oil (80%).

e.r= 95:5 Optical rotation: $[\alpha]_D^{25}$ -181.95 (c=0.1, $CHCl_3$)

(1*R*,2*R*,3*R*,5*S*)-1,2-diphenylbicyclo[3.1.0]hexan-3-ol (30)

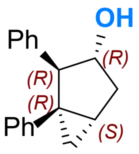

(1*R*,2*R*,3*R*,5*S*)-30

The product (1*R*,2*R*,3*R*,5*S*)-**30** was purified by flash chromatography using as eluent a mixture of petroleum ether/ethyl acetate (50:50). The product was isolated as a colorless oil (36 mg, 71%).

^{1}H NMR ($CDCl_3$, 400 MHz) δ 7.17 – 6.90 (m, 10H), 4.28 (d, *J* = 6.5 Hz, 1H), 3.60 (s, 1H), 2.61 (ddd, *J* = 14.3, 6.5, 4.7Hz, 1H), 2.27 – 2.18 (m, 1H), 1.94 (d, *J* = 14.3 Hz, 1H), 1.62 (t, *J* = 4.2 Hz, 1H), 1.58 (s, 1H), 0.83 (m, 1H).

^{13}C NMR ($CDCl_3$, 100 MHz) δ 143.5, 142.8, 128.2, 127.9, 127.7, 125.9, 124.9, 81.0, 60.2, 37.6, 37.1, 24.7, 23.4.

HRMS-(ESI+) for $C_{18}H_{19}O$ $[M+H]^+$: calculated: 251.1417; found: 251.1443.

Optical rotation: $[\alpha]_D^{25}$ -135.7 (c=0.6, $CHCl_3$).

(1S,2S,3R,5S)-1,2-diphenyl-6-oxabicyclo[3.1.0]hexan-3-ol ((1*S*,2*S*,3*R*,5*S*)-31)

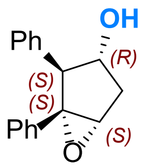

(1*S*,2*S*,3*R*,5*S*)-31

The product (**1*S*,2*S*,3*R*,5*S*)-31** was purified by flash chromatography using a mixture of *n*-hexane and ethyl acetate (90:10) as eluent. The product was isolated as a colorless oil (126.6 mg, 91% yield) with >20:1 dr and 94:6 er.

^{1}H NMR ($CDCl_3$, 300 MHz) δ 7.39 – 7.01 (m, 10H), 4.39 (s, 1H), 4.06 (dd, *J* = 11.6, 5.8 Hz, 1H), 3.87 (s, 1H), 3.08 (d, *J* = 12.2 Hz, 1H), 2.58 (dd, *J* = 15.3, 5.8 Hz, 1H), 2.31 (d, *J* = 15.4 Hz, 1H).

^{13}C NMR ($CDCl_3$, 75 MHz) δ 139.3, 134.7, 128.9, 128.3, 128.0, 127.0, 78.1, 70.7, 65.2, 57.6, 36.5.

HRMS-(APCI) for $C_{17}H_{17}O_2$ [M+H]$^+$: calculated: 253.1223; found: 253.1220.

Specific rotation: $[\alpha]_D^{21}$ -101.9 (*c* = 0.71, $CHCl_3$).

Enantiomeric purity was determined by chiral uHPLC analysis [Chiralpak IB N-3, T_{oven}: 40 °C, flow: 1 mL/min; 10% IPA, 90% *n*-Hexane, λ = 234 nm, minor enantiomer t_R = 2.17 min, major enantiomer t_R = 3.53 min].

(1S,2R,3R,5S)-1,2-diphenyl-6-azabicyclo[3.1.0]hexan-3-ol ((1*S*,2*R*,3*R*,5*S*)-32)

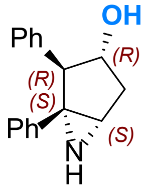

(1*S*,2*R*,3*R*,5*S*)-32

The product (**1*S*,2*R*,3*R*,5*S*)-32** was purified by flash chromatography using a mixture of *n*-hexane and ethyl acetate (90:10 to 60:40) as eluent. The product was isolated as a yellow oil (15,7 mg, 63% yield) in >20:1 dr and 96.5:3.5 er.

^{1}H NMR ($CDCl_3$, 500 MHz) δ 7.25 – 7.04 (m, 10H), 3.94 (d, *J* = 5.5 Hz, 1H), 3.73 (s, 1H), 3.54 (s, 1H), 2.36 (ddd, *J* = 14.3, 5.6, 2.3 Hz, 1H), 2.05 (d, *J* = 14.3 Hz, 1H).

^{13}C NMR ($CDCl_3$, 126 MHz) δ 140.4, 138.3, 128.7, 128.5, 128.2, 127.7, 127.4, 126.4, 78.8, 57.8, 51.6, 42.1, 35.8.

HRMS-(APCI) for $C_{17}H_{18}NO$ [M+H]$^+$: calculated: 252.1383; found: 252.1376.

Specific rotation $[\alpha]_D^{21}$ -88.1 (*c* = 0.65, $CHCl_3$).

Enantiomeric purity was determined by chiral uHPLC analysis [Chiralpak IB N-3, T_{oven}: 40 °C, flow: 1 mL/min; 10% IPA, 90% *n*-Hexane, λ = 230 nm, minor enantiomer t_R = 2.62 min, major enantiomer t_R = 2.97 min].

^{1}H, ^{13}C, ^{11}B, ^{19}F Spectra for products

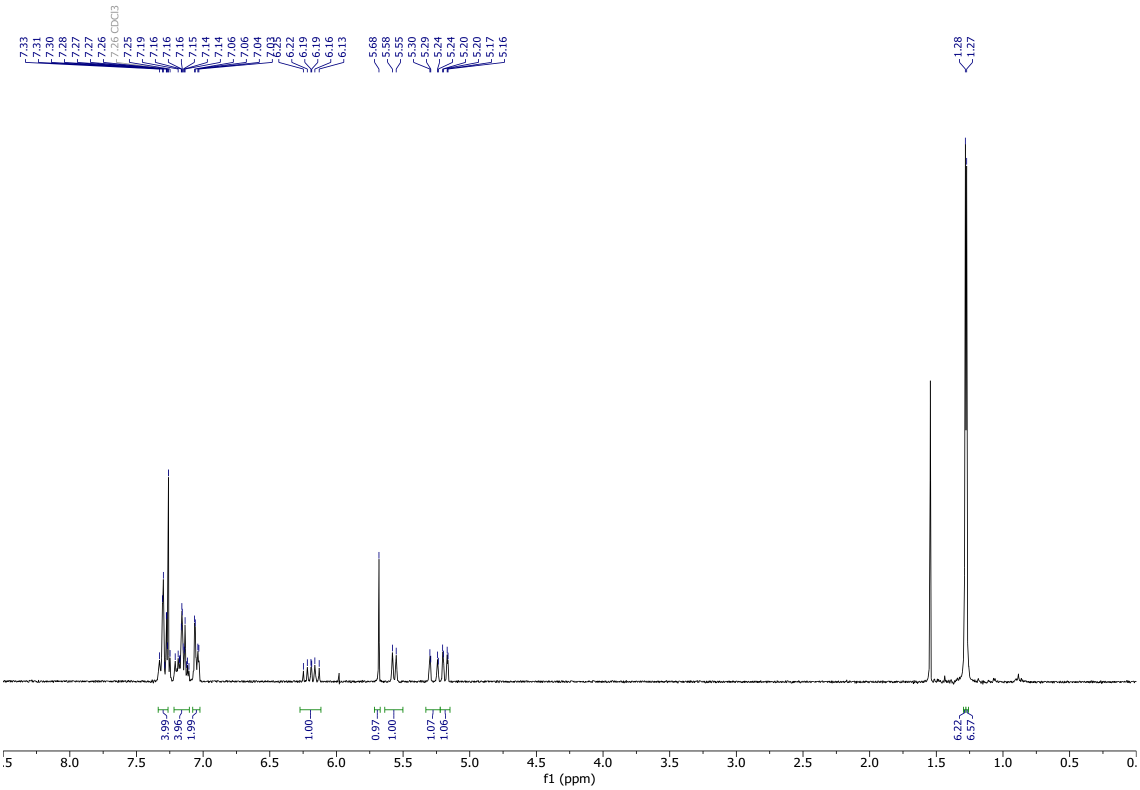

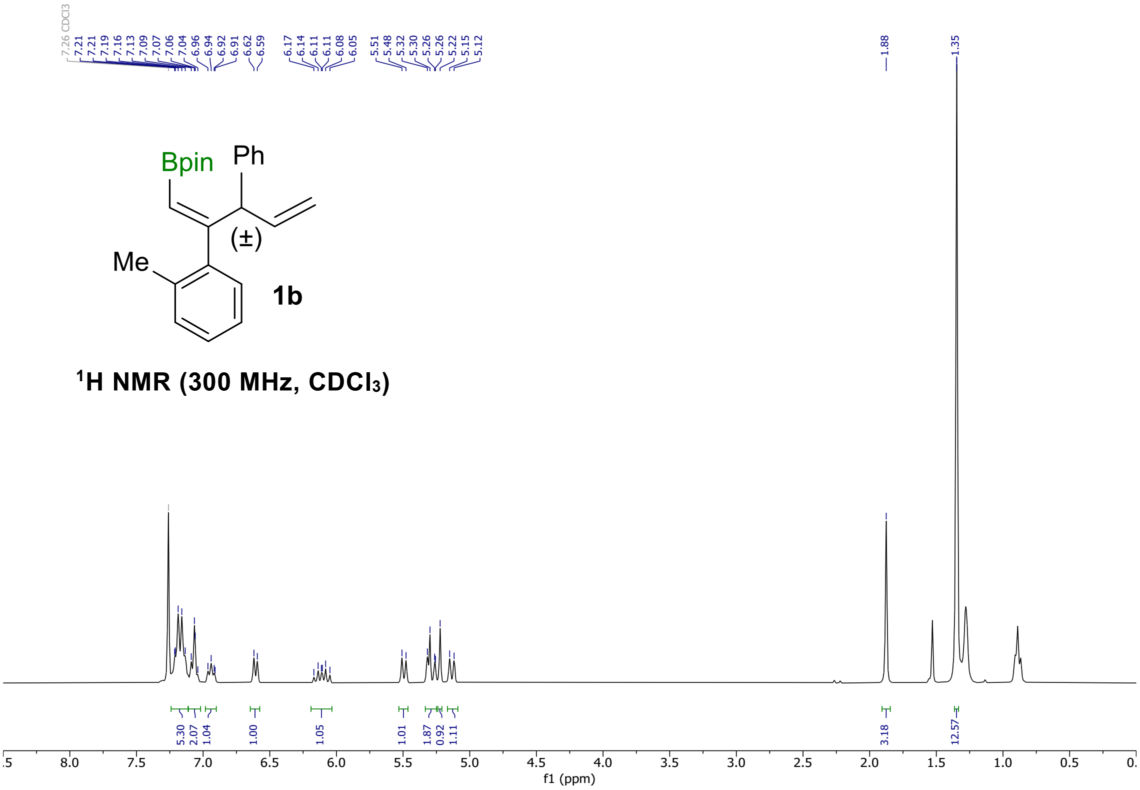

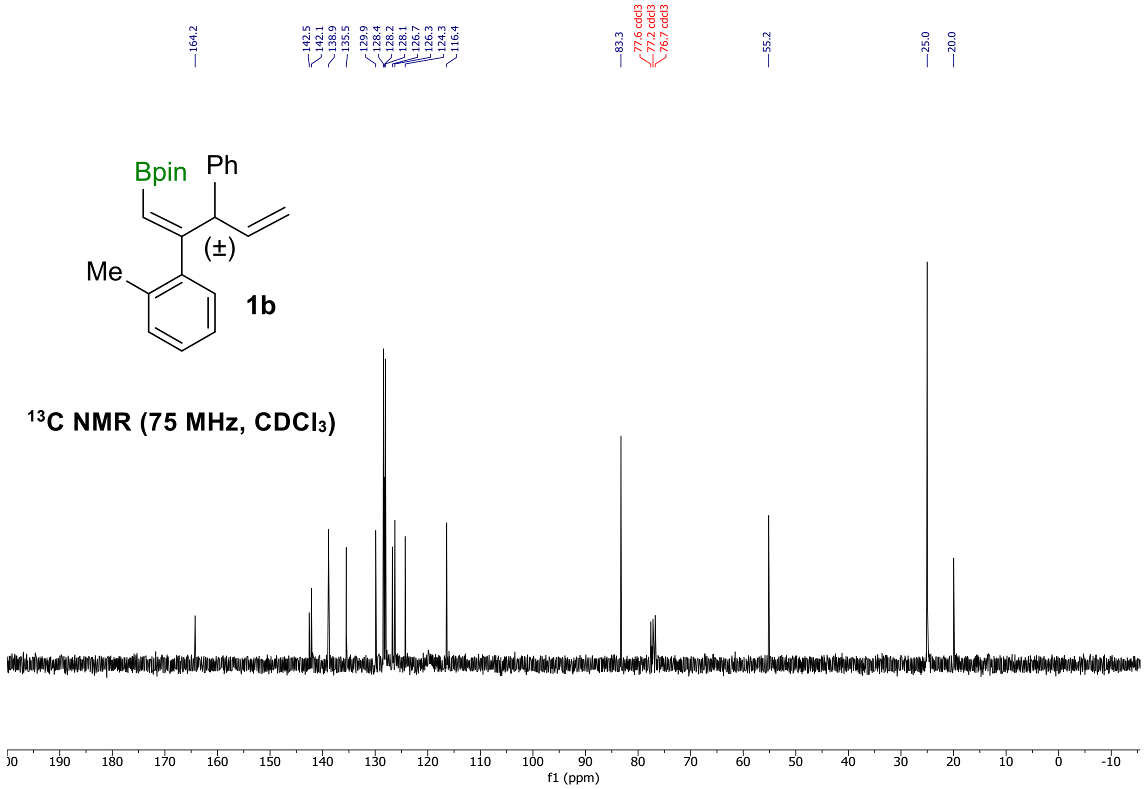
164.2
142.5
142.1
138.9
135.5
129.9
128.4
128.2
128.1
126.7
126.3
124.3
116.4
83.3
77.6 cdcl3
77.2 cdcl3
76.7 cdcl3
55.2
25.0
20.0
Bpin
Ph
(±)
Me
1b
^{13}C NMR (75 MHz, CDCl$_3$)
f1 (ppm)

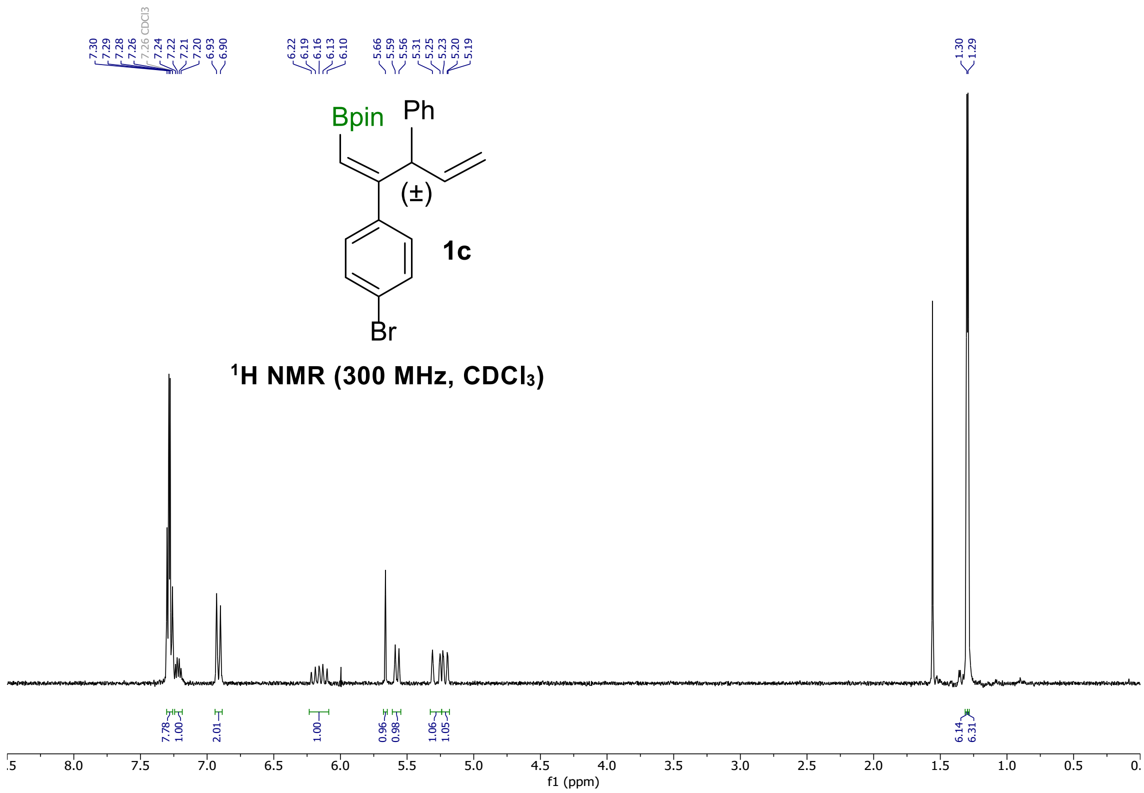
7.30
7.29
7.28
7.26
7.26 CDCl3
7.24
7.22
7.21
7.20
6.93
6.90
6.22
6.19
6.16
6.13
6.10
5.66
5.59
5.56
5.31
5.25
5.23
5.20
5.19
1.30
1.29
Bpin
Ph
(±)
1c
Br
^{1}H NMR (300 MHz, CDCl$_3$)
7.78
1.00
2.01
1.00
0.96
0.98
1.06
1.05
6.14
6.31
f1 (ppm)

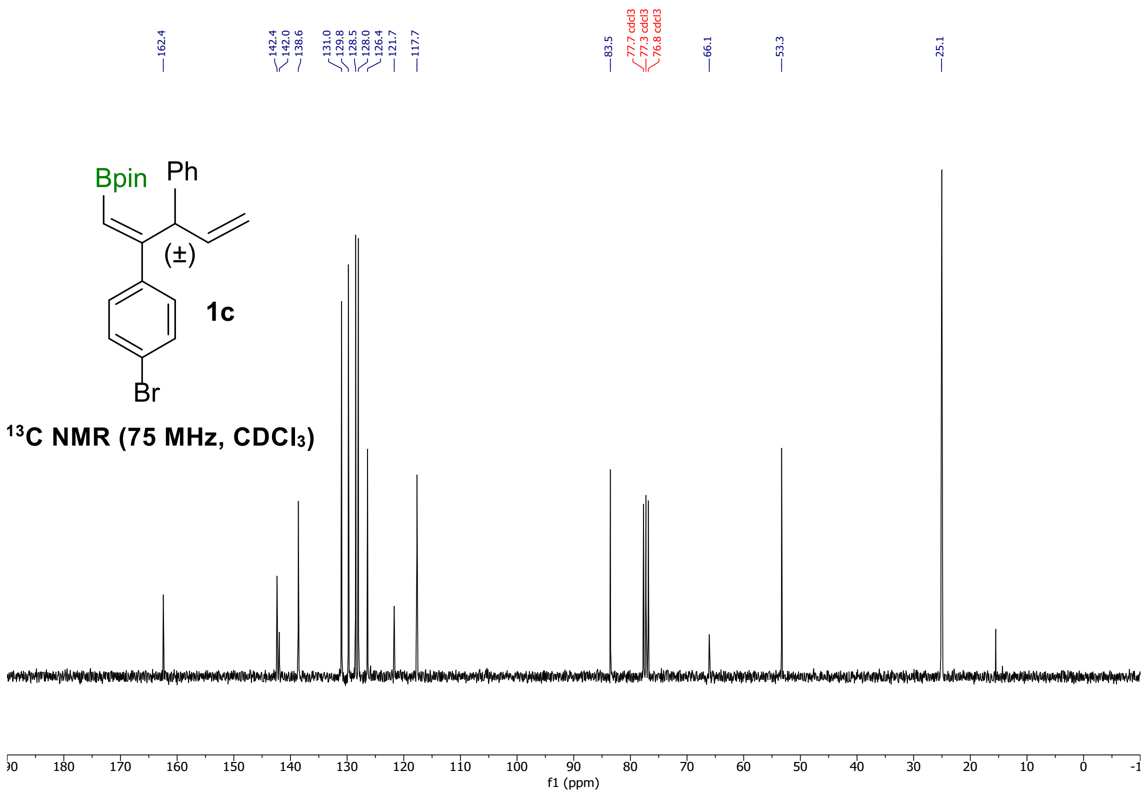
162.4
142.4
142.0
138.6
131.0
129.8
128.5
128.0
126.4
121.7
117.7
83.5
77.7 cdcl3
77.3 cdcl3
76.8 cdcl3
66.1
53.3
25.1
Bpin
Ph
(±)
1c
Br
13C NMR (75 MHz, CDCl3)
f1 (ppm)

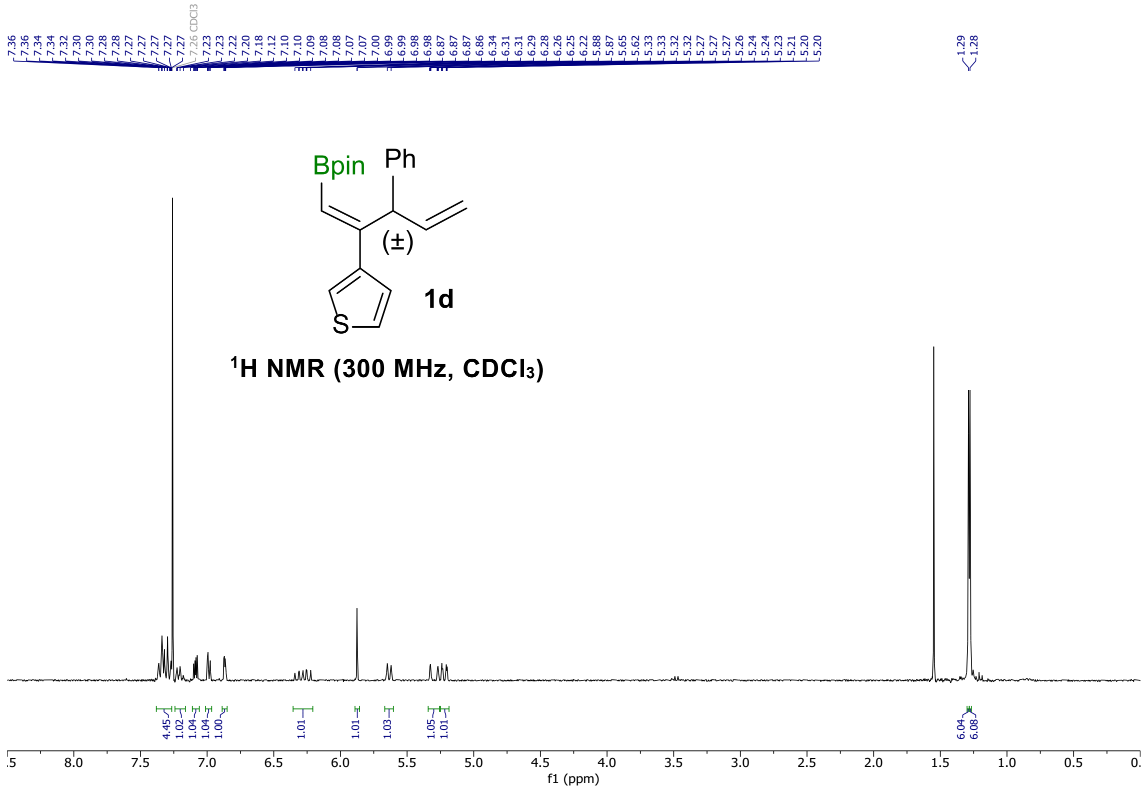
7.36 7.36 7.34 7.34 7.32 7.30 7.30 7.28 7.28 7.27 7.27 7.27 7.27 7.26 CDCl3 7.23 7.23 7.22 7.20 7.18 7.12 7.10 7.10 7.09 7.08 7.08 7.07 7.07 7.00 6.99 6.99 6.98 6.98 6.87 6.87 6.87 6.86 6.34 6.31 6.31 6.29 6.28 6.26 6.25 6.22 5.88 5.87 5.65 5.62 5.33 5.33 5.32 5.32 5.27 5.27 5.27 5.26 5.24 5.24 5.23 5.21 5.20 5.20
1.29
1.28
Bpin
Ph
(±)
S
1d
1H NMR (300 MHz, CDCl3)
4.45 1.02 1.04 1.04 1.00 1.01 1.01 1.03 1.05 1.01 6.04 6.08
f1 (ppm)

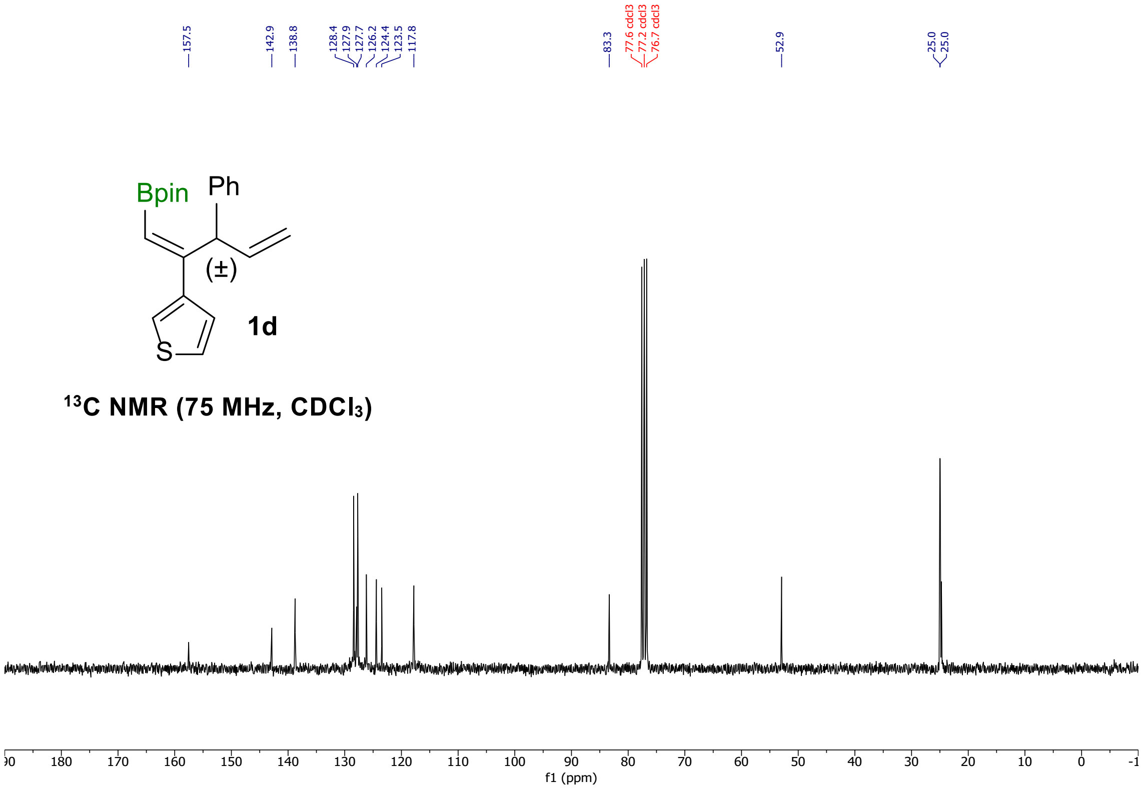
157.5
142.9
138.8
128.4
127.9
127.7
126.2
124.4
123.5
117.8
83.3
77.6 cdcl3
77.2 cdcl3
76.7 cdcl3
52.9
25.0
25.0
Bpin
Ph
(±)
S
1d
^{13}C NMR (75 MHz, $CDCl_3$)
f1 (ppm)

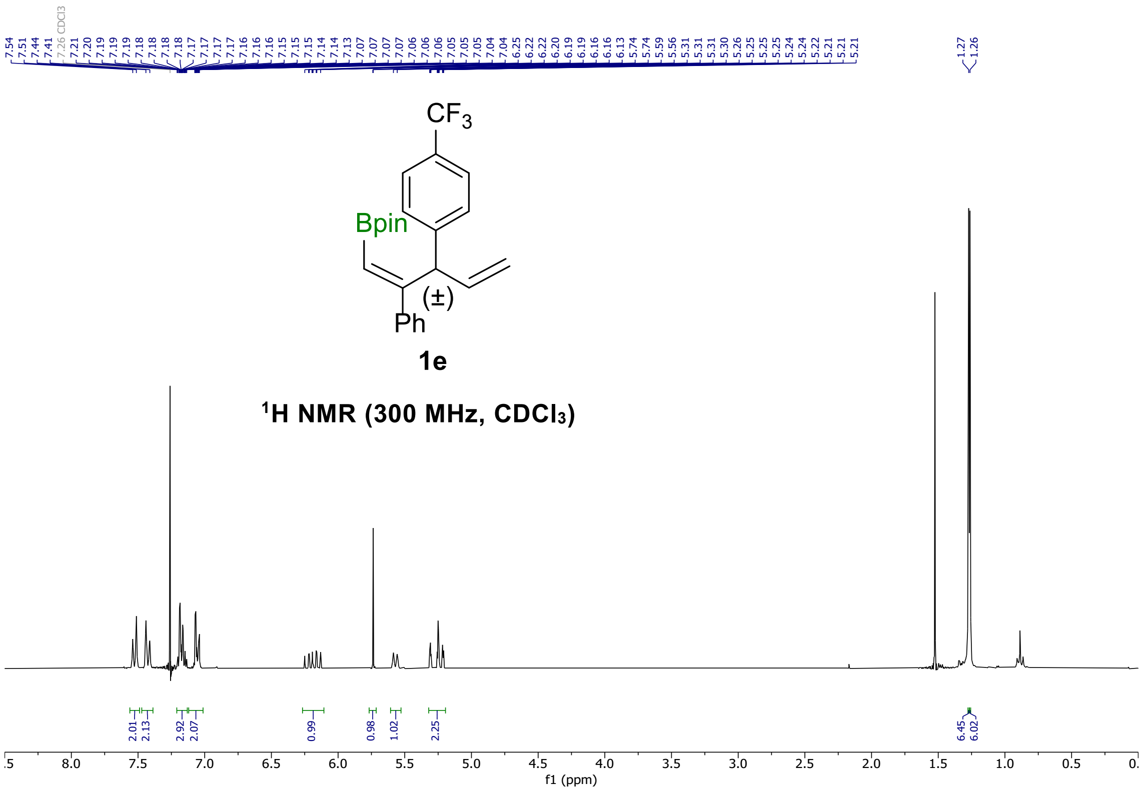
7.54
7.51
7.44
7.41
7.26 CDCl3
1.27
1.26
CF_3
Bpin
(±)
Ph
1e
^{1}H NMR (300 MHz, $CDCl_3$)
2.01
2.13
2.92
2.07
0.99
0.98
1.02
2.25
6.45
6.02
f1 (ppm)

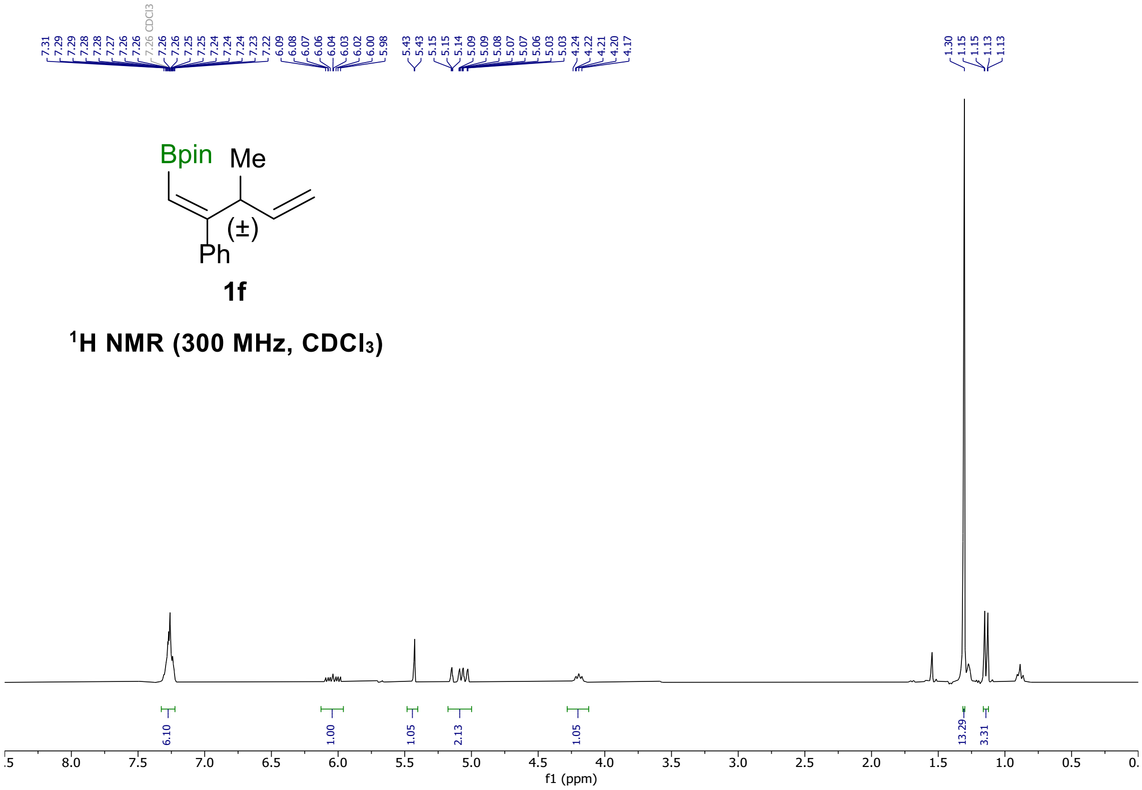
Bpin
Me
(±)
Ph
1f
^{1}H NMR (300 MHz, $CDCl_3$)
f1 (ppm)

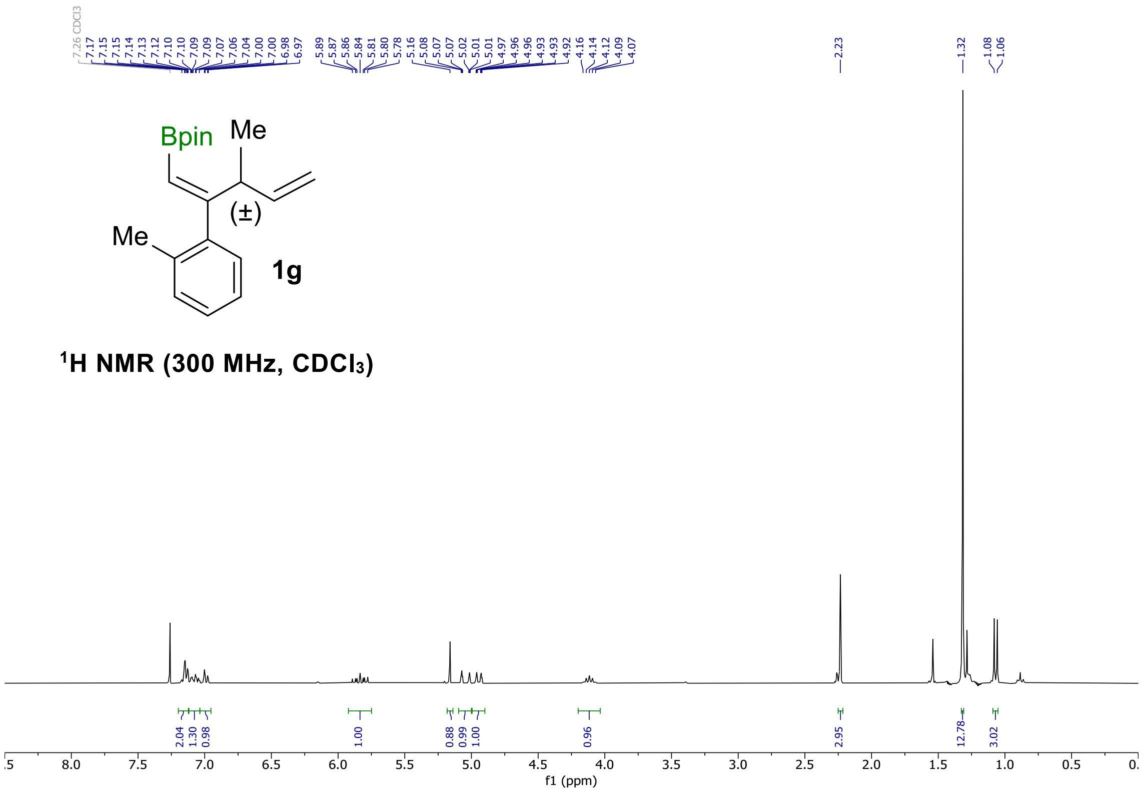
Bpin
Me
(±)
Me
1g
^{1}H NMR (300 MHz, $CDCl_3$)
f1 (ppm)

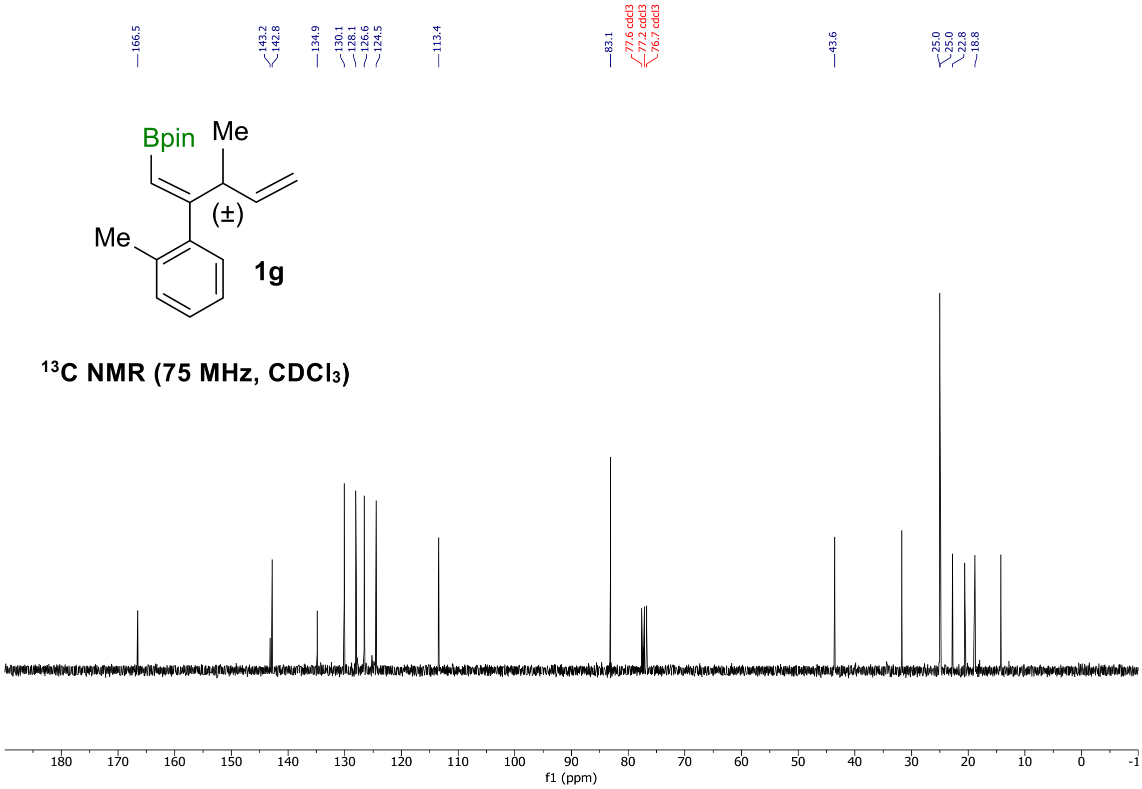

^{13}C NMR (75 MHz, CDCl$_3$)

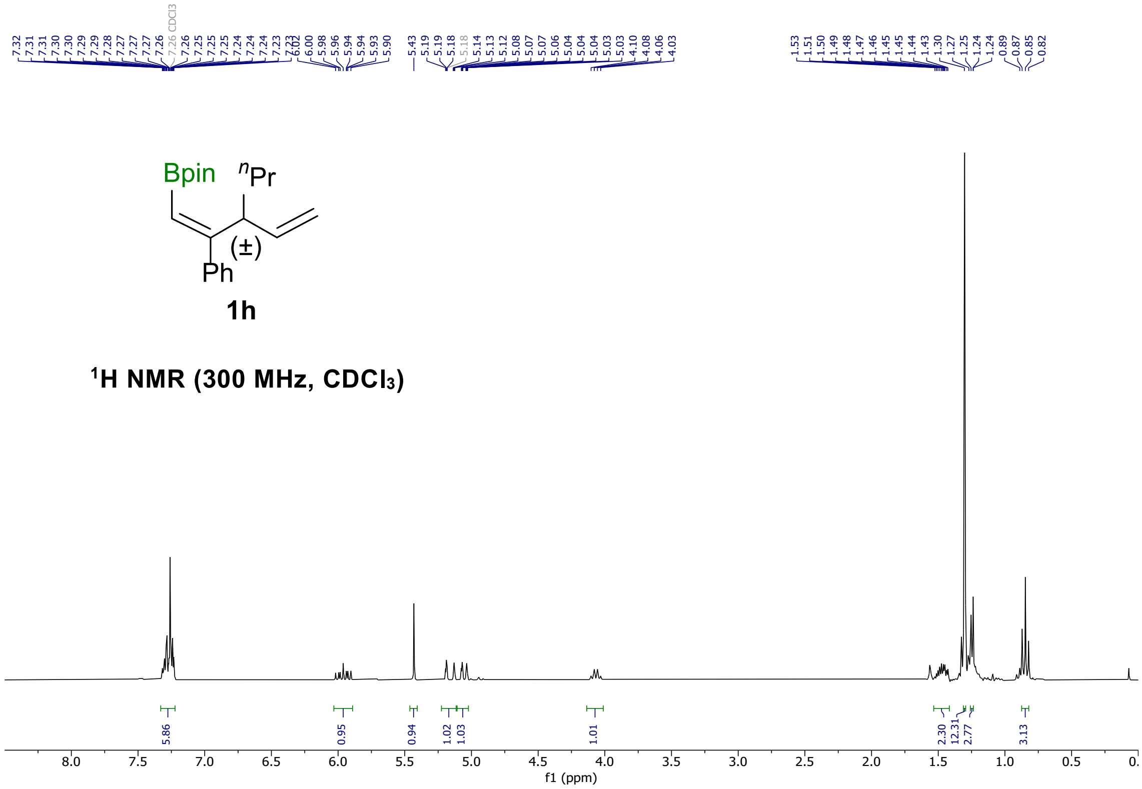

^{1}H NMR (300 MHz, CDCl$_3$)

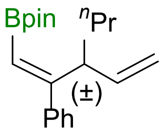

1h

^{13}C NMR (75 MHz, CDCl$_3$)

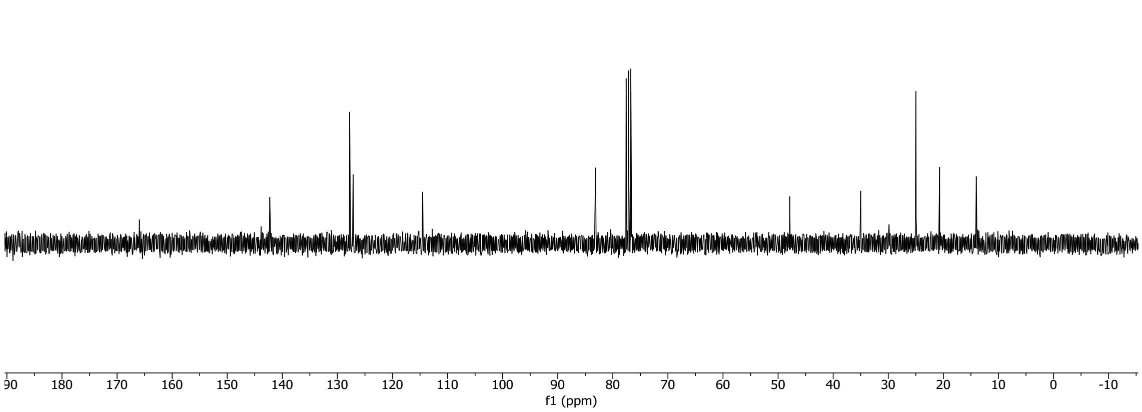

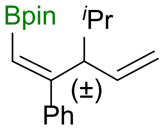

1i

^{1}H NMR (300 MHz, CDCl$_3$)

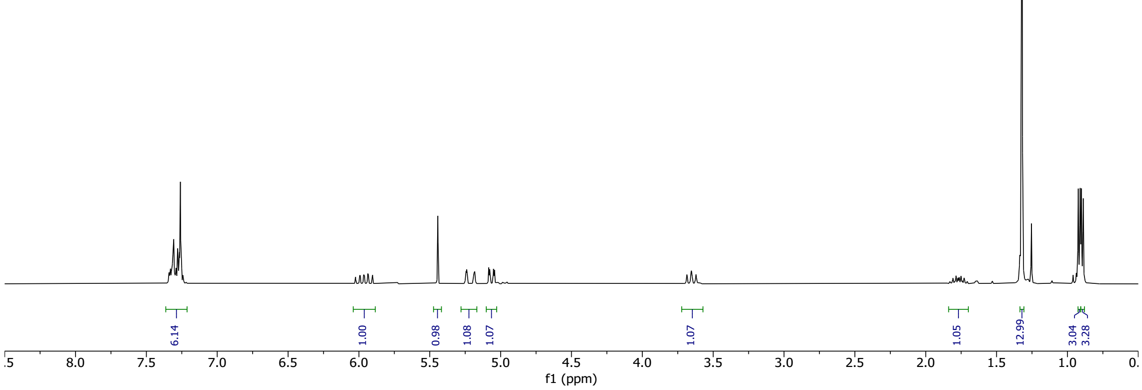

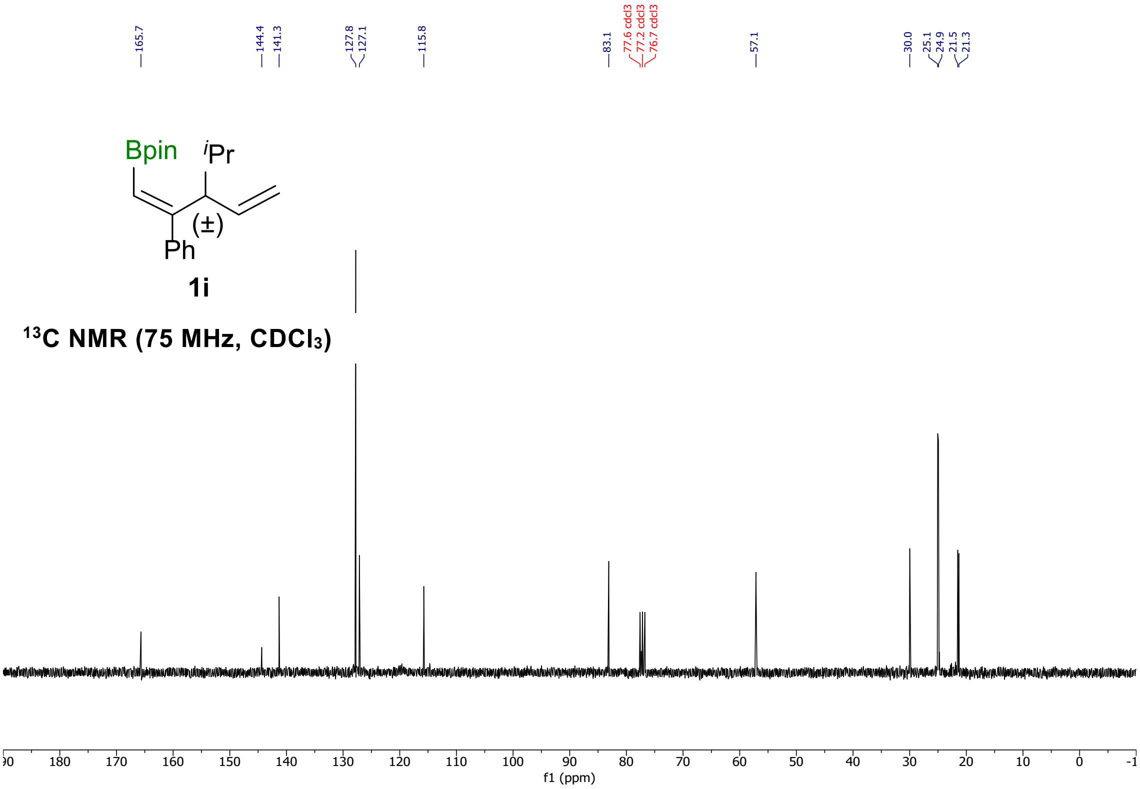
165.7
144.4
141.3
127.8
127.1
115.8
83.1
77.6 cdcl3
77.2 cdcl3
76.7 cdcl3
57.1
30.0
25.1
24.9
21.5
21.3
Bpin
iPr
(±)
Ph
1i
13C NMR (75 MHz, CDCl3)
f1 (ppm)

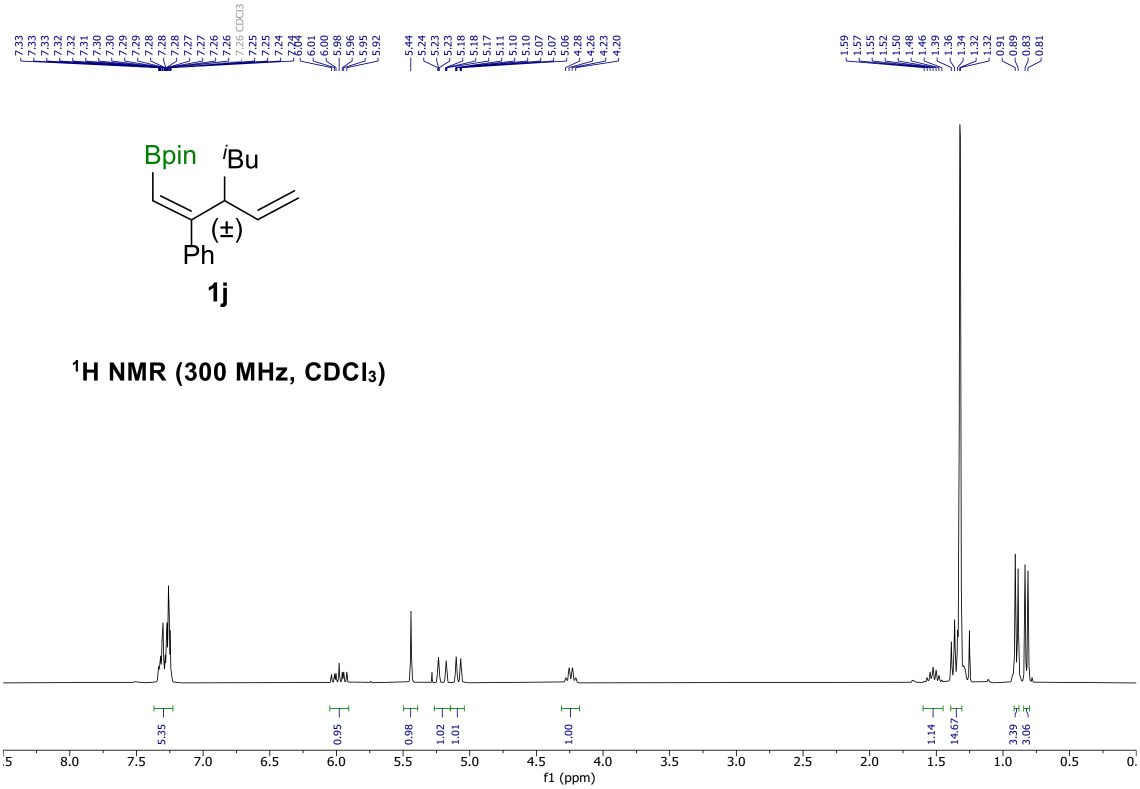
Bpin
iBu
(±)
Ph
1j
1H NMR (300 MHz, CDCl3)
5.35
0.95
0.98
1.02
1.01
1.00
1.14
14.67
3.39
3.06
f1 (ppm)

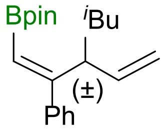

1j

^{13}C NMR (75 MHz, CDCl$_3$)

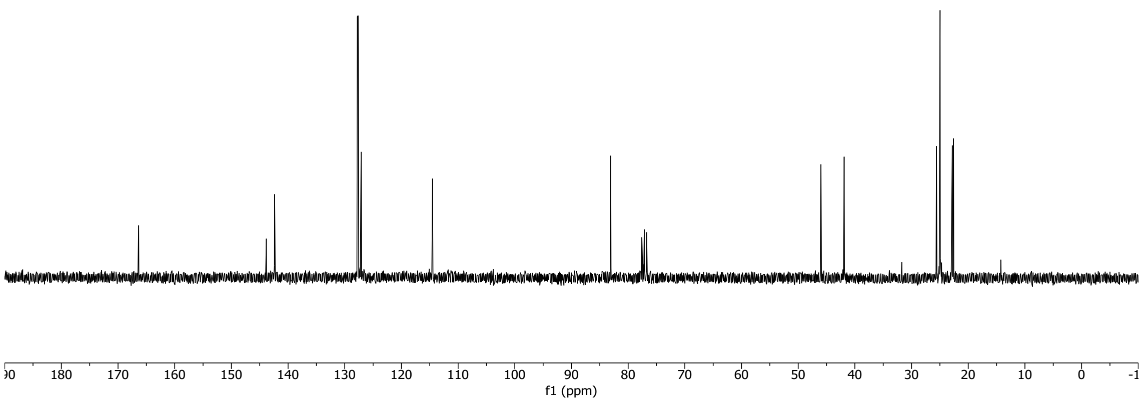

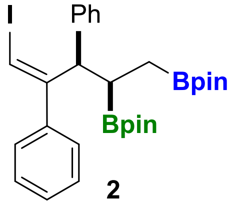

2

^{1}H NMR (400 MHz, CDCl$_3$)

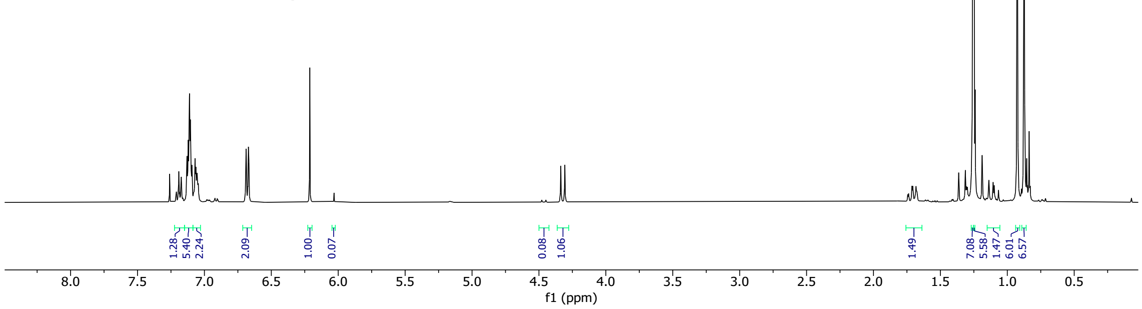

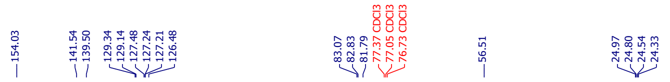

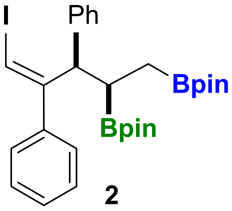

2

^{13}C NMR (100 MHz, CDCl$_3$)

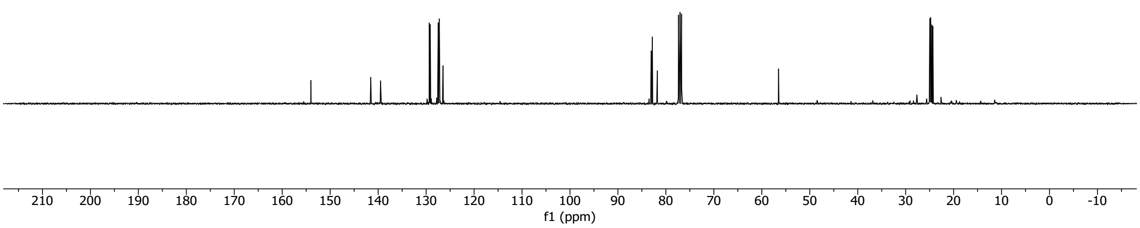

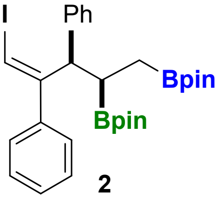

2

^{11}B NMR (129 MHz, CDCl$_3$)

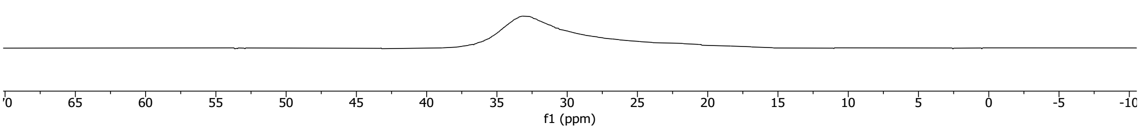

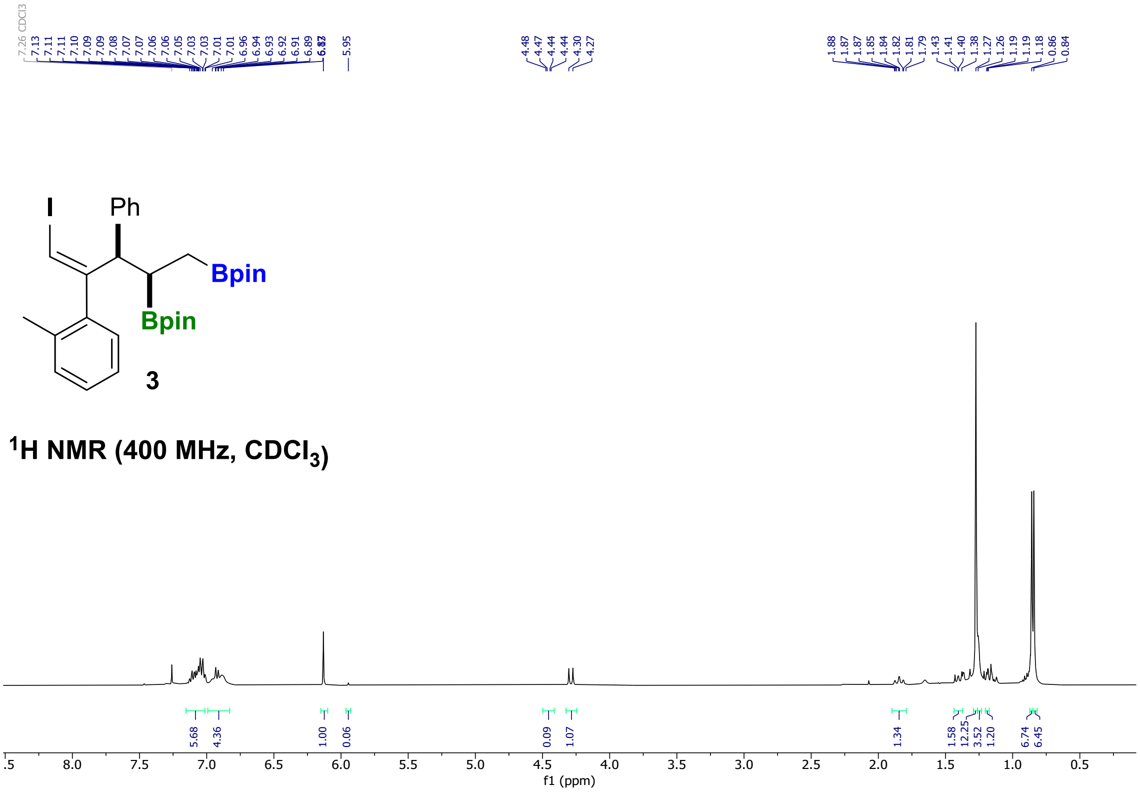
7.26 CDCl3
7.13 7.11 7.11 7.10 7.09 7.09 7.08 7.07 7.07 7.06 7.06 7.05 7.03 7.03 7.01 7.01 6.96 6.94 6.93 6.92 6.91 6.89 6.87 5.95
4.48 4.47 4.44 4.44 4.30 4.27
1.88 1.87 1.87 1.85 1.84 1.82 1.81 1.79 1.43 1.41 1.40 1.38 1.27 1.26 1.19 1.19 1.18 0.86 0.84
I
Ph
Bpin
Bpin
3
1H NMR (400 MHz, CDCl3)
5.68 4.36 1.00 0.06 0.09 1.07 1.34 1.58 12.25 3.52 1.20 6.74 6.45
f1 (ppm)

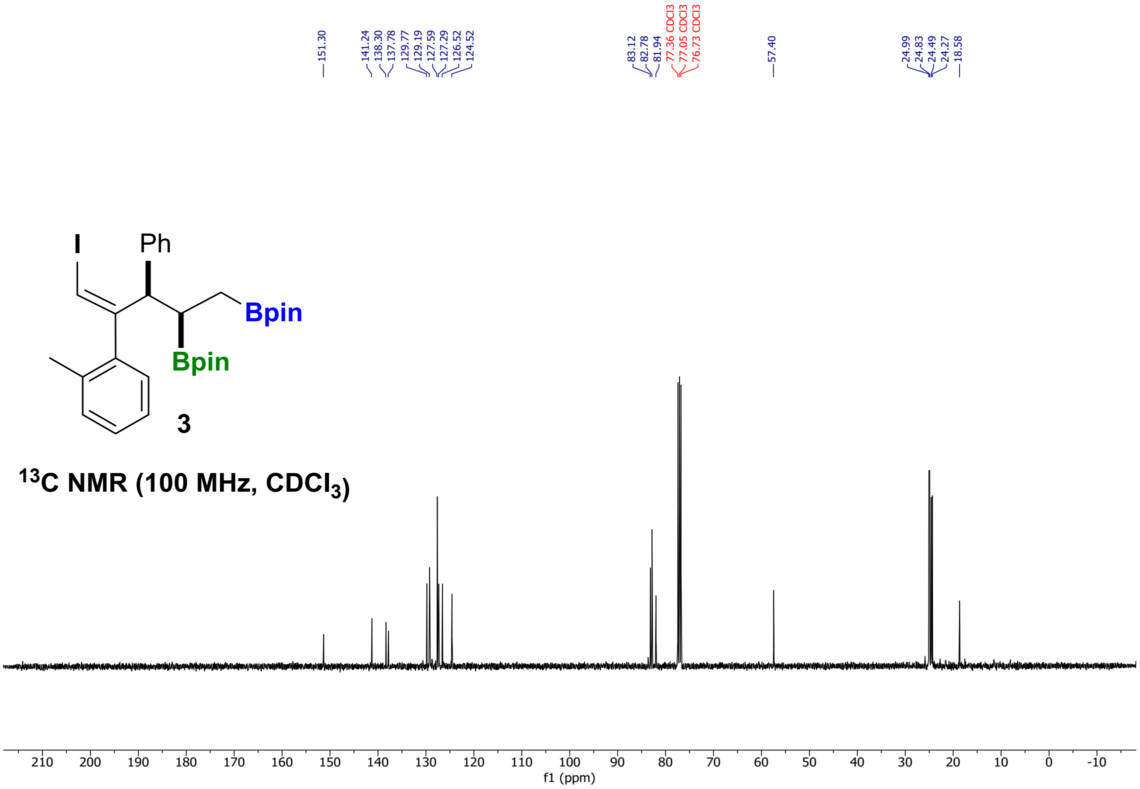
151.30
141.24 138.30 137.78 129.77 129.19 127.59 127.29 126.52 124.52
83.12 82.78 81.94 77.36 CDCl3 77.05 CDCl3 76.73 CDCl3
57.40
24.99 24.83 24.49 24.27 18.58
I
Ph
Bpin
Bpin
3
13C NMR (100 MHz, CDCl3)
f1 (ppm)

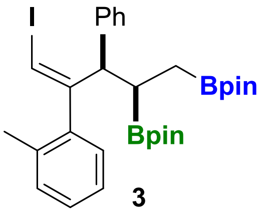

3

^{11}B NMR (129 MHz, $CDCl_3$)

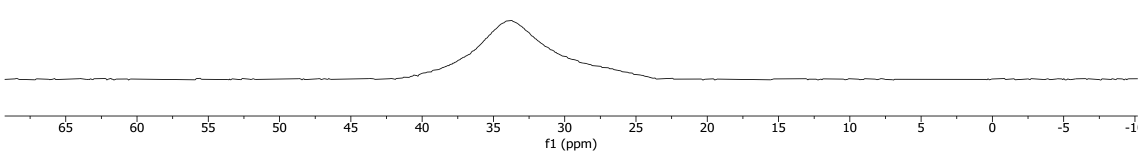

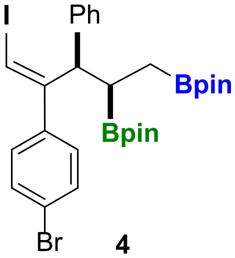

4

^{1}H NMR (400 MHz, $CDCl_3$)

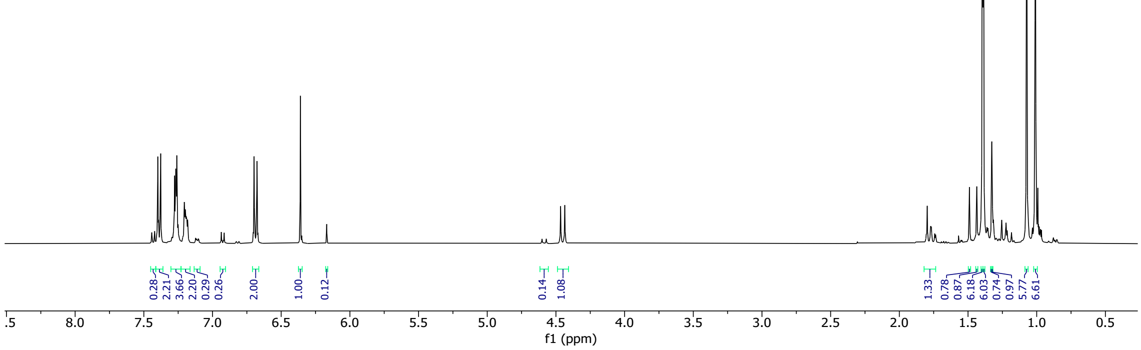

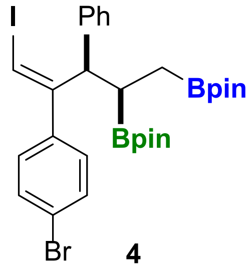

4

^{13}C NMR (100 MHz, CDCl$_3$)

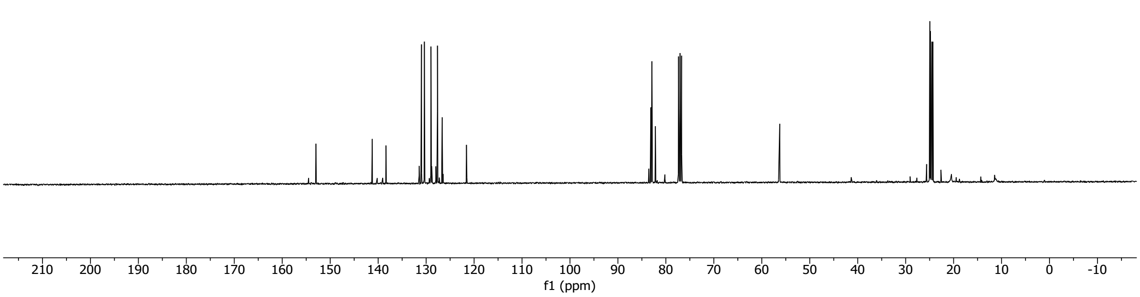

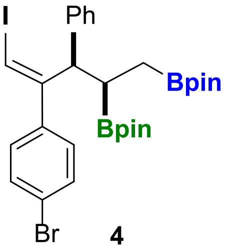

4

^{11}B NMR (129 MHz, CDCl$_3$)

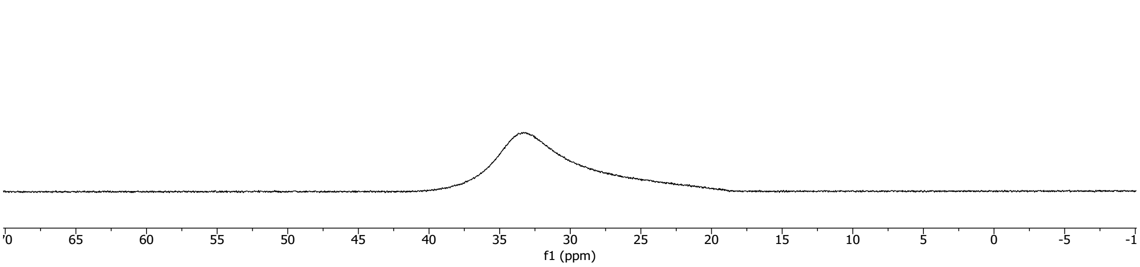

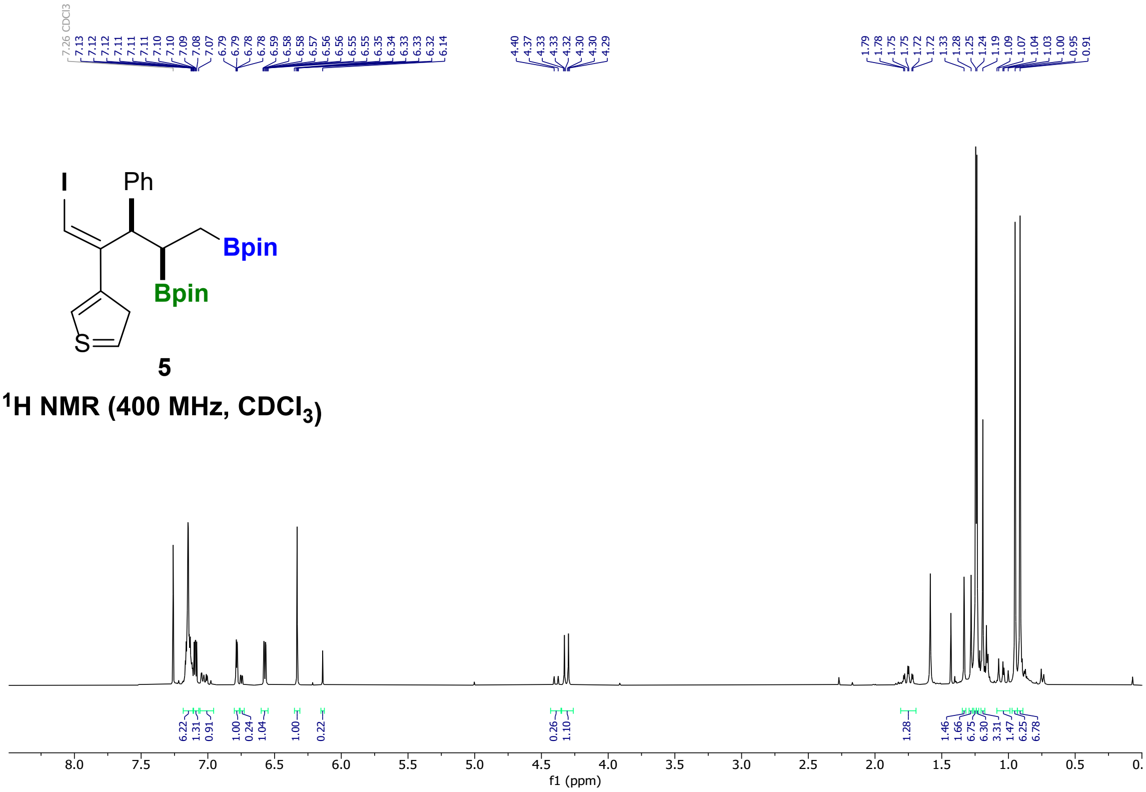

5

^{1}H NMR (400 MHz, $CDCl_3$)

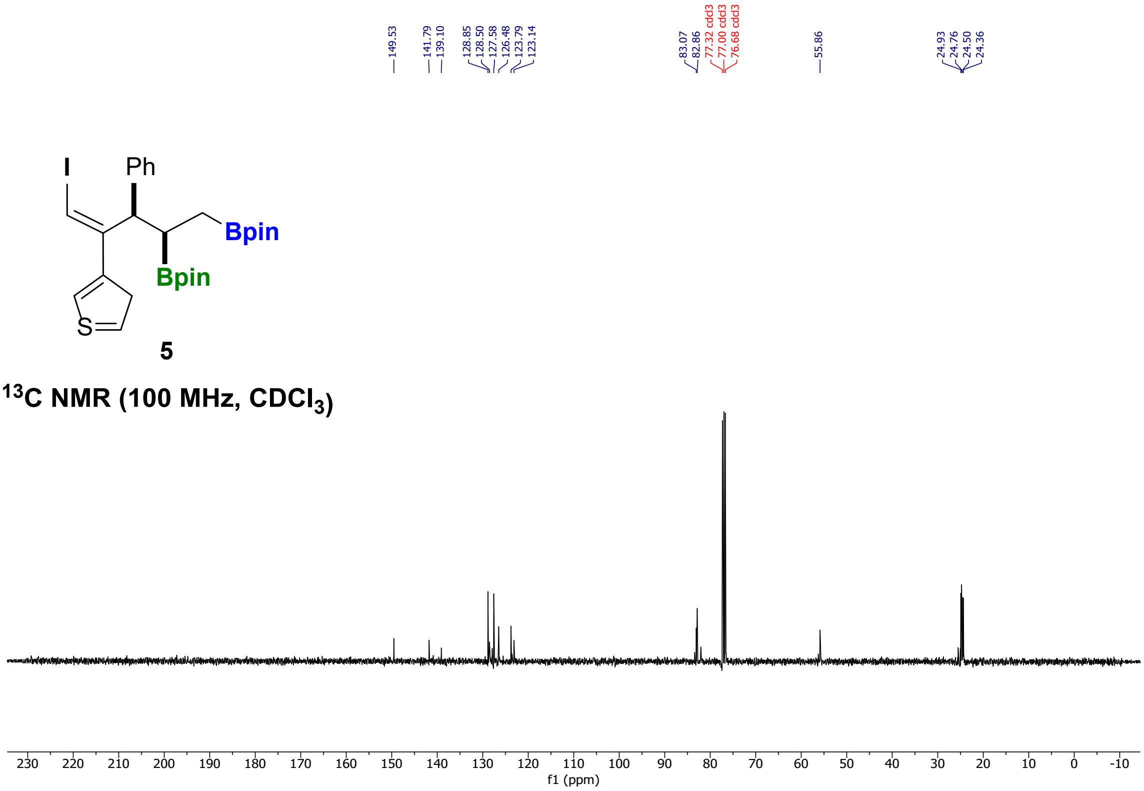

5

^{13}C NMR (100 MHz, $CDCl_3$)

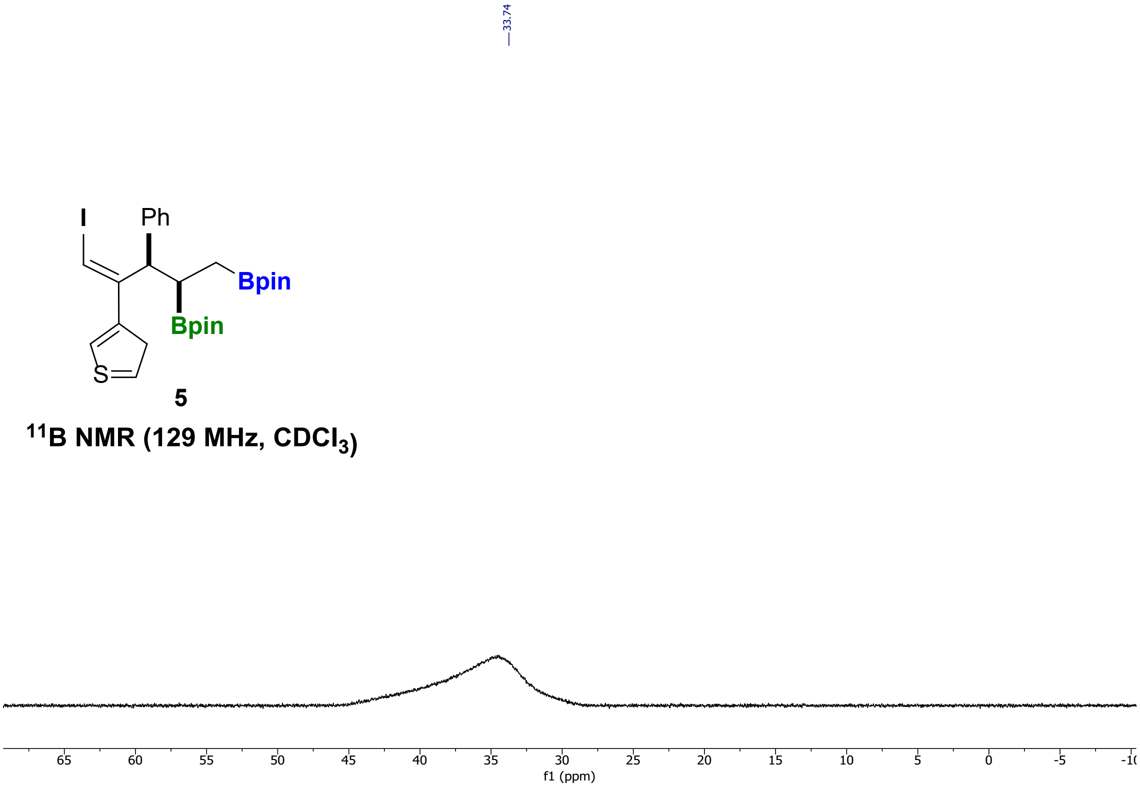

5

^{11}B NMR (129 MHz, $CDCl_3$)

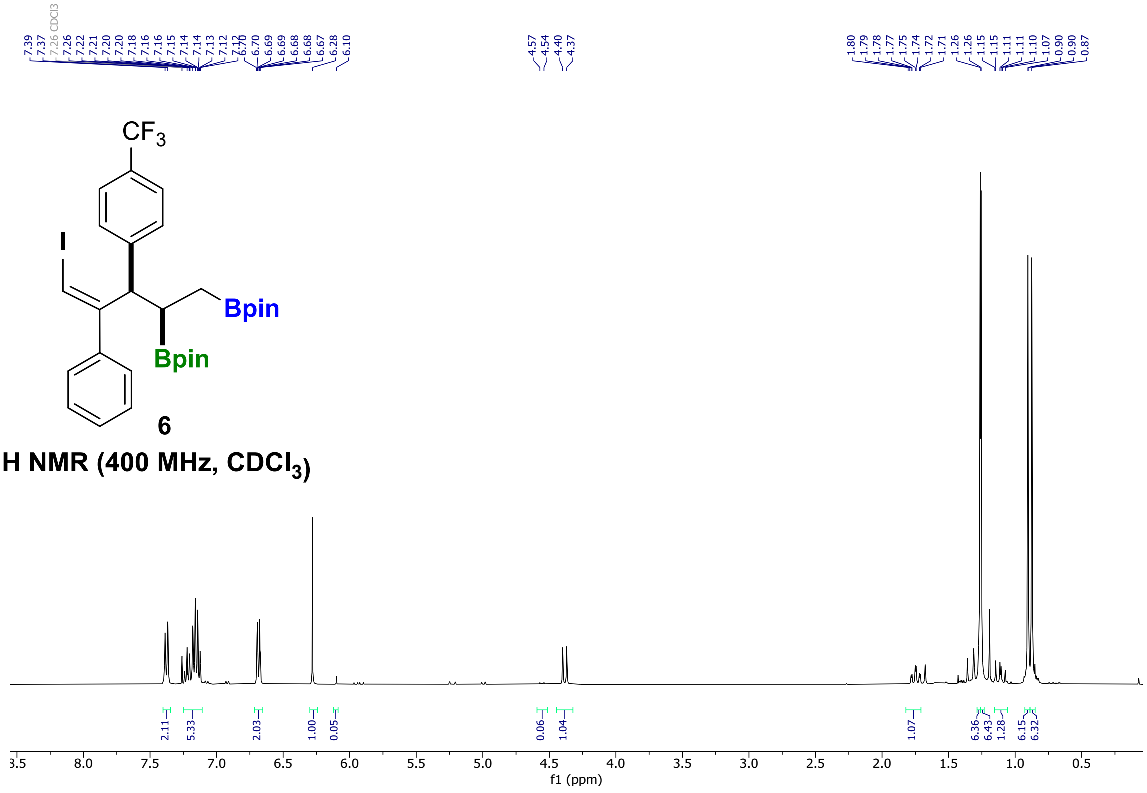

6

^{1}H NMR (400 MHz, $CDCl_3$)

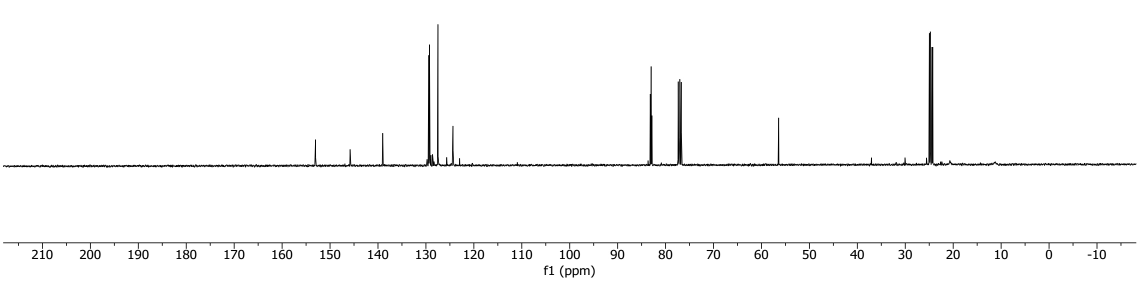

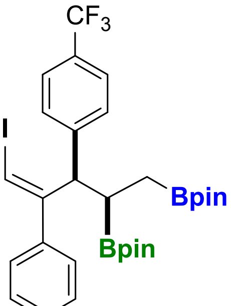

6

^{13}C NMR (100 MHz, $CDCl_3$)

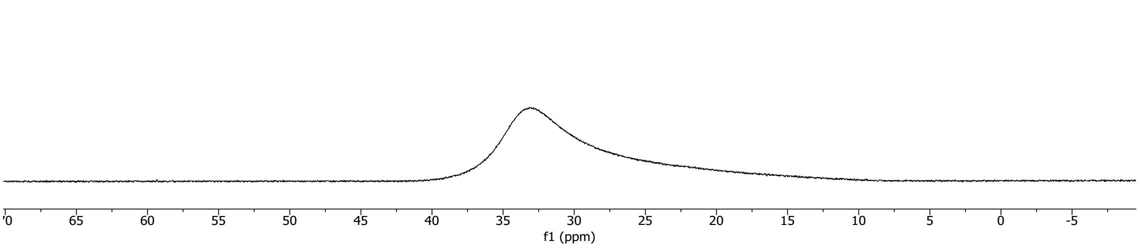

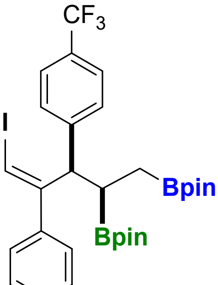

6

^{11}B NMR (129 MHz, $CDCl_3$)

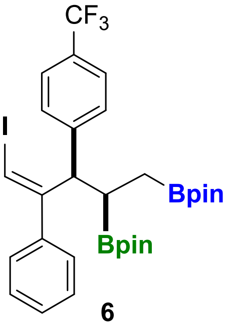

6

^{19}F NMR (377 MHz, CDCl$_3$)

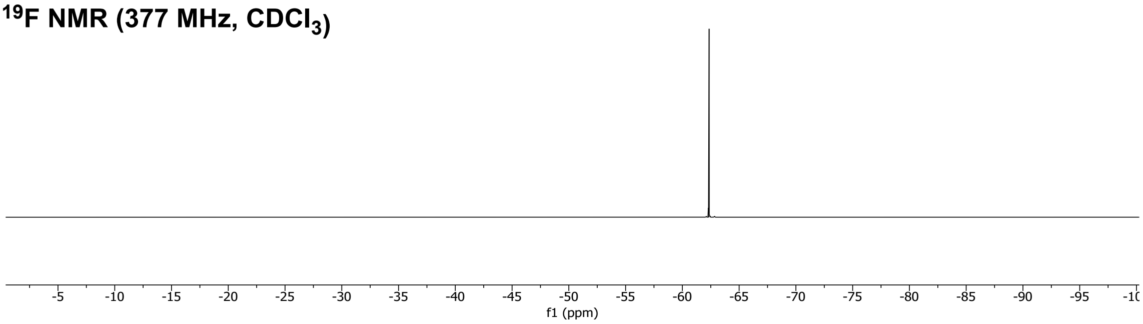

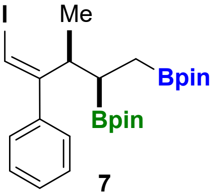

7

^{1}H NMR (400 MHz, CDCl$_3$)

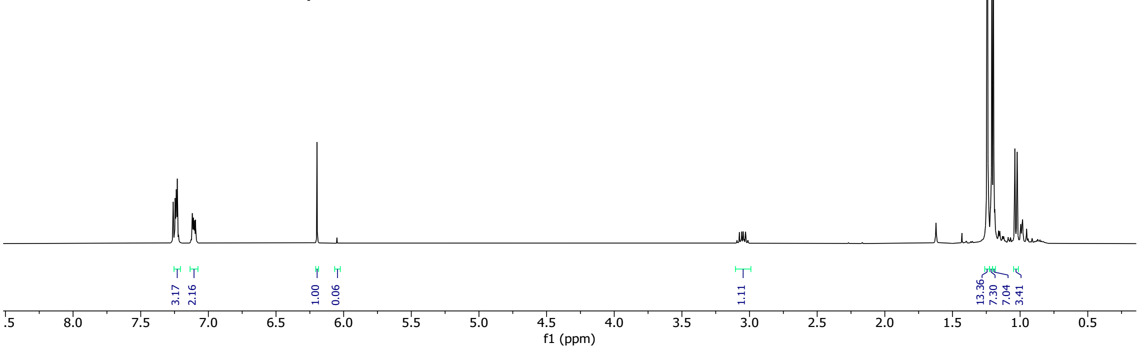

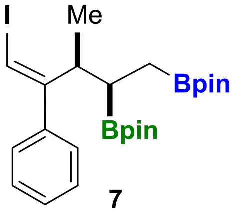

7

^{13}C NMR (100 MHz, CDCl$_3$)

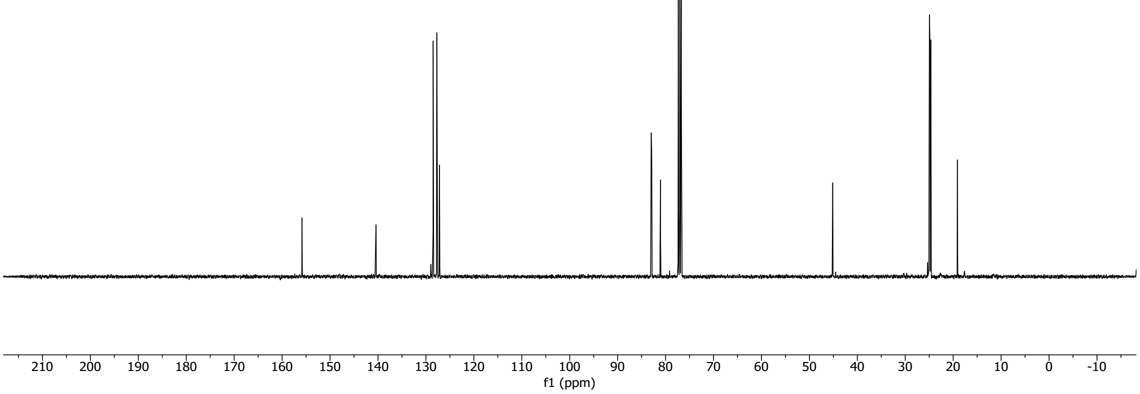

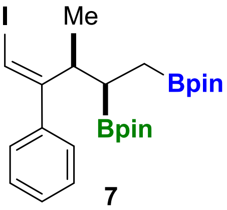

7

^{11}B NMR (129 MHz, CDCl$_3$)

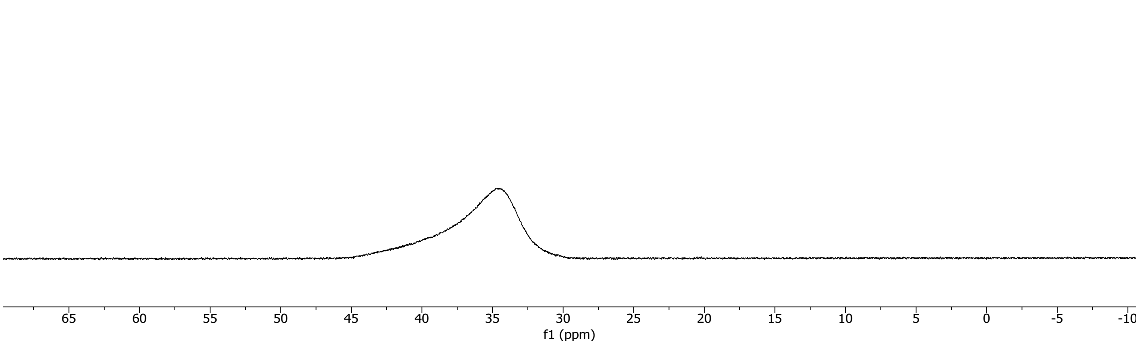

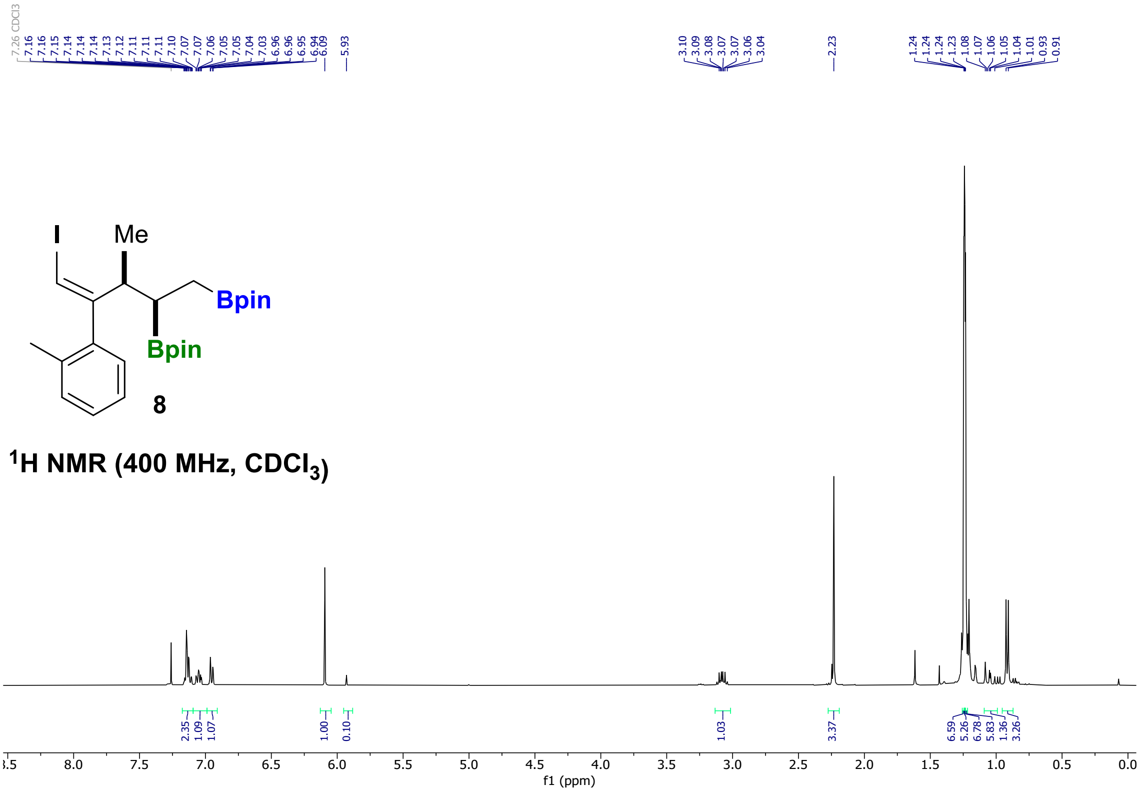
I
Me
Bpin
Bpin
8
^{1}H NMR (400 MHz, CDCl$_3$)
f1 (ppm)

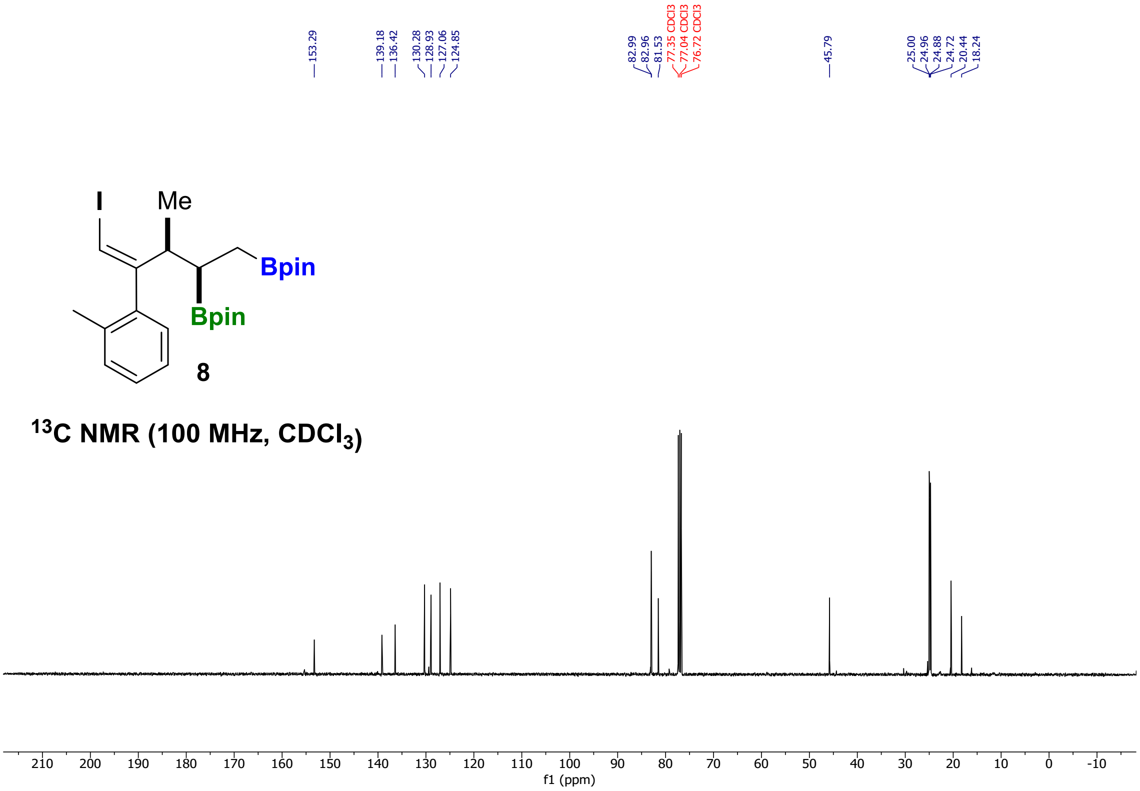
I
Me
Bpin
Bpin
8
^{13}C NMR (100 MHz, CDCl$_3$)
f1 (ppm)

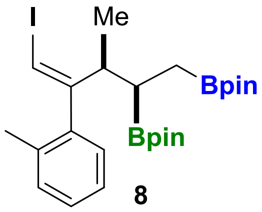

8

^{11}B NMR (129 MHz, $CDCl_3$)

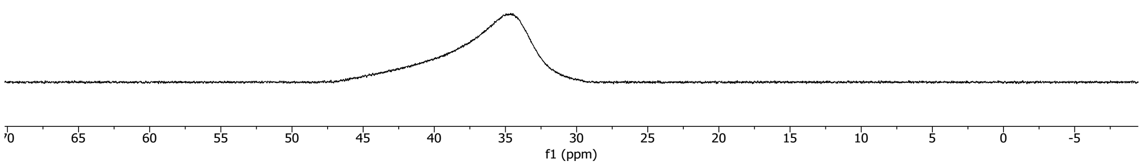

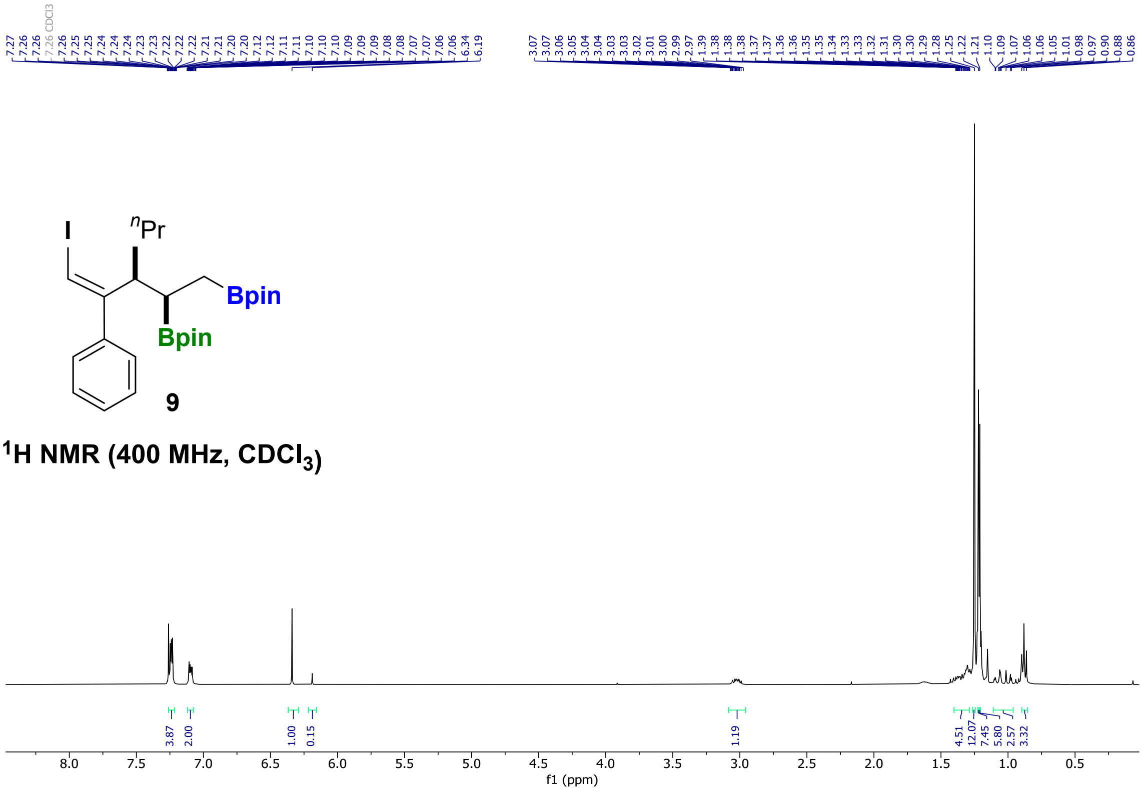

9

^{1}H NMR (400 MHz, $CDCl_3$)

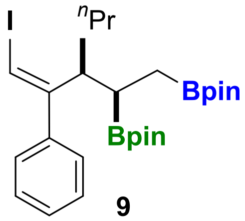

9

^{13}C NMR (100 MHz, $CDCl_3$)

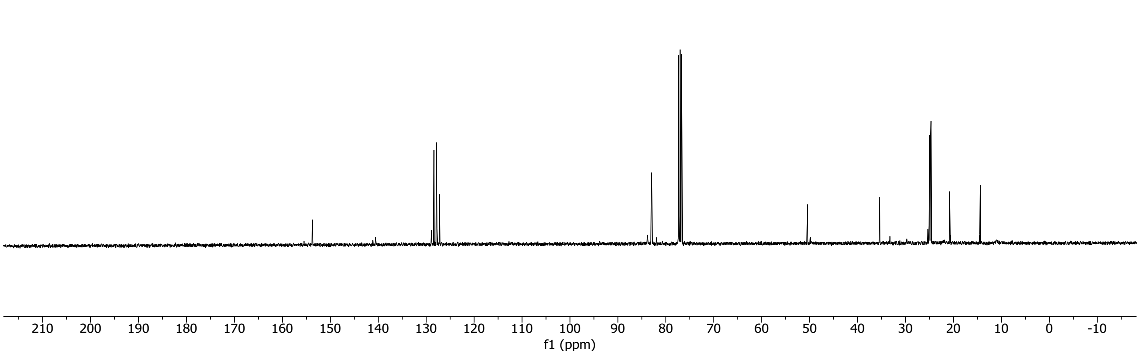

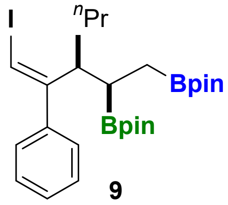

9

^{11}B NMR (129 MHz, $CDCl_3$)

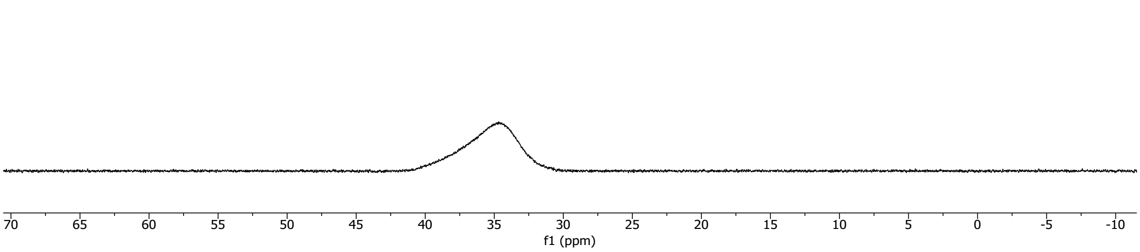

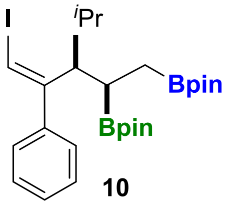

10

^{1}H NMR (400 MHz, $CDCl_3$)

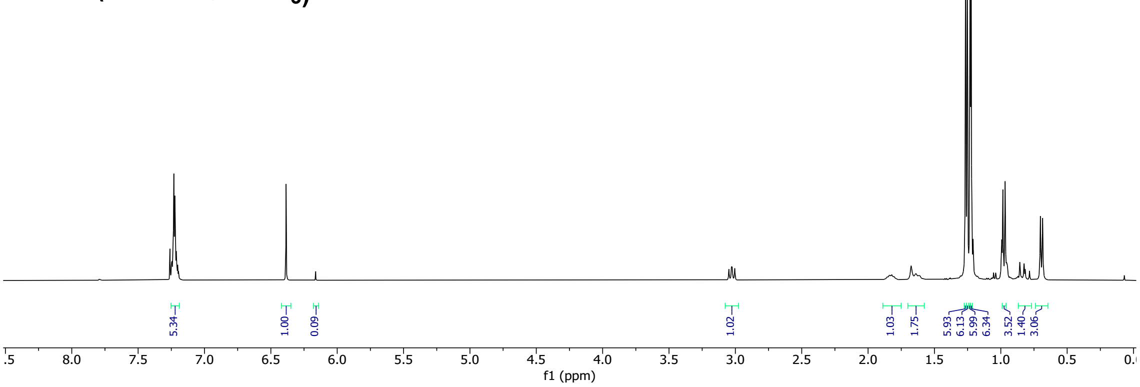

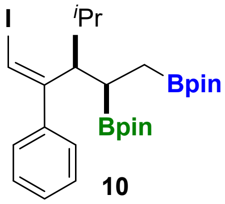

10

^{13}C NMR (100 MHz, $CDCl_3$)

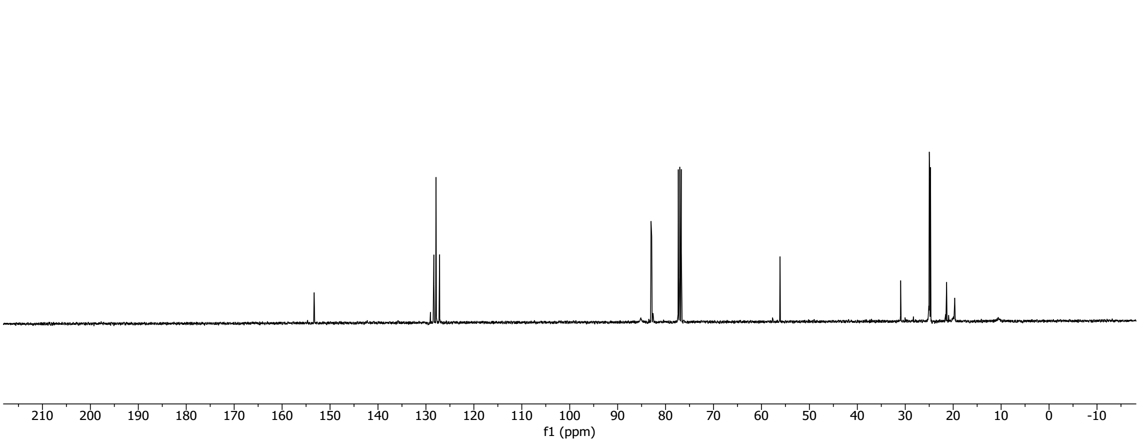

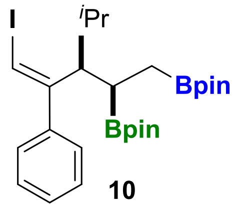

10

^{11}B NMR (129 MHz, CDCl$_3$)

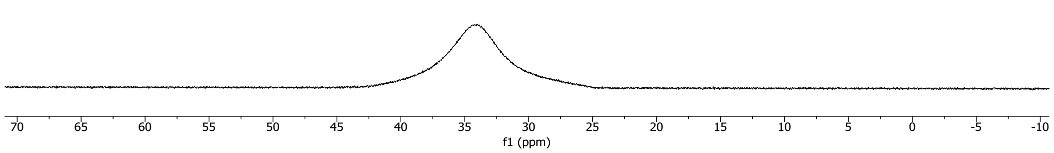

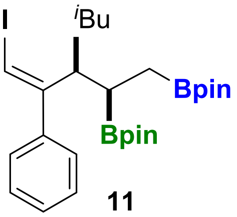

11

^{1}H NMR (400 MHz, CDCl$_3$)

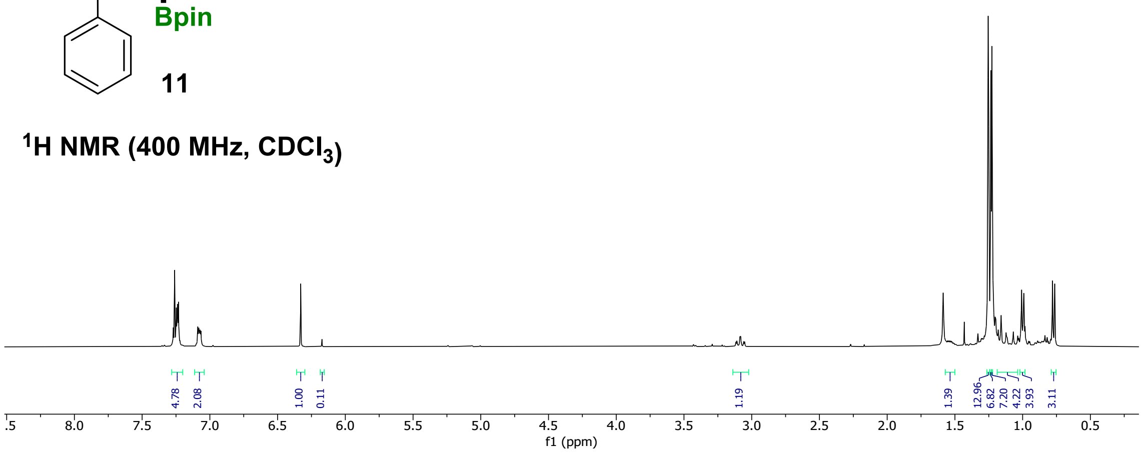

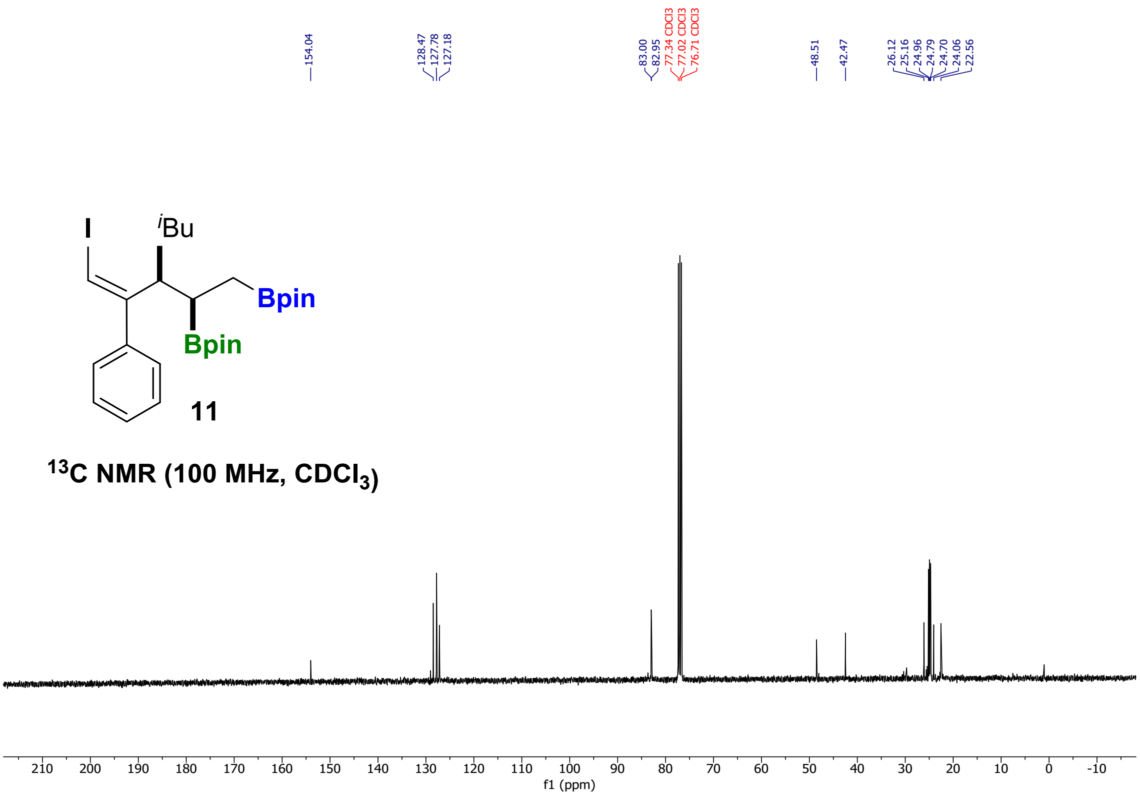
154.04
128.47
127.78
127.18
83.00
82.95
77.34 CDCl3
77.02 CDCl3
76.71 CDCl3
48.51
42.47
26.12
25.16
24.96
24.79
24.70
24.06
22.56
I
iBu
Bpin
Bpin
11
13C NMR (100 MHz, CDCl3)
f1 (ppm)

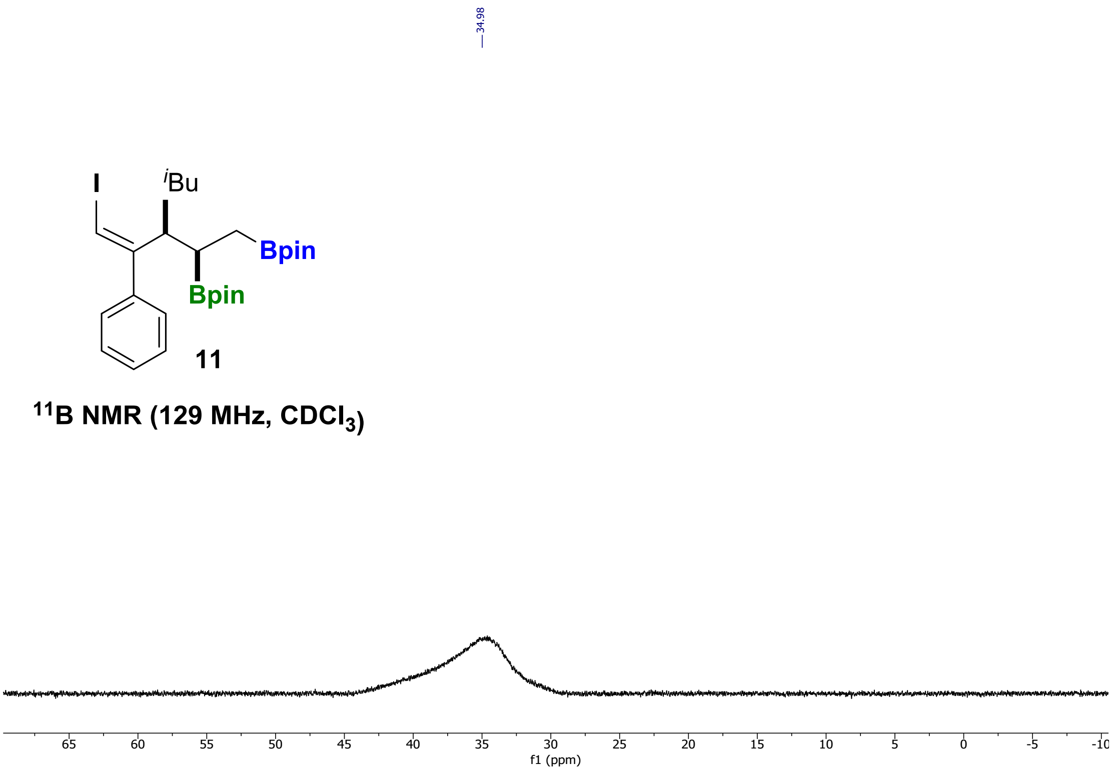
34.98
I
iBu
Bpin
Bpin
11
11B NMR (129 MHz, CDCl3)
f1 (ppm)

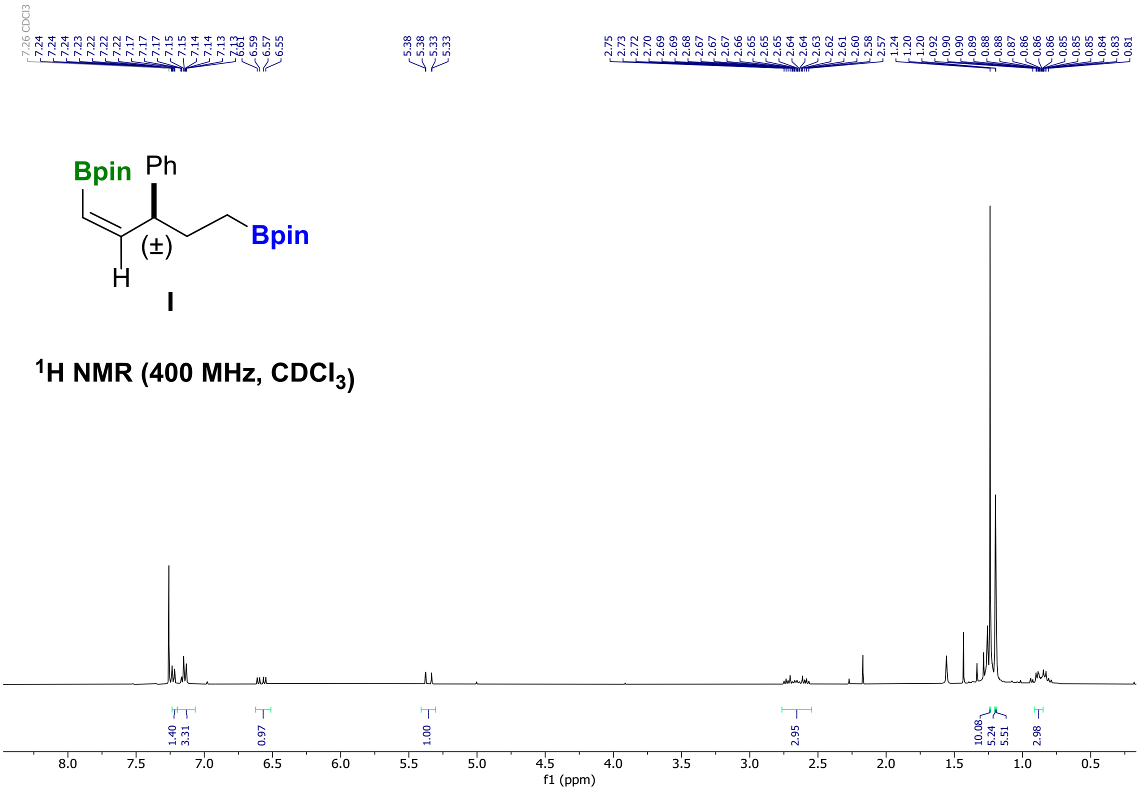
Bpin
Ph
(±)
Bpin
H
I
^{1}H NMR (400 MHz, CDCl$_3$)
f1 (ppm)

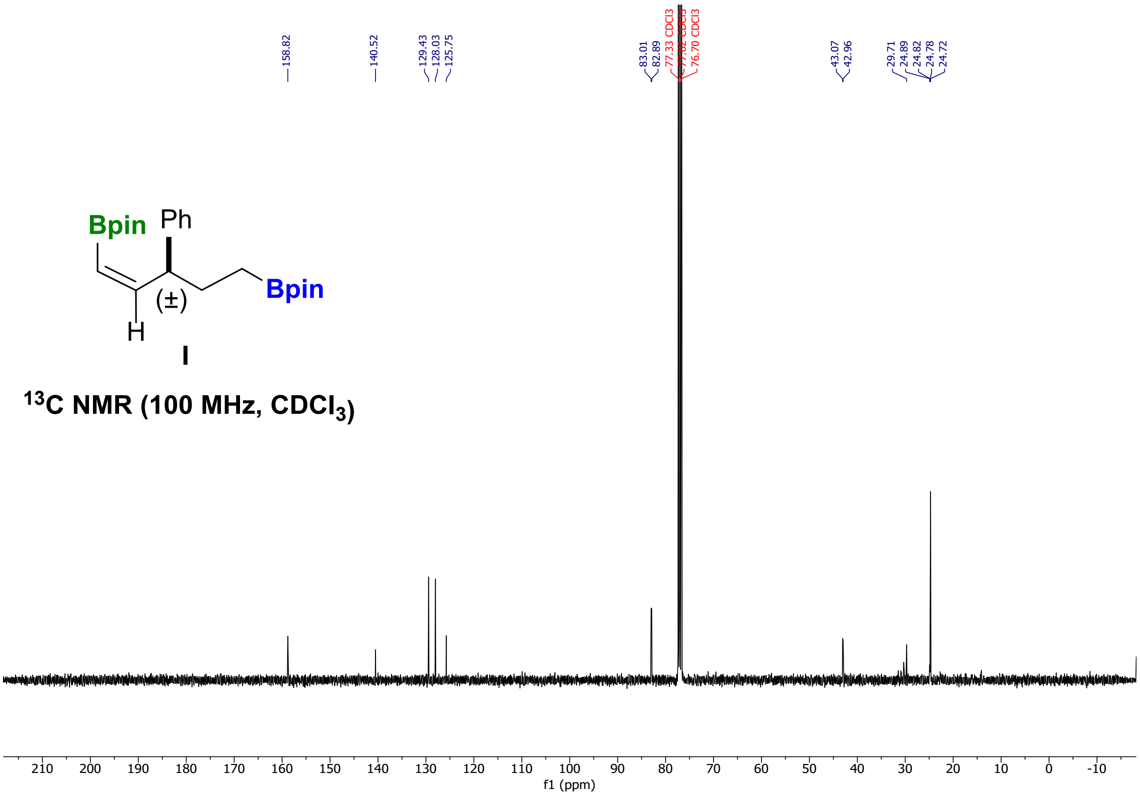
Bpin
Ph
(±)
Bpin
H
I
^{13}C NMR (100 MHz, CDCl$_3$)
f1 (ppm)

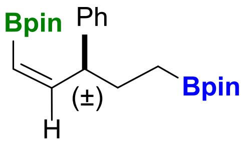

I

^{11}B NMR (129 MHz, $CDCl_3$)

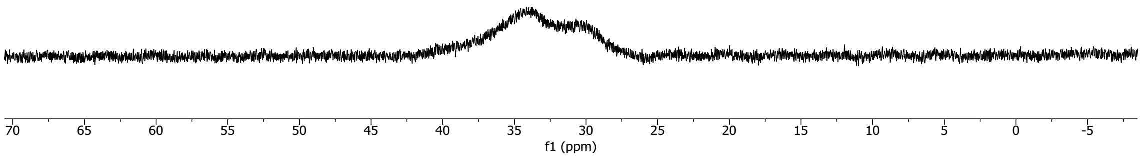

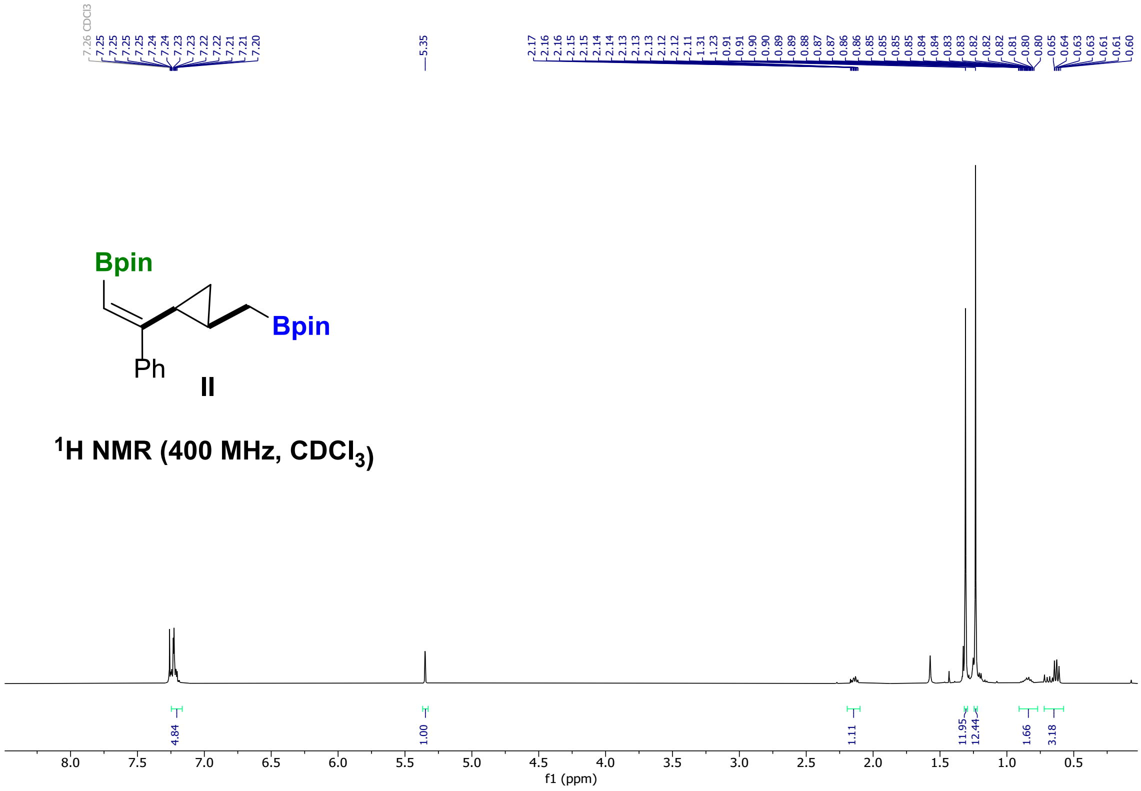

II

^{1}H NMR (400 MHz, $CDCl_3$)

1D-NMR NOE experiment for **II**

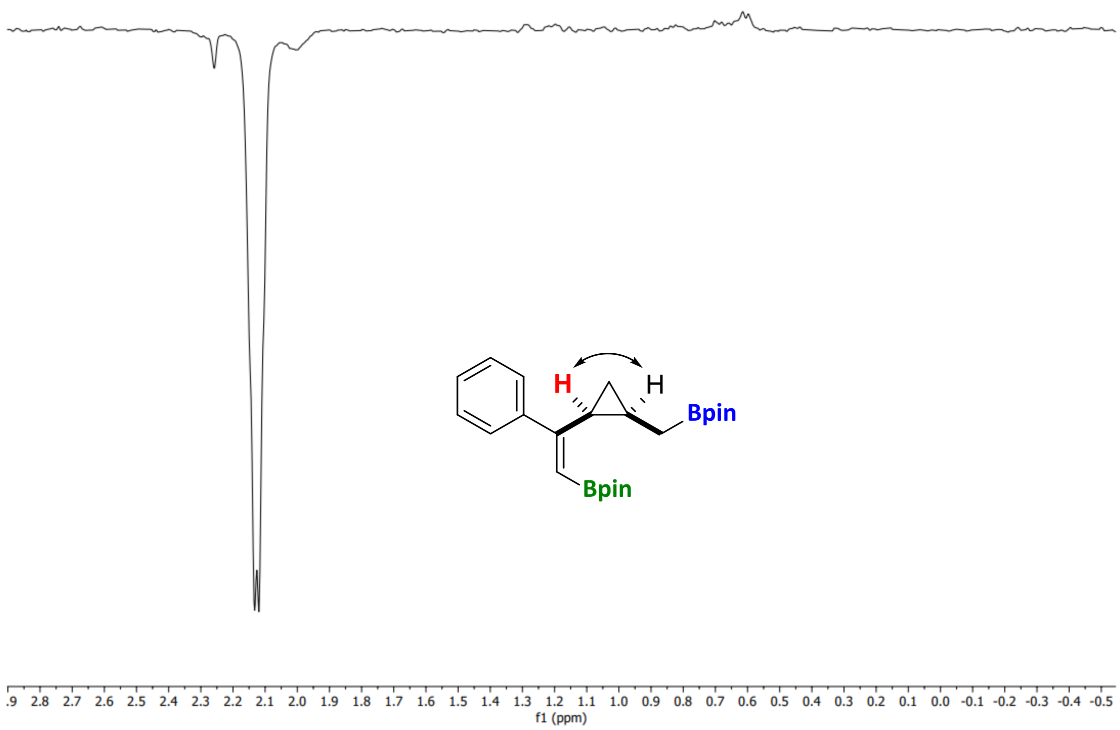

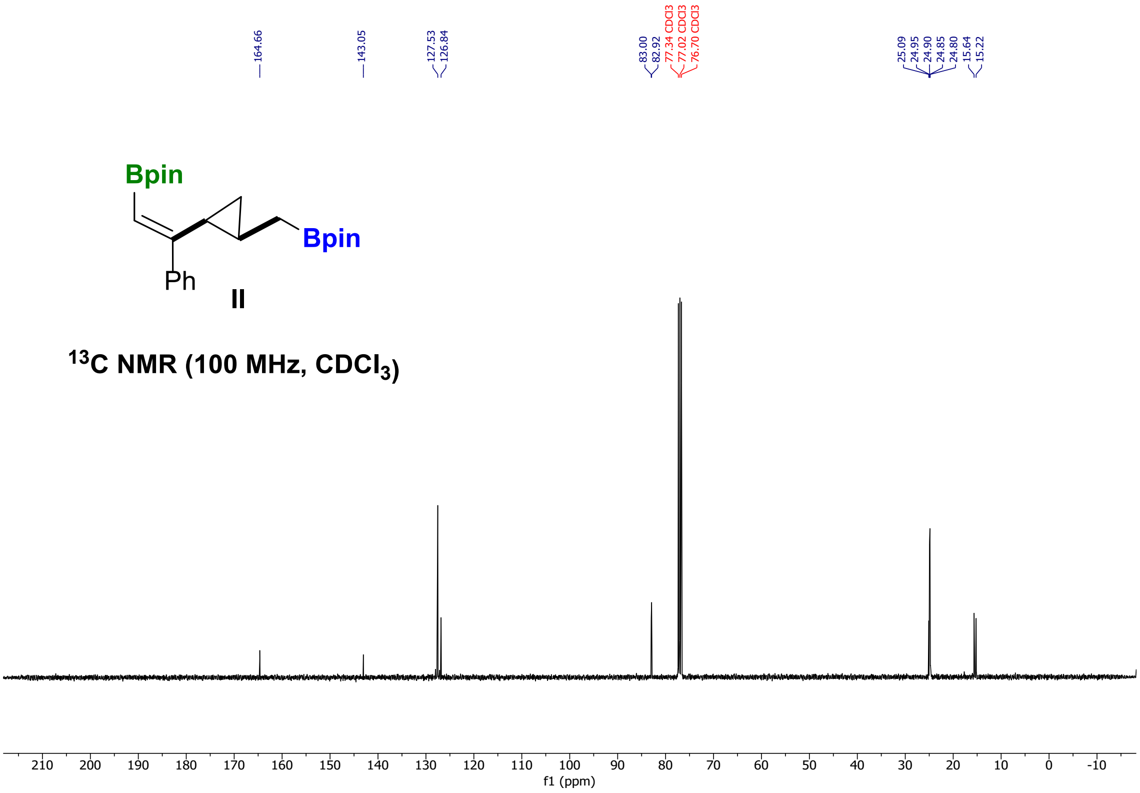

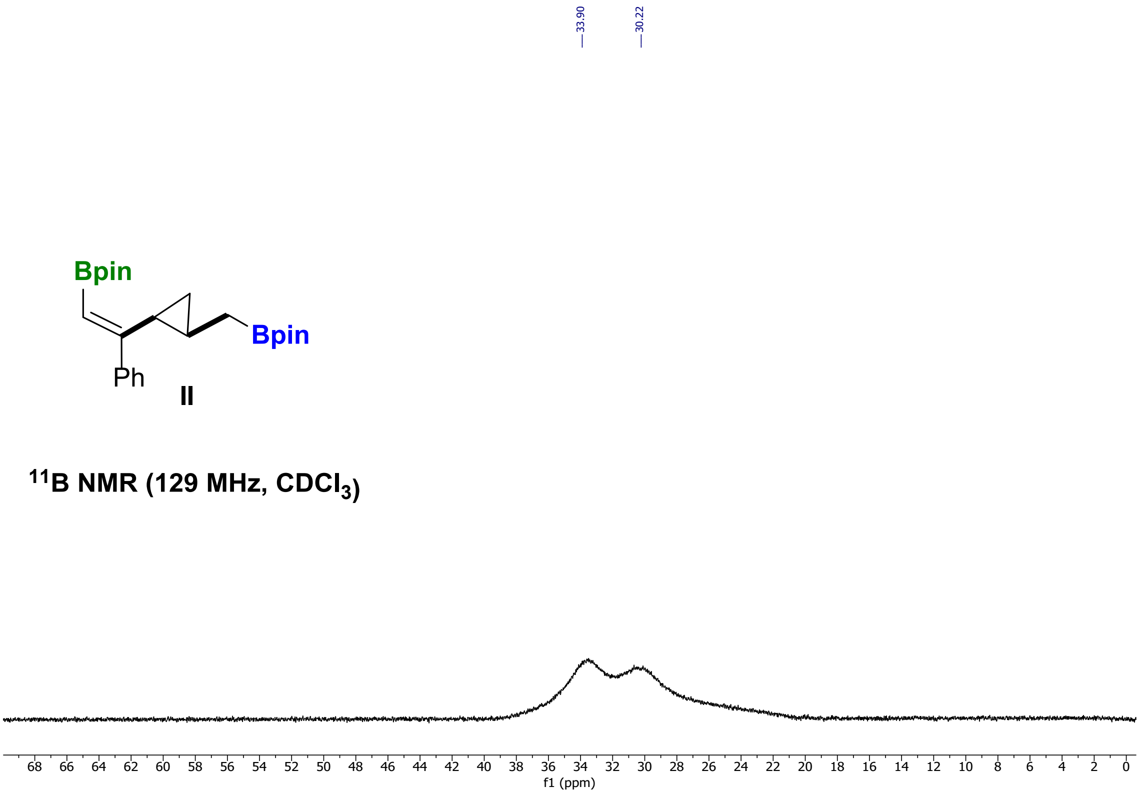

II

^{11}B NMR (129 MHz, $CDCl_3$)

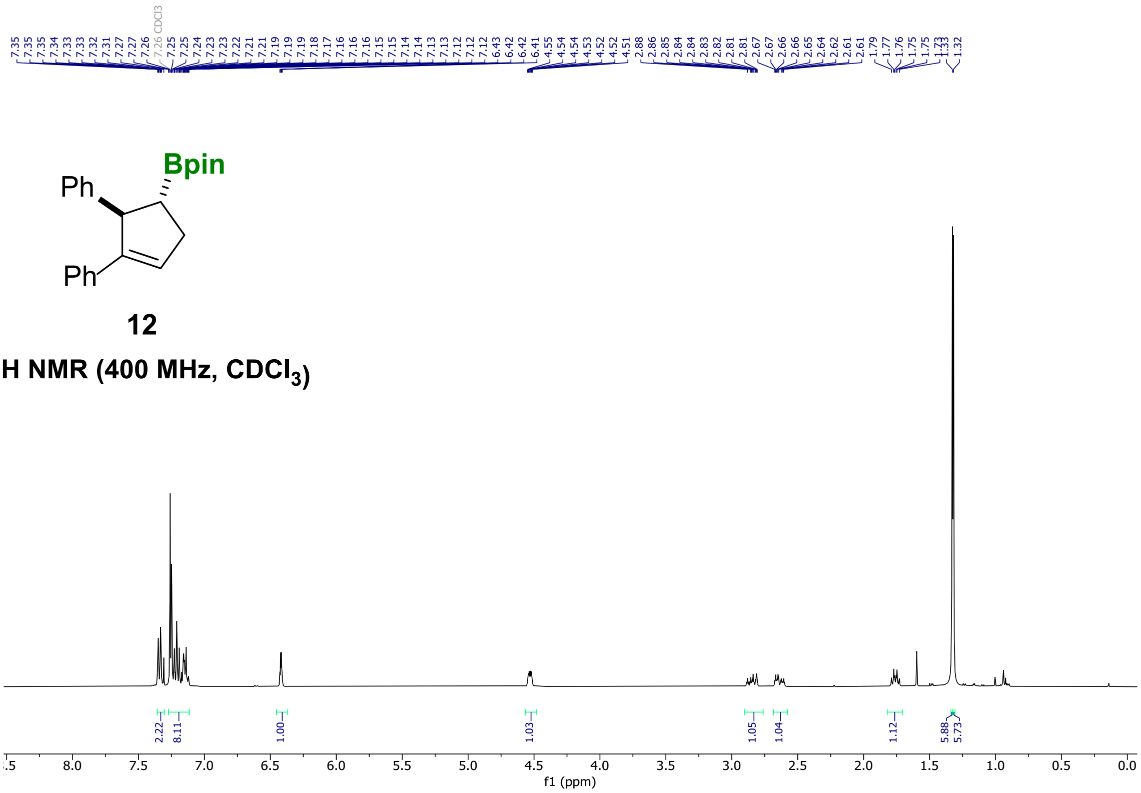

12

^{1}H NMR (400 MHz, $CDCl_3$)

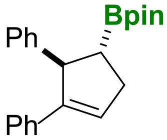

12

^{13}C NMR (100 MHz, CDCl$_3$)

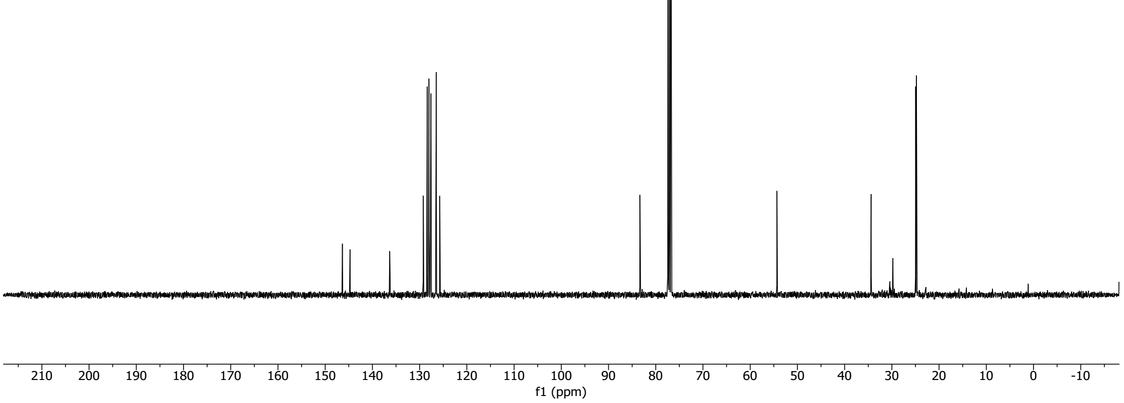

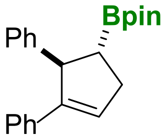

12

^{11}B NMR (129 MHz, CDCl$_3$)

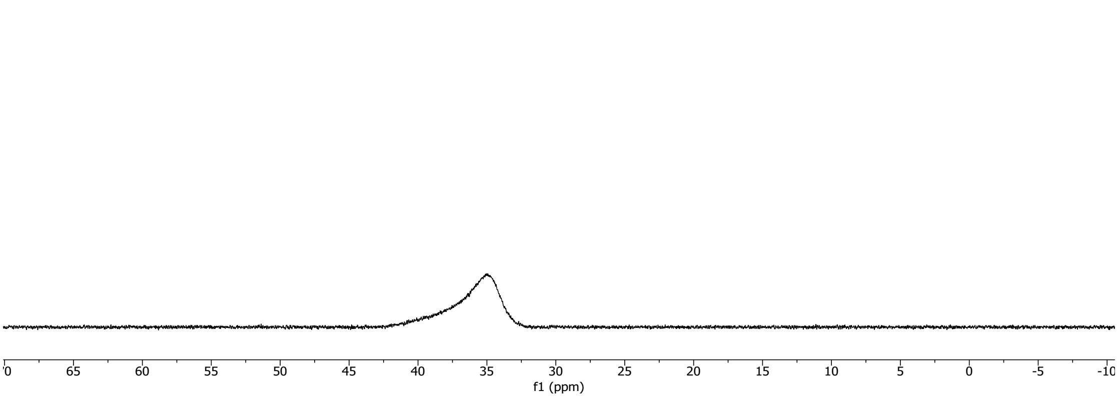

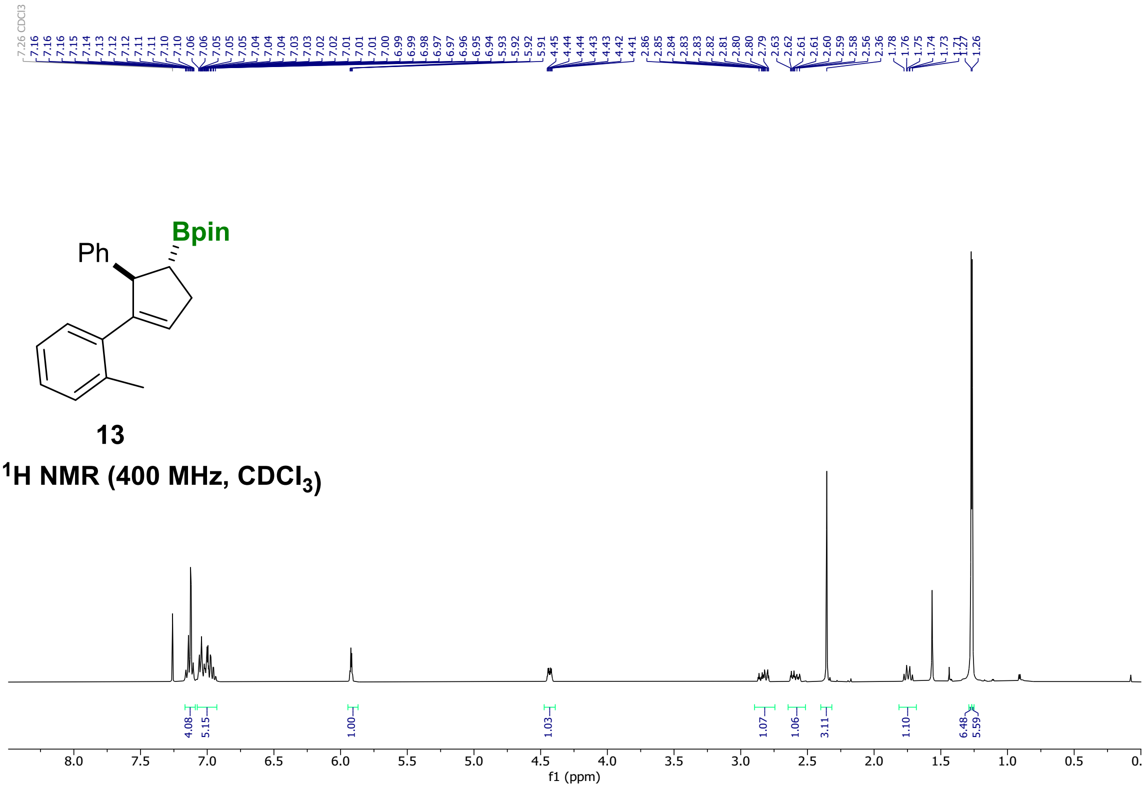
7.26 CDCl3
7.16
7.16
7.16
7.15
7.14
7.13
7.12
7.12
7.11
7.11
7.10
7.10
7.06
7.06
7.05
7.05
7.04
7.04
7.04
7.03
7.03
7.02
7.02
7.01
7.01
7.01
7.00
6.99
6.99
6.98
6.97
6.97
6.96
6.95
6.94
5.93
5.92
5.92
5.91
4.45
4.44
4.44
4.43
4.43
4.42
4.41
2.86
2.85
2.84
2.83
2.83
2.82
2.81
2.80
2.80
2.79
2.63
2.62
2.61
2.61
2.60
2.59
2.58
2.56
2.36
1.78
1.76
1.75
1.74
1.73
1.71
1.27
1.26
Bpin
Ph
13
^{1}H NMR (400 MHz, CDCl$_3$)
4.08
5.15
1.00
1.03
1.07
1.06
3.11
1.10
6.48
5.59
8.0
7.5
7.0
6.5
6.0
5.5
5.0
4.5
4.0
3.5
3.0
2.5
2.0
1.5
1.0
0.5
0.
f1 (ppm)

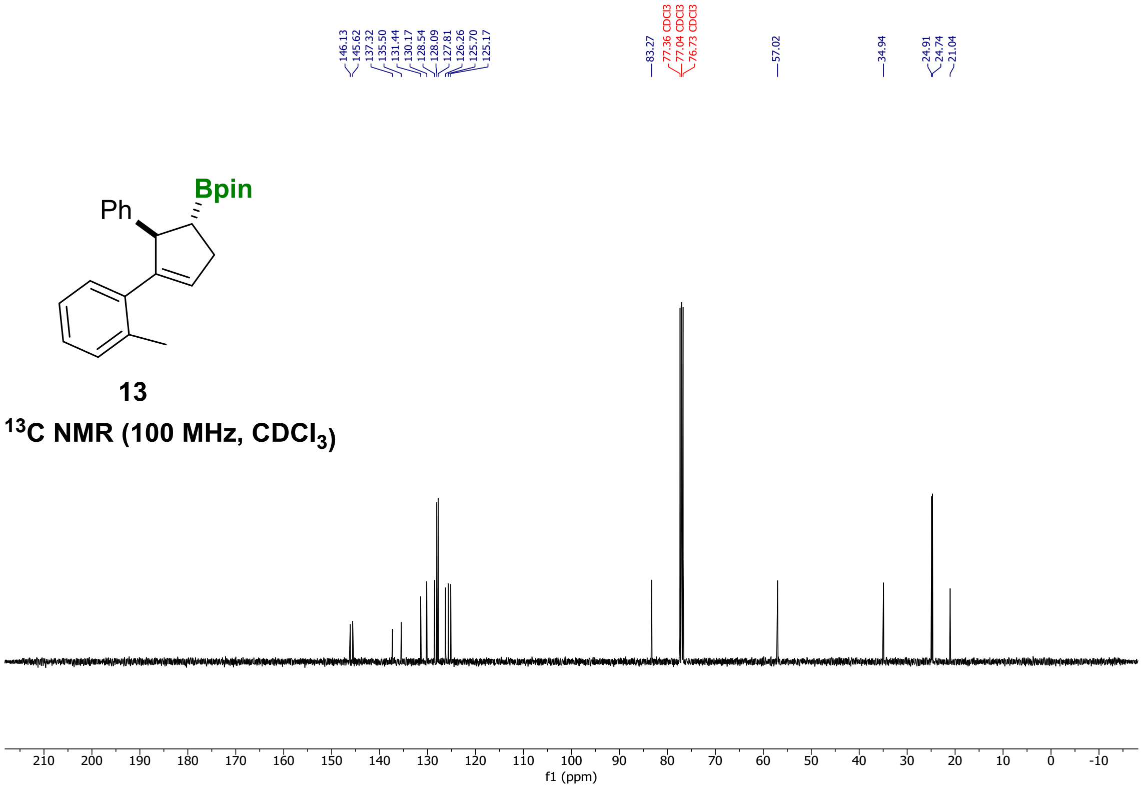
146.13
145.62
137.32
135.50
131.44
130.17
128.54
128.09
127.81
126.26
125.70
125.17
83.27
77.36 CDCl3
77.04 CDCl3
76.73 CDCl3
57.02
34.94
24.91
24.74
21.04
Bpin
Ph
13
^{13}C NMR (100 MHz, CDCl$_3$)
210
200
190
180
170
160
150
140
130
120
110
100
90
80
70
60
50
40
30
20
10
0
-10
f1 (ppm)

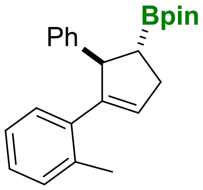

13

^{11}B NMR (129 MHz, CDCl$_3$)

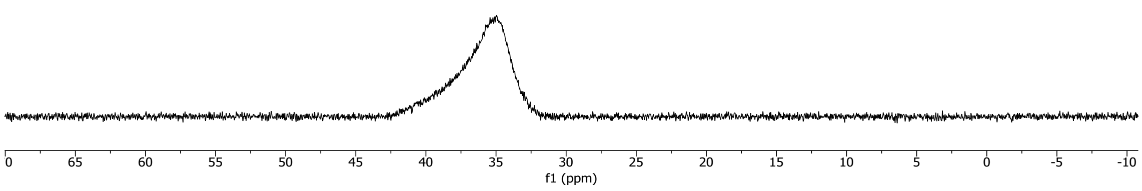

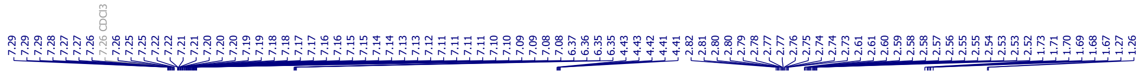

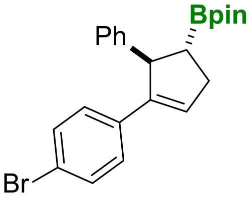

14

^{1}H NMR (400 MHz, CDCl$_3$)

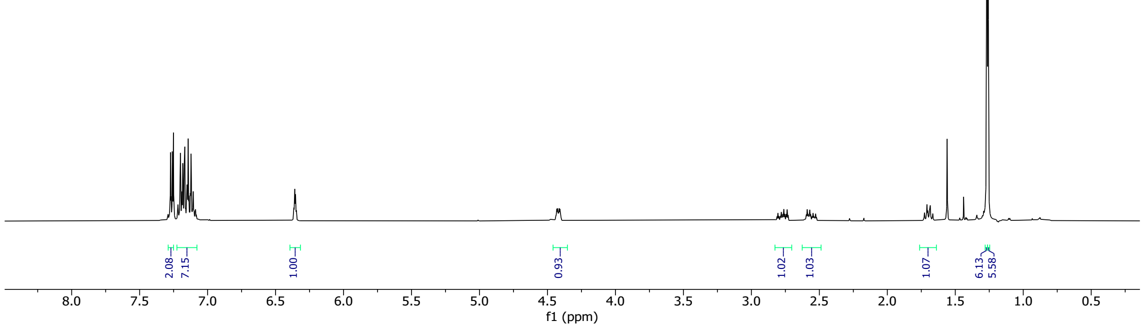

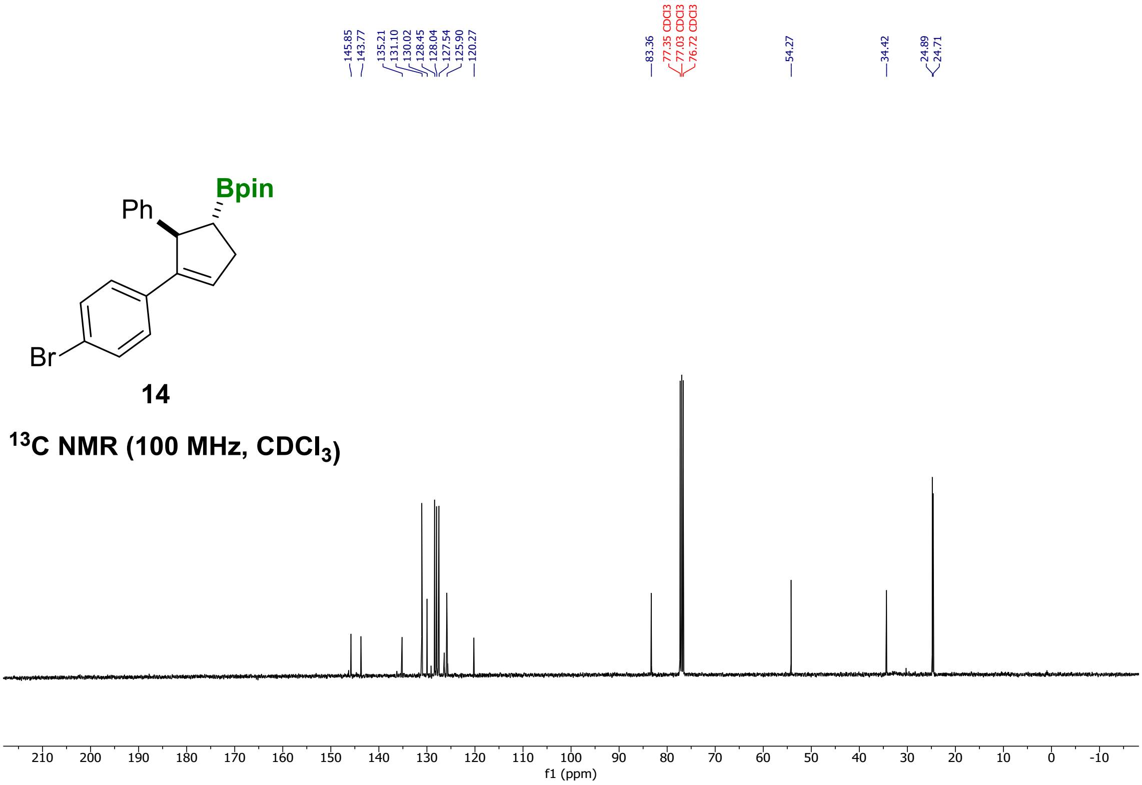

14

^{13}C NMR (100 MHz, $CDCl_3$)

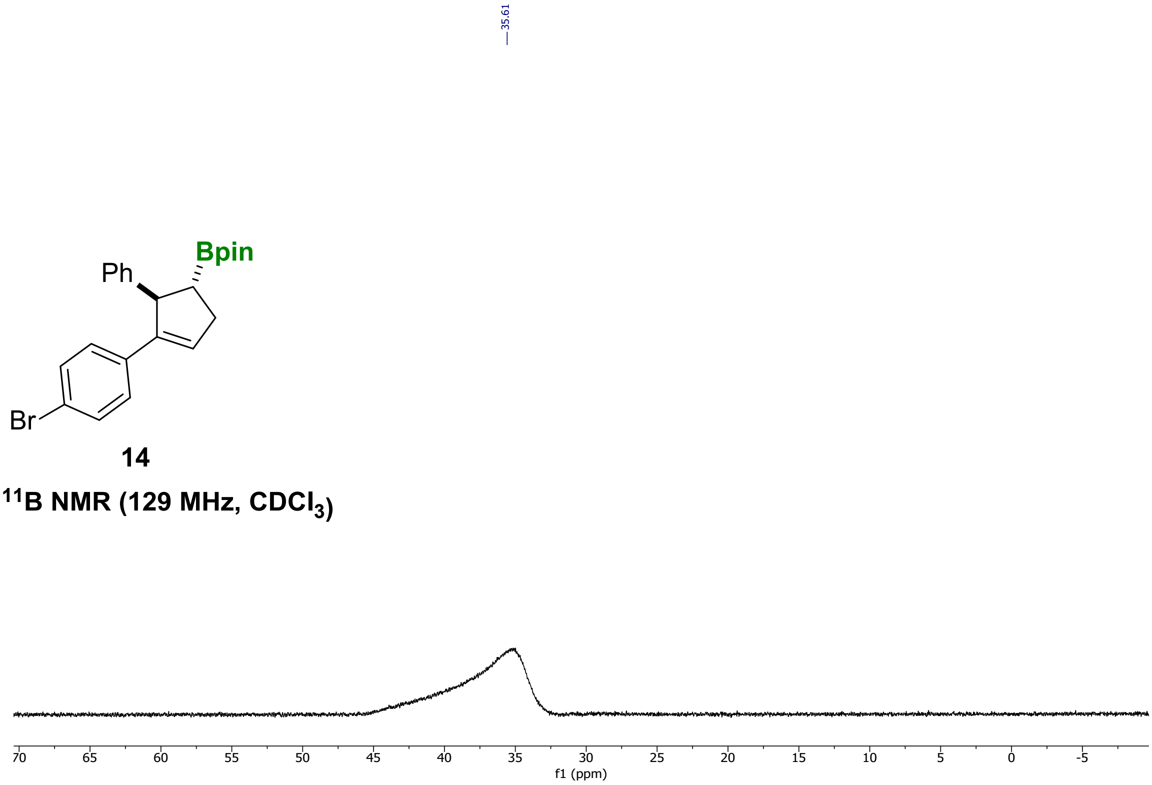

14

^{11}B NMR (129 MHz, $CDCl_3$)

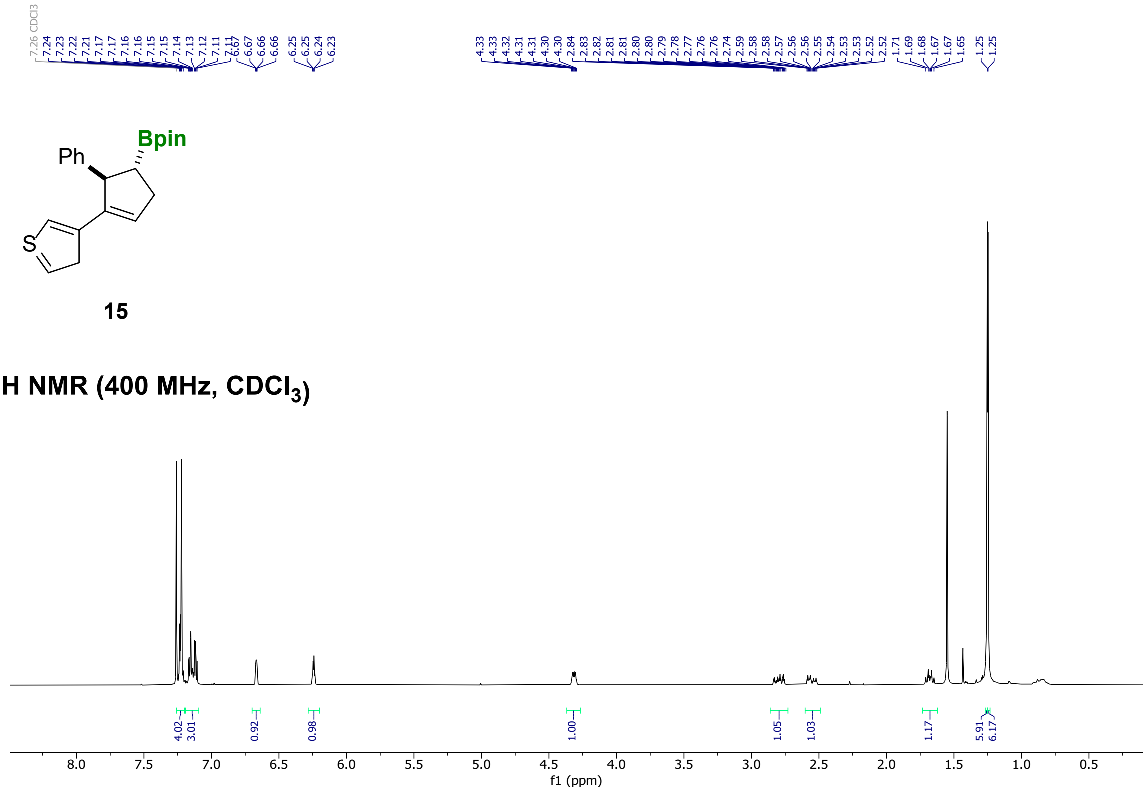

15

^{1}H NMR (400 MHz, CDCl$_3$)

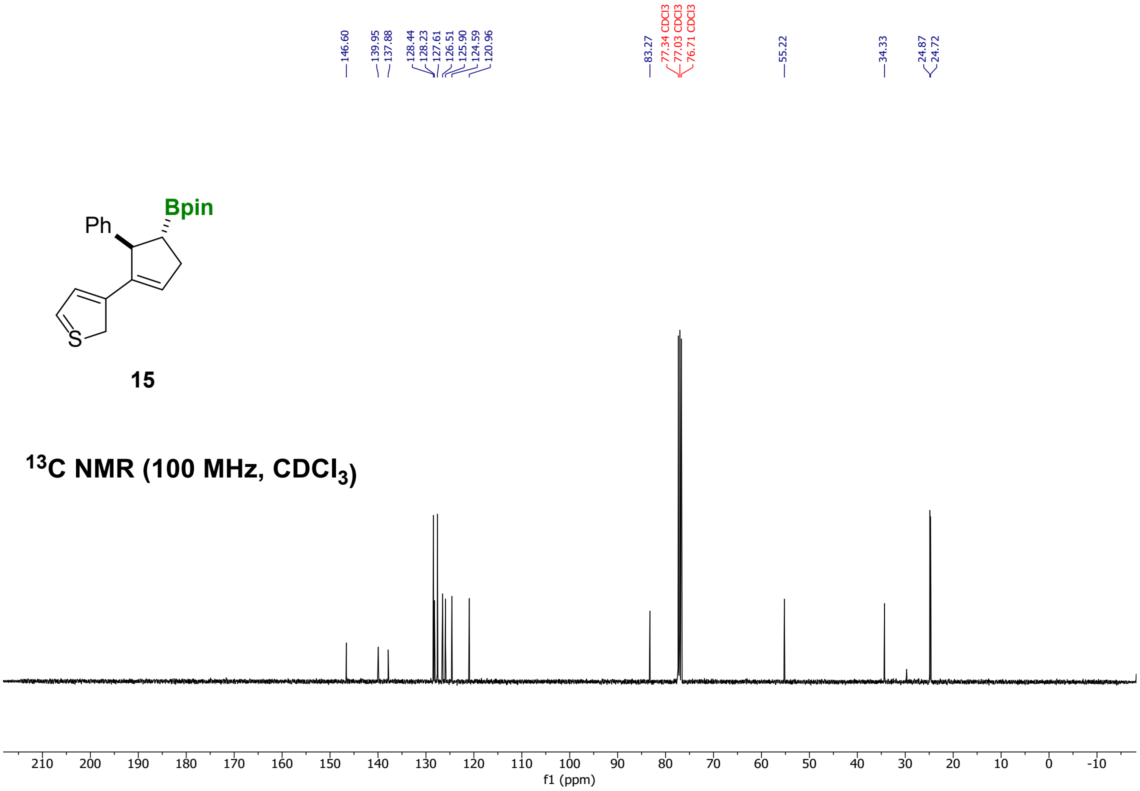

15

^{13}C NMR (100 MHz, CDCl$_3$)

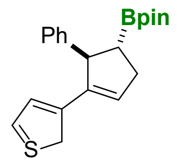

15

^{11}B NMR (129 MHz, $CDCl_3$)

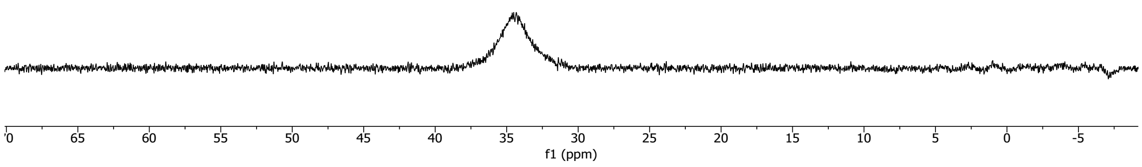

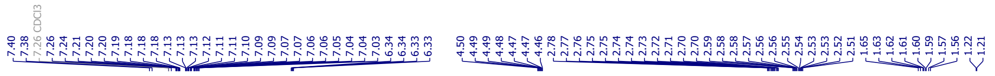

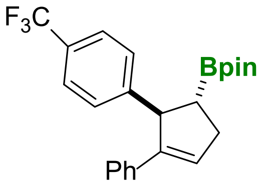

16

^{1}H NMR (400 MHz, $CDCl_3$)

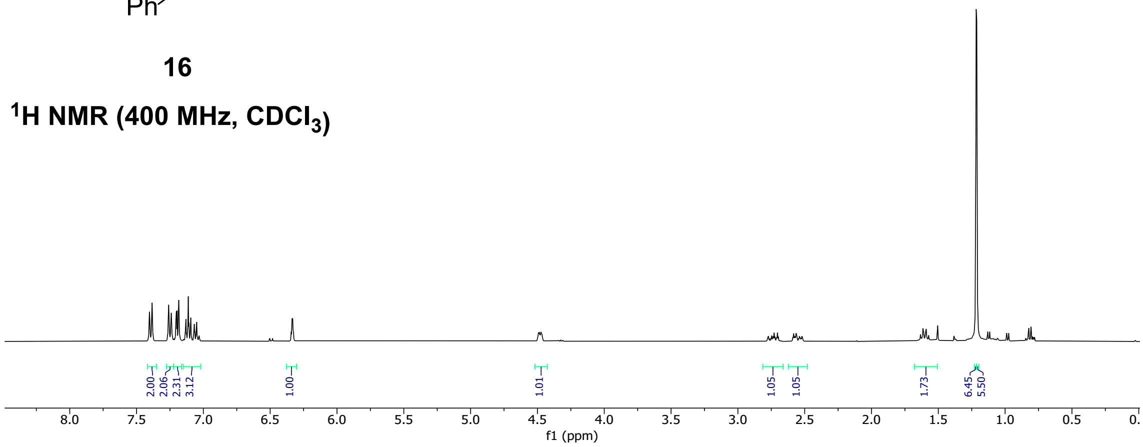

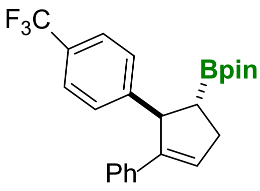

16

^{13}C NMR (100 MHz, CDCl$_3$)

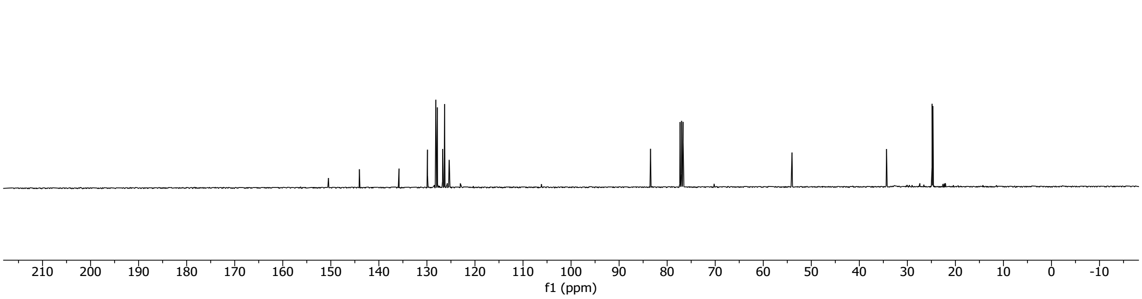

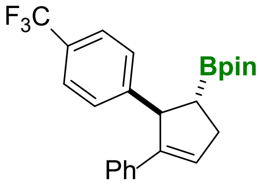

16

^{11}B NMR (129 MHz, CDCl$_3$)

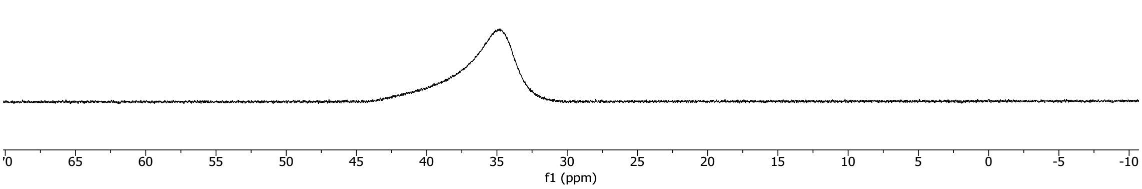

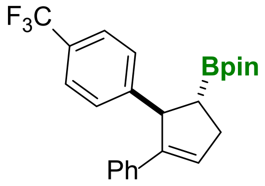

16

^{19}F NMR (377 MHz, $CDCl_3$)

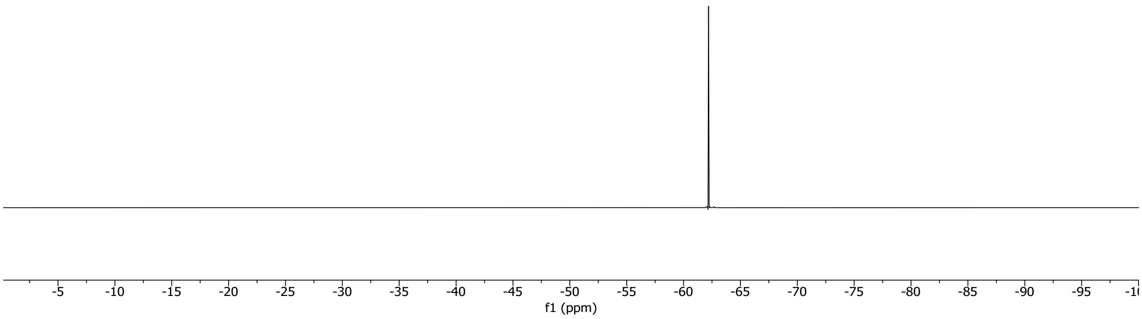

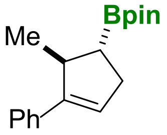

17

^{11}B NMR (129 MHz, $CDCl_3$)

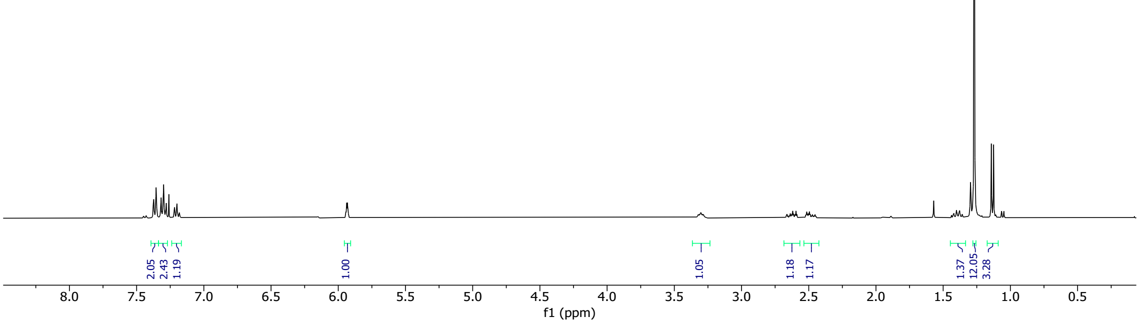

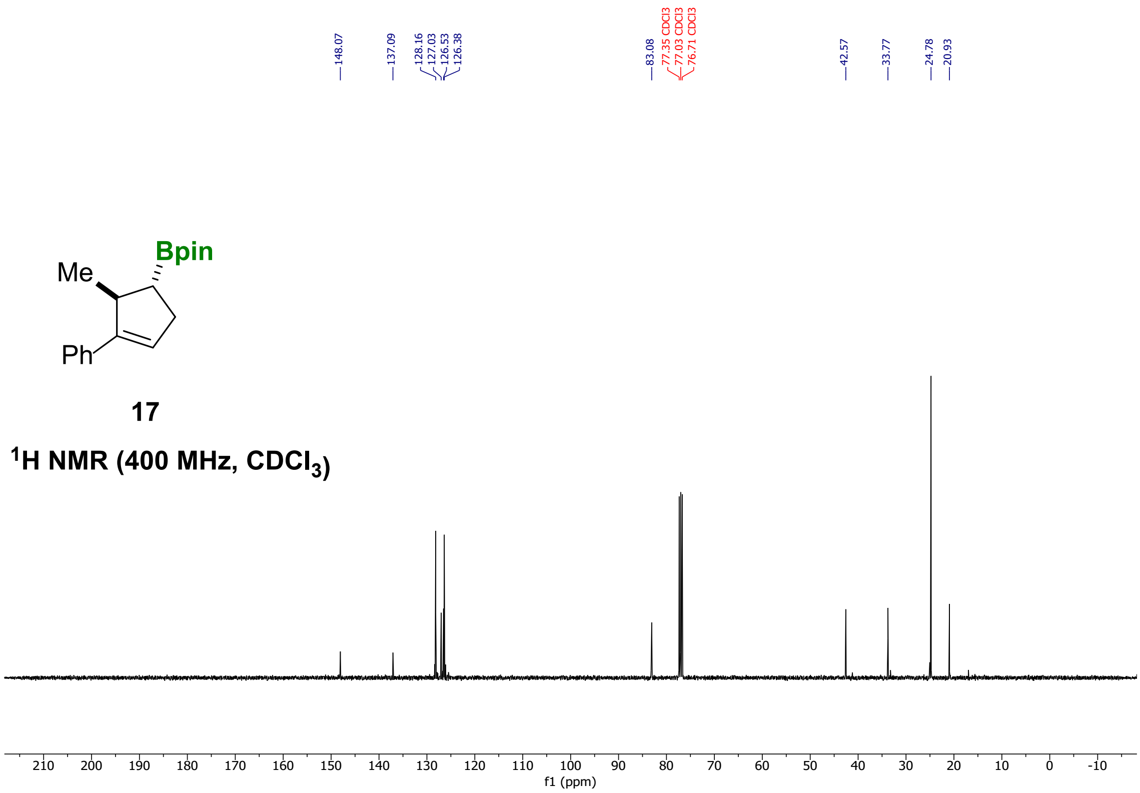

17

^{1}H NMR (400 MHz, $CDCl_3$)

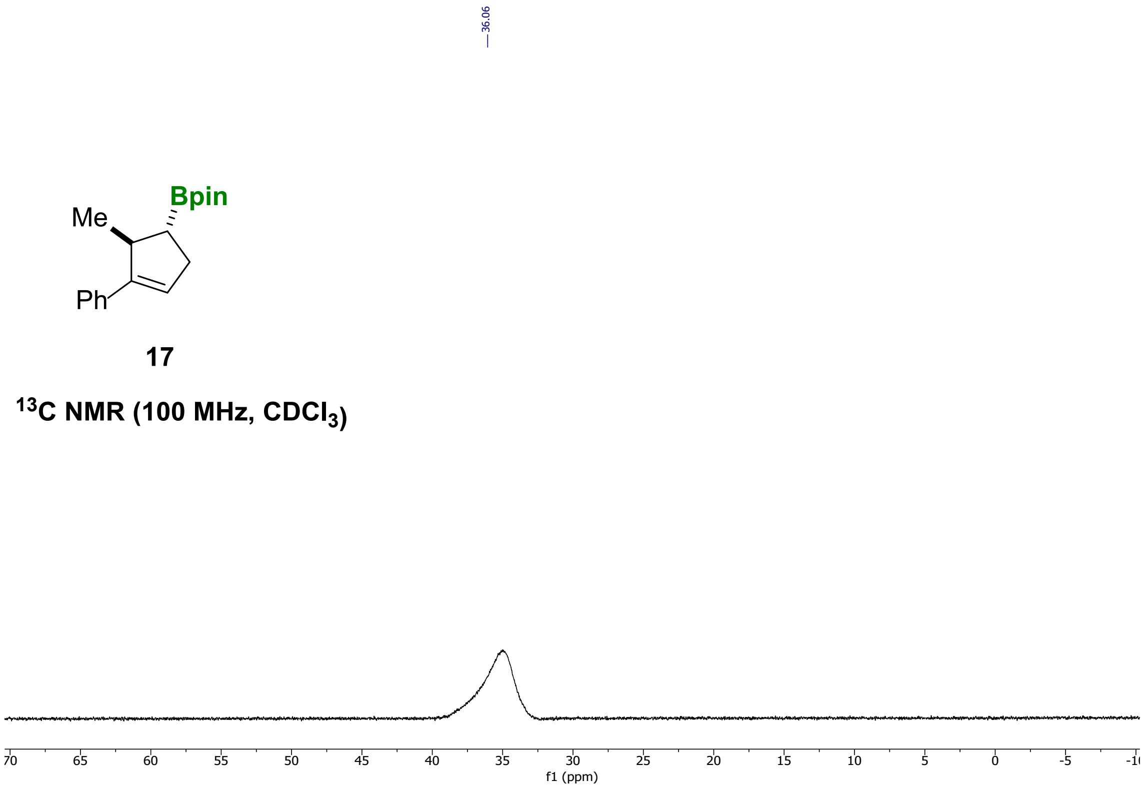

17

^{13}C NMR (100 MHz, $CDCl_3$)

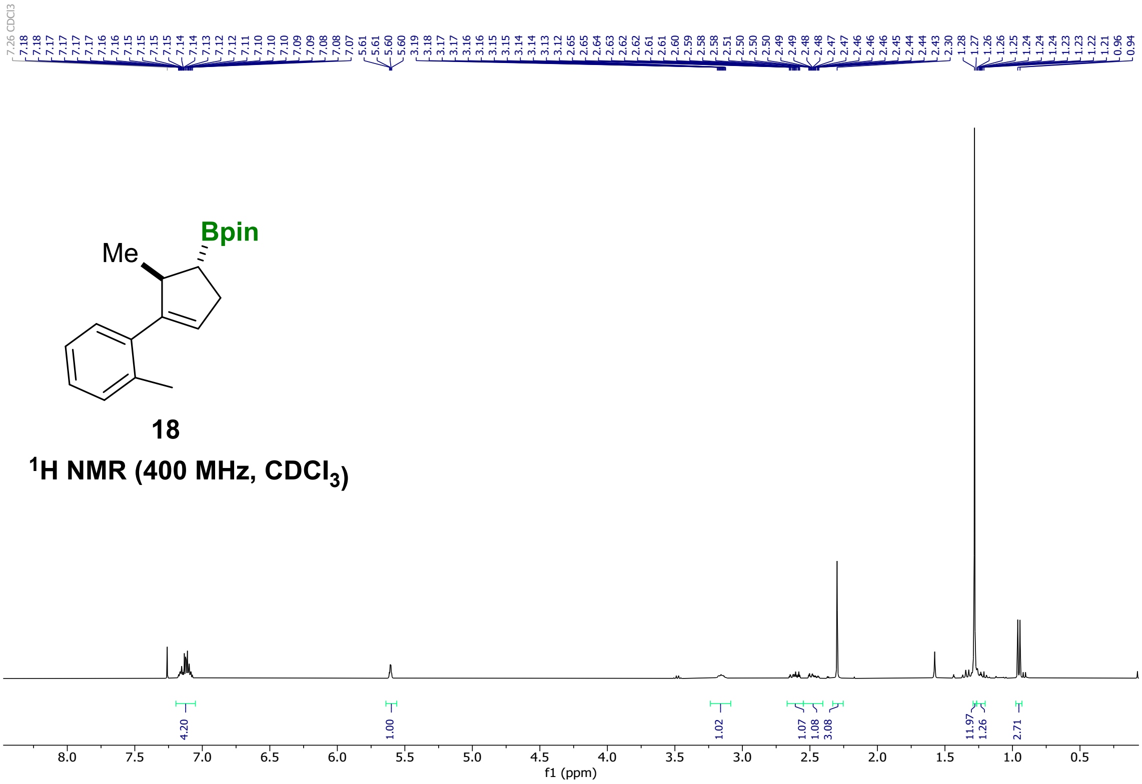

18

^{1}H NMR (400 MHz, $CDCl_3$)

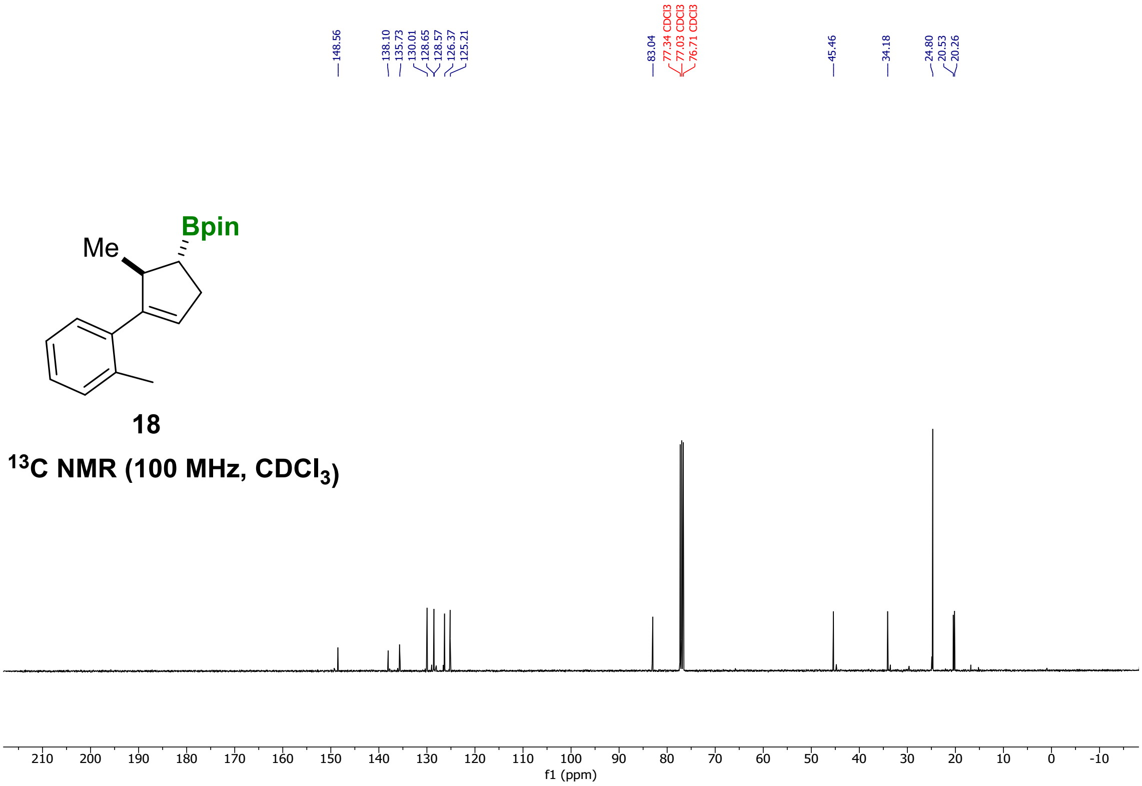

18

^{13}C NMR (100 MHz, $CDCl_3$)

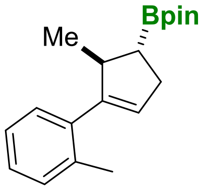

18

^{11}B NMR (129 MHz, $CDCl_3$)

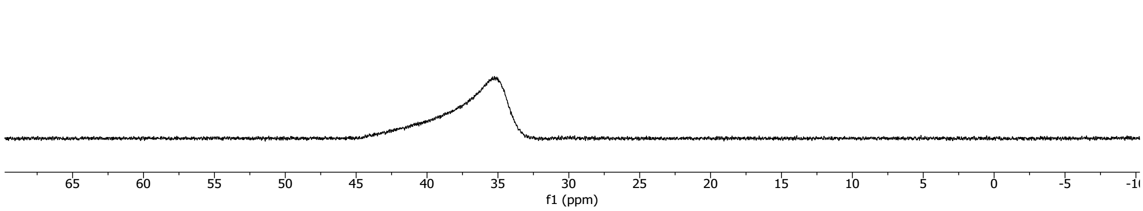

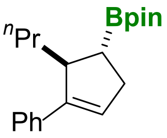

19

^{1}H NMR (400 MHz, $CDCl_3$)

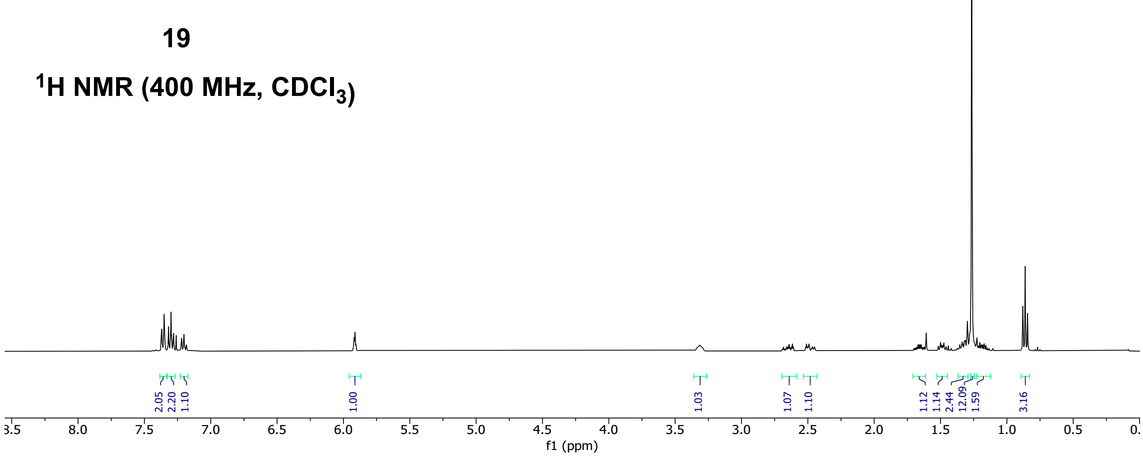

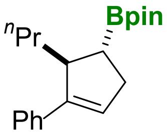

19

^{13}C NMR (100 MHz, $CDCl_3$)

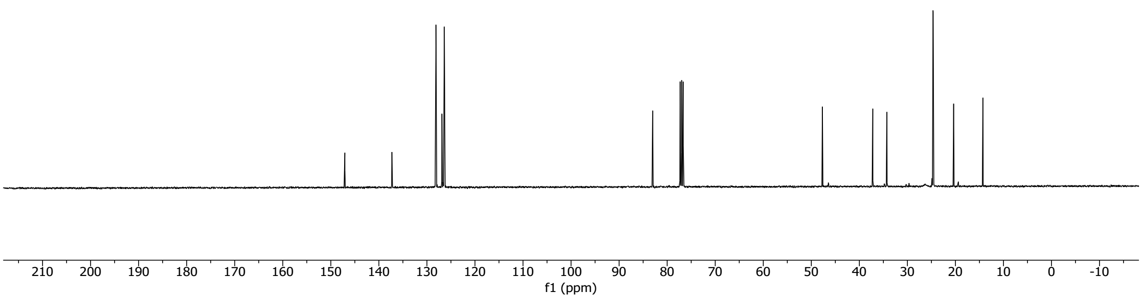

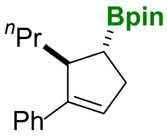

19

^{11}B NMR (129 MHz, $CDCl_3$)

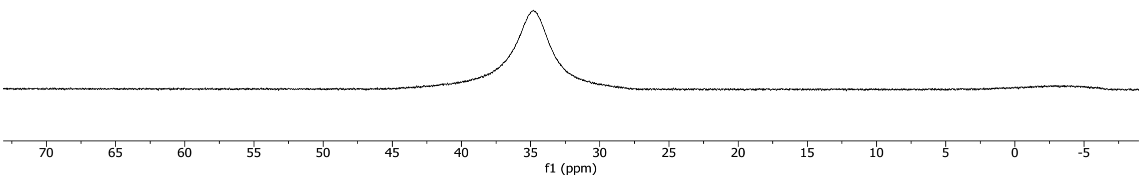

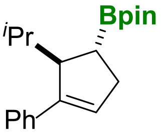

20

^{1}H NMR (400 MHz, CDCl$_3$)

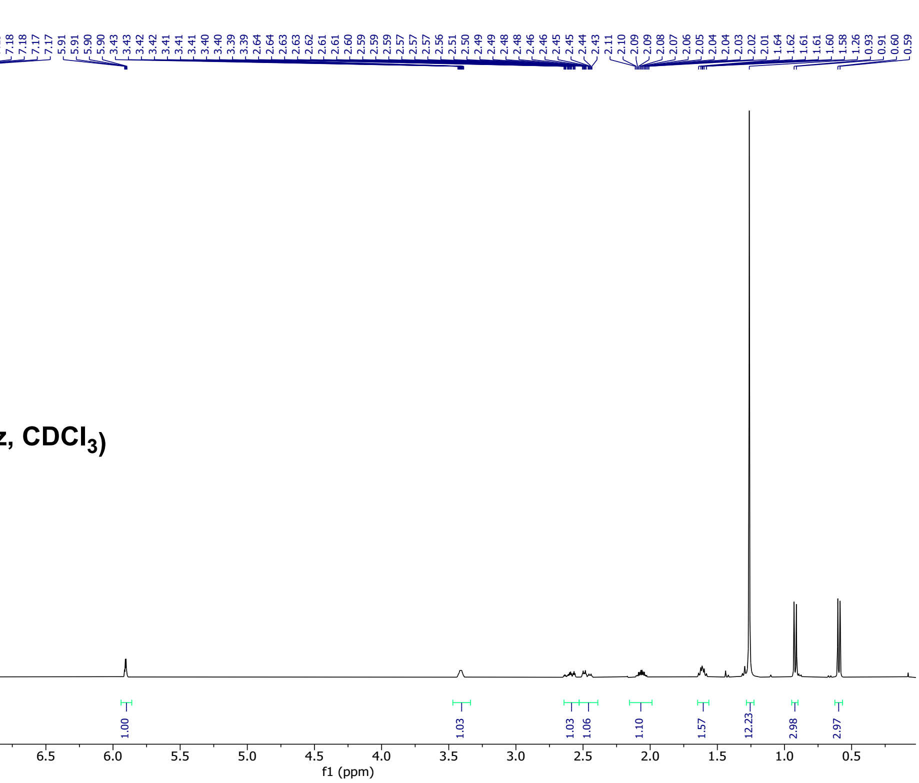

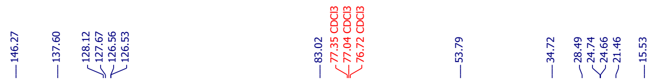

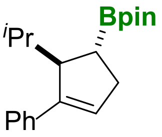

20

^{13}C NMR (100 MHz, CDCl$_3$)

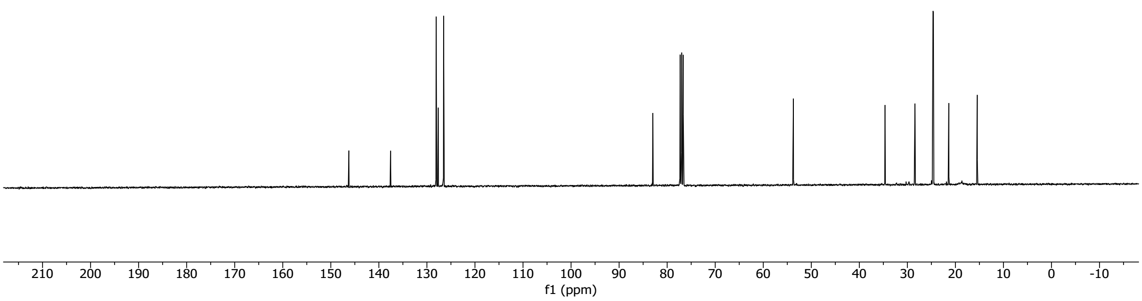

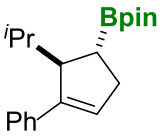

20

^{11}B NMR (129 MHz, CDCl$_3$)

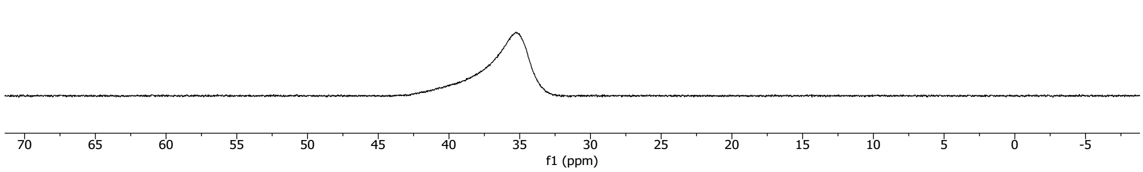

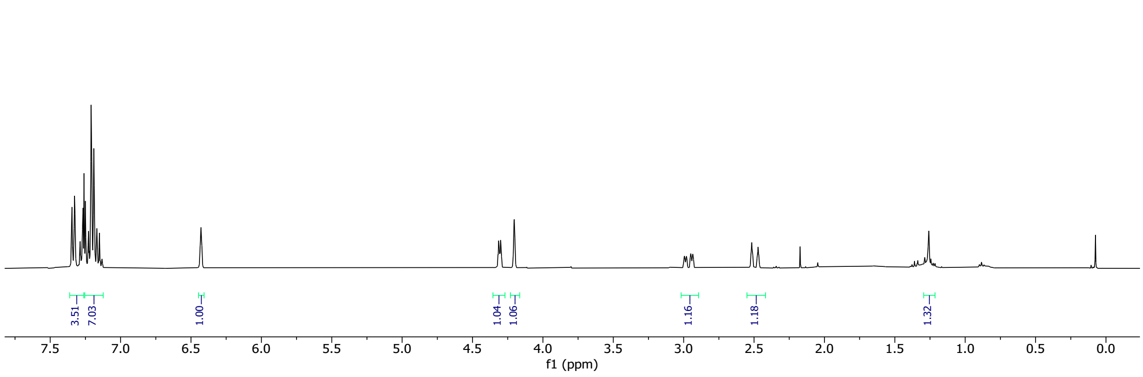

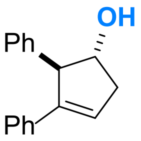

21

^{1}H NMR (400 MHz, CDCl$_3$)

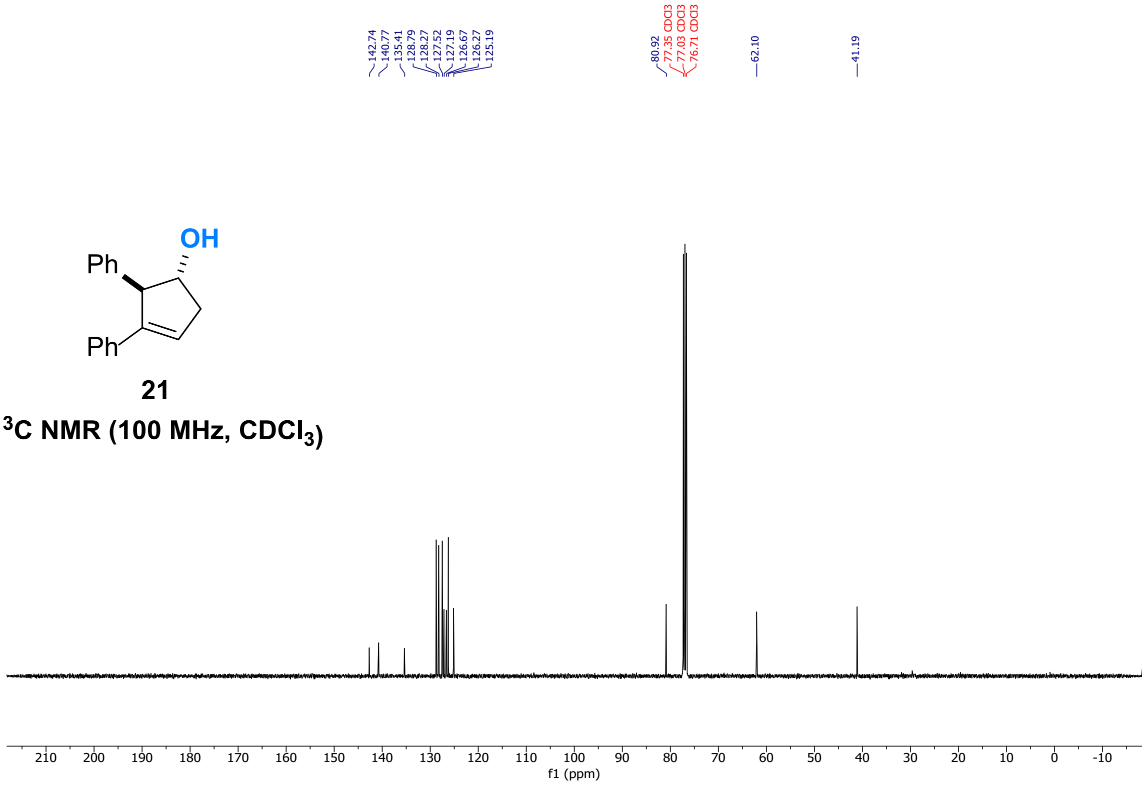

21

^{13}C NMR (100 MHz, $CDCl_3$)

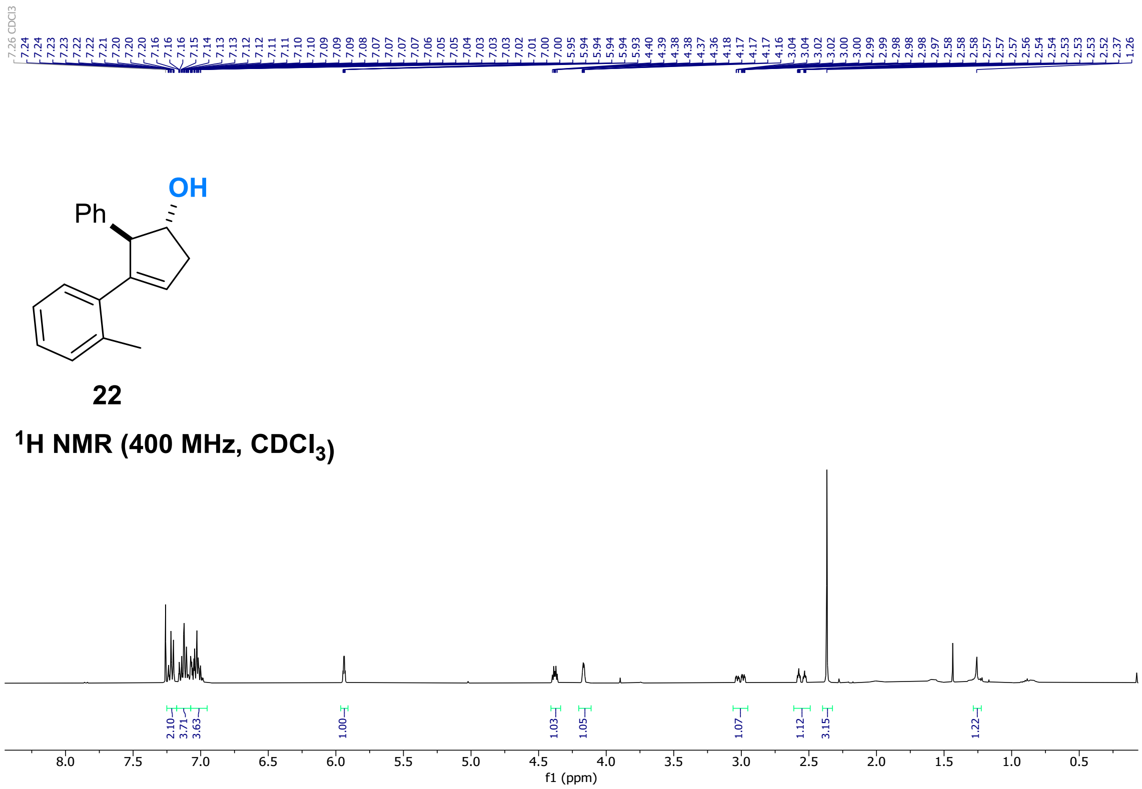

22

^{1}H NMR (400 MHz, $CDCl_3$)

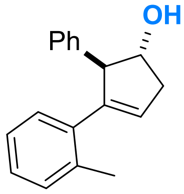

22

^{13}C NMR (100 MHz, CDCl$_3$)

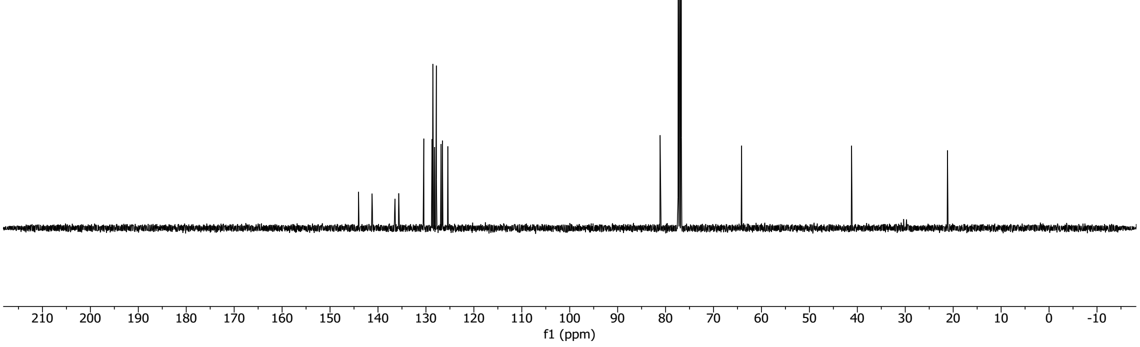

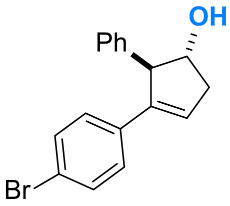

23

^{1}H NMR (400 MHz, CDCl$_3$)

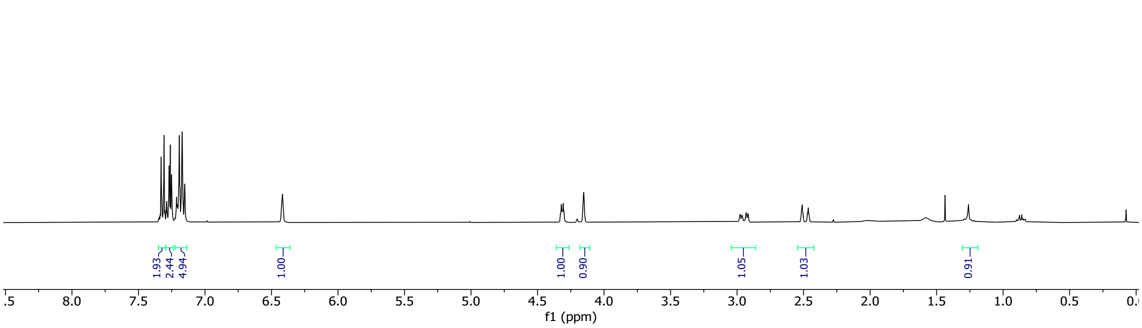

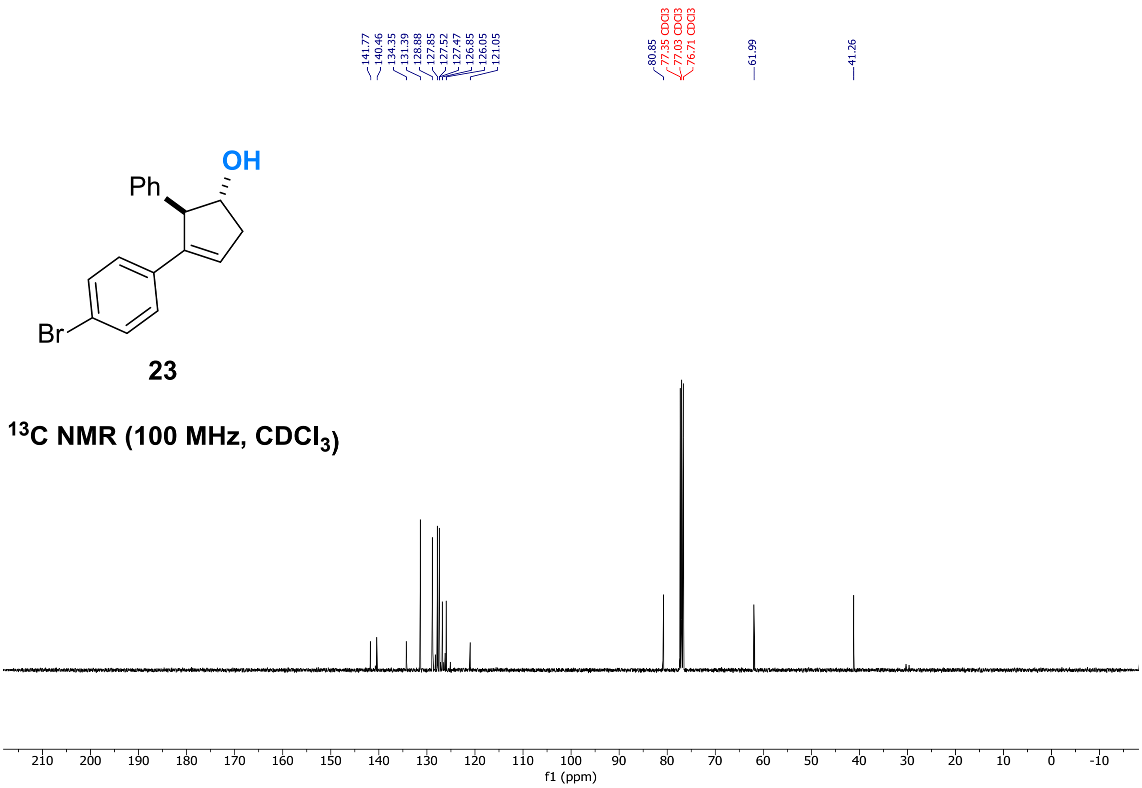

23

^{13}C NMR (100 MHz, CDCl$_3$)

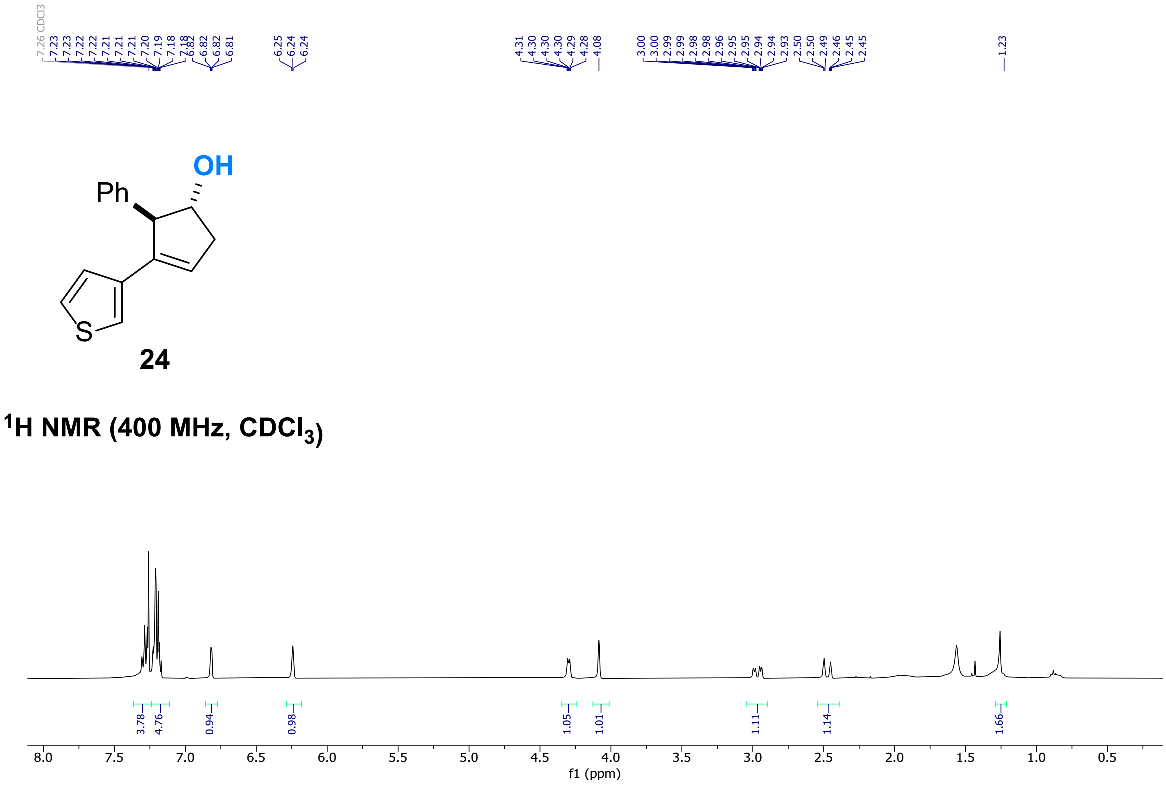

24

^{1}H NMR (400 MHz, CDCl$_3$)

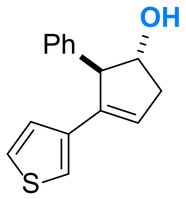

24

^{13}C NMR (100 MHz, $CDCl_3$)

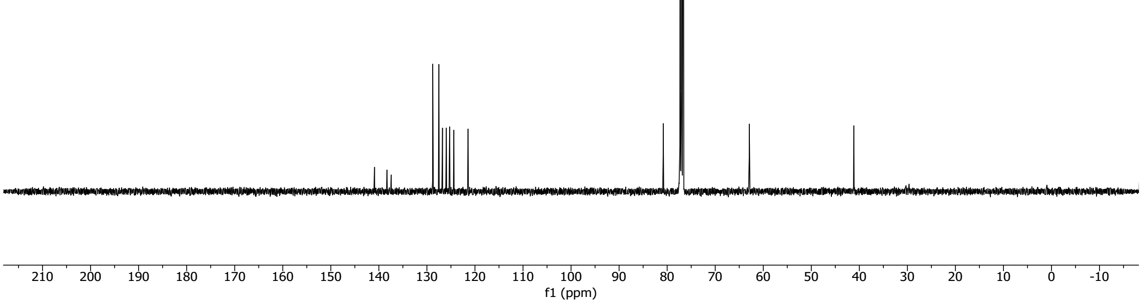

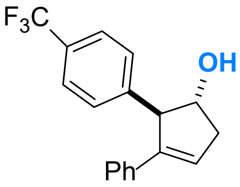

25

^{1}H NMR (400 MHz, $CDCl_3$)

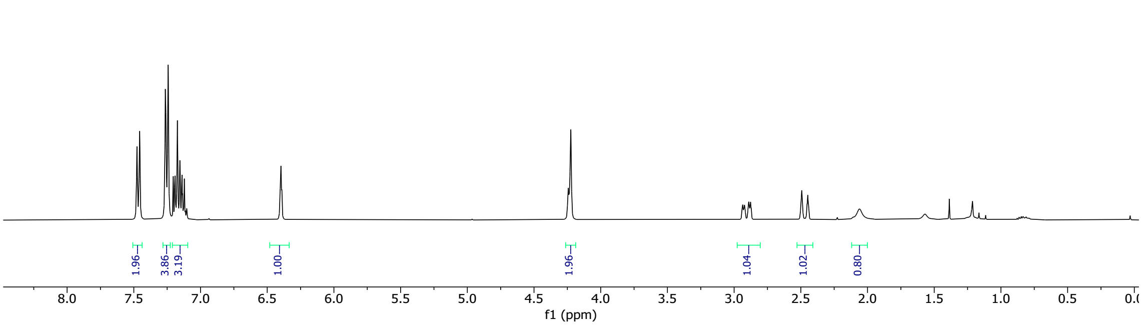

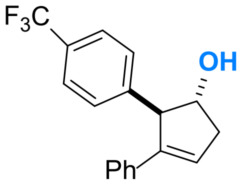

25

^{13}C NMR (100 MHz, CDCl$_3$)

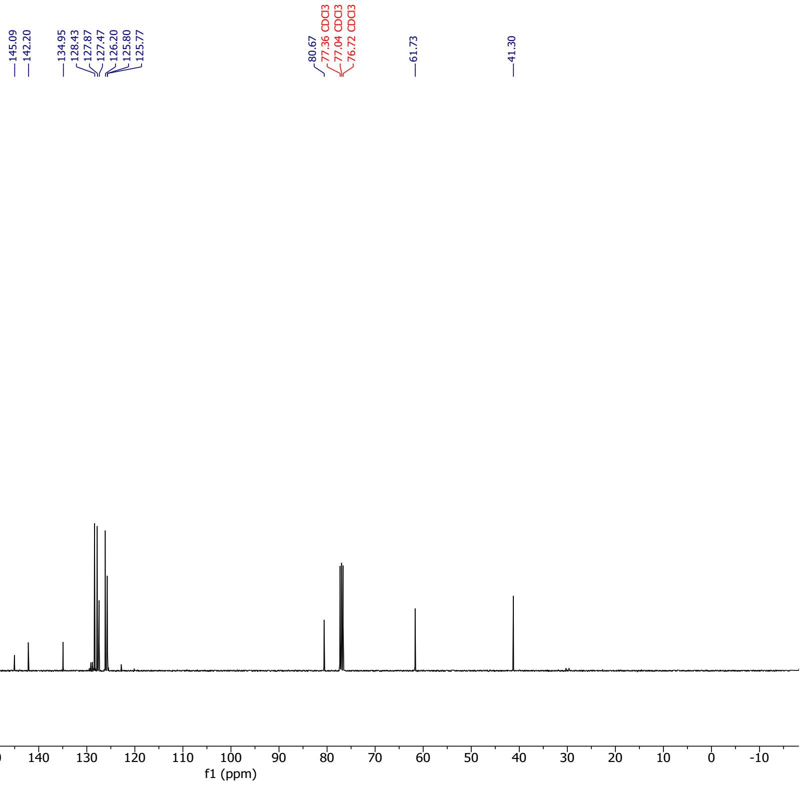

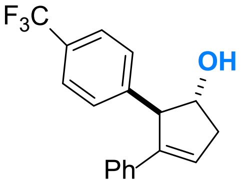

25

^{19}F NMR (377 MHz, CDCl$_3$)

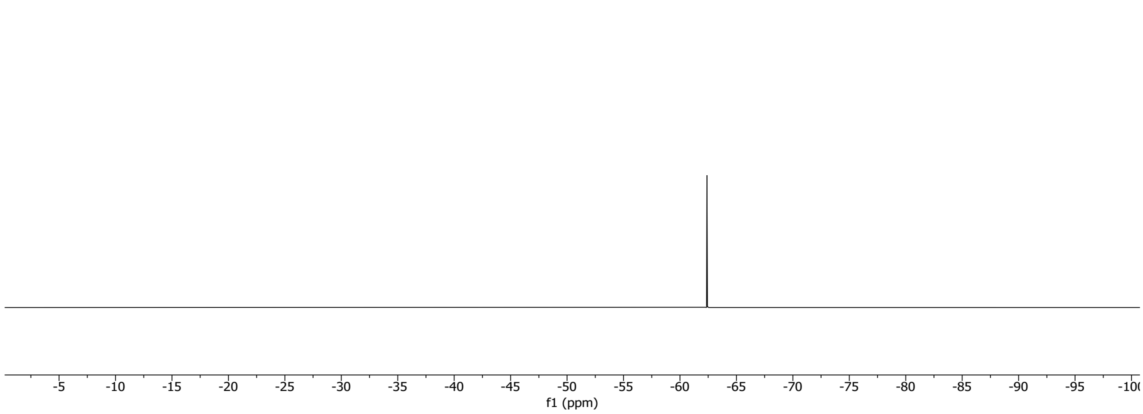

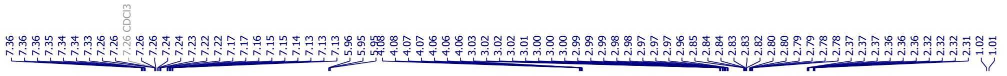

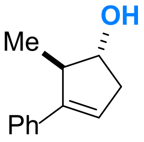

26

^{1}H NMR (400 MHz, CDCl$_3$)

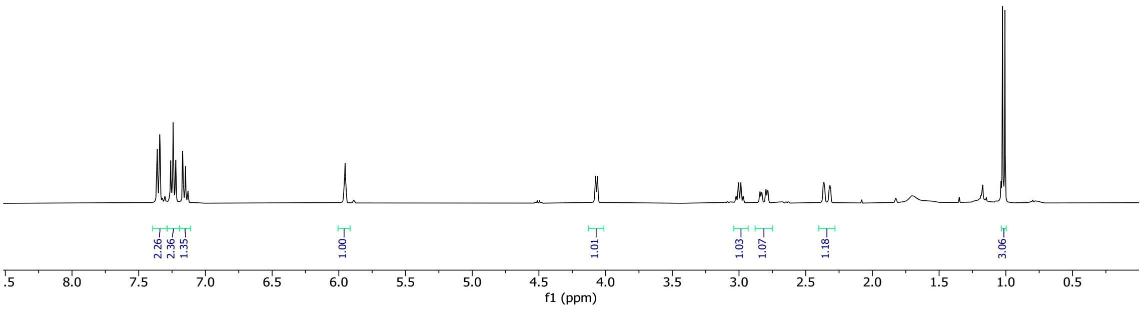

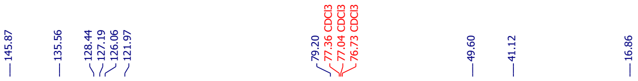

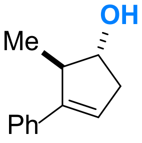

26

^{13}C NMR (100 MHz, CDCl$_3$)

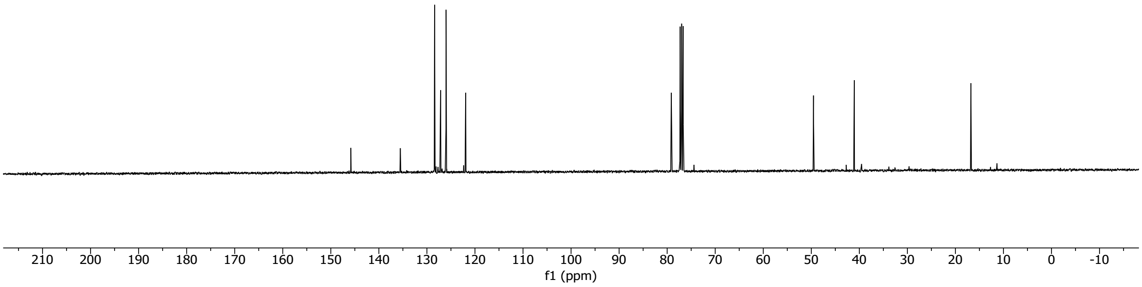

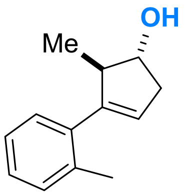

27

^{1}H NMR (400 MHz, CDCl$_3$)

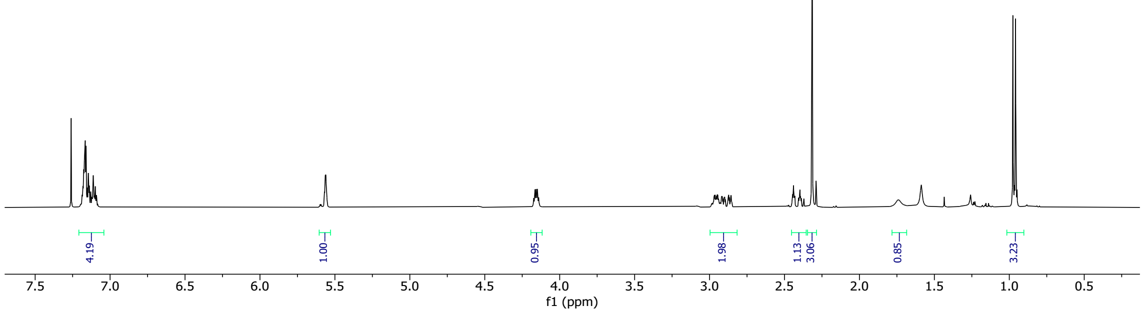

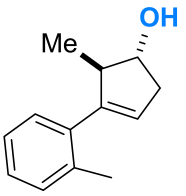

27

^{13}C NMR (100 MHz, CDCl$_3$)

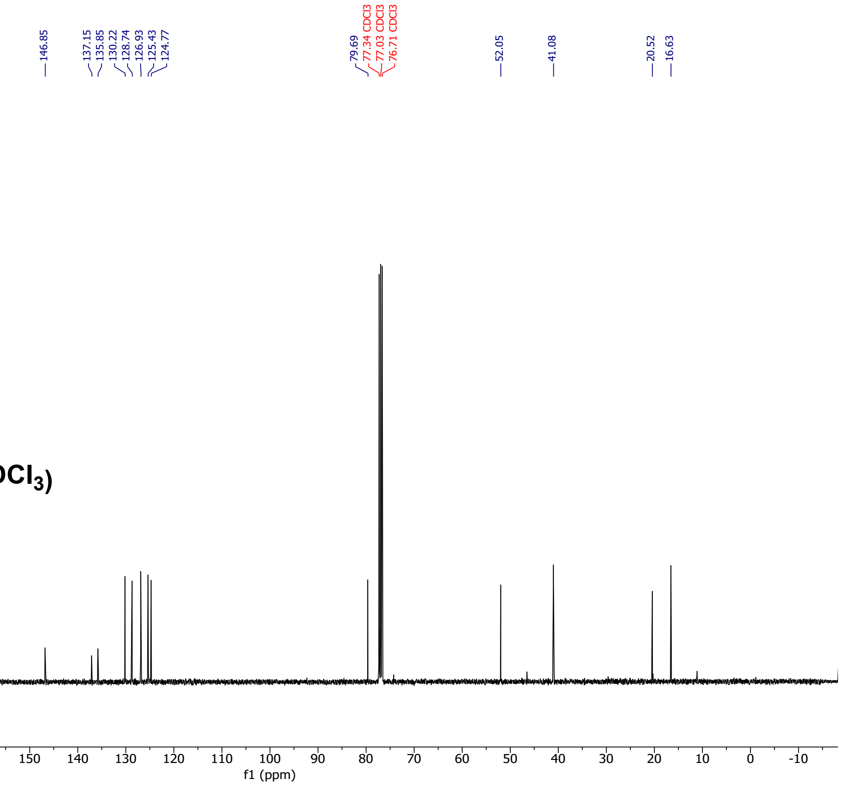

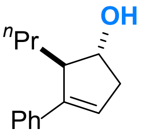

28

^{1}H NMR (400 MHz, $CDCl_3$)

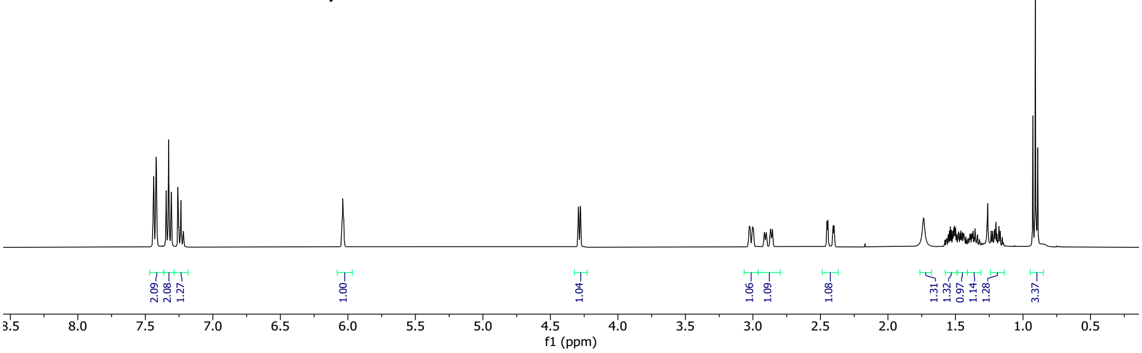

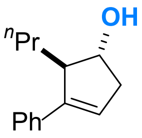

28

^{13}C NMR (100 MHz, $CDCl_3$)

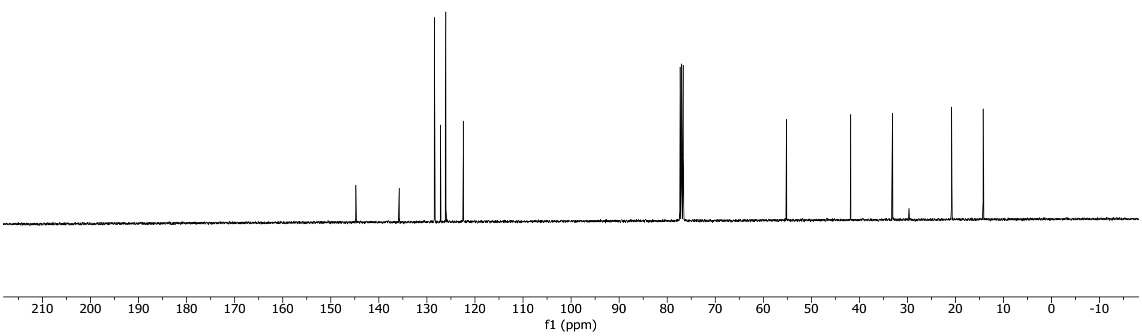

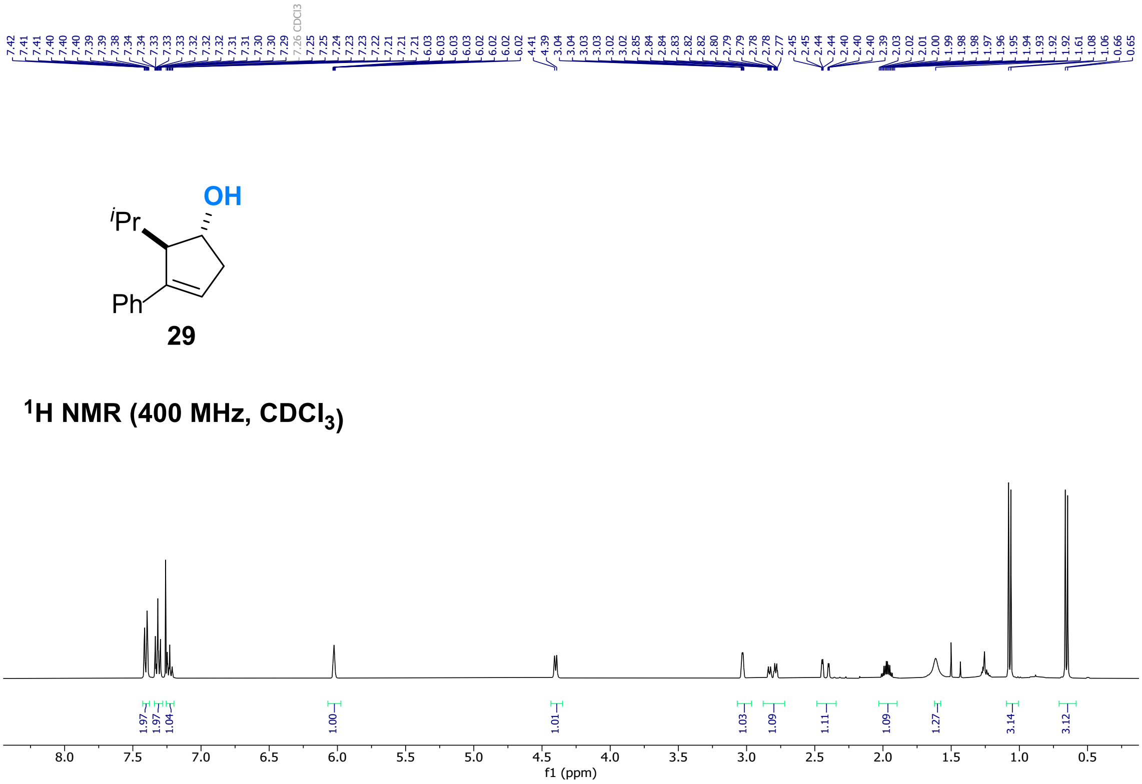

OH
iPr
Ph
29
1H NMR (400 MHz, CDCl3)
f1 (ppm)

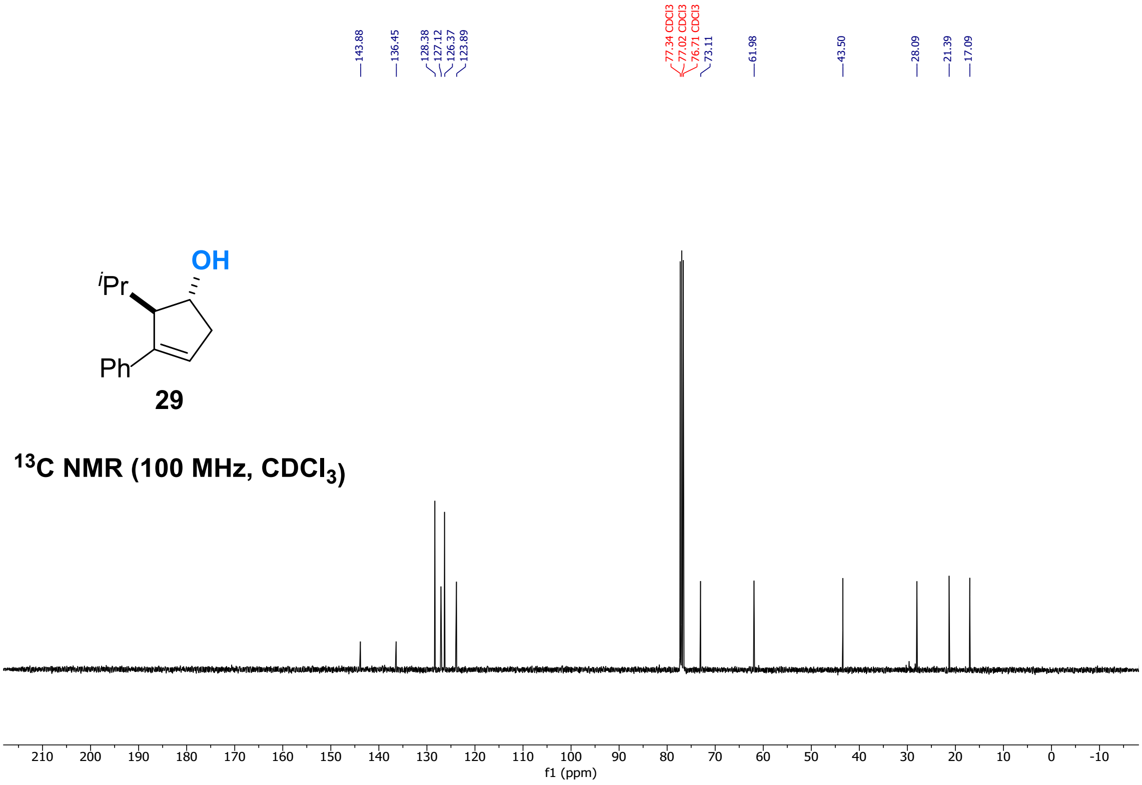

OH
iPr
Ph
29
13C NMR (100 MHz, CDCl3)
f1 (ppm)

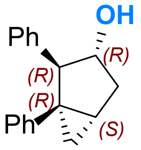

(1*R*,2*R*,3*R*,5*S*)-30

^{1}H NMR (400 MHz, $CDCl_3$)

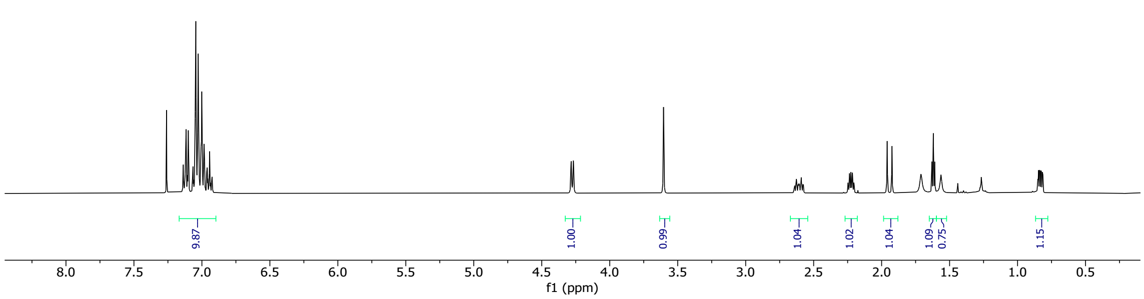

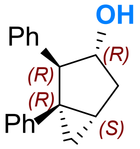

(1*R*,2*R*,3*R*,5*S*)-30

^{13}C NMR (100 MHz, $CDCl_3$)

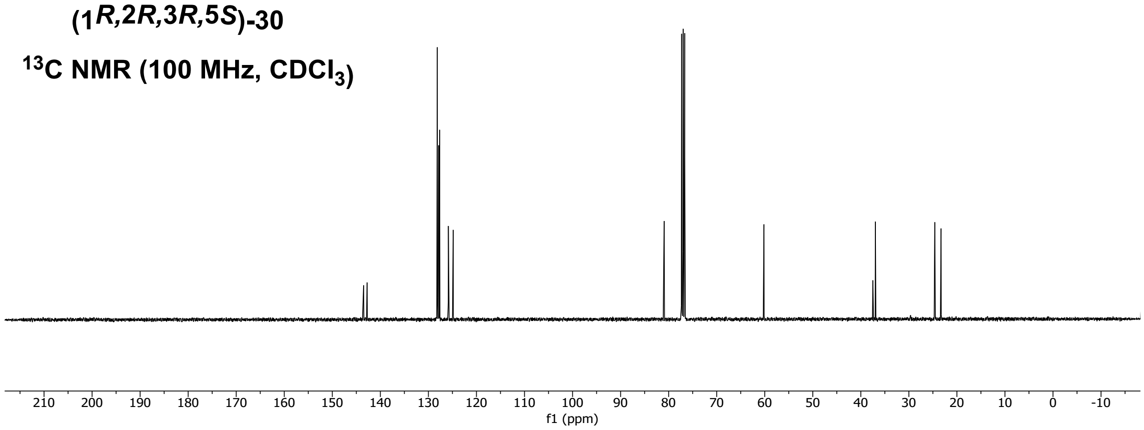

2D COSY experiment for (1*R*,2*R*,3*R*,5*S*)-**30**

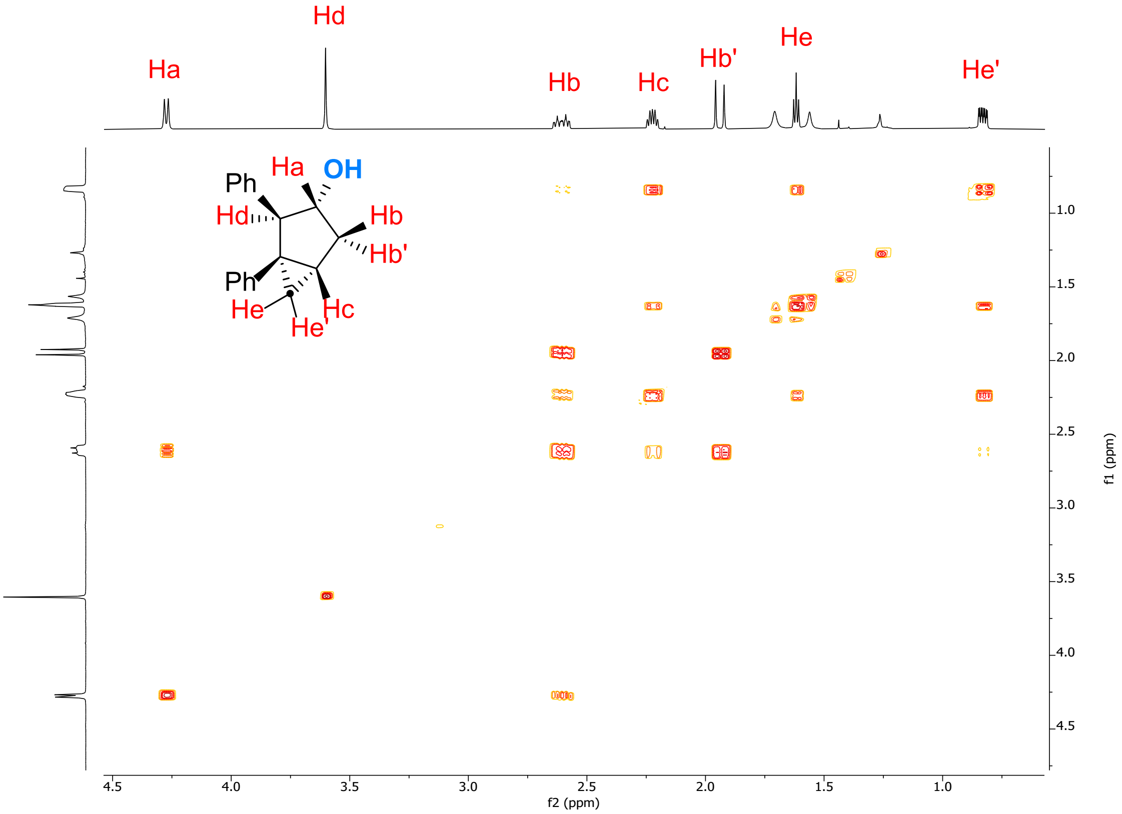

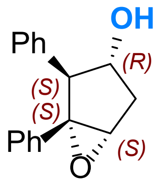

(1*S*,2*S*,3*R*,5*S*)-31

^{1}H NMR (300 MHz, $CDCl_3$)

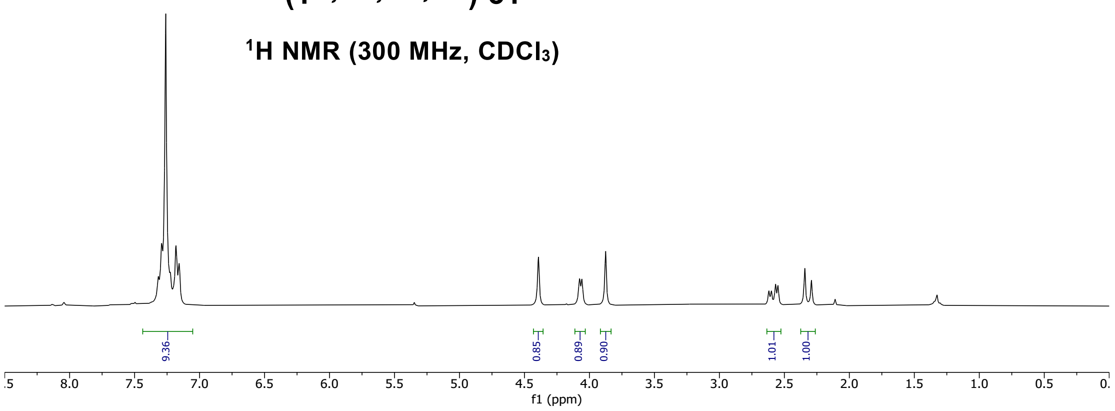

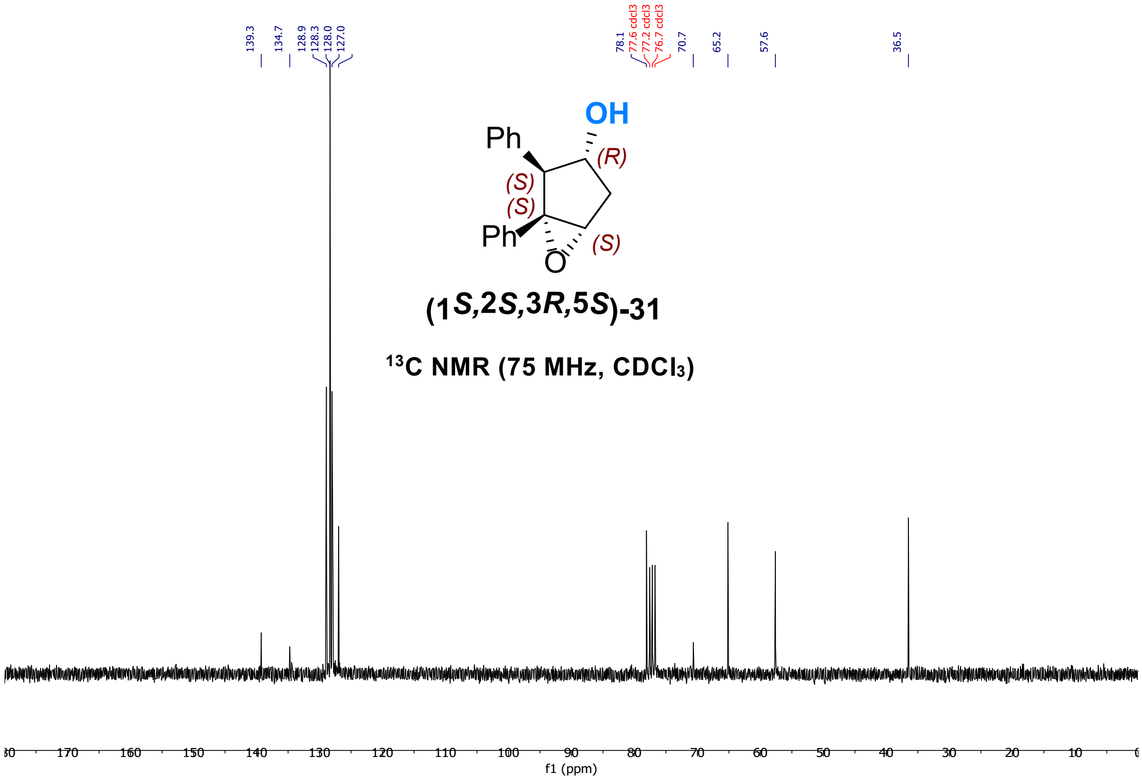

2D COSY experiment for (1*S*,2*S*,3*R*,5*S*)-**31**

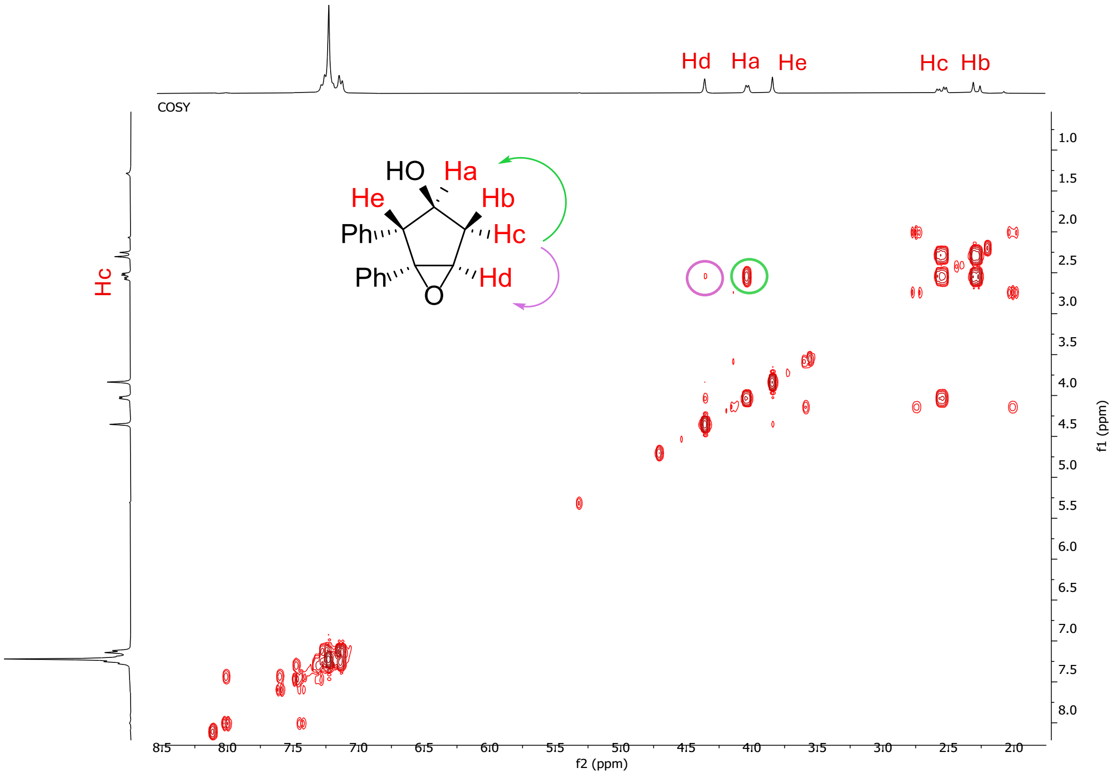

2D NOESY experiment for (1*S*,2*S*,3*R*,5*S*)-**31**

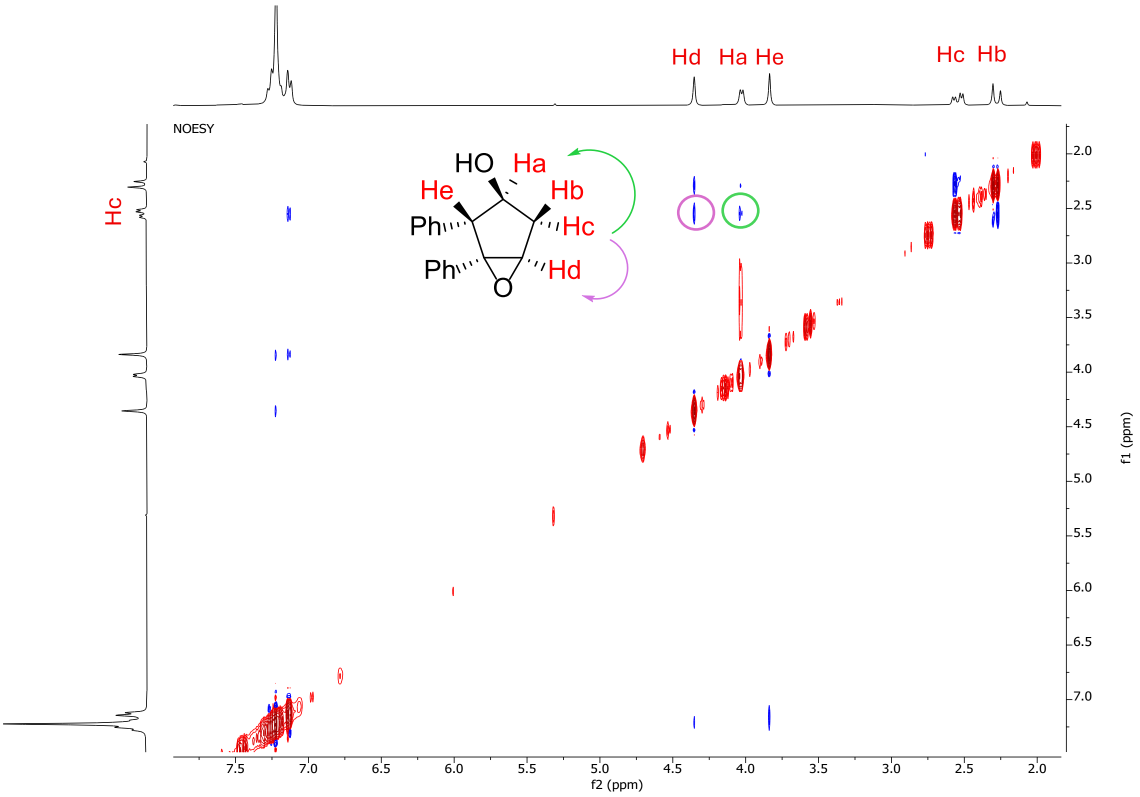

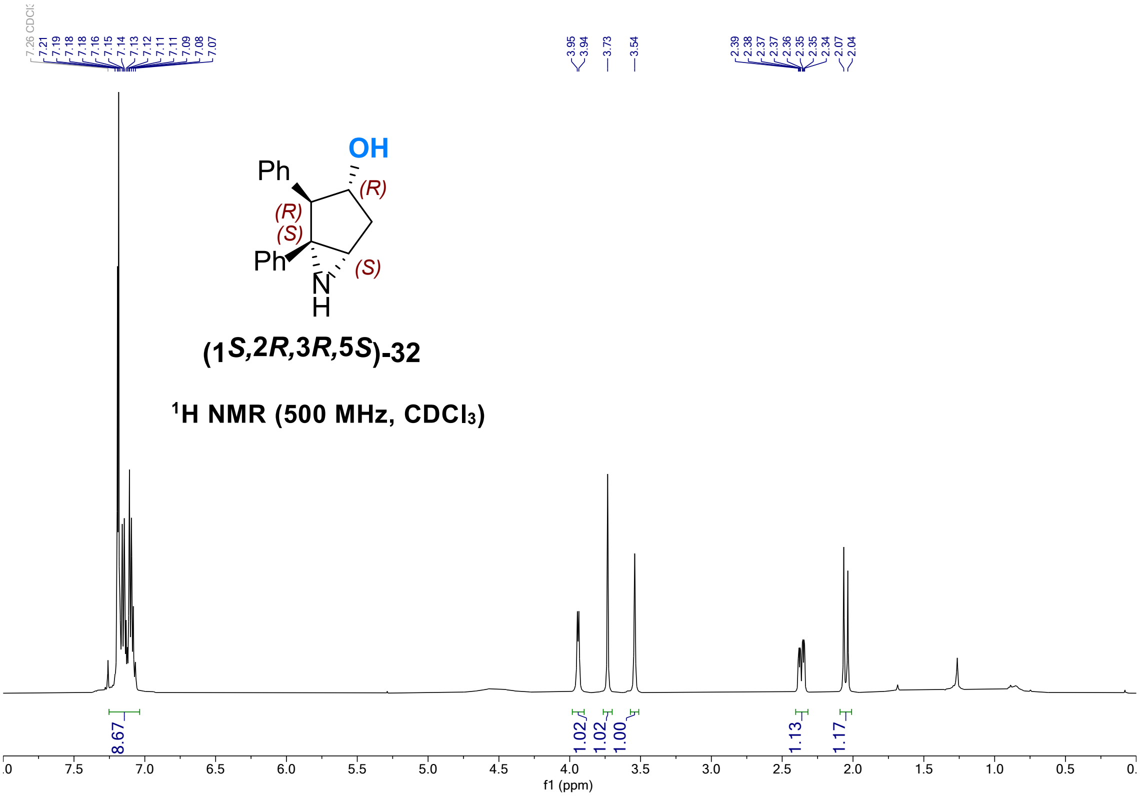

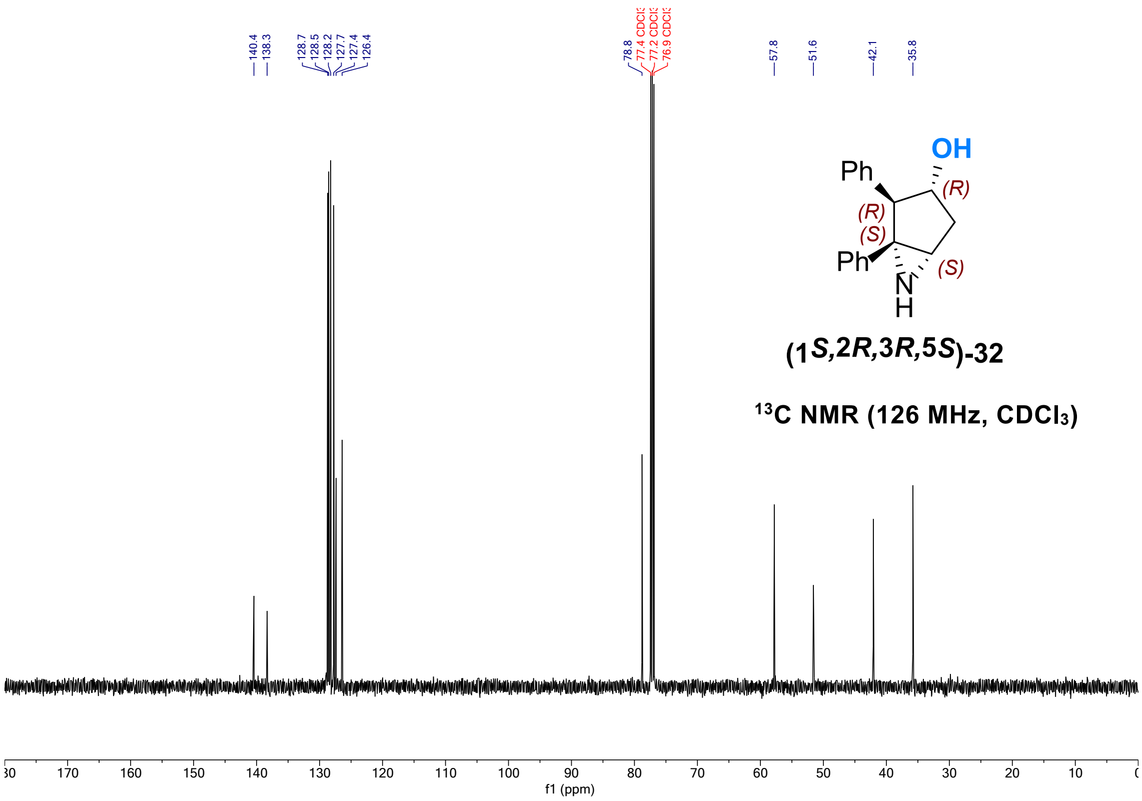

(1*S*,2*R*,3*R*,5*S*)-32

^{13}C NMR (126 MHz, CDCl$_3$)

2D NOESY experiment for (1*S*,2*R*,3*R*,5*S*)-**32**

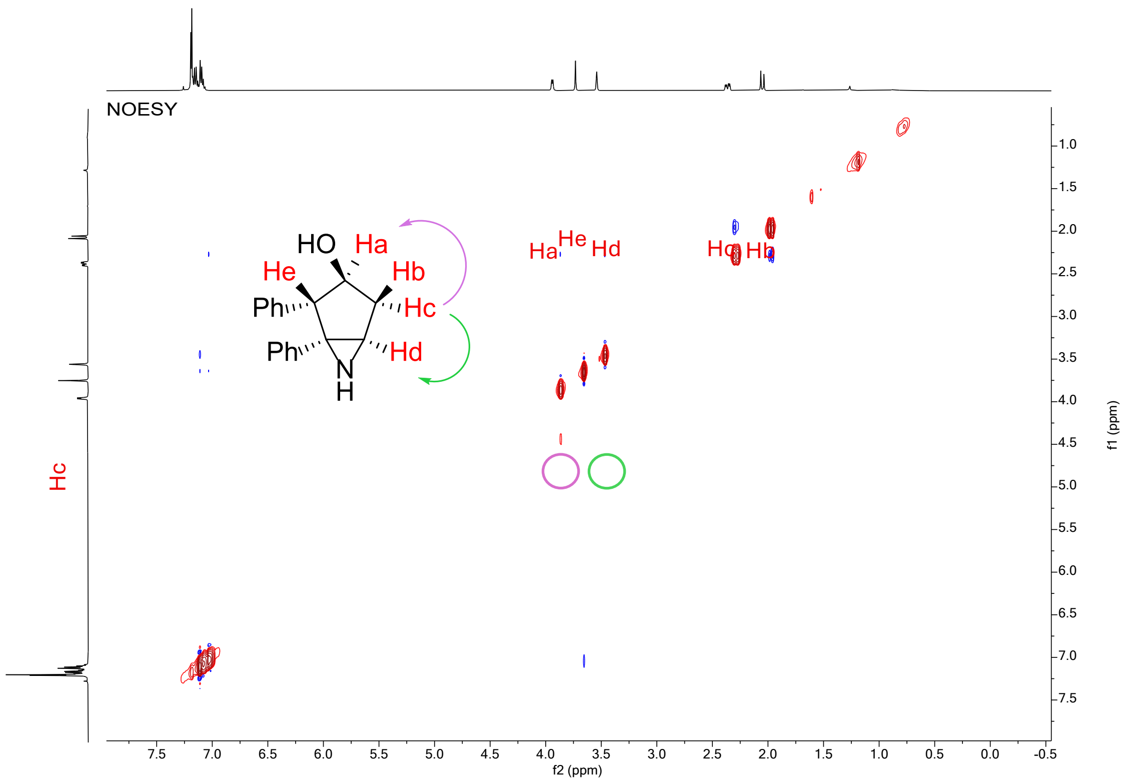

2D HMBC experiment for (1*S*,2*R*,3*R*,5*S*)-**32**

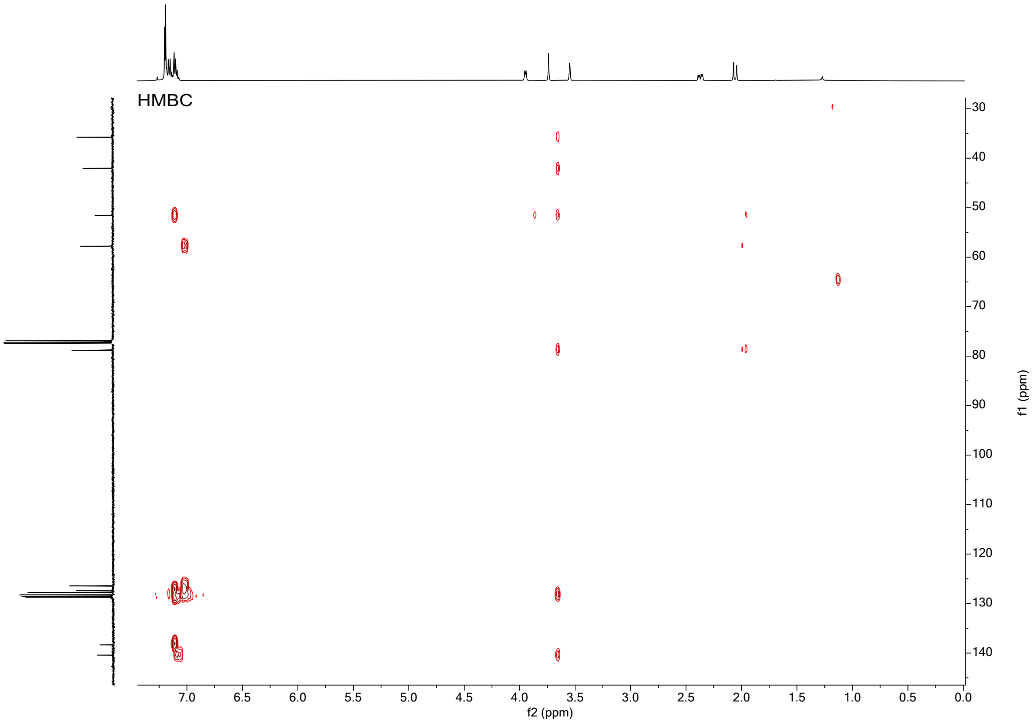

11B NMR experiment to demonstrate formation of boracycle intermediate

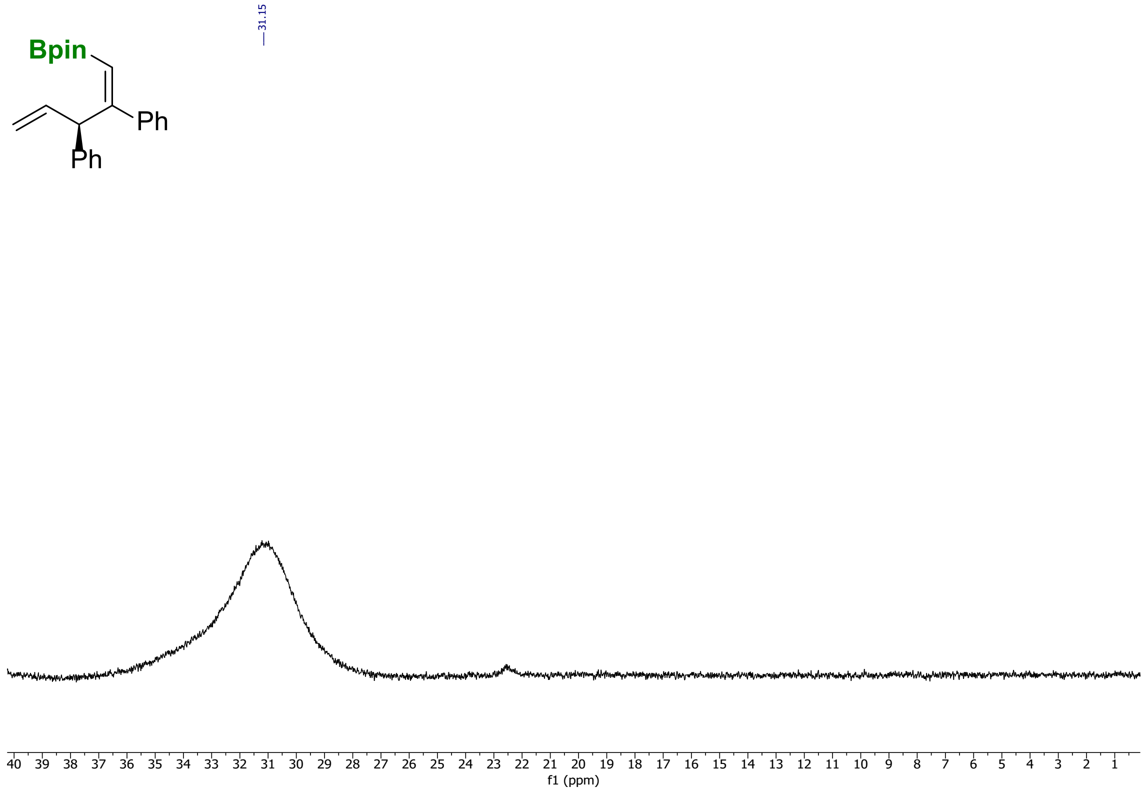

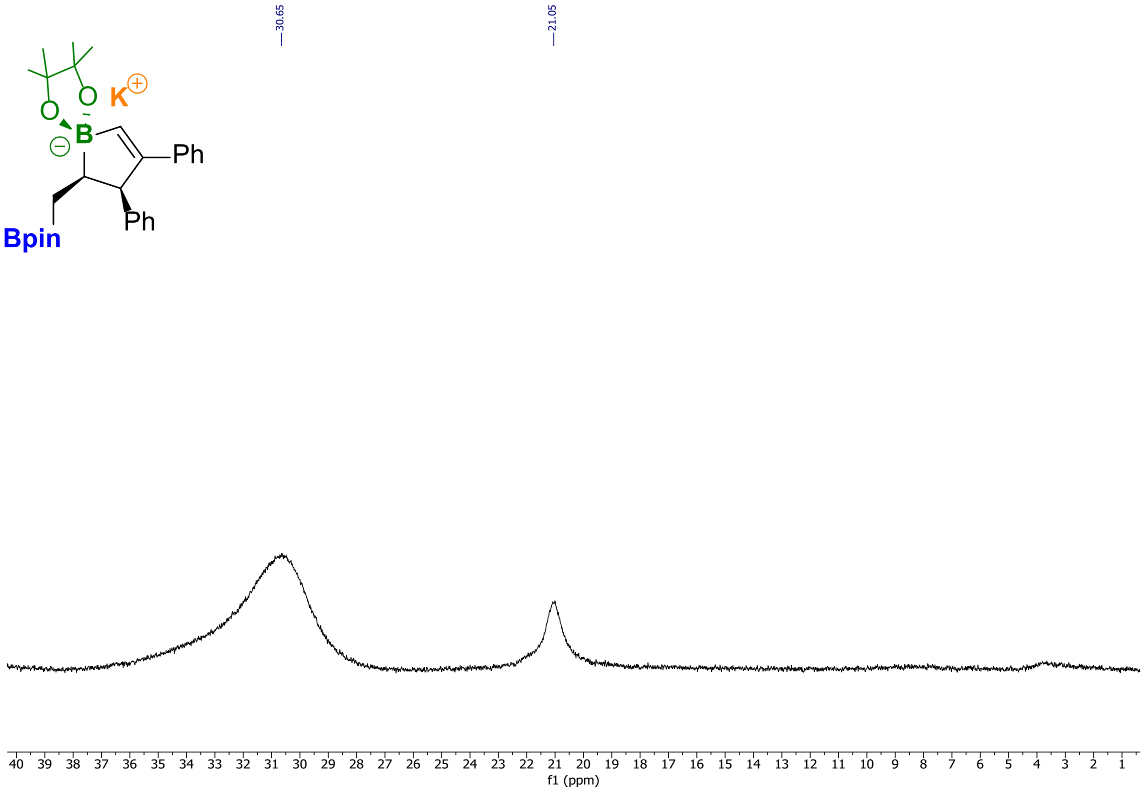

HPLC Analysis

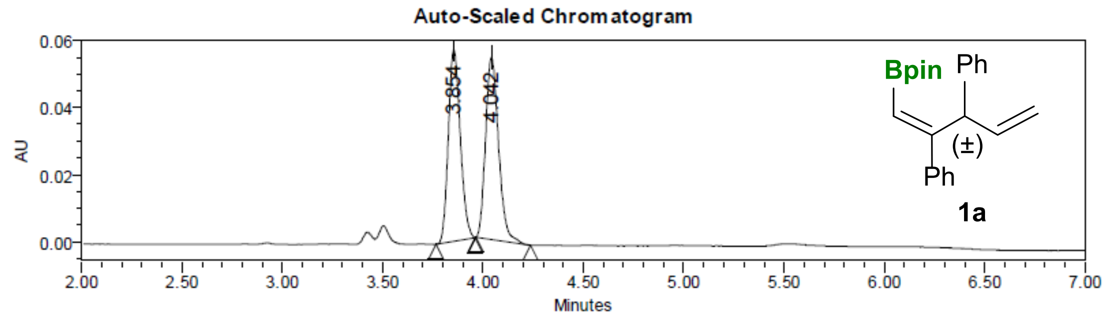

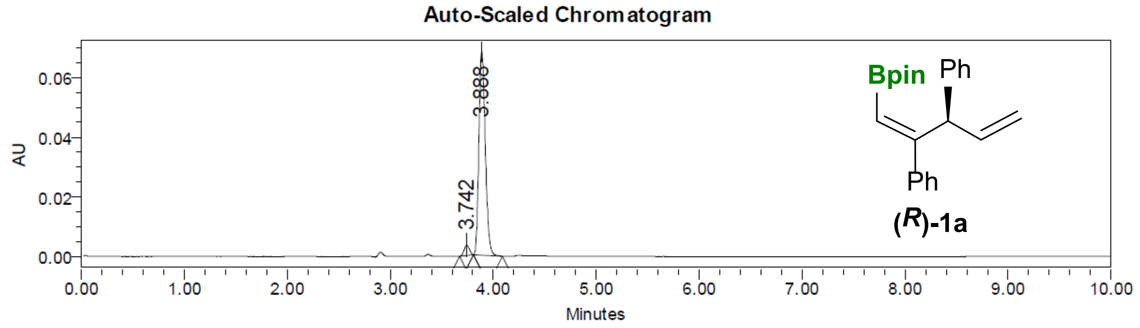

Peak Results

	Name	RT	Area	% Area
1		3.854	236754	49.10
2		4.042	245406	50.90

Peak Results

	Name	RT	Area	% Area
1		3.742	12114	3.94
2		3.888	295215	96.06

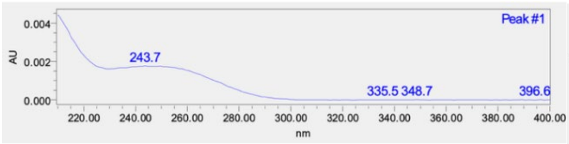

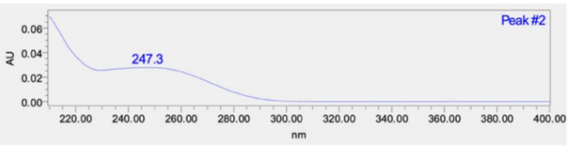

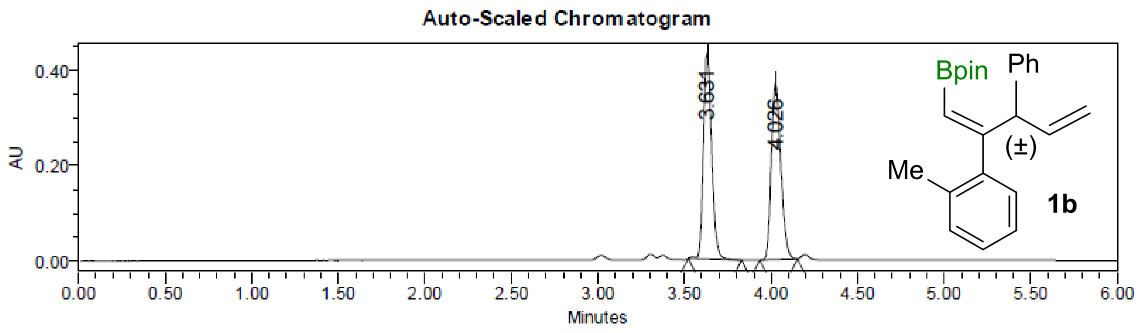

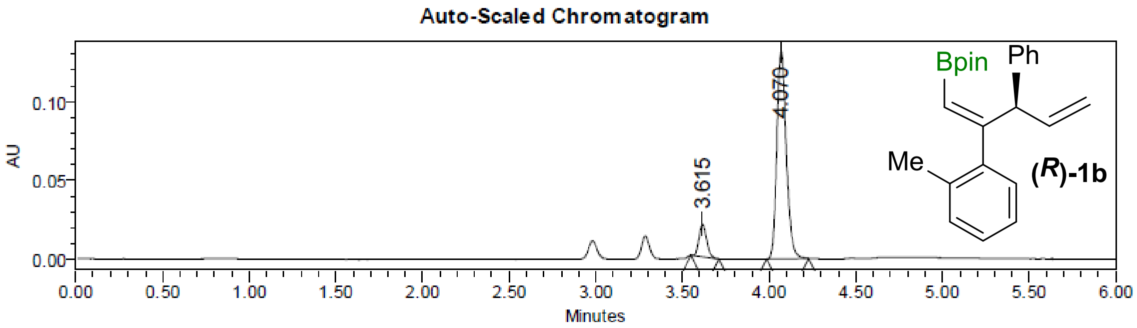

	Name	RT	Area	% Area
1		3.631	1425019	50.25
2		4.026	1410582	49.75

Peak Results

	Name	RT	Area	% Area
1		3.615	63715	11.33
2		4.070	498571	88.67

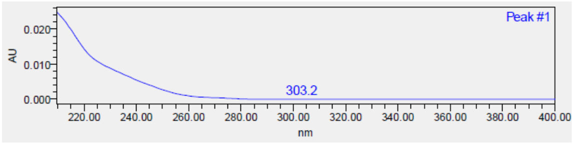

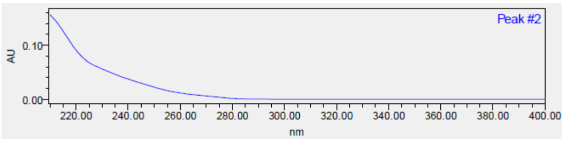

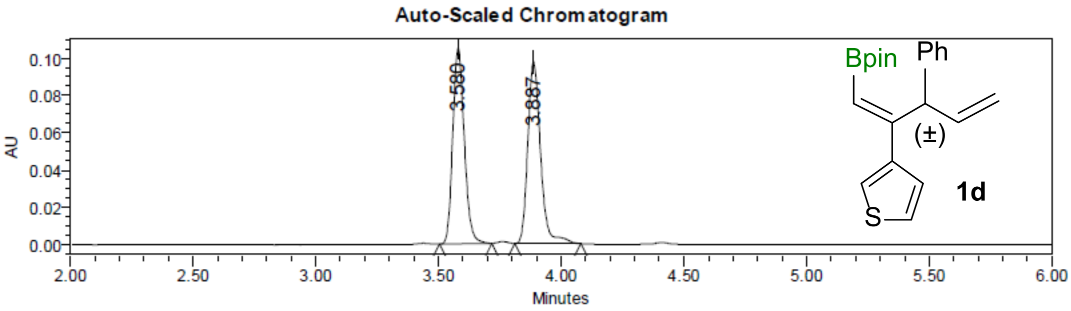

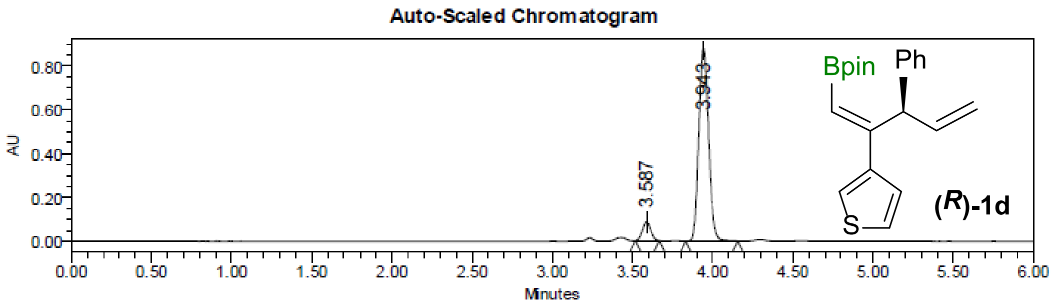

Peak Results

	Name	RT	Area	% Area
1		3.580	342347	49.27
2		3.887	352423	50.73

Peak Results

	Name	RT	Area	% Area
1		3.587	322872	7.96
2		3.943	3732413	92.04

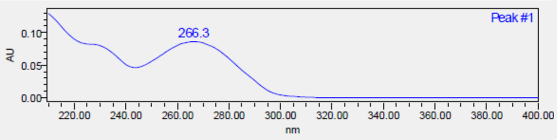

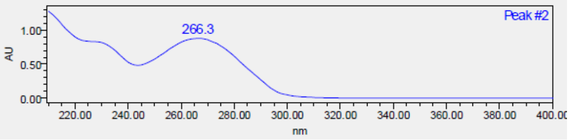

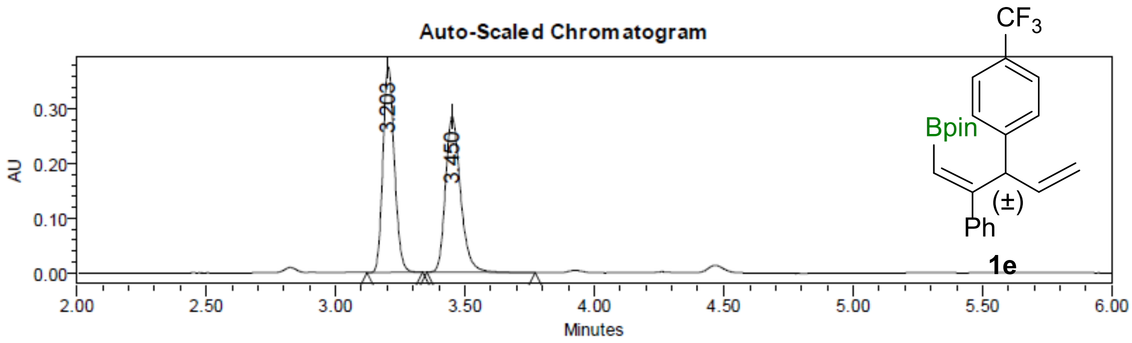

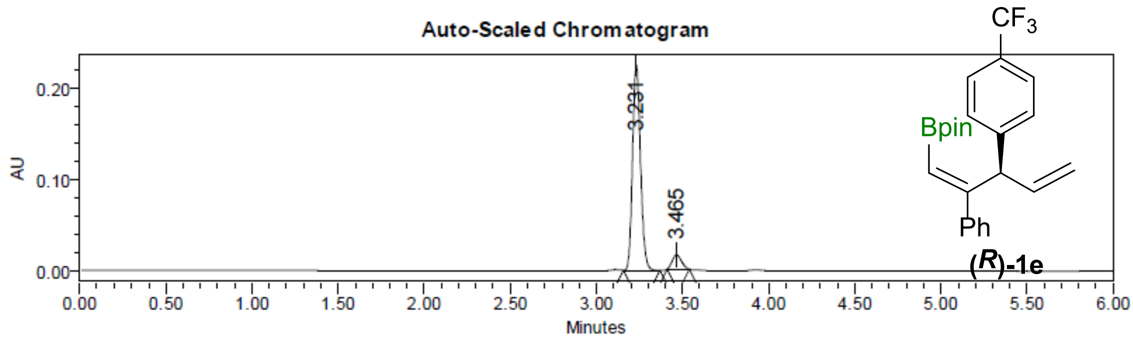

Peak Results

	Name	RT	Area	% Area
1		3.203	1155742	50.05
2		3.450	1153323	49.95

Peak Results

	Name	RT	Area	% Area
1		3.231	721005	92.56
2		3.465	57926	7.44

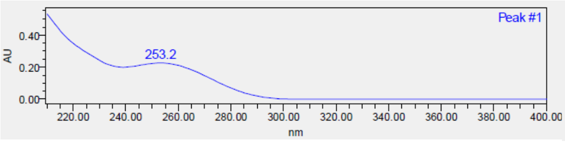

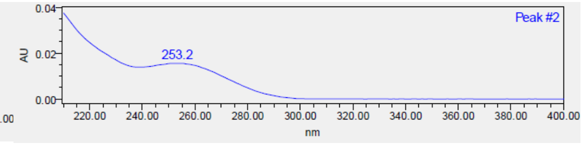

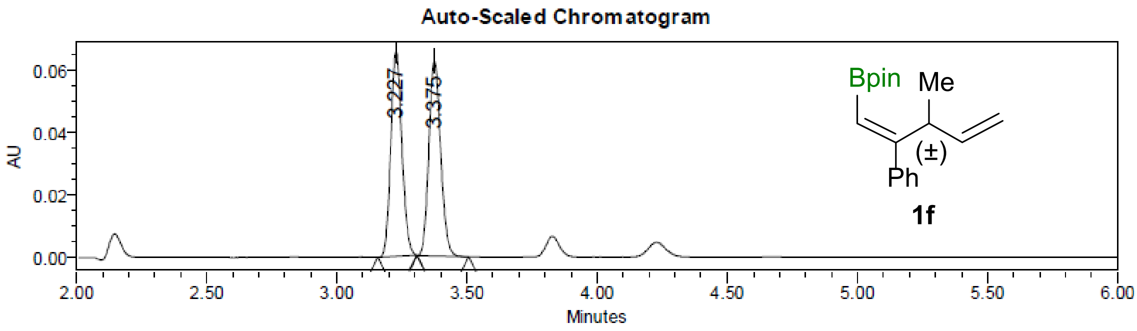

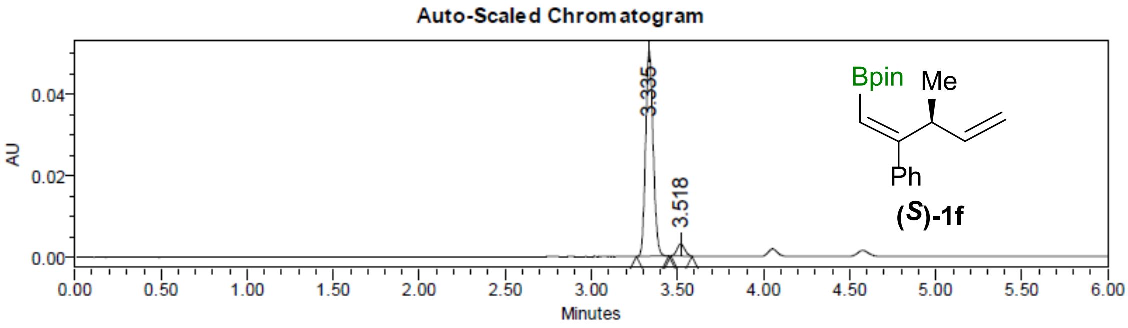

Peak Results

	Name	RT	Area	% Area
1		3.227	194516	49.67
2		3.375	197132	50.33

Peak Results

	Name	RT	Area	% Area
1		3.335	154090	94.48
2		3.518	9009	5.52

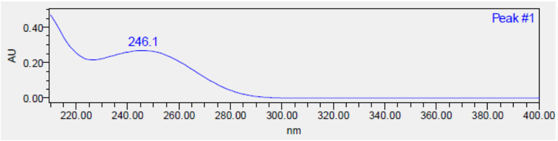

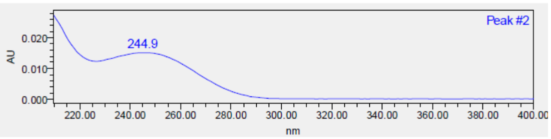

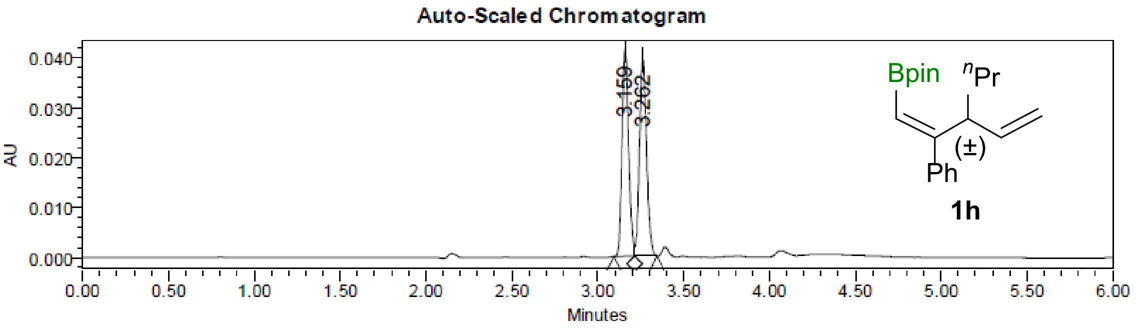

Auto-Scaled Chromatogram

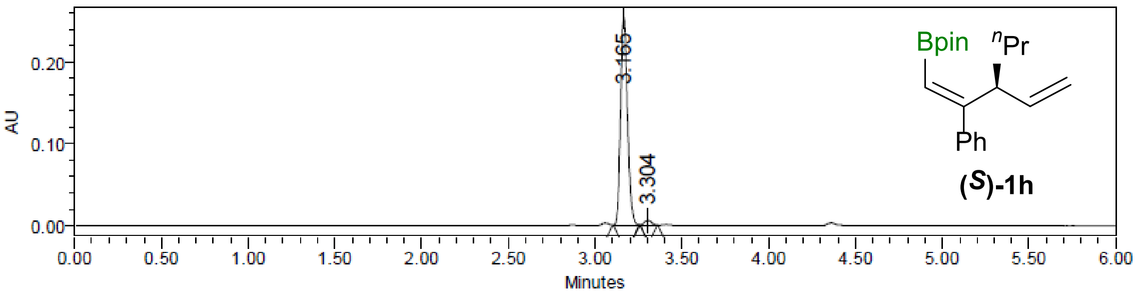

Peak Results

	Name	RT	Area	% Area
1		3.159	111387	49.83
2		3.262	112145	50.17

Peak Results

	Name	RT	Area	% Area
1		3.165	697954	98.03
2		3.304	14051	1.97

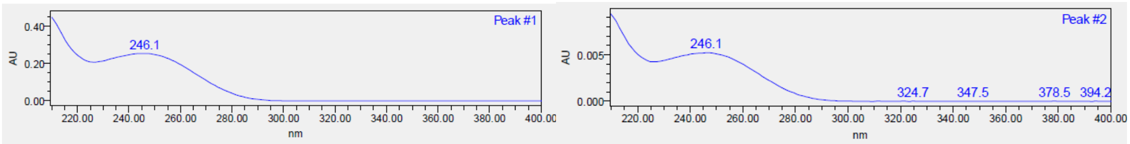

Auto-Scaled Chromatogram

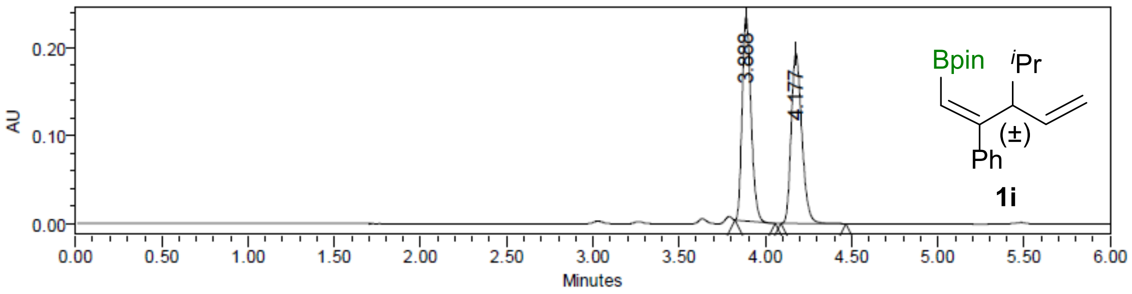

Auto-Scaled Chromatogram

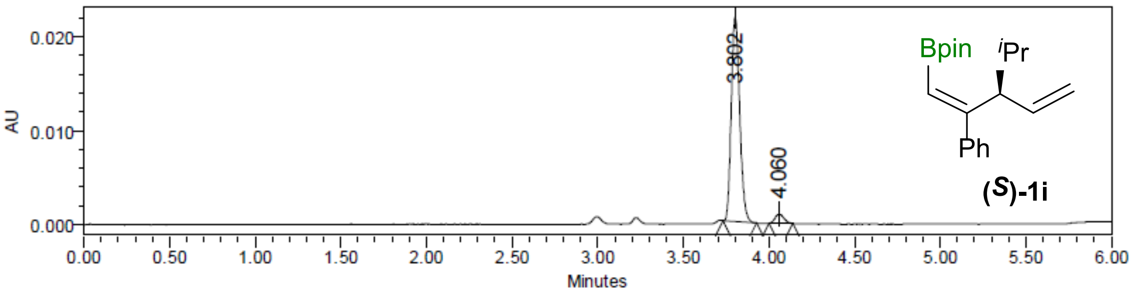

Peak Results

	Name	RT	Area	% Area
1		3.888	827235	49.73
2		4.177	836377	50.27

Peak Results

	Name	RT	Area	% Area
1		3.802	74322	95.47
2		4.060	3527	4.53

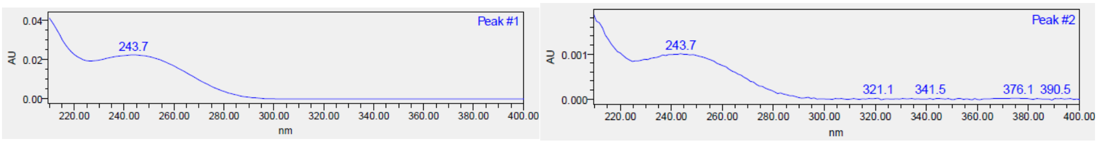

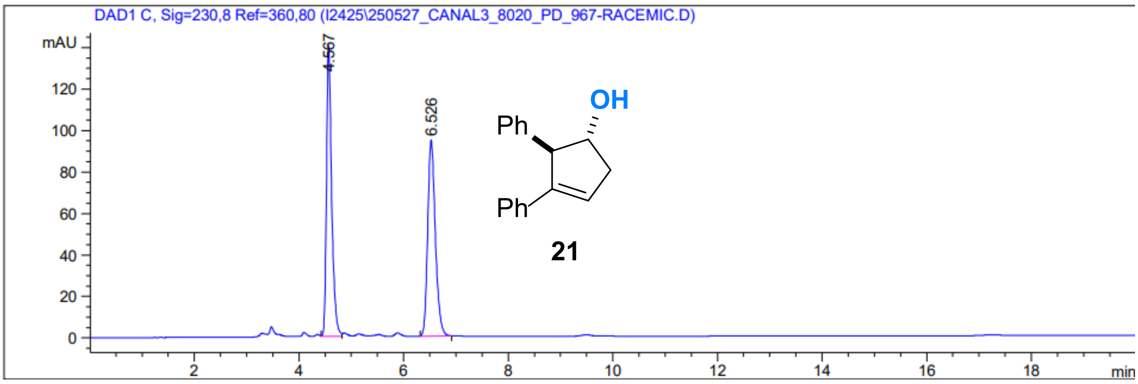

Peak #	RetTime [min]	Type	Width [min]	Area [mAU*s]	Height [mAU]	Area %
1	4.573	BB	0.1016	21.77311	3.19273	4.8059
2	6.542	BB	0.1549	431.27646	42.30873	95.1941

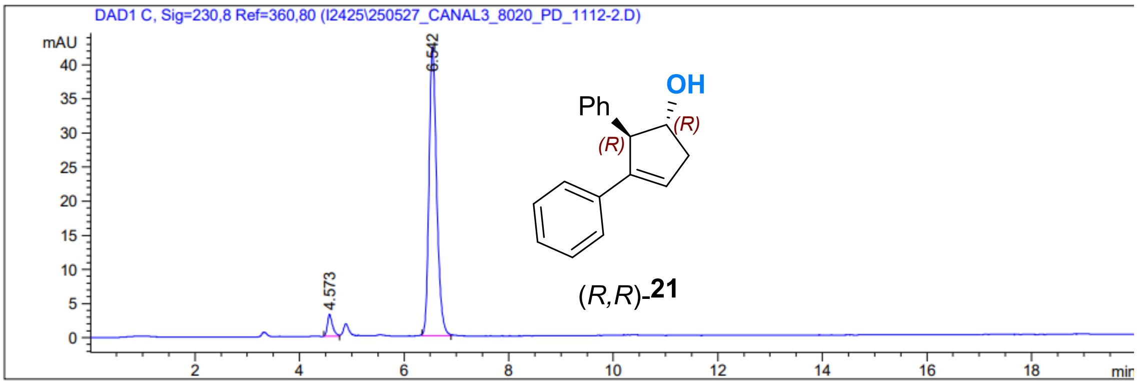

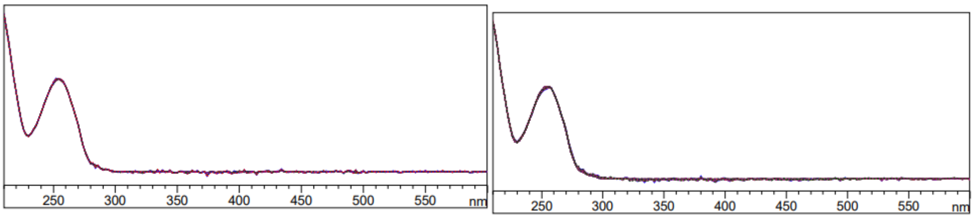

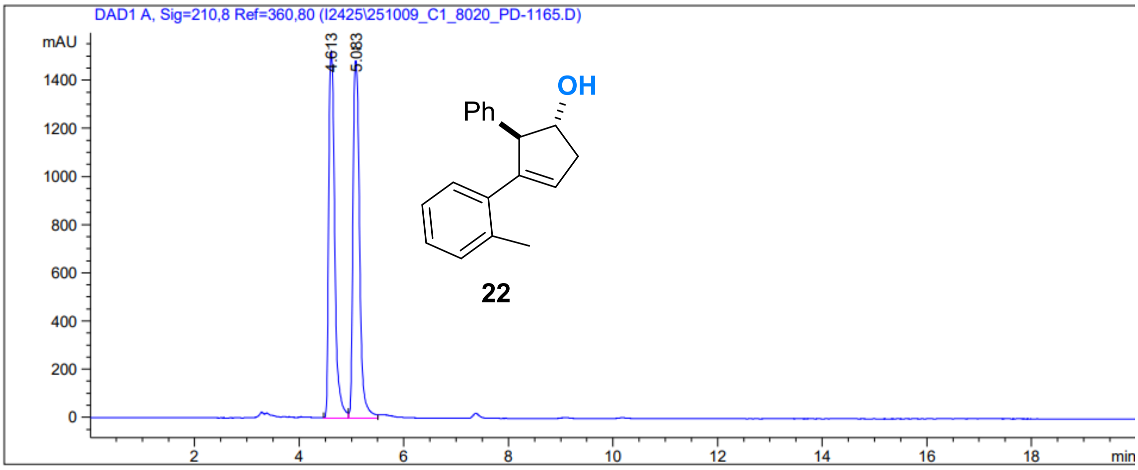

Peak #	RetTime [min]	Type	Width [min]	Area [mAU*s]	Height [mAU]	Area %
1	4.605	MM	0.0919	839.47260	152.19389	11.6585
2	5.074	VV	0.1096	6361.05225	909.08423	88.3415

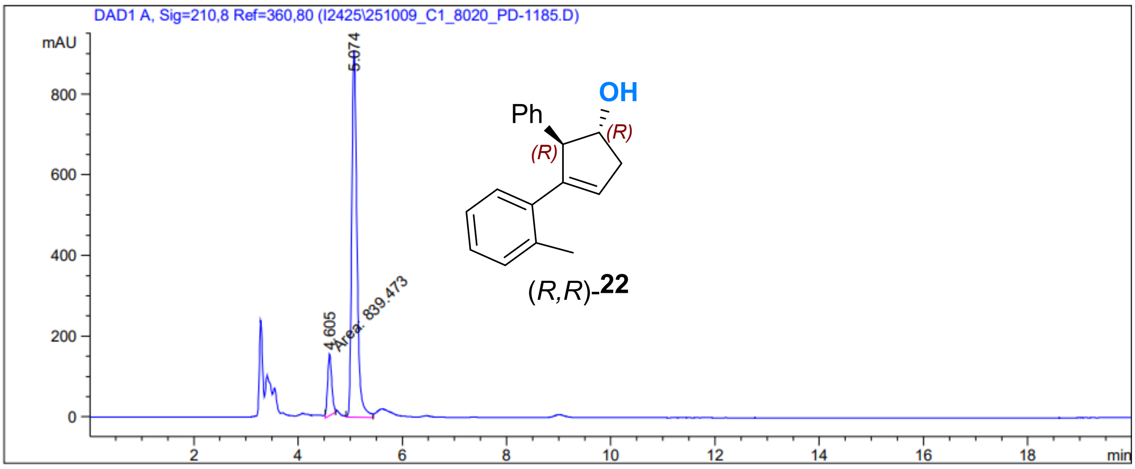

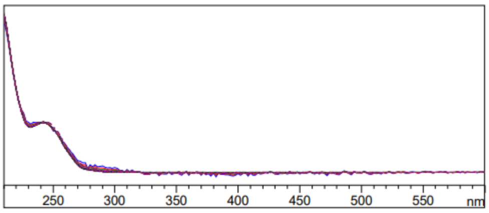

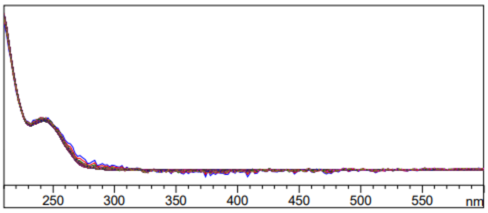

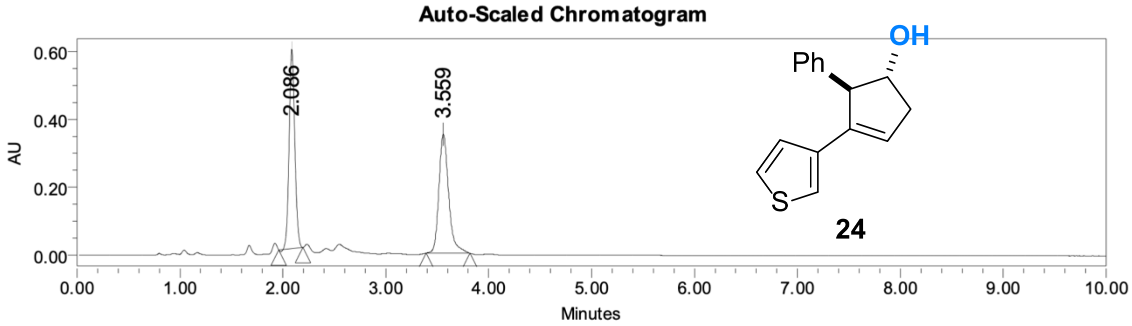

Peak Results

	Name	RT	Area	% Area
1		2.078	693435	10.96
2		3.504	5635973	89.04

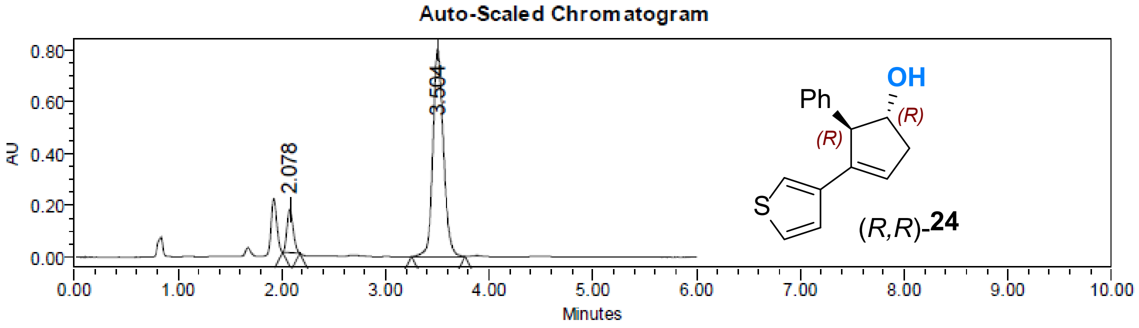

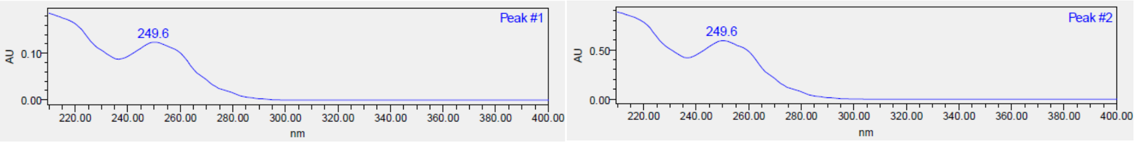

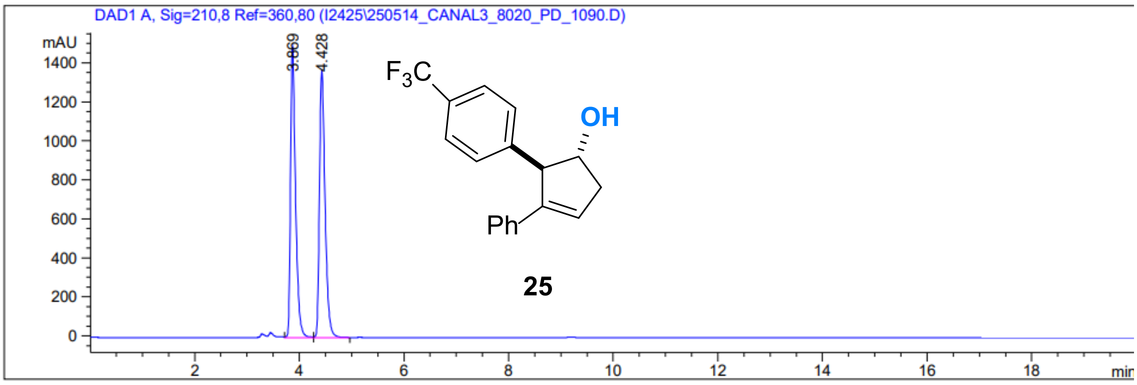

Peak #	RetTime [min]	Type	Width [min]	Area [mAU*s]	Height [mAU]	Area %
1	3.832	MM	0.2360	738.09821	52.11480	4.5762
2	4.219	MM	0.1601	1.53909e4	1602.23401	95.4238

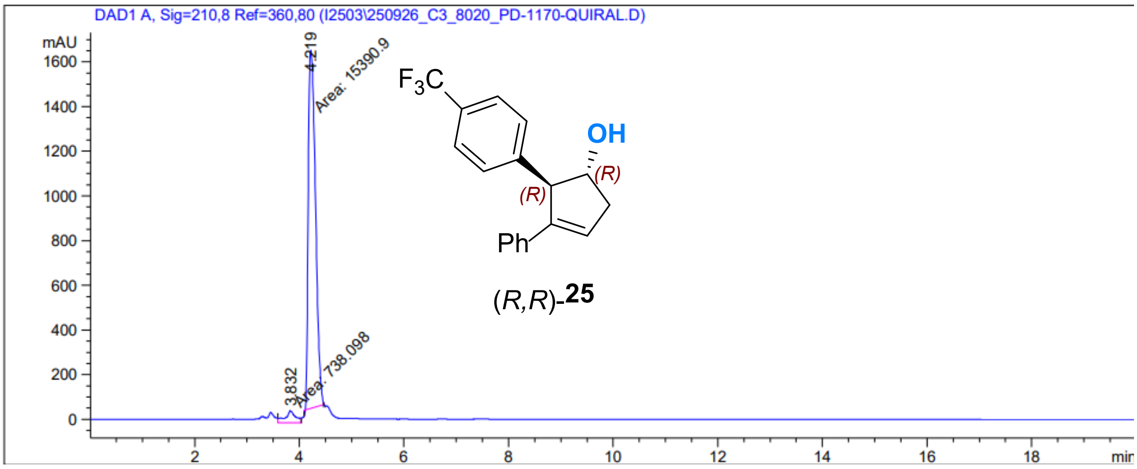

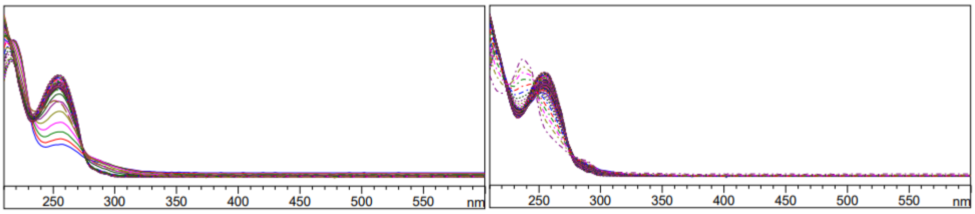

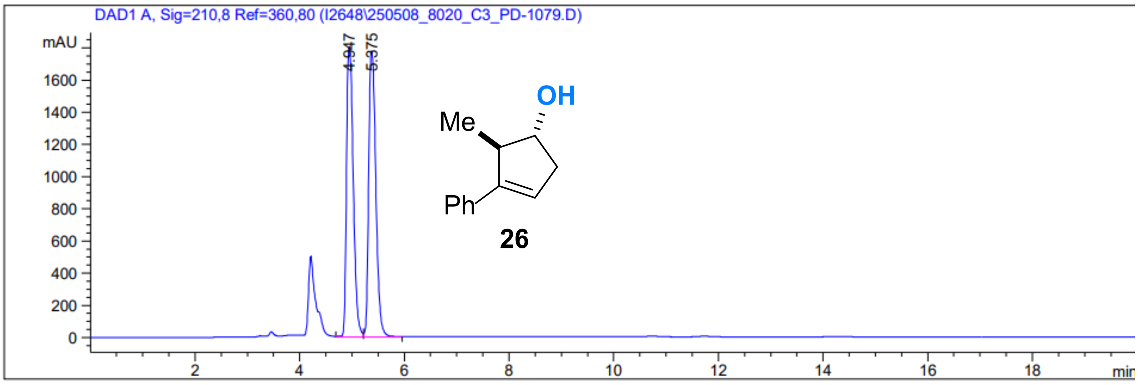

Peak #	RetTime [min]	Type	Width [min]	Area [mAU*s]	Height [mAU]	Area %
1	4.803	VV	0.1123	1091.43152	144.14288	5.7277
2	6.686	VB	0.1884	1.79638e4	1492.44141	94.2723

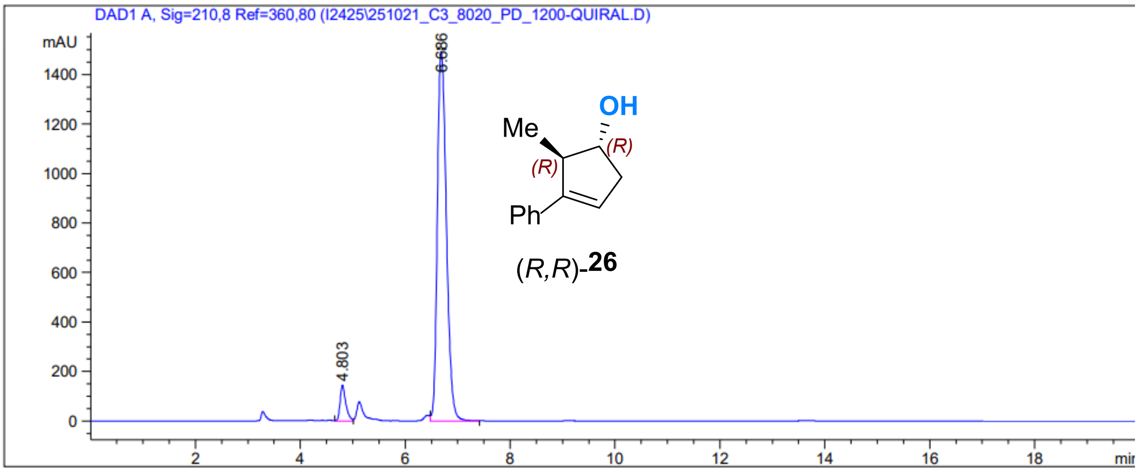

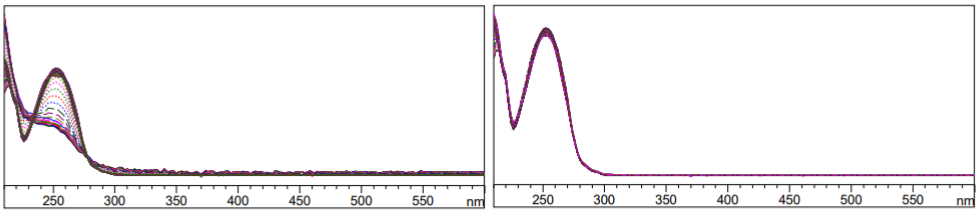

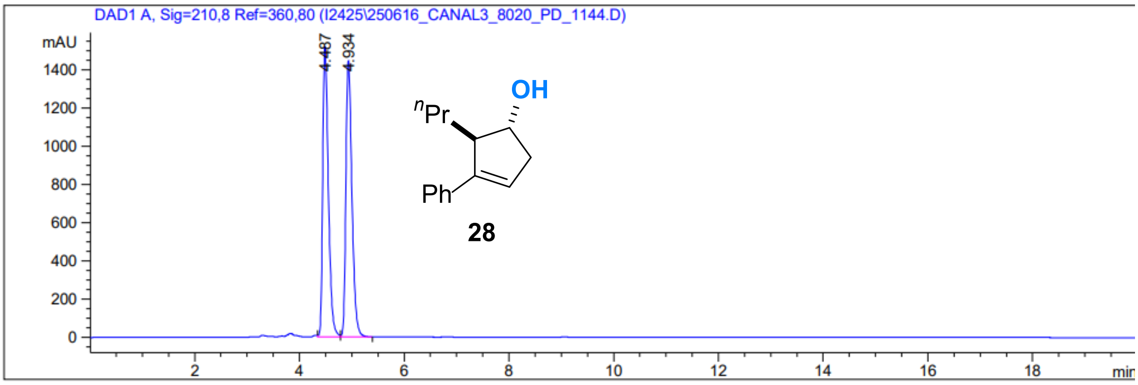

Peak #	RetTime [min]	Type	Width [min]	Area [mAU*s]	Height [mAU]	Area %
1	4.491	VV	0.1067	239.02304	32.91098	2.7254
2	4.936	VB	0.1200	8531.17480	1058.35156	97.2746

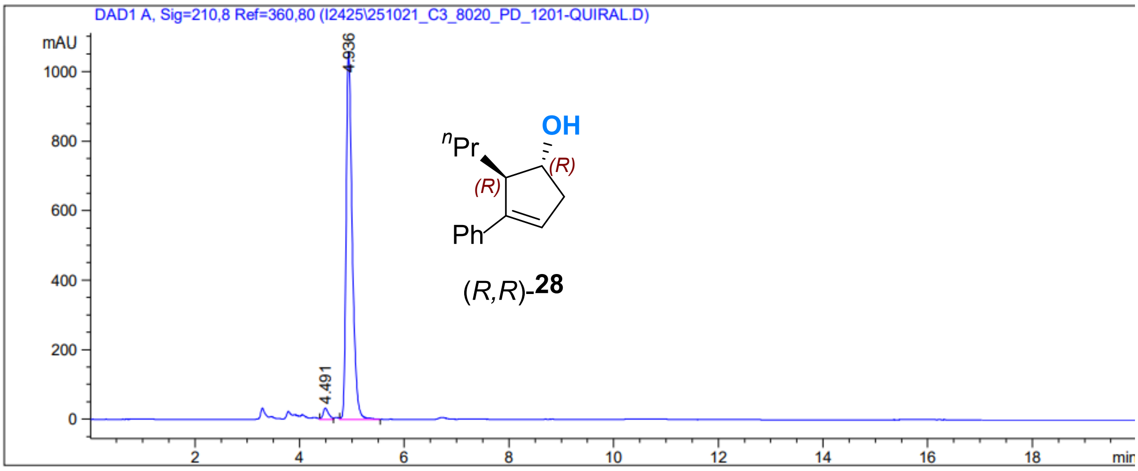

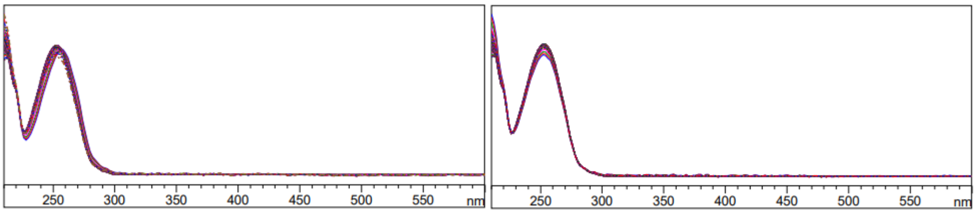

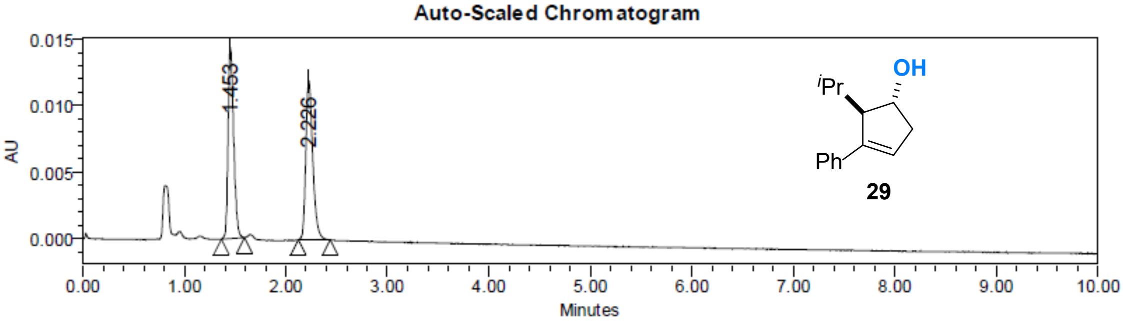

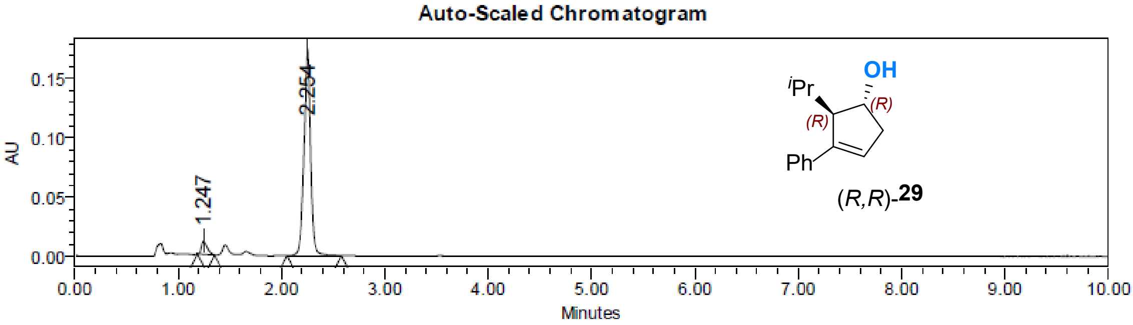

Peak Results

	Name	RT	Area	% Area
1		1.453	54687	49.29
2		2.226	56262	50.71

Peak Results

	Name	RT	Area	% Area
1		1.247	43087	5.26
2		2.254	775334	94.74

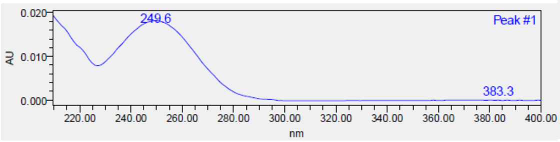

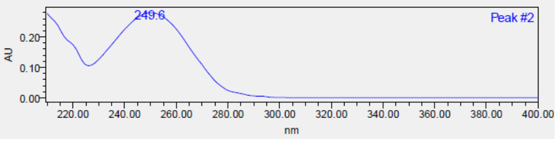

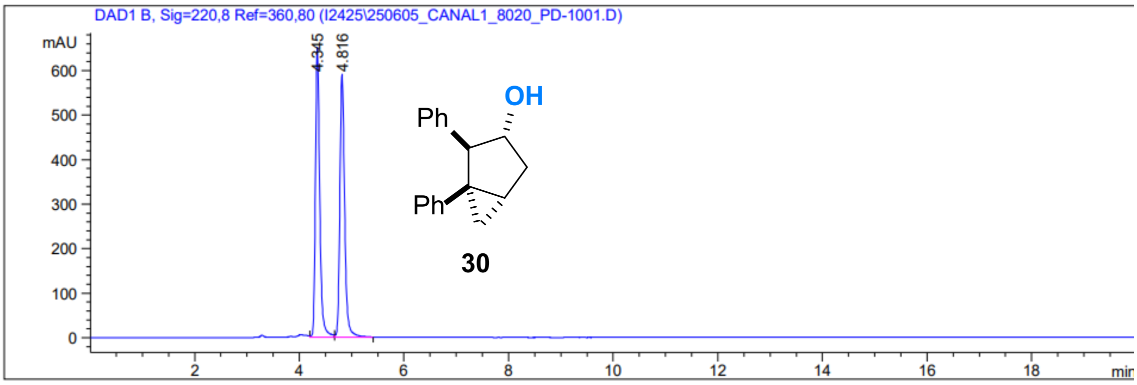

Peak #	RetTime [min]	Type	Width [min]	Area [mAU*s]	Height [mAU]	Area %
1	4.343	VV	0.0935	1.05000e4	1713.83447	93.7060
2	4.816	VV	0.1028	705.25714	101.79942	6.2940

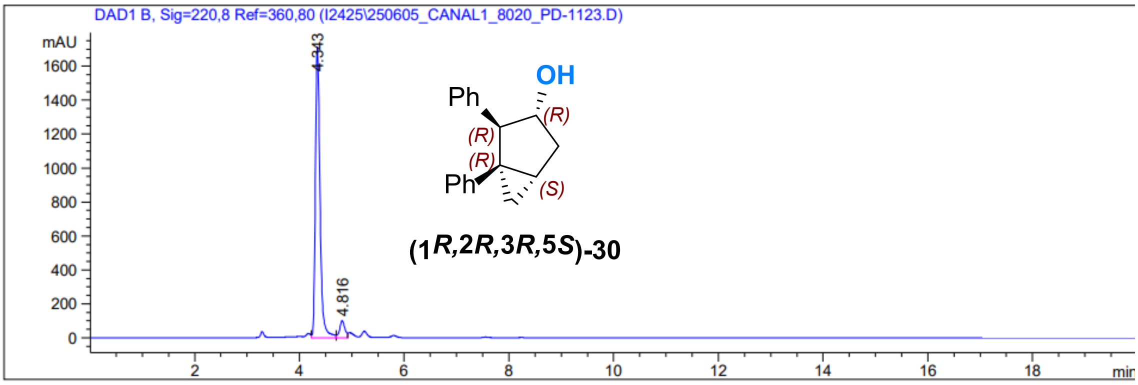

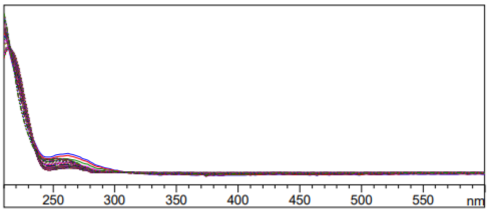

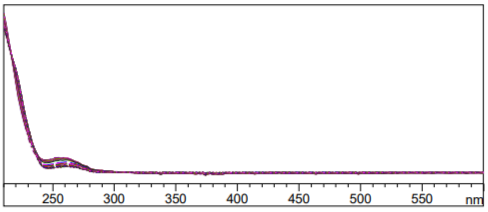

Auto-Scaled Chromatogram

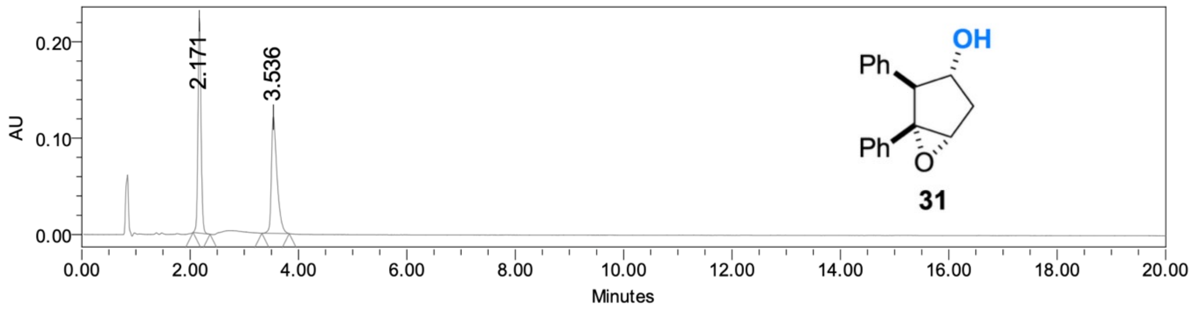

Auto-Scaled Chromatogram

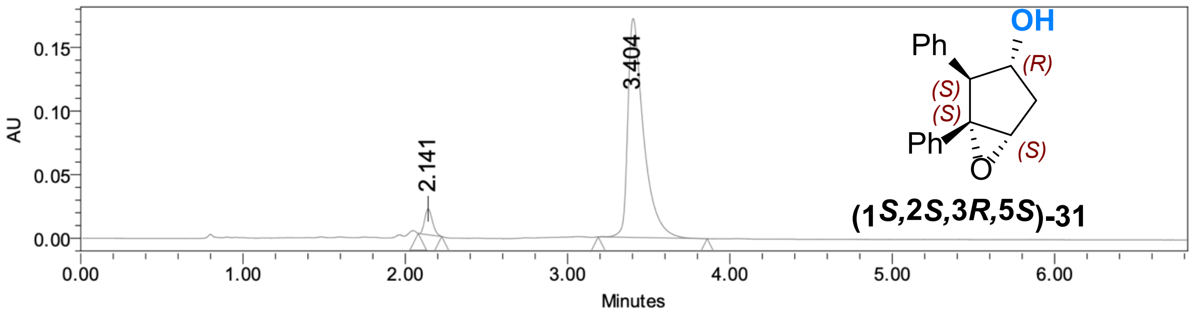

Unknown Peak Results

	Peak Type	RT	Area	% Area	Height
1	Unknown	2.171	940351	50.36	244719
2	Unknown	3.536	926731	49.64	131419

Unknown Peak Results

	Peak Type	RT	Area	% Area	Height
1	Unknown	2.141	73606	5.68	21744
2	Unknown	3.404	1222853	94.32	182641

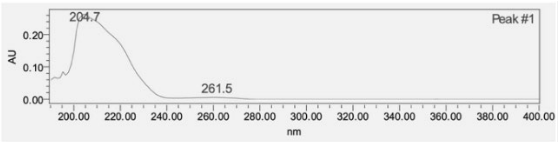

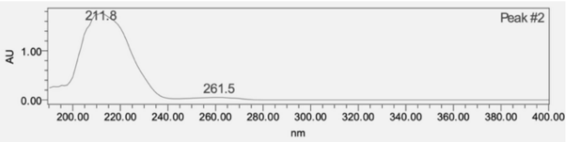

Auto-Scaled Chromatogram

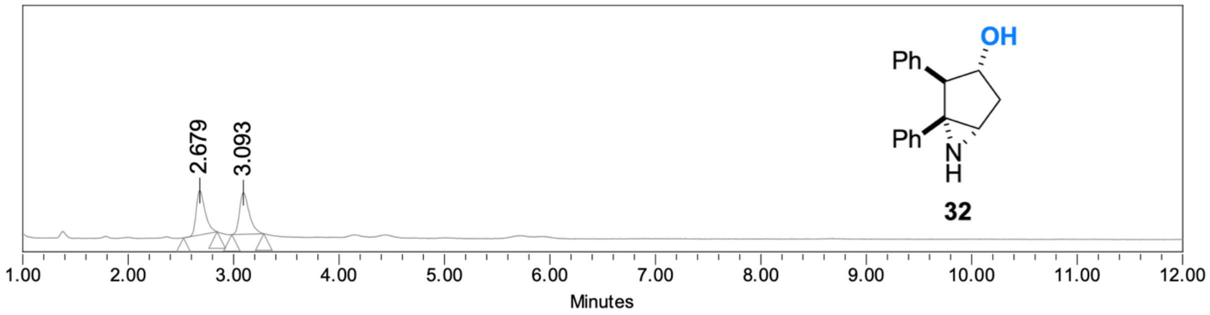

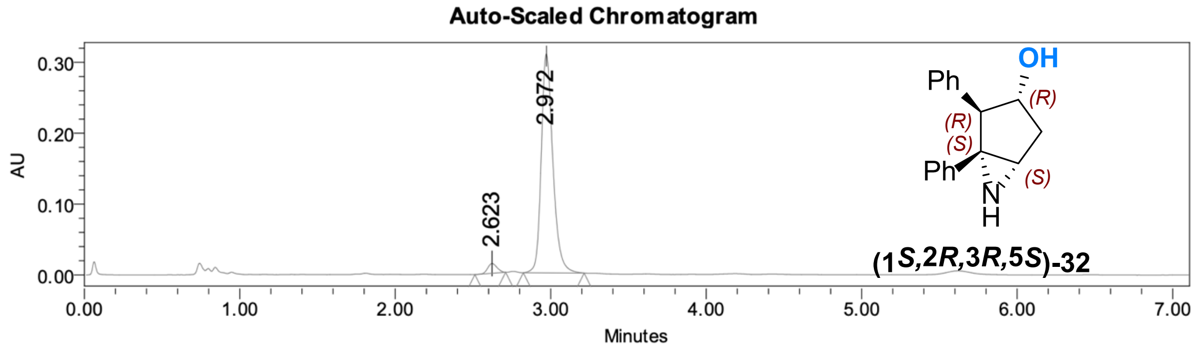

Unknown Peak Results

	Peak Type	RT	Area	% Area	Height
1	Unknown	2.680	109182	50.89	17267
2	Unknown	3.093	105361	49.11	15833

Unknown Peak Results

	Peak Type	RT	Area	% Area	Height
1	Unknown	2.623	49653	3.48	11969
2	Unknown	2.972	1377490	96.52	267345

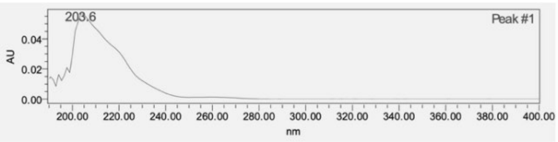

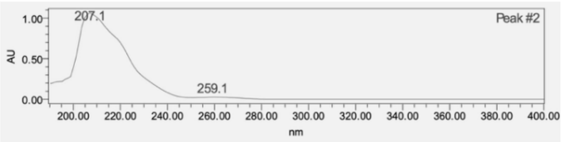

X-Ray Diffraction of compound (1*R*,2*R*)-2,3-diphenylcyclopent-3-en-1-ol (*R,R*)-**2**

CCDC 2471993

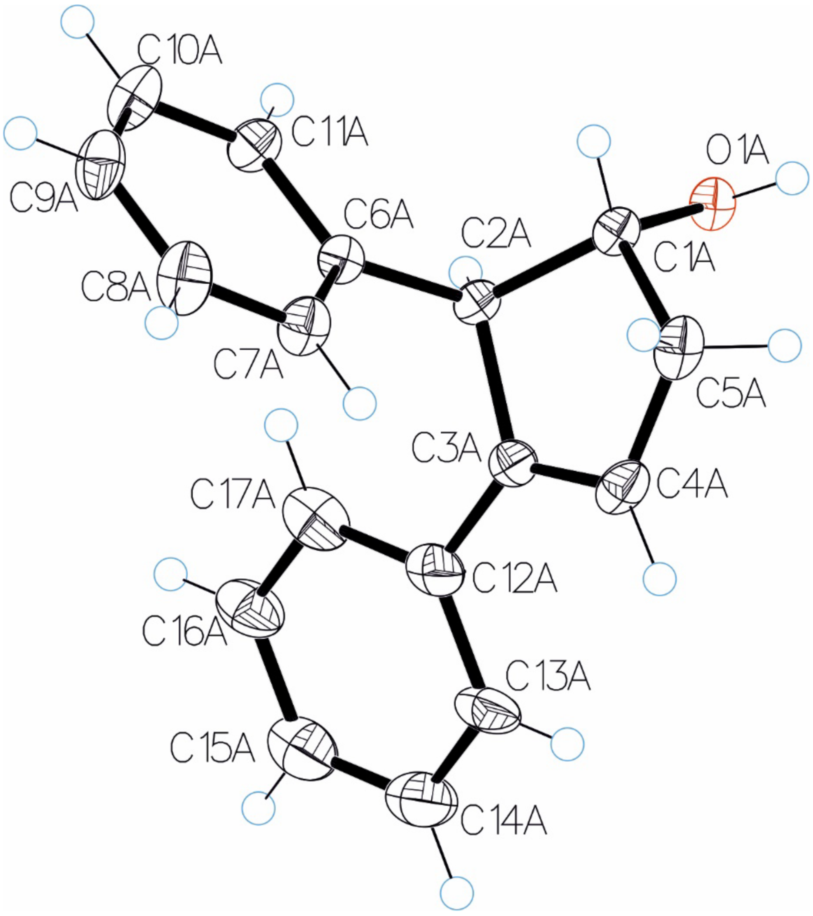

A suitable crystal was selected and analyzed on a **Bruker APEX-II CCD** diffractometer. The crystal was kept at 296.15 K during data collection. Using Olex2, the structure was solved with the SHELXT structure solution program using Intrinsic Phasing and refined with the SHELXL refinement package using Least Squares minimisation.

Crystal Data for $C_{34}H_{32}O_2$ (M =472.59 g/mol): orthorhombic, space group $P2_12_12_1$ (no. 19), a = 5.9024(9) Å, b = 16.421(2) Å, c = 26.539(4) Å, V = 2572.3(7) Å^3, Z = 4, T = 296.15 K, μ(MoKα) = 0.074 mm^{-1}, $Dcalc$ = 1.220 g/cm^3, 26784 reflections measured (2.916° ≤ 2Θ ≤ 59.232°), 6827 unique (R_{int} = 0.0541, R_{sigma} = 0.0640) which were used in all calculations. The final R_1 was 0.0639 (I > 2σ(I)) and wR_2 was 0.1297 (all data).

Table 1 Crystal data and structure refinement for mo_PD1112_0m_a.

Identification code	mo_PD1112_0m_a
Empirical formula	$C_{34}H_{32}O_2$
Formula weight	472.59
Temperature/K	296.15
Crystal system	orthorhombic
Space group	$P2_12_12_1$
a/Å	5.9024(9)
b/Å	16.421(2)
c/Å	26.539(4)
α/°	90
β/°	90
γ/°	90
Volume/Å^3	2572.3(7)
Z	4
ρ_{calc}g/cm^3	1.220
μ/mm^{-1}	0.074
F(000)	1008.0
Crystal size/mm^3	0.2 × 0.1 × 0.05
Radiation	MoKα (λ = 0.71073)
2Θ range for data collection/°	2.916 to 59.232
Index ranges	-8 ≤ h ≤ 7, -17 ≤ k ≤ 22, -36 ≤ l ≤ 27
Reflections collected	26784

Independent reflections	6827 [R_{int} = 0.0541, R_{sigma} = 0.0640]
Data/restraints/parameters	6827/115/363
Goodness-of-fit on F^2	1.139
Final R indexes [I>=2σ (I)]	R_1 = 0.0639, wR_2 = 0.1217
Final R indexes [all data]	R_1 = 0.0903, wR_2 = 0.1297
Largest diff. peak/hole / e Å^{-3}	0.26/-0.31

Table 2 Fractional Atomic Coordinates (×10^4) and Equivalent Isotropic Displacement Parameters (Å^2×10^3) for mo_PD1112_0m_a. U_{eq} is defined as 1/3 of the trace of the orthogonalised U_{IJ} tensor.

**Atom**	***x***	***y***	***z***	**U(eq)**
O1A	5539(4)	2277.5(13)	5635.8(8)	19.1(5)
C1A	6780(5)	1939.0(19)	6050.2(12)	17.6(6)
C2A	5509(5)	1165.3(18)	6219.9(11)	16.5(6)
C3A	6613(6)	509.0(19)	5902.3(12)	20.5(7)
C4A	8598(6)	765(2)	5725.8(12)	26.4(8)
C5A	9099(5)	1626(2)	5881.3(13)	24.7(7)
C6A	5696(5)	1024.0(18)	6785.1(11)	17.8(6)
C7A	7611(6)	676(2)	7003.1(13)	25.1(7)
C8A	7800(6)	605(2)	7523.2(13)	30.7(8)
C1B	9225(5)	4100.7(18)	5341.1(12)	18.4(6)
C2B	8131(5)	4308.4(18)	5855.0(11)	17.4(6)
C3B	9622(5)	3860.4(18)	6229.5(12)	18.9(6)
C4B	11549(5)	3629.2(19)	6010.7(13)	21.3(7)

Table 2 Fractional Atomic Coordinates (×10[4]) and Equivalent Isotropic Displacement Parameters (Å[2]×10[3]) for mo_PD1112_0m_a. U_{eq} is defined as 1/3 of the trace of the orthogonalised U_{IJ} tensor.

**Atom**	***x***	***y***	***z***	**U(eq)**
C5B	11660(5)	3860(2)	5466.3(12)	23.2(7)
C6B	8027(5)	5223.5(19)	5939.7(11)	18.5(6)
C7B	9820(6)	5657(2)	6147.7(12)	25.8(7)
C8B	9718(6)	6496(2)	6203.0(13)	28.0(8)
C9B	7823(6)	6922(2)	6049.8(13)	26.9(8)
C10B	6022(6)	6502(2)	5842.0(15)	32.6(8)
C11B	6133(6)	5663(2)	5786.6(14)	28.8(8)
C12B	8935(6)	3708.6(18)	6757.3(12)	21.3(7)
C13B	10264(7)	3238(2)	7077.7(14)	34.6(9)
C14B	9576(9)	3062(3)	7563.6(15)	47.3(11)
C15B	7532(8)	3357(2)	7741.2(15)	42.6(11)
C16B	6213(7)	3835(3)	7437.3(14)	37.2(9)
C17B	6900(6)	4008(2)	6946.1(13)	27.2(8)
C9A	6068(6)	870(2)	7828.5(13)	30.0(8)
C10A	4144(6)	1210(2)	7619.9(13)	30.3(8)
C11A	3972(6)	1286(2)	7101.5(12)	23.0(7)
O1B	8000(4)	3427.4(13)	5129.4(8)	18.9(5)
C12A	5405(5)	-276.4(15)	5801.4(11)	28.8(8)
C13A	6078(9)	-803(2)	5419.7(13)	32.5(17)
C14A	4757(14)	-1477(2)	5300.8(18)	39.5(19)

Table 2 Fractional Atomic Coordinates (×10[4]) and Equivalent Isotropic Displacement Parameters (Å[2]×10[3]) for mo_PD1112_0m_a. U_{eq} is defined as 1/3 of the trace of the orthogonalised U_{IJ} tensor.

**Atom**	***x***	***y***	***z***	**U(eq)**
C15A	2762(13)	-1624(2)	5564(3)	38.4(19)
C16A	2088(7)	-1097(3)	5945(3)	33.9(16)
C17A	3409(6)	-424(3)	6064.3(16)	32(2)
C13'	6670(50)	-742(14)	5456(9)	45(6)
C14'	5810(40)	-1423(12)	5227(8)	32(5)
C15'	3660(40)	-1631(11)	5396(8)	20(4)
C16'	2550(40)	-1237(11)	5784(9)	23(4)
C17'	3420(30)	-553(14)	6015(8)	23(6)

Table 3 Anisotropic Displacement Parameters (Å[2]×10[3]) for mo_PD1112_0m_a. The Anisotropic displacement factor exponent takes the form: $-2\pi^2[h^2a^{*2}U_{11}+2hka^*b^*U_{12}+\ldots]$.

**Atom**	**U_{11}**	**U_{22}**	**U_{33}**	**U_{23}**	**U_{13}**	**U_{12}**
O1A	12.6(11)	24.8(12)	19.8(11)	3.0(9)	-1.3(9)	0.3(9)
C1A	11.7(14)	23.0(15)	18.1(15)	0.2(12)	-1.5(12)	1.4(12)
C2A	11.2(13)	20.7(15)	17.6(15)	-1.4(12)	-0.5(12)	1.3(12)
C3A	22.3(17)	22.1(15)	17.0(15)	2.6(12)	-3.1(14)	7.8(13)
C4A	21.9(18)	37.5(19)	20.0(17)	4.0(14)	2.0(14)	14.0(15)
C5A	9.1(14)	38.6(19)	26.4(18)	7.5(14)	0.5(13)	2.5(13)
C6A	13.8(14)	20.5(14)	19.3(16)	-0.2(12)	-1.4(13)	-2.0(12)
C7A	16.8(16)	33.1(18)	25.4(18)	2.9(14)	-0.1(14)	1.8(14)

Table 3 Anisotropic Displacement Parameters ($Å^2×10^3$) for mo_PD1112_0m_a. The Anisotropic displacement factor exponent takes the form: $-2π^2[h^2a^{*2}U_{11}+2hka^*b^*U_{12}+…]$.

Atom	U_{11}	U_{22}	U_{33}	U_{23}	U_{13}	U_{12}
C8A	22.6(18)	46(2)	23.7(19)	7.7(16)	-8.0(15)	-2.4(16)
C1B	16.3(15)	20.4(15)	18.5(16)	0.5(12)	2.0(13)	-1.7(12)
C2B	14.2(15)	19.7(15)	18.3(16)	-0.4(12)	1.6(13)	-3.4(12)
C3B	18.7(15)	15.8(14)	22.1(16)	-1.7(12)	-2.8(13)	-2.9(12)
C4B	12.4(15)	24.7(16)	26.9(17)	-1.7(13)	-4.4(13)	1.0(12)
C5B	13.8(15)	31.6(18)	24.4(17)	-2.6(14)	0.6(13)	-6.9(14)
C6B	15.6(15)	26.5(16)	13.5(15)	0.2(12)	2.9(12)	-0.6(13)
C7B	18.0(17)	35.1(18)	24.2(18)	0.6(14)	-3.3(14)	1.8(14)
C8B	29.5(19)	26.1(17)	28.5(19)	-3.9(14)	-3.2(16)	-4.1(15)
C9B	31(2)	21.8(16)	28.2(19)	0.9(14)	5.6(16)	-0.7(14)
C10B	24.2(18)	26.2(17)	47(2)	4.6(16)	-0.2(17)	6.2(15)
C11B	17.5(17)	31.0(18)	38(2)	0.9(15)	-2.5(15)	-3.1(14)
C12B	23.9(17)	20.0(15)	20.1(16)	-1.4(12)	-1.6(13)	-1.8(13)
C13B	34(2)	43(2)	27.3(19)	5.7(16)	0.2(17)	15.1(18)
C14B	57(3)	57(3)	28(2)	15.0(19)	-1(2)	23(2)
C15B	62(3)	43(2)	23(2)	6.9(17)	8.0(19)	10(2)
C16B	39(2)	48(2)	25(2)	-1.1(16)	8.6(17)	5.9(19)
C17B	27.9(18)	32.6(18)	21.2(17)	2.1(14)	0.9(15)	6.2(15)
C9A	32(2)	41(2)	16.9(17)	3.2(14)	-3.4(15)	-7.4(16)
C10A	24.4(18)	45(2)	21.6(18)	-1.7(15)	4.8(15)	-3.1(16)

Table 3 Anisotropic Displacement Parameters (Å^2×10^3) for mo_PD1112_0m_a. The Anisotropic displacement factor exponent takes the form: -2π^2[h^{2}a*^{2}U$_{11}$+2hka*b*U$_{12}$+…].

Atom	U_{11}	U_{22}	U_{33}	U_{23}	U_{13}	U_{12}
C11A	15.4(16)	28.9(17)	24.8(18)	-1.1(13)	0.5(13)	1.7(13)
O1B	14.4(11)	24.9(11)	17.6(11)	-2.7(9)	1.2(9)	-3.2(9)
C12A	41(2)	25.0(17)	20.2(17)	1.6(13)	-10.8(16)	10.6(16)
C13A	50(4)	20(3)	27(3)	-2.4(19)	-12(3)	11(2)
C14A	53(5)	30(3)	36(3)	-5(2)	-6(3)	9(3)
C15A	49(4)	29(3)	37(4)	0(2)	-13(3)	0(2)
C16A	42(3)	24(3)	36(3)	-3(2)	-12(2)	-2(2)
C17A	34(3)	27(3)	36(3)	0(2)	-14(3)	-3(2)
C13'	46(8)	45(8)	45(9)	-1(6)	-18(6)	0(5)
C14'	30(7)	33(7)	32(7)	2(5)	-1(5)	0(5)
C15'	25(7)	18(6)	16(6)	-5(4)	-1(5)	8(5)
C16'	28(6)	14(6)	26(7)	-12(5)	-5(5)	7(5)
C17'	30(8)	12(6)	26(8)	-3(5)	-9(6)	3(5)

Table 4 Bond Lengths for mo_PD1112_0m_a.

Atom	Atom	Length/Å	Atom	Atom	Length/Å
O1A	C1A	1.433(4)	C8B	C9B	1.380(5)
C1A	C2A	1.543(4)	C9B	C10B	1.381(5)
C1A	C5A	1.529(4)	C10B	C11B	1.388(5)
C2A	C3A	1.515(4)	C12B	C13B	1.391(5)

Table 4 Bond Lengths for mo_PD1112_0m_a.

Atom	Atom	Length/Å	Atom	Atom	Length/Å
C2A	C6A	1.522(4)	C12B	C17B	1.392(5)
C3A	C4A	1.331(5)	C13B	C14B	1.382(5)
C3A	C12A	1.498(4)	C14B	C15B	1.383(6)
C4A	C5A	1.502(5)	C15B	C16B	1.368(6)
C6A	C7A	1.392(4)	C16B	C17B	1.394(5)
C6A	C11A	1.388(4)	C9A	C10A	1.382(5)
C7A	C8A	1.390(5)	C10A	C11A	1.385(5)
C8A	C9A	1.375(5)	C12A	C13A	1.3900
C1B	C2B	1.547(4)	C12A	C17A	1.3900
C1B	C5B	1.527(4)	C12A	C13'	1.408(12)
C1B	O1B	1.436(4)	C12A	C17'	1.377(11)
C2B	C3B	1.518(4)	C13A	C14A	1.3900
C2B	C6B	1.521(4)	C14A	C15A	1.3900
C3B	C4B	1.332(4)	C15A	C16A	1.3900
C3B	C12B	1.479(4)	C16A	C17A	1.3900
C4B	C5B	1.495(5)	C13'	C14'	1.37(2)
C6B	C7B	1.390(5)	C14'	C15'	1.384(19)
C6B	C11B	1.391(5)	C15'	C16'	1.384(18)
C7B	C8B	1.387(5)	C16'	C17'	1.38(2)

Table 5 Bond Angles for mo_PD1112_0m_a.

Atom	Atom	Atom	Angle/˚	Atom	Atom	Atom	Angle/˚
O1A	C1A	C2A	107.1(2)	C9B	C8B	C7B	120.4(3)
O1A	C1A	C5A	111.3(3)	C8B	C9B	C10B	119.3(3)
C5A	C1A	C2A	104.1(2)	C9B	C10B	C11B	120.0(3)
C3A	C2A	C1A	102.4(2)	C10B	C11B	C6B	121.5(3)
C3A	C2A	C6A	114.1(2)	C13B	C12B	C3B	121.2(3)
C6A	C2A	C1A	112.2(2)	C13B	C12B	C17B	117.6(3)
C4A	C3A	C2A	110.5(3)	C17B	C12B	C3B	121.2(3)
C4A	C3A	C12A	129.0(3)	C14B	C13B	C12B	121.4(4)
C12A	C3A	C2A	120.5(3)	C13B	C14B	C15B	120.1(4)
C3A	C4A	C5A	112.0(3)	C16B	C15B	C14B	119.8(4)
C4A	C5A	C1A	102.8(3)	C15B	C16B	C17B	120.2(4)
C7A	C6A	C2A	122.1(3)	C12B	C17B	C16B	121.0(3)
C11A	C6A	C2A	119.7(3)	C8A	C9A	C10A	120.2(3)
C11A	C6A	C7A	118.1(3)	C9A	C10A	C11A	119.6(3)
C8A	C7A	C6A	120.9(3)	C10A	C11A	C6A	121.3(3)
C9A	C8A	C7A	119.9(3)	C13A	C12A	C3A	122.0(3)
C5B	C1B	C2B	105.0(2)	C13A	C12A	C17A	120.0
O1B	C1B	C2B	107.7(2)	C17A	C12A	C3A	117.6(3)
O1B	C1B	C5B	111.1(2)	C13'	C12A	C3A	109.3(11)
C3B	C2B	C1B	103.2(3)	C17'	C12A	C3A	127.9(10)
C3B	C2B	C6B	113.9(3)	C17'	C12A	C13'	122.7(15)

Table 5 Bond Angles for mo_PD1112_0m_a.

Atom	Atom	Atom	Angle/˚	Atom	Atom	Atom	Angle/˚
C6B	C2B	C1B	111.4(2)	C12A	C13A	C14A	120.0
C4B	C3B	C2B	110.4(3)	C15A	C14A	C13A	120.0
C4B	C3B	C12B	126.8(3)	C14A	C15A	C16A	120.0
C12B	C3B	C2B	122.9(3)	C17A	C16A	C15A	120.0
C3B	C4B	C5B	112.7(3)	C16A	C17A	C12A	120.0
C4B	C5B	C1B	103.5(3)	C14'	C13'	C12A	122(2)
C7B	C6B	C2B	122.3(3)	C13'	C14'	C15'	113(2)
C7B	C6B	C11B	117.5(3)	C14'	C15'	C16'	124.2(19)
C11B	C6B	C2B	120.1(3)	C17'	C16'	C15'	122.1(18)
C8B	C7B	C6B	121.2(3)	C12A	C17'	C16'	113.8(17)

Table 6 Torsion Angles for mo_PD1112_0m_a.

A	B	C	D	Angle/˚	A	B	C	D	Angle/˚
O1A	C1A	C2A	C3A	90.3(3)	C2B	C6B	C11B	C10B	-177.6(3)
O1A	C1A	C2A	C6A	-146.9(2)	C3B	C2B	C6B	C7B	29.9(4)
O1A	C1A	C5A	C4A	-88.3(3)	C3B	C2B	C6B	C11B	-153.1(3)
C1A	C2A	C3A	C4A	18.7(3)	C3B	C4B	C5B	C1B	-13.2(4)
C1A	C2A	C3A	C12A	-157.9(3)	C3B	C12B	C13B	C14B	-176.9(4)
C1A	C2A	C6A	C7A	-80.0(4)	C3B	C12B	C17B	C16B	177.3(3)
C1A	C2A	C6A	C11A	96.5(3)	C4B	C3B	C12B	C13B	-4.5(5)
C2A	C1A	C5A	C4A	26.8(3)	C4B	C3B	C12B	C17B	177.8(3)

Table 6 Torsion Angles for mo_PD1112_0m_a.

A	B	C	D	Angle/˚	A	B	C	D	Angle/˚
C2A	C3A	C4A	C5A	-1.7(4)	C5B	C1B	C2B	C3B	-21.9(3)
C2A	C3A	C12A	C13A	163.8(3)	C5B	C1B	C2B	C6B	100.7(3)
C2A	C3A	C12A	C17A	-8.8(4)	C6B	C2B	C3B	C4B	-106.2(3)
C2A	C3A	C12A	C13'	173.7(14)	C6B	C2B	C3B	C12B	74.5(4)
C2A	C3A	C12A	C17'	-9.0(14)	C6B	C7B	C8B	C9B	-0.2(5)
C2A	C6A	C7A	C8A	175.6(3)	C7B	C6B	C11B	C10B	-0.5(5)
C2A	C6A	C11A	C10A	-176.2(3)	C7B	C8B	C9B	C10B	0.2(6)
C3A	C2A	C6A	C7A	35.9(4)	C8B	C9B	C10B	C11B	-0.3(6)
C3A	C2A	C6A	C11A	-147.7(3)	C9B	C10B	C11B	C6B	0.5(6)
C3A	C4A	C5A	C1A	-16.3(4)	C11B	C6B	C7B	C8B	0.3(5)
C3A	C12A	C13A	C14A	-172.5(3)	C12B	C3B	C4B	C5B	178.1(3)
C3A	C12A	C17A	C16A	172.8(3)	C12B	C13B	C14B	C15B	-0.1(7)
C3A	C12A	C13'	C14'	-169(2)	C13B	C12B	C17B	C16B	-0.5(5)
C3A	C12A	C17'	C16'	169.9(11)	C13B	C14B	C15B	C16B	-1.2(7)
C4A	C3A	C12A	C13A	-12.1(5)	C14B	C15B	C16B	C17B	1.6(7)
C4A	C3A	C12A	C17A	175.3(3)	C15B	C16B	C17B	C12B	-0.7(6)
C4A	C3A	C12A	C13'	-2.2(15)	C17B	C12B	C13B	C14B	0.9(6)
C4A	C3A	C12A	C17'	175.1(13)	C9A	C10A	C11A	C6A	0.2(5)
C5A	C1A	C2A	C3A	-27.6(3)	C11A	C6A	C7A	C8A	-0.9(5)
C5A	C1A	C2A	C6A	95.1(3)	O1B	C1B	C2B	C3B	96.5(3)
C6A	C2A	C3A	C4A	-102.7(3)	O1B	C1B	C2B	C6B	-140.8(3)

Table 6 Torsion Angles for mo_PD1112_0m_a.

A	B	C	D	Angle/˚	A	B	C	D	Angle/˚
C6A	C2A	C3A	C12A	80.7(4)	O1B	C1B	C5B	C4B	-94.8(3)
C6A	C7A	C8A	C9A	0.8(6)	C12A	C3A	C4A	C5A	174.6(3)
C7A	C6A	C11A	C10A	0.3(5)	C12A	C13A	C14A	C15A	0.0
C7A	C8A	C9A	C10A	-0.2(6)	C12A	C13'	C14'	C15'	-4(3)
C8A	C9A	C10A	C11A	-0.3(6)	C13A	C12A	C17A	C16A	0.0
C1B	C2B	C3B	C4B	14.7(3)	C13A	C14A	C15A	C16A	0.0
C1B	C2B	C3B	C12B	-164.5(3)	C14A	C15A	C16A	C17A	0.0
C1B	C2B	C6B	C7B	-86.4(4)	C15A	C16A	C17A	C12A	0.0
C1B	C2B	C6B	C11B	90.7(4)	C17A	C12A	C13A	C14A	0.0
C2B	C1B	C5B	C4B	21.4(3)	C13'	C12A	C17'	C16'	-13(3)
C2B	C3B	C4B	C5B	-1.1(4)	C13'	C14'	C15'	C16'	-5(3)
C2B	C3B	C12B	C13B	174.6(3)	C14'	C15'	C16'	C17'	5(3)
C2B	C3B	C12B	C17B	-3.1(5)	C15'	C16'	C17'	C12A	4(3)
C2B	C6B	C7B	C8B	177.4(3)	C17'	C12A	C13'	C14'	14(3)

Table 7 Hydrogen Atom Coordinates (Å×10^4) and Isotropic Displacement Parameters (Å^2×10^3) for mo_PD1112_0m_a.

Atom	*x*	*y*	*z*	U(eq)
H1A	6270(50)	2650(20)	5509(11)	29
H1AA	6922.33	2330.92	6326.83	21
H2A	3907.74	1212.39	6126.81	20

Table 7 Hydrogen Atom Coordinates (Å×10[4]) and Isotropic Displacement Parameters (Å[2]×10[3]) for mo_PD1112_0m_a.

**Atom**	***x***	***y***	***z***	**U(eq)**
H4A	9561.54	449.15	5529.33	32
H5AA	9679.79	1942.04	5600.84	30
H5AB	10183.63	1642.3	6155.65	30
H7A	8778.55	489.84	6798.06	30
H8A	9096.52	377.46	7664.5	37
H1B	9189.79	4571.98	5114.73	22
H2B	6594.77	4082.63	5867.07	21
H4B	12700.05	3353.69	6178.5	26
H5BA	12152	3404.28	5261.31	28
H5BB	12687.71	4313.64	5414.56	28
H7B	11111.39	5379.83	6251.73	31
H8B	10935.27	6774.24	6344.27	34
H9B	7759.65	7484.4	6086.05	32
H10B	4734.03	6782.95	5739.04	39
H11B	4914.31	5388.05	5643.99	35
H13B	11643.24	3036.79	6962.51	41
H14B	10489.5	2745.53	7771.4	57
H15B	7055.44	3230.86	8065.92	51
H16B	4855.71	4045.09	7558.78	45
H17B	5982.21	4328.16	6741.47	33

Table 7 Hydrogen Atom Coordinates (Å×10^4) and Isotropic Displacement Parameters (Å^2×10^3) for mo_PD1112_0m_a.

Atom	*x*	*y*	*z*	U(eq)
H9A	6192.68	819.81	8176.48	36
H10A	2969.94	1387.33	7826.45	36
H11A	2675.65	1517.23	6962.74	28
H1BA	8780(50)	3221(16)	4870(14)	28
H13A	7413.44	-704.34	5243.8	39
H14A	5207.91	-1828.93	5045.39	47
H15A	1877.73	-2074.76	5484.14	46
H16A	753.06	-1196	6121.3	41
H17A	2958.57	-71.4	6319.72	39
H13'	8144.51	-581.58	5380.81	54
H14'	6583.96	-1717.25	4982.8	38
H15'	2930.13	-2062.28	5238.43	24
H16'	1158.96	-1440.22	5893.23	27
H17'	2739.37	-302.42	6290.18	27

Table 8 Atomic Occupancy for mo_PD1112_0m_a.

Atom	*Occupancy*	Atom	*Occupancy*	Atom	*Occupancy*
C13A	0.785(19)	H13A	0.785(19)	C14A	0.785(19)
H14A	0.785(19)	C15A	0.785(19)	H15A	0.785(19)
C16A	0.785(19)	H16A	0.785(19)	C17A	0.785(19)

Table 8 Atomic Occupancy for mo_PD1112_0m_a.

Atom	*Occupancy*	Atom	*Occupancy*	Atom	*Occupancy*
H17A	0.785(19)	C13'	0.215(19)	H13'	0.215(19)
C14'	0.215(19)	H14'	0.215(19)	C15'	0.215(19)
H15'	0.215(19)	C16'	0.215(19)	H16'	0.215(19)
C17'	0.215(19)	H17'	0.215(19)		

Computational Details

Geometry optimizations, transition state searches, and energy evaluations were performed with Gaussian 16 package.[4] The quantum mechanics calculations were performed within the framework of Density Functional Theory (DFT)[5] by using the ωB97X-D functional.[6] For Cu, P, K and I, we employed effective core potentials (ECPs) with double-ζ valence basis set (LANL2DZ),[7] supplemented with polarized shells with the following exponents: Cu (f = 3.525), P (d = 0.387), K (d = 1.000) and I (d = 0.289).[8] For all other atoms, the 6-31G(d,p) basis set was used.[9] Solvent effects of THF were included using the implicit solvation model SMD.[10] Free energies were computed at a concentration of 1 M and a temperature of 303.15 K and given in kcal·mol^{-1}.

To quantify the steric hindrance of substrate substituents, we used the distance-weighted volume parameter (V_W),[11] which measures the steric bulkiness of the molecular environment and its impact on the copper center. The descriptor quantifies the bulk produced by substrate substituents considering three parameters: (1) The number of atoms involve (*N*); (2) the size of the atoms (*r* = van der Waals radii in Å); and (3) the distance (*d*) from the atom to the copper center (in Å). The factor r^3 is divided by *d* for each atom and the sum is extended to all the atoms in the given fragment, as given by the following equation:

$$V_W = \sum_{i=1}^{N} \frac{r^3}{d_i}$$

Independent Gradient Model based on Hirshfeld partition (IGMH) analyses [12,13] were performed using the Multiwfn program (version 3.8)[14,15] to evaluate ligand-substrate and intrasubstrate interactions. The isosurfaces were generated at an isovalue of 0.008 a.u. and colored according to sign(λ_2)ρ values in the range of −0.05 to 0.05 a.u.

A data set collection of computational results is available in the ioChem-BD repository[16,17] and can be accessed via http://dx.doi.org/10.19061/iochem-bd-2-89

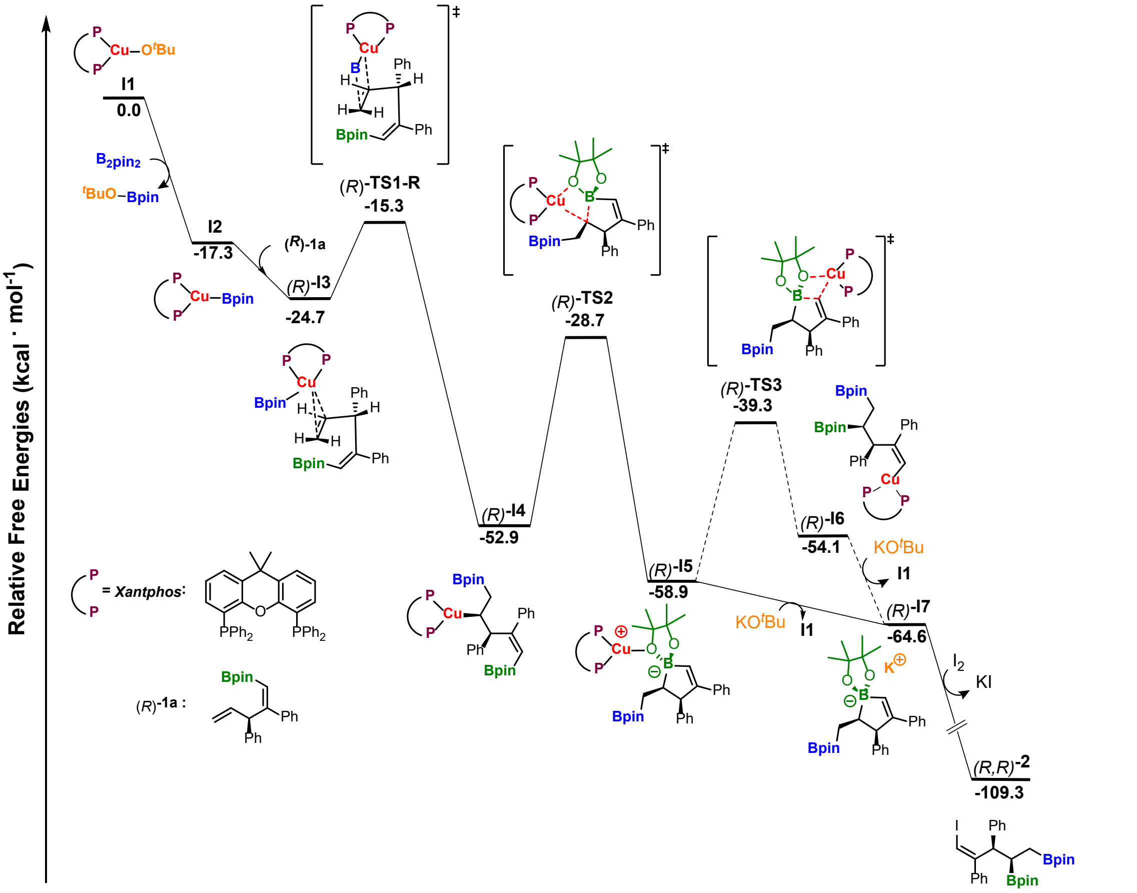

Figure S1. Free-energy profile (kcal·mol^{-1}) for the borylcupration of skipped (*E*)-diene (*R*)-**1a** with B_2pin_2 catalyzed by Cu-Xantphos complex in the presence of KO^{t}Bu base, followed by I_2-iodonolysis.

Conformational analysis of two diastereoisomeric transition states (*R*)-TS1

Table 1. Free-energies (ΔG, in Hartree) and relative free-energy differences (Δ(ΔG) in kcal·mol^{-1}) for the conformational analysis of *(R)*-**TS1-R** and *(R)*-**TS1-S** transition states.

TS	ΔG (Ha)	Δ(ΔG) (kcal·mol^{-1})
**(*R*)-TS1-R**	-3267.31115	0.0
**(*R*)-TS1-R$_A$**	-3267.30268	+5.3
**(*R*)-TS1-R$_B$**	-3267.30056	+6.6
**(*R*)-TS1-R$_C$**	-3267.30392	+4.5
**(*R*)-TS1-R$_D$**	-3267.28643	+15.5
**(*R*)-TS1-R$_E$**	-3267.29294	+11.4
**(*R*)-TS1-R$_F$**	-3267.30134	+6.2
**(R)-TS1-S**	-3267.30927	+1.2
**(*R*)-TS1-S$_A$**	-3267.29906	+7.6
**(*R*)-TS1-S$_B$**	-3267.28681	+15.3
**(*R*)-TS1-S$_C$**	-3267.29977	+7.1

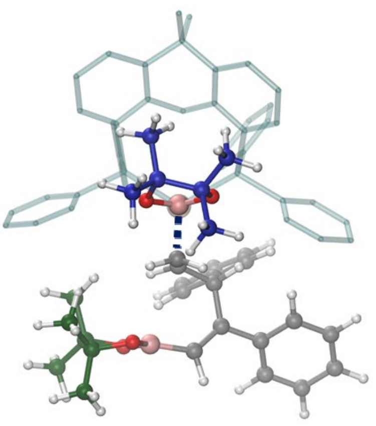

(*R*)-**TS1-R (0.0)**

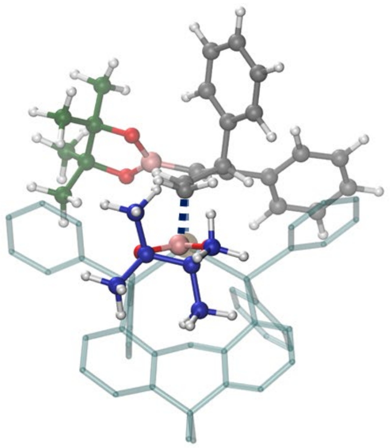

(*R*)-**TS1-R (+1.2)**

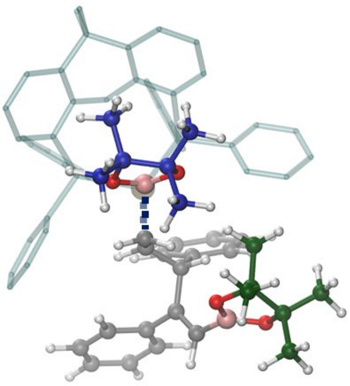

(*R*)-**TS1-R$_A$ (+5.3)**

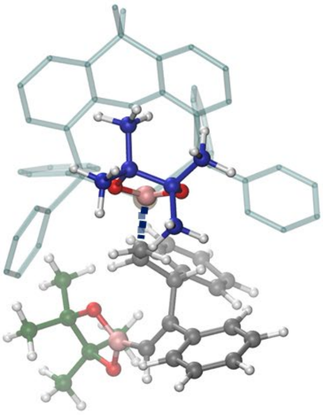

(*R*)-**TS1-R$_B$ (+6.6)**

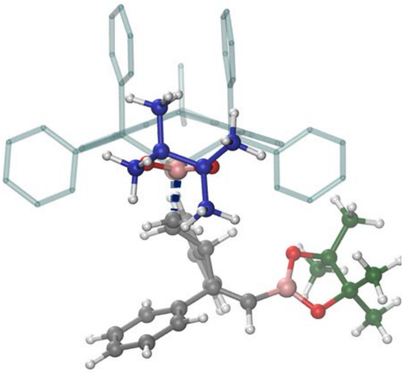

(*R*)-**TS1-R$_C$ (+4.5)**

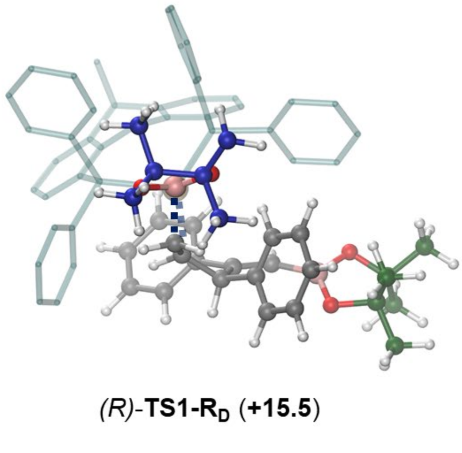

(*R*)-**TS1-R$_D$ (+15.5)**

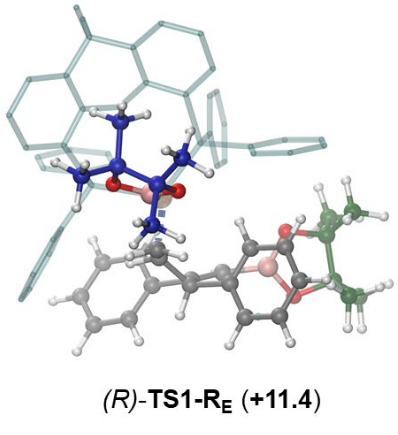

(*R*)-**TS1-R$_E$ (+11.4)**

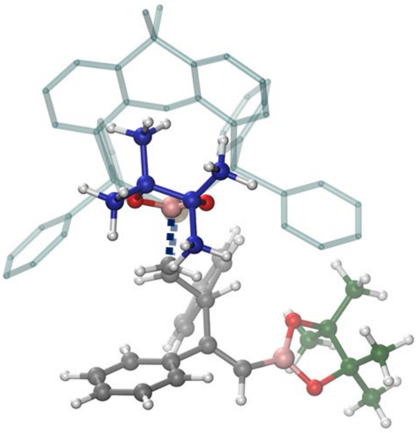

(R)-**TS1-R$_F$** (**+6.2**)

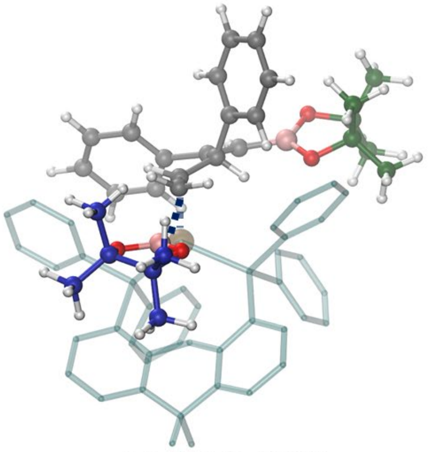

(R)-**TS1-S$_A$** (**+7.6**)

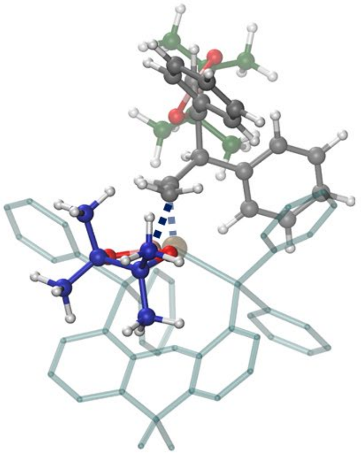

(R)-**TS1-S$_B$** (**+15.3**)

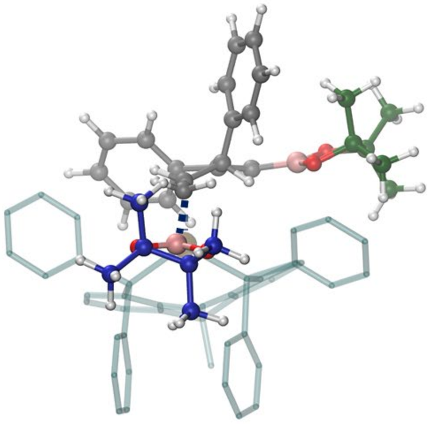

(R)-**TS1-S$_C$** (**+7.1**)

Figure S2. 3D structures resulting from the conformational analysis of (*R)*-**TS1-R** and (*R*)-**TS1-S** transition states. Relative free energy differences in kcal·mol^{-1}.

Evaluation of ligand-substrate and intra-substrate interactions for (*R*)-TS1-R and (*R*)-TS1-S: distance-weighted volume (V_W) and model systems.

We have evaluated the ligand-substrate interactions in transition states (*R*)-**TS1-R** and (*R*)-**TS1-S** using the V_W parameter. The alignment hypothesis follows three steps: the Cu center was placed in the origin, the *z* axis was aligned along the vector bisecting the P-Cu-P angle, and the y,z plane include the Cu and the two P atoms . To estimate the steric effect of the substrate we considered the atoms of both phenyl groups (R_1 and R_2) and the Bpin moiety bonded to the substrate. The value of V_W at the two transition states differs only 0.1. This indicates that ligand-substrate interactions have very similar extension, and therefore, they are not the primary factor explaining the energy difference between the diastereomeric transition states (*R*)-**TS1-R** and (*R*)-**TS1-S**. To confirm these results, we analyzed the free

energy difference between both transition states using a model ligand, XantPH$_2$, where the phenyl groups of the Xantphos were substituted by hydrogen atoms, setting to almost zero the steric effect of the ligand on the reaction center (see Figure S3a). For XantPH$_2$, ligand the free-energy difference between (*R*)-**TS1-R-PH$_2$** and (*R*)-**TS1-S-PH$_2$** (+1.3 kcal·mol^{-1}) remains very similar to that previously computed for real-world Xantphos ligand (+1.2 kcal·mol^{-1}). This further indicates that ligand-substrate interaction cannot explain the computed diastereoselectivity. Figure S3a also shows the non-covalent interactions for the transition states of the XantPH$_2$ ligand, (*R*)-**TS1-R-PH$_2$** and (*R*)-**TS1-S-PH$_2$**, derived from the Independent Gradient Model based on Hirshfeld partition (IGMH) method. The analysis shows non-bonding repulsive intrasubstrate interactions, R$_1$···R$_2$ and R$_1$···Bpin, at the (*R*)-**TS1-S-PH$_2$** structure, similar to that found for in (*R*)-**TS1-S** structure with Xantphos ligand. Finally, Figure S3 illustrates the two different conformations of the substrate (*R*)-**1a** obtained from its geometry at the transition states (*R*)-**TS1-R** and (*R*)-**TS1-S**. The computed free-energy difference between the conformers (+0.8 kcal·mol^{-1}) is consistent with the discussed intrasubstrate interactions.

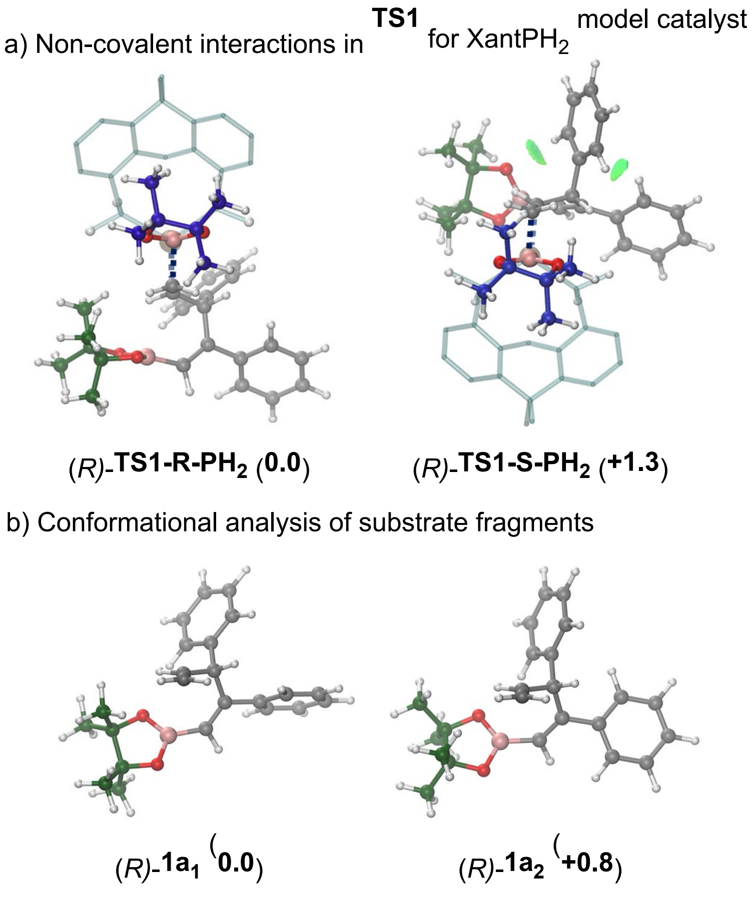

Figure S3. Molecular structures of transition states (*R*)-**TS1-R-PH$_2$** and (*R*)-**TS1-S-PH$_2$** and non-covalent interaction analysis via independent gradient model based on Hirshfeld partition of molecular density, IGMH (a); Molecular structures of two different conformers of the reactant, (*R*)-**1a$_1$** and (*R*)-**1a$_2$**, derived from (*R*)-**TS1-R** and (*R*)-**TS1-S** structures, respectively (b). Relative free-energies in kcal·mol^{-1}.

Cartesian coordinates in Å and electronic energies (E) in Hartree for the most representative structures

I0

E: -1804.528455

C	2.277820	1.132461	-0.605607
C	1.260666	2.054024	-0.359944
C	1.377423	3.417276	-0.619881
C	2.573205	3.855863	-1.187825
C	3.606722	2.961028	-1.454426
C	3.467257	1.608640	-1.156458
H	2.709848	4.906399	-1.420450
H	4.531192	3.322042	-1.893131
H	4.280822	0.921639	-1.364748
C	0.217385	4.316212	-0.194311
C	-1.072228	3.542132	-0.466897
C	-1.063713	2.167016	-0.234676
C	-2.273958	4.111128	-0.884014
C	-2.183934	1.348947	-0.397472
C	-3.414988	3.328946	-1.044387
H	-2.329028	5.175577	-1.083761
C	-3.373731	1.960222	-0.802759
H	-4.343208	3.789910	-1.365826
H	-4.266853	1.362994	-0.950109
O	0.097014	1.557205	0.177125
P	1.920192	-0.633424	-0.242989
P	-1.979191	-0.474198	-0.215782
Cu	-0.066255	-1.357523	-1.267730
C	1.892257	-0.701853	1.593687
C	1.422221	-1.881520	2.180595
C	2.327195	0.343938	2.411221
C	1.410336	-2.021372	3.563916
H	1.062912	-2.692998	1.552123
C	2.298951	0.207620	3.797449
H	2.691233	1.268017	1.971873
C	1.846871	-0.975460	4.374894
H	1.044599	-2.941162	4.008875
H	2.635369	1.027334	4.425044
H	1.826752	-1.081084	5.455282
C	-1.970820	-0.780353	1.599802
C	-2.172087	-2.098772	2.028970
C	-1.725264	0.214748	2.546764
C	-2.154112	-2.408614	3.384098
H	-2.356446	-2.885575	1.301555
C	-1.692679	-0.101654	3.903435
H	-1.569277	1.243120	2.238181
C	-1.912372	-1.409221	4.325172
H	-2.322770	-3.432267	3.704283
H	-1.497587	0.680504	4.630581
H	-1.890439	-1.651073	5.383376
C	-3.609599	-1.142123	-0.728883
C	-3.697248	-1.771417	-1.973618
C	-4.752804	-1.034775	0.073552
C	-4.917731	-2.278283	-2.416641
H	-2.806170	-1.879464	-2.587827
C	-5.968064	-1.544046	-0.370580
H	-4.690532	-0.553538	1.045512
C	-6.051399	-2.164708	-1.617236
H	-4.977618	-2.768726	-3.383258
H	-6.850595	-1.458865	0.256026
H	-7.000791	-2.564003	-1.961115
C	3.484345	-1.486942	-0.682744
C	3.527065	-2.180802	-1.895986
C	4.620457	-1.451292	0.133960
C	4.698314	-2.822348	-2.293401
H	2.638045	-2.229907	-2.520989
C	5.786530	-2.097041	-0.264085
H	4.593065	-0.920956	1.081642
C	5.826875	-2.780563	-1.479065
H	4.723833	-3.361061	-3.235573
H	6.664287	-2.068308	0.374306
H	6.737497	-3.285838	-1.786604
C	0.241410	5.668502	-0.909353
H	-0.581755	6.300548	-0.565755
H	1.164914	6.206707	-0.679818
H	0.164426	5.554895	-1.994925
C	0.328789	4.551390	1.331394
H	1.259164	5.079781	1.563262
H	-0.513483	5.156678	1.681575
H	0.325768	3.606689	1.883849
Cl	-0.114064	-2.720279	-3.105098

I1

E: -2022.505656

C	2.261161	1.412400	-0.818369
C	1.237468	2.355666	-0.715953
C	1.325772	3.651911	-1.217527
C	2.501154	3.993666	-1.886526
C	3.539267	3.074119	-2.020070
C	3.427912	1.794809	-1.482567
H	2.618727	4.987313	-2.305284
H	4.446033	3.360030	-2.543211
H	4.245240	1.089466	-1.591090
C	0.163719	4.601339	-0.926424
C	-1.122363	3.778611	-1.009524
C	-1.083770	2.464166	-0.544061
C	-2.348182	4.256275	-1.469452
C	-2.194137	1.617303	-0.528646
C	-3.482515	3.448365	-1.446310
H	-2.428417	5.270145	-1.845966
C	-3.409044	2.140305	-0.980684
H	-4.430194	3.839983	-1.801202
H	-4.297060	1.517643	-0.988047
O	0.095563	1.947538	-0.069230
P	1.909079	-0.257303	-0.137340
P	-1.956361	-0.152513	-0.066202
Cu	-0.107062	-1.277978	-0.877077
C	1.979640	0.006285	1.683906
C	1.585521	-1.065675	2.492868
C	2.392380	1.197910	2.284092
C	1.622123	-0.953651	3.878217
H	1.250619	-1.993404	2.035274
C	2.413753	1.313498	3.672691
H	2.700635	2.040698	1.672360
C	2.033397	0.238647	4.471194
H	1.314710	-1.792634	4.494459
H	2.733243	2.245609	4.129178
H	2.051256	0.330395	5.552962
C	-1.893337	-0.170891	1.772561
C	-2.017536	-1.414113	2.406980
C	-1.662450	0.968657	2.544416
C	-1.937523	-1.508693	3.791623
H	-2.192080	-2.310929	1.817006
C	-1.567287	0.868300	3.930925
H	-1.562945	1.941675	2.074530
C	-1.710928	-0.366024	4.557109
H	-2.046609	-2.476138	4.272194
H	-1.381892	1.760766	4.520569
H	-1.639296	-0.440008	5.637907
C	-3.597897	-0.885120	-0.444347
C	-3.743117	-1.539514	-1.671420
C	-4.694384	-0.796110	0.420637
C	-4.968366	-2.093621	-2.033321
H	-2.889583	-1.627413	-2.338492
C	-5.915974	-1.357194	0.060762
H	-4.593804	-0.288919	1.375655
C	-6.054241	-2.005072	-1.166172

H	-5.069235	-2.603069	-2.986550
H	-6.761377	-1.288069	0.738491
H	-7.007907	-2.444186	-1.443116
C	3.432180	-1.222201	-0.476882
C	3.324166	-2.291366	-1.373652
C	4.661482	-0.943161	0.132836
C	4.450152	-3.060358	-1.669085
H	2.346473	-2.539519	-1.797065
C	5.779177	-1.714648	-0.166918
H	4.742490	-0.124228	0.842988
C	5.674055	-2.771955	-1.071723
H	4.365933	-3.892350	-2.362355
H	6.731377	-1.495027	0.306628
H	6.547557	-3.374669	-1.302743
O	0.215572	-2.898454	-1.887418
C	-0.066798	-4.136569	-1.336645
C	0.654280	-5.219788	-2.161748
H	0.458930	-6.233626	-1.790204
H	0.330112	-5.168005	-3.207172
H	1.736719	-5.048376	-2.135657
C	-1.582125	-4.419766	-1.353644
H	-2.108158	-3.666487	-0.755198
H	-1.964810	-4.357599	-2.378882
H	-1.831452	-5.410725	-0.952540
C	0.426292	-4.224262	0.125018
H	0.262998	-5.213015	0.572848
H	1.497671	-3.997700	0.172058
H	-0.104908	-3.484011	0.739904
C	0.143992	5.797986	-1.879658
H	-0.678142	6.474400	-1.631790
H	1.065123	6.378754	-1.784241
H	0.035598	5.484190	-2.922278
C	0.317019	5.116166	0.525042
H	1.246753	5.685373	0.625902
H	-0.522582	5.769426	0.783678
H	0.342644	4.290363	1.242738

I2

E: -2200.628971

C	-0.863089	-2.380668	1.021249
C	-1.680605	-1.453601	1.668338
C	-2.247688	-1.664814	2.922832
C	-1.955645	-2.873182	3.555207
C	-1.147846	-3.825438	2.937117
C	-0.609018	-3.587098	1.676026
H	-2.364627	-3.083353	4.537596
H	-0.937917	-4.762047	3.443540
H	0.022546	-4.334183	1.206215
C	-3.189729	-0.588881	3.462770
C	-2.623567	0.765069	3.032311
C	-2.028052	0.849373	1.774155
C	-2.700125	1.939338	3.779128
C	-1.518944	2.032263	1.233625
C	-2.211044	3.140302	3.270475
H	-3.147305	1.926104	4.767109
C	-1.626306	3.189666	2.009412
H	-2.281564	4.045151	3.865380
H	-1.234754	4.129920	1.636915
O	-1.920874	-0.278974	0.998289
P	-0.123776	-1.868826	-0.584175
P	-0.603459	1.928062	-0.365989
Cu	1.048288	0.202596	-0.302933
B	3.047858	0.324321	0.153292
O	3.695243	-0.433073	1.140251
O	4.001020	1.130855	-0.481447
C	5.120290	-0.294881	0.993375
C	5.243935	1.067301	0.242663
C	-1.530768	-1.987081	-1.760919
C	-1.357346	-1.397748	-3.018173
C	-2.741348	-2.617898	-1.463081
C	-2.370012	-1.457018	-3.969308
H	-0.427165	-0.884764	-3.250719
C	-3.761624	-2.660921	-2.410838
H	-2.891513	-3.077977	-0.490754
C	-3.576047	-2.085782	-3.665163
H	-2.224655	-0.995608	-4.940847
H	-4.700849	-3.149125	-2.168420
H	-4.371981	-2.120560	-4.402984
C	-1.916862	1.871159	-1.660605
C	-1.552634	2.209660	-2.970815
C	-3.219594	1.433304	-1.412628
C	-2.480747	2.136324	-4.003885
H	-0.540969	2.545844	-3.183971
C	-4.144447	1.349871	-2.451235
H	-3.528118	1.163663	-0.408248
C	-3.781171	1.706470	-3.746352
H	-2.186122	2.412913	-5.011770
H	-5.153133	1.006607	-2.242546
H	-4.505082	1.643739	-4.553131
C	0.072878	3.626612	-0.577165
C	1.449623	3.814520	-0.422956
C	-0.740881	4.726333	-0.881045
C	2.002641	5.086980	-0.560119
H	2.091241	2.962307	-0.210626
C	-0.185962	5.993969	-1.017851
H	-1.810639	4.588760	-1.011475
C	1.187697	6.175618	-0.855479
H	3.073308	5.223538	-0.441852
H	-0.824017	6.840485	-1.252833
H	1.620377	7.165588	-0.965244
C	0.932877	-3.312122	-1.017179
C	2.265680	-3.286874	-0.587397
C	0.464535	-4.415742	-1.736890
C	3.112736	-4.354986	-0.870283
H	2.639673	-2.429266	-0.031973
C	1.317647	-5.478029	-2.025391
H	-0.566795	-4.447125	-2.075776
C	2.641687	-5.449693	-1.592081
H	4.144977	-4.325891	-0.533845
H	0.946685	-6.328935	-2.588858
H	3.305493	-6.278451	-1.819564
C	-4.569117	-0.774477	2.786255
H	-4.990378	-1.749314	3.052570
H	-5.260055	0.007311	3.117890
H	-4.490768	-0.723310	1.695924
C	-3.363234	-0.684099	4.980011
H	-2.411659	-0.558295	5.505308
H	-4.061637	0.077732	5.336335
H	-3.789255	-1.651913	5.257462
C	5.602428	-1.487919	0.162865
H	6.690777	-1.492243	0.046516
H	5.310534	-2.411151	0.673066
H	5.142774	-1.488554	-0.830121
C	6.403383	1.152463	-0.740821
H	6.411855	2.136192	-1.220523
H	7.360551	1.020092	-0.224786
H	6.322531	0.395954	-1.524378
C	5.274145	2.272839	1.186049
H	6.217150	2.336278	1.738119
H	5.162463	3.187075	0.595205
H	4.449178	2.231122	1.903556
C	5.773459	-0.328584	2.368562
H	5.633626	-1.315074	2.821421
H	6.850116	-0.141587	2.291658
H	5.337753	0.415075	3.039186

(R)-**I3**

E: -3268.476489

Cu	-0.274679	0.128919	-0.684254
C	1.648489	0.764952	-1.630972
H	1.352606	1.799232	-1.772440
H	1.775292	0.170778	-2.532052

C	2.091865	0.317171	-0.426587
P	-0.871159	-1.955203	0.298928
P	-1.014317	1.898312	0.873051
B	-1.558048	0.267184	-2.306060
O	-2.181578	1.422777	-2.791193
O	-2.078674	-0.833804	-2.994806
C	-3.315274	1.052590	-3.596413
C	-2.933966	-0.389063	-4.060863
C	0.181762	1.856856	5.362960
C	0.757293	1.896258	3.022107
C	-1.557005	1.920756	3.685708
C	-1.167593	1.876622	5.023895
C	1.146708	1.869733	4.355894
H	-2.614207	1.946609	3.440079
H	-1.925018	1.861982	5.801831
H	0.482348	1.826097	6.405822
C	-0.762547	-2.342131	2.096821
C	-0.634490	-2.746103	4.860745
C	-1.490799	-3.378435	2.694977
C	0.022086	-1.511561	2.897298
C	0.085199	-1.709711	4.274525
C	-1.420990	-3.583321	4.068893
H	0.587190	-0.705826	2.441008
H	0.697537	-1.050877	4.881360
H	-0.588543	-2.901718	5.934619
C	-2.669556	-2.261341	0.010128
C	-5.437045	-2.486962	-0.382411
C	-3.201212	-3.361774	-0.661636
C	-3.562382	-1.300234	0.481300
C	-4.943870	-1.385399	0.317323
C	-4.574032	-3.467264	-0.864814
H	-2.541108	-4.132090	-1.045602
H	-4.975056	-4.321752	-1.400574
H	-6.503806	-2.593033	-0.546398
C	-2.847497	2.111765	0.867635
C	-5.641132	2.259267	0.668375
C	-3.485070	3.345029	0.709926
C	-3.653218	0.976111	0.950601
C	-5.044456	1.013007	0.847521
C	-4.869842	3.417317	0.610169
H	-2.894914	4.252876	0.645737
H	-5.351683	4.381004	0.479824
H	-6.718888	2.336283	0.575793
C	-0.479725	3.567218	0.298228
C	0.263806	6.058757	-0.740027
C	-0.440186	3.774810	-1.087062
C	-0.154726	4.621832	1.156036
C	0.216069	5.861179	0.637839
C	-0.070778	5.014712	-1.600909
H	-0.725661	2.967807	-1.758019
H	-0.191189	4.481213	2.231878
H	0.468367	6.672723	1.313887
H	-0.043393	5.164475	-2.676163
H	0.557391	7.023770	-1.142206
C	-0.134690	-3.398203	-0.576555
C	0.876299	-5.529205	-2.084830
C	0.344729	-4.547111	0.057239
C	-0.084676	-3.316209	-1.976075
C	0.407240	-4.382285	-2.723934
C	0.856488	-5.603578	-0.694545
H	0.321169	-4.623575	1.139874
H	-0.468811	-2.428987	-2.475069
H	0.426087	-4.315086	-3.807628
H	1.231313	-6.489249	-0.190552
H	1.259803	-6.360416	-2.668989
H	1.516845	1.883301	2.245474
H	-2.120520	-4.021132	2.085740
H	2.202522	1.846604	4.608016
H	-1.987136	-4.390453	4.524194
O	-3.017486	-0.224777	1.140924
C	-5.805695	-0.305693	0.969992

C	-0.598826	1.931759	2.670324
C	-7.196237	-0.222825	0.336150
H	-7.728872	-1.170488	0.450988
H	-7.803224	0.537609	0.834391
H	-7.139357	0.019330	-0.729910
C	-5.953234	-0.646852	2.471659
H	-6.537247	0.128174	2.978867
H	-6.466612	-1.606380	2.592222
H	-4.977670	-0.717349	2.962124
C	-2.103589	-0.408570	-5.346158
H	-1.707633	-1.417460	-5.497537
H	-2.701643	-0.140579	-6.222951
H	-1.255878	0.279633	-5.276536
C	-4.107248	-1.352506	-4.185077
H	-3.745756	-2.335710	-4.502663
H	-4.624129	-1.478377	-3.231170
H	-4.825823	-0.997969	-4.932223
C	-4.540786	1.079286	-2.679878
H	-4.449871	0.331614	-1.887747
H	-4.608714	2.063361	-2.207521
H	-5.467794	0.893370	-3.232342
C	-3.484766	2.067101	-4.718734
H	-2.565492	2.185947	-5.296559
H	-4.288209	1.764029	-5.398831
H	-3.748879	3.042411	-4.297892
C	4.147200	-1.092312	-1.002555
C	4.875811	-0.035922	-1.391315
C	4.662600	-2.477253	-1.215103
H	5.799491	-0.268801	-1.926340
B	4.639122	1.489428	-1.161091
C	3.859054	-3.488808	-1.754910
C	5.979630	-2.791882	-0.858247
O	4.778970	2.089148	0.066262
O	4.467547	2.390834	-2.178167
C	4.366782	-4.769360	-1.952496
H	2.831578	-3.277745	-2.037332
C	6.486027	-4.074014	-1.049124
H	6.602569	-2.023629	-0.409426
C	4.519040	3.502510	-0.091550
C	4.689054	3.714743	-1.642901
C	5.680999	-5.068021	-1.600246
H	3.729365	-5.535941	-2.382436
H	7.508163	-4.298189	-0.758431
C	5.511055	4.273818	0.768187
C	3.101328	3.762301	0.406004
C	6.106975	4.115888	-2.049094
C	3.677822	4.667363	-2.264174
H	6.072610	-6.069993	-1.748196
H	5.390859	5.351371	0.618423
H	5.326357	4.056424	1.824399
H	6.543733	4.001794	0.541396
H	3.023517	3.441306	1.449011
H	2.854218	4.825490	0.361318
H	2.358187	3.215231	-0.177856
H	6.187462	4.078044	-3.139091
H	6.346732	5.131580	-1.722037
H	6.850412	3.429970	-1.631960
H	3.769775	5.666555	-1.826495
H	3.862170	4.748919	-3.339395
H	2.653588	4.318697	-2.118805
H	2.105330	1.006226	0.410542
C	3.075675	-1.371047	1.199751
C	3.563916	-0.421749	2.103029
C	2.902975	-2.685187	1.637251
C	3.829703	-0.774435	3.423651
H	3.765798	0.591823	1.765616
C	3.179594	-3.043053	2.953770
H	2.547517	-3.434409	0.936895
C	3.634470	-2.085367	3.855711
H	4.205375	-0.025211	4.114837
H	3.027897	-4.069086	3.275850

H	3.842487	-2.358120	4.886050
C	2.808433	-1.008341	-0.256106
H	2.167061	-1.791557	-0.673315

(R)-**I4**

E: -3268.523797

Cu	0.067064	-0.092019	-0.036290
P	1.446317	1.709356	0.730411
P	1.301724	-2.118482	0.230422
B	-0.949038	0.246293	-2.910197
O	-0.082589	-0.560994	-3.623149
O	-0.609110	1.580740	-3.031642
C	1.045653	0.241186	-4.024950
C	0.438990	1.686582	-4.021479
C	1.135911	-2.834746	4.802341
C	0.068642	-2.367978	2.691747
C	2.456998	-2.704460	2.783045
C	2.370812	-2.889266	4.162323
C	-0.017892	-2.576360	4.062869
H	3.424076	-2.766284	2.292469
H	3.272801	-3.083573	4.734740
H	1.072384	-2.985662	5.875733
C	1.865705	1.498044	2.507986
C	2.359899	1.103594	5.231703
C	3.088718	1.883622	3.065922
C	0.894437	0.915074	3.325706
C	1.137154	0.726909	4.682663
C	3.335174	1.681359	4.421182
H	-0.056695	0.606263	2.901761
H	0.371358	0.273599	5.304143
H	2.554292	0.946291	6.288450
C	3.096192	2.018870	-0.030674
C	5.604249	2.250481	-1.263516
C	3.511897	3.240232	-0.564651
C	3.975739	0.940307	-0.127024
C	5.236147	1.021832	-0.715231
C	4.750827	3.348545	-1.189755
H	2.858139	4.104363	-0.509897
H	5.057902	4.298766	-1.614564
H	6.569802	2.361829	-1.744562
C	3.031098	-2.430875	-0.329918
C	5.587397	-2.610028	-1.474047
C	3.443013	-3.603203	-0.971005
C	3.950514	-1.382852	-0.256058
C	5.224244	-1.432587	-0.822760
C	4.710388	-3.690244	-1.536361
H	2.762101	-4.443693	-1.050867
H	5.014532	-4.603053	-2.038315
H	6.565349	-2.695459	-1.935067
C	0.429537	-3.576805	-0.470491
C	-0.870697	-5.719092	-1.708481
C	-0.095498	-3.433747	-1.759809
C	0.298287	-4.799233	0.194022
C	-0.352807	-5.865269	-0.423239
C	-0.736847	-4.503690	-2.377686
H	-0.004464	-2.483547	-2.281564
H	0.704872	-4.922150	1.193626
H	-0.454878	-6.810635	0.101159
H	-1.142542	-4.382713	-3.377701
H	-1.381558	-6.549438	-2.186739
C	0.685236	3.380776	0.647589
C	-0.567602	5.867834	0.398868
C	0.797236	4.329787	1.666618
C	-0.056324	3.686903	-0.498414
C	-0.668791	4.930522	-0.626934
C	0.164571	5.564688	1.545454
H	1.363693	4.102364	2.564569
H	-0.175510	2.943671	-1.283030
H	-1.243040	5.157693	-1.519529
H	0.244003	6.291037	2.348759
H	-1.062525	6.829989	0.307702
H	-0.827021	-2.133907	2.121831
H	3.852816	2.341382	2.444528
H	-0.983445	-2.519756	4.556523
H	4.290125	1.978467	4.844382
O	3.541919	-0.250086	0.401293
C	6.130578	-0.215062	-0.645954
C	1.306005	-2.446656	2.037728
C	7.250959	-0.174978	-1.687522
H	7.894535	0.693942	-1.526305
H	7.890723	-1.056927	-1.599258
H	6.855453	-0.131813	-2.707009
C	6.758844	-0.275639	0.766902
H	7.378890	-1.172647	0.864664
H	7.387804	0.604148	0.937237
H	5.991303	-0.303090	1.546275
C	-0.238407	2.058608	-5.340177
H	-0.801210	2.985943	-5.199575
H	0.493707	2.218293	-6.136960
H	-0.939389	1.281795	-5.660092
C	1.417085	2.775656	-3.609836
H	0.916112	3.748576	-3.609299
H	1.822546	2.600308	-2.612275
H	2.251440	2.823295	-4.317187
C	2.132347	0.038930	-2.968632
H	1.781469	0.356106	-1.980938
H	2.379781	-1.025329	-2.910910
H	3.045013	0.591871	-3.209250
C	1.541418	-0.237452	-5.381004
H	0.739773	-0.253198	-6.122214
H	2.344009	0.411612	-5.745716
H	1.941163	-1.251784	-5.289577
C	-4.094295	1.316295	-0.351453
C	-4.991138	0.320716	-0.432316
C	-4.528446	2.724721	-0.579019
H	-6.008482	0.618380	-0.701370
B	-4.812679	-1.223886	-0.287528
C	-3.871246	3.529494	-1.519742
C	-5.591868	3.275822	0.144402
O	-4.682011	-1.902211	0.902527
O	-4.981006	-2.072114	-1.355153
C	-4.280659	4.840761	-1.742223
H	-3.042719	3.118123	-2.091438
C	-5.993775	4.592203	-0.068573
H	-6.093808	2.666001	0.890349
C	-4.555920	-3.310251	0.599721
C	-5.185421	-3.402857	-0.835142
C	-5.340587	5.379092	-1.014225
H	-3.770598	5.445046	-2.487268
H	-6.815305	5.005087	0.509798
C	-5.290274	-4.103541	1.671219
C	-3.069476	-3.658041	0.628404
C	-6.695994	-3.639373	-0.816557
C	-4.509864	-4.403046	-1.760829
H	-5.652842	6.405685	-1.181497
H	-5.286575	-5.170892	1.428053
H	-4.786419	-3.974169	2.633787
H	-6.325040	-3.773554	1.783049
H	-2.661770	-3.398577	1.609726
H	-2.911092	-4.727753	0.468774
H	-2.506363	-3.110067	-0.131385
H	-7.088745	-3.503472	-1.828195
H	-6.940241	-4.653202	-0.486432
H	-7.202509	-2.926807	-0.158420
H	-4.574013	-5.415028	-1.347705
H	-5.009532	-4.401016	-2.734168
H	-3.458180	-4.156490	-1.915659
C	-2.490082	1.331927	1.497816
C	-2.689034	0.266344	2.385193
C	-2.247051	2.598567	2.038539
C	-2.631157	0.460522	3.763104
H	-2.925257	-0.717807	1.994375

C	-2.181733	2.796063	3.416680
H	-2.109561	3.443568	1.370051
C	-2.372220	1.726535	4.287031
H	-2.793454	-0.381183	4.431255
H	-1.979787	3.789823	3.806384
H	-2.321468	1.876852	5.361558
C	-2.603348	1.143887	-0.013673
H	-2.098594	2.006218	-0.460424
C	-2.135578	-0.319571	-2.069337
H	-3.095040	0.109661	-2.400660
H	-2.209892	-1.393208	-2.282820
C	-1.915805	-0.126561	-0.535796
H	-2.379307	-0.986386	-0.036068

(R)-**I5**

E: -3268.532148

Cu	0.779360	0.713588	-0.448338
C	-4.312333	1.592844	-0.340285
H	-4.555271	2.295378	0.466417
H	-4.314169	2.227279	-1.240082
C	-2.901285	1.011552	-0.171289
H	-2.668764	0.489092	-1.108082
P	0.963141	-1.240130	-1.768882
P	2.827303	1.282909	0.636012
B	-5.501364	0.583452	-0.488190
O	-6.820843	0.975275	-0.459530
O	-5.371875	-0.773398	-0.689281
C	-7.637726	-0.210398	-0.399123
C	-6.678201	-1.313104	-0.975613
C	6.371721	1.672906	-2.321034
C	4.008545	1.739301	-1.847338
C	5.572908	1.180892	-0.097102
C	6.624591	1.313450	-0.998051
C	5.063683	1.890977	-2.743410
H	5.778948	0.911835	0.934711
H	7.643762	1.141578	-0.665380
H	7.194245	1.780099	-3.021744
C	2.217615	-0.980959	-3.087239
C	4.046367	-0.430500	-5.130230
C	1.950631	0.023255	-4.027878
C	3.412911	-1.694709	-3.171194
C	4.326677	-1.413126	-4.185883
C	2.853344	0.287645	-5.051042
H	3.636282	-2.478776	-2.454263
H	5.256047	-1.971635	-4.239591
H	4.756694	-0.219662	-5.923766
C	1.461429	-2.805726	-0.934965
C	2.193939	-5.068876	0.545659
C	0.841085	-4.036035	-1.168285
C	2.453982	-2.757759	0.044208
C	2.850115	-3.864897	0.793479
C	1.200312	-5.155534	-0.426484
H	0.064463	-4.116875	-1.921028
H	0.706290	-6.104165	-0.609703
H	2.461229	-5.956530	1.108159
C	3.443515	0.083783	1.892988
C	4.352112	-1.922935	3.626359
C	3.914238	0.408769	3.166926
C	3.472097	-1.260529	1.518396
C	3.925659	-2.283017	2.349177
C	4.351454	-0.590649	4.030014
H	3.938694	1.442993	3.490693
H	4.704967	-0.327407	5.021631
H	4.706486	-2.682844	4.313913
C	2.878612	2.901224	1.511766
C	2.683526	5.423344	2.719971
C	2.149431	3.071335	2.696706
C	3.510380	4.011023	0.942626
C	3.409091	5.264143	1.542762
C	2.059016	4.321139	3.299701
H	1.649991	2.222915	3.155179
H	4.078471	3.906083	0.023719
H	3.900613	6.117429	1.085502
H	1.491225	4.433856	4.217950
H	2.604291	6.401457	3.184144
C	-0.521162	-1.780313	-2.705180
C	-2.907454	-2.585878	-3.918637
C	-0.519631	-2.213137	-4.034043
C	-1.720982	-1.772030	-1.993305
C	-2.912268	-2.168505	-2.589799
C	-1.711774	-2.611830	-4.635528
H	0.404305	-2.239239	-4.603193
H	-1.715978	-1.446909	-0.959624
H	-3.833092	-2.110539	-2.017612
H	-1.705431	-2.942357	-5.669811
H	-3.833975	-2.886339	-4.399208
H	2.988276	1.891822	-2.184549
H	1.024638	0.590416	-3.966618
H	4.858761	2.164366	-3.773117
H	2.629443	1.058918	-5.781674
O	3.059329	-1.544043	0.241250
C	4.011641	-3.693860	1.769994
C	4.255651	1.392051	-0.517576
C	-0.853267	1.405917	1.259990
C	4.003940	-4.766382	2.861997
H	4.100723	-5.763313	2.424262
H	4.855531	-4.639426	3.535190
H	3.083555	-4.732461	3.452321
C	5.331786	-3.799168	0.969137
H	6.188587	-3.653702	1.635047
H	5.413529	-4.787241	0.504614
H	5.381393	-3.043173	0.179316
C	-6.779626	-1.462654	-2.494036
H	-5.987809	-2.130635	-2.845275
H	-7.740775	-1.892231	-2.790708
H	-6.656877	-0.498836	-2.996864
C	-6.797930	-2.674852	-0.307217
H	-6.083937	-3.371362	-0.757691
H	-6.586072	-2.615574	0.762311
H	-7.803604	-3.085280	-0.443871
C	-7.994965	-0.426760	1.071418
H	-7.098526	-0.578165	1.678733
H	-8.506935	0.464583	1.445265
H	-8.659979	-1.285618	1.202903
C	-8.904326	0.018564	-1.211973
H	-8.679048	0.329984	-2.234026
H	-9.508460	-0.893785	-1.248047
H	-9.504721	0.803547	-0.742744
B	-1.678849	2.100018	0.037842
O	-2.092389	3.499666	0.086634
O	-0.663943	2.083986	-1.126006
C	-1.180920	4.303059	-0.627351
C	-0.664118	3.349656	-1.764344
C	-1.912857	5.539523	-1.144405
H	-1.272591	6.136346	-1.804051
H	-2.818052	5.262568	-1.689979
H	-2.210337	6.172126	-0.301415
C	-0.047363	4.742972	0.308221
H	0.629747	5.462559	-0.164075
H	-0.485921	5.215248	1.192586
H	0.538242	3.883804	0.640051
C	0.738571	3.670030	-2.268342
H	1.475149	3.590528	-1.463788
H	1.020983	2.966800	-3.060596
H	0.789704	4.682138	-2.685012
C	-1.634712	3.272608	-2.948427
H	-1.635213	4.189397	-3.547646
H	-1.339660	2.439438	-3.594822
H	-2.653024	3.082948	-2.597228
C	-1.332357	0.173741	1.528285
C	-2.728922	-0.060754	0.951017
H	-2.836365	-1.072596	0.545506

H	0.057283	1.756588	1.747780
C	-0.660573	-0.851970	2.363517
C	0.103887	-0.488726	3.480905
C	-0.720184	-2.208012	2.015468
C	0.793952	-1.444571	4.217854
H	0.141750	0.555467	3.778786
C	-0.027917	-3.166456	2.750570
H	-1.297124	-2.516075	1.147883
C	0.731745	-2.788825	3.854866
H	1.382353	-1.139767	5.078119
H	-0.076561	-4.209474	2.452374
H	1.269905	-3.535429	4.431165
C	-3.714010	0.010051	2.108976
C	-4.419575	-1.127409	2.508029
C	-3.905350	1.193485	2.831625
C	-5.294013	-1.090248	3.593454
H	-4.283451	-2.054144	1.956815
C	-4.789429	1.241577	3.904737
H	-3.351378	2.083819	2.545163
C	-5.486861	0.097835	4.293147
H	-5.828603	-1.989520	3.886984
H	-4.930654	2.173738	4.444543
H	-6.171861	0.133599	5.135193

(R)-**I6**

E: -3268.526756

Cu	-0.637448	0.757925	-0.393460
C	2.923502	1.013858	2.154307
H	3.632030	0.996172	2.996811
H	2.395489	1.970240	2.248222
C	3.729763	0.974157	0.828996
H	4.403761	1.843584	0.852909
P	-2.702513	1.542817	0.633350
P	-1.079567	-1.149820	-1.648639
B	1.908502	-0.153430	2.443586
O	1.955458	-1.404117	1.878457
O	0.937089	-0.054901	3.411449
C	1.138727	-2.286601	2.680240
C	0.179691	-1.287218	3.422013
C	-3.848253	0.311686	-5.064520
C	-2.069527	0.691598	-3.480722
C	-3.145216	-1.466887	-3.591272
C	-3.973664	-0.998325	-4.607724
C	-2.892063	1.154685	-4.503032
H	-3.248732	-2.490046	-3.241742
H	-4.717925	-1.658682	-5.042580
H	-4.497980	0.676705	-5.854243
C	-3.672937	2.361930	-0.695241
C	-4.957726	3.566786	-2.871448
C	-3.066672	3.424434	-1.380111
C	-4.922086	1.903967	-1.119119
C	-5.556512	2.500265	-2.207323
C	-3.711315	4.030531	-2.452391
H	-5.408949	1.081985	-0.602756
H	-6.525463	2.131838	-2.530486
H	-5.457168	4.033203	-3.715209
C	-3.925763	0.510387	1.542828
C	-5.459754	-1.332383	2.995884
C	-4.536156	0.885675	2.742259
C	-4.133278	-0.795472	1.093680
C	-4.887696	-1.738556	1.791242
C	-5.294339	-0.030485	3.462979
H	-4.394279	1.889779	3.128171
H	-5.755530	0.268627	4.398678
H	-6.047908	-2.032718	3.578977
C	-1.934229	-2.630438	-0.966677
C	-3.250147	-4.752361	0.304194
C	-1.468090	-3.938978	-1.110205
C	-3.081373	-2.430793	-0.196076
C	-3.767571	-3.464274	0.440400
C	-2.115289	-4.989425	-0.467692
H	-0.580417	-4.133100	-1.703019
H	-1.736050	-6.000735	-0.572246
H	-3.743193	-5.587254	0.790224
C	0.385817	-1.878999	-2.481721
C	2.721667	-2.882466	-3.643458
C	1.527603	-2.095928	-1.700866
C	0.423484	-2.171125	-3.847867
C	1.589698	-2.667956	-4.425981
C	2.686532	-2.600734	-2.280365
H	1.530377	-1.850212	-0.640775
H	-0.452969	-2.002902	-4.466486
H	1.612998	-2.881468	-5.490462
H	3.568114	-2.719047	-1.662521
H	3.634913	-3.254633	-4.098198
C	-2.339840	2.897593	1.821768
C	-1.639634	4.874098	3.673549
C	-3.140568	4.033725	1.978648
C	-1.183335	2.762729	2.599164
C	-0.839679	3.743073	3.526481
C	-2.789333	5.018400	2.898764
H	-4.037874	4.152462	1.377930
H	-0.541591	1.894434	2.475377
H	0.060426	3.625712	4.122460
H	-3.414378	5.899274	3.010142
H	-1.366499	5.644721	4.388138
H	-1.338091	1.358526	-3.030073
H	-2.088011	3.781319	-1.069577
H	-2.795248	2.178909	-4.848274
H	-3.234773	4.859325	-2.967235
O	-3.535623	-1.140605	-0.093570
C	-5.070976	-3.116994	1.158617
C	-2.188221	-0.623938	-3.018715
C	0.587395	2.301915	-0.348432
C	-5.459313	-4.176511	2.192201
H	-6.408200	-3.918137	2.669477
H	-5.606009	-5.147365	1.711817
H	-4.695862	-4.283722	2.968978
C	-6.190945	-3.016301	0.095504
H	-6.326480	-3.982268	-0.401689
H	-7.135646	-2.731476	0.569871
H	-5.951854	-2.269497	-0.667792
C	-0.131071	-1.652614	4.866622
H	-0.794325	-0.898042	5.299529
H	-0.641125	-2.619894	4.915821
H	0.772980	-1.701238	5.476943
C	-1.120451	-1.033486	2.667222
H	-1.659690	-0.213121	3.147658
H	-0.930314	-0.751322	1.625981
H	-1.767488	-1.915605	2.673106
C	0.438286	-3.272646	1.760175
H	-0.145152	-2.759460	0.996576
H	1.179739	-3.900413	1.256795
H	-0.233358	-3.923975	2.328558
C	2.078134	-3.034395	3.624998
H	2.621058	-2.346599	4.280478
H	1.530004	-3.747338	4.247480
H	2.809482	-3.592122	3.033118
B	4.672561	-0.289917	0.840512
O	5.829547	-0.285164	1.587970
O	4.475685	-1.480637	0.193590
C	6.300016	-1.647275	1.658401
C	5.658047	-2.286795	0.378870
C	7.820436	-1.652562	1.681062
H	8.199809	-2.679070	1.651349
H	8.239291	-1.100840	0.837027
H	8.178020	-1.185768	2.603575
C	5.746591	-2.229440	2.958421
H	6.084039	-3.257006	3.121564
H	6.090153	-1.616528	3.796686
H	4.653778	-2.214310	2.951331
C	5.242310	-3.741812	0.541781

H	6.103258	-4.357776	0.820914
H	4.468729	-3.850693	1.304969
H	4.845720	-4.128326	-0.401954
C	6.517788	-2.120176	-0.873855
H	7.397038	-2.770465	-0.843344
H	5.923744	-2.383387	-1.753693
H	6.851476	-1.085964	-0.994314
C	2.801207	1.117879	-0.392314
H	2.085303	0.288759	-0.354075
H	0.120101	3.296654	-0.263799
C	3.489790	0.989801	-1.749074
C	2.685349	0.844448	-2.886448
C	4.874796	0.994085	-1.926894
C	3.239582	0.684038	-4.150103
H	1.605844	0.841640	-2.763322
C	5.439302	0.838415	-3.193519
H	5.535951	1.133039	-1.076236
C	4.625535	0.677170	-4.310152
H	2.588978	0.556206	-5.010584
H	6.520368	0.843184	-3.302406
H	5.064436	0.550169	-5.295484
C	1.934236	2.378710	-0.306676
C	2.628993	3.685525	-0.077301
C	3.734549	4.115373	-0.827259
C	2.165316	4.538981	0.937397
C	4.336613	5.347767	-0.583269
H	4.119862	3.488407	-1.623428
C	2.764258	5.770626	1.183310
H	1.320645	4.220216	1.542499
C	3.856524	6.182948	0.422829
H	5.184507	5.657381	-1.188273
H	2.380268	6.405875	1.976953
H	4.329378	7.142019	0.613310

(R)-**I7**

E: -1507.219223

C	-1.026601	-0.403791	1.038797
H	-0.528785	-1.238262	1.541478
H	-1.258354	0.324979	1.825958
C	-0.115087	0.190979	0.037048
H	-0.746452	0.302567	-0.792062
B	-2.289245	-0.852338	0.271243
O	-2.923836	-0.067089	-0.661215
O	-2.971775	-2.025513	0.515914
C	-4.235567	-0.622480	-0.872938
C	-4.029831	-2.121218	-0.459540
C	2.304801	0.014498	-0.253629
C	2.069796	1.250384	0.174027
C	-3.509950	-2.993107	-1.603157
H	-3.198810	-3.961549	-1.197566
H	-4.285299	-3.166987	-2.357805
H	-2.643069	-2.533790	-2.091729
C	-5.243406	-2.776265	0.182051
H	-5.012908	-3.816851	0.434208
H	-5.543008	-2.262234	1.098964
H	-6.089907	-2.773287	-0.515843
C	-5.190595	0.125192	0.058751
H	-4.928752	-0.042112	1.109661
H	-5.115336	1.199018	-0.145048
H	-6.229419	-0.185243	-0.097843
C	-4.643989	-0.402860	-2.322788
H	-3.890672	-0.787560	-3.019868
H	-5.602643	-0.900814	-2.529434
H	-4.767524	0.672661	-2.513400
B	0.614057	1.524152	0.295918
O	0.257204	1.983232	1.632599
O	0.240786	2.581040	-0.572919
C	0.007930	3.325678	1.551300
C	-0.511244	3.462316	0.106773
C	-0.985950	3.725717	2.633979
H	-1.306141	4.767570	2.517279
H	-1.869343	3.083088	2.612822
H	-0.522123	3.624713	3.621131
C	1.309170	4.107053	1.757873
H	1.144065	5.188490	1.811374
H	1.765817	3.782530	2.698123
H	2.014992	3.901905	0.947697
C	-0.314845	4.824358	-0.513972
H	0.746442	5.079021	-0.590631
H	-0.740206	4.799882	-1.536341
H	-0.820398	5.609413	0.059787
C	-1.959923	3.040550	-0.017389
H	-2.654678	3.764763	0.419275
H	-2.186112	2.941183	-1.090303
H	-2.106440	2.070140	0.466215
C	1.035099	-0.755479	-0.322754
H	0.900002	-1.233900	-1.309614
H	2.920959	1.900381	0.341536
C	3.585450	-0.589900	-0.630458
C	3.762585	-1.974168	-0.708745
C	4.675741	0.236100	-0.961011
C	4.999448	-2.526442	-1.084681
H	2.937162	-2.636253	-0.464284
C	5.911499	-0.310126	-1.338211
H	4.554404	1.316712	-0.929741
C	6.074387	-1.693873	-1.399093
H	5.116012	-3.606330	-1.129408
H	6.740666	0.347741	-1.588114
H	7.030481	-2.119509	-1.693002
K	0.841557	1.740401	-2.321061
C	1.104390	-1.886669	0.684033
C	0.578694	-3.135882	0.361989
C	1.693228	-1.689524	1.932229
C	0.636602	-4.193866	1.289123
H	0.113164	-3.294183	-0.620493
C	1.757247	-2.743019	2.863829
H	2.111070	-0.706557	2.184617
C	1.227720	-3.990200	2.538075
H	0.218604	-5.177841	1.031576
H	2.223839	-2.582672	3.846710
H	1.275225	-4.814610	3.264380

(*R*)-**1a**

E: -1067.807334

C	1.726421	0.814923	3.286929
H	2.620487	1.424145	3.168951
H	1.498755	0.464071	4.289932
C	0.949169	0.506372	2.252330
H	0.061555	-0.107903	2.387463
C	1.236388	0.929373	0.829193
H	2.124347	1.570199	0.870125
C	0.714767	-1.211285	-0.431713
C	1.612668	-0.318153	0.021589
C	-2.900232	-1.214968	0.677255
C	-3.016847	-1.255335	-0.886057
O	-1.511142	-0.862711	0.871949
O	-1.666143	-1.585094	-1.289746
B	-0.833902	-1.192308	-0.273502
C	-3.104828	-2.579657	1.333615
H	-2.814062	-2.515616	2.385952
H	-4.151636	-2.892687	1.284864
H	-2.488414	-3.347723	0.856866
C	-3.966485	-2.312047	-1.427964
H	-4.983615	-2.132204	-1.065626
H	-3.983093	-2.267505	-2.520823
H	-3.663355	-3.318598	-1.132653
C	-3.335972	0.109495	-1.492726
H	-3.193062	0.060528	-2.575912
H	-4.370860	0.402909	-1.294182
H	-2.671409	0.882092	-1.097408
C	-3.769461	-0.167649	1.356107
H	-4.828220	-0.362104	1.157583

H	-3.614491	-0.206240	2.438454
H	-3.525076	0.839566	1.013355
H	1.111613	-2.032504	-1.030386
C	3.068061	-0.523720	-0.224095
C	3.648092	-1.784602	-0.036518
C	3.884053	0.526771	-0.664559
C	5.000997	-1.991902	-0.288381
H	3.032820	-2.602130	0.327809
C	5.234749	0.318007	-0.926272
H	3.456721	1.512659	-0.827414
C	5.799000	-0.941673	-0.736424
H	5.433817	-2.974792	-0.127422
H	5.847395	1.141790	-1.280172
H	6.854788	-1.102420	-0.932366
C	0.137715	1.777761	0.194136
C	-0.834691	2.412458	0.968905
C	0.146733	2.013762	-1.185258
C	-1.772425	3.261738	0.383940
H	-0.860203	2.244769	2.041246
C	-0.782751	2.867692	-1.771911
H	0.888770	1.521562	-1.808013
C	-1.748560	3.496519	-0.988435
H	-2.522217	3.742143	1.006007
H	-0.754775	3.037777	-2.844154
H	-2.478156	4.158920	-1.444310

(R,R)-**2a**
E: -1490.431879

C	-0.701275	-0.683306	-1.673611
H	-1.031299	0.251689	-2.146444
H	-0.238089	-1.286748	-2.461266
C	0.334733	-0.353155	-0.566976
H	-0.088619	-0.643469	0.400679
B	-1.957526	-1.385426	-1.055215
O	-2.782050	-0.718382	-0.185162
O	-2.334543	-2.687625	-1.249943
C	-1.178115	1.965598	1.009490
B	1.668077	-1.167306	-0.786261
O	2.086120	-2.204717	0.003994
O	2.535977	-0.883131	-1.808301
C	3.270625	-2.768081	-0.614920
C	3.763712	-1.596722	-1.536922
C	4.248139	-3.164605	0.480090
H	5.193659	-3.503146	0.044967
H	4.450022	-2.327865	1.152825
H	3.831506	-3.986274	1.070147
C	2.818662	-3.996517	-1.401548
H	3.667498	-4.502151	-1.870647
H	2.332592	-4.699375	-0.719343
H	2.097888	-3.723999	-2.177887
C	4.377945	-2.043120	-2.854200
H	5.261914	-2.662781	-2.673691
H	3.667418	-2.613200	-3.455771
H	4.689399	-1.167669	-3.431497
C	4.699096	-0.618587	-0.825182
H	5.682901	-1.066409	-0.656936
H	4.828777	0.268569	-1.451593
H	4.294027	-0.294150	0.136620
C	0.667230	1.158455	-0.537628
H	0.955830	1.437300	-1.554701
H	-2.043963	2.583223	1.218955
C	1.844824	1.500478	0.367184
C	2.759280	2.479436	-0.031653
C	2.046872	0.860185	1.594120
C	3.860749	2.793256	0.758972
H	2.613146	2.983426	-0.983731
C	3.147301	1.172615	2.388182
H	1.351265	0.096929	1.928028
C	4.060445	2.138100	1.972282
H	4.567996	3.546084	0.423561
H	3.290787	0.657336	3.333227
H	4.922064	2.378456	2.587785
C	-0.533023	2.010083	-0.156383
C	-1.075066	2.947996	-1.189424
C	-2.186193	2.566312	-1.947434
C	-0.492248	4.198963	-1.414070
C	-2.701455	3.416453	-2.921677
H	-2.636838	1.593034	-1.773156
C	-1.009273	5.047809	-2.389702
H	0.370742	4.512851	-0.833731
C	-2.112953	4.659976	-3.145166
H	-3.565008	3.109271	-3.503823
H	-0.545624	6.015868	-2.555856
H	-2.514960	5.323777	-3.904596
C	-3.917564	-1.566843	0.087045
C	-3.358554	-2.988502	-0.268723
C	-5.043745	-1.105321	-0.836880
H	-5.237429	-0.044570	-0.652122
H	-4.769899	-1.222068	-1.889875
H	-5.967726	-1.660454	-0.651689
C	-4.324162	-1.391163	1.541356
H	-5.121439	-2.093844	1.804306
H	-3.479622	-1.549080	2.215986
H	-4.700043	-0.375888	1.698654
C	-4.378201	-3.931951	-0.885117
H	-3.904550	-4.891566	-1.111875
H	-5.198179	-4.115286	-0.183345
H	-4.793344	-3.526505	-1.810151
C	-2.654822	-3.667519	0.904254
H	-3.363912	-3.944421	1.689509
H	-2.169149	-4.578283	0.544872
H	-1.882869	-3.021836	1.333840
I	-0.694555	0.739920	2.652035

(*R*)-**TS1-R**
E: -3268.462838

Cu	-0.122456	0.137877	-0.193330
C	1.346269	0.681901	-1.547843
H	1.363551	1.729357	-1.843666
H	1.556926	0.006633	-2.379373
C	1.960312	0.356986	-0.277079
P	-0.931309	-2.060830	-0.008896
P	-1.225502	1.635698	1.216307
B	-0.720886	0.613745	-2.129901
O	-1.168119	1.856818	-2.561232
O	-1.115100	-0.364558	-3.029698
C	-2.161693	1.647488	-3.584800
C	-1.729000	0.265801	-4.173483
C	-0.569567	0.690488	5.704463
C	0.270186	1.038693	3.470913
C	-2.096845	1.213548	3.907353
C	-1.866012	0.912250	5.249137
C	0.500856	0.758444	4.812415
H	-3.111165	1.395931	3.564673
H	-2.703065	0.854977	5.938416
H	-0.392473	0.458110	6.750328
C	-1.179555	-2.742480	1.682382
C	-1.511691	-3.667969	4.298974
C	-2.085415	-3.774661	1.956799
C	-0.447379	-2.180664	2.728073
C	-0.609205	-2.643124	4.032063
C	-2.249864	-4.234386	3.259237
H	0.252125	-1.378610	2.519439
H	-0.031276	-2.194068	4.833493
H	-1.644419	-4.025703	5.315790
C	-2.622139	-2.264339	-0.716487
C	-5.250940	-2.338174	-1.687751
C	-2.990506	-3.195480	-1.687086
C	-3.606224	-1.393991	-0.249200
C	-4.925676	-1.411577	-0.696561
C	-4.293261	-3.222619	-2.176834
H	-2.255915	-3.892667	-2.075190

H	-4.565792	-3.942558	-2.941833
H	-6.262452	-2.385205	-2.075911
C	-3.047721	1.847586	0.981309
C	-5.756546	2.087417	0.285949
C	-3.678899	3.095960	0.986522
C	-3.830568	0.735112	0.666927
C	-5.175848	0.821345	0.302498
C	-5.019775	3.214224	0.641093
H	-3.113253	3.985827	1.239535
H	-5.492022	4.191268	0.639954
H	-6.797098	2.202831	0.003272
C	-0.683885	3.394995	1.089164
C	-0.062567	6.104088	0.755536
C	-0.604512	3.950118	-0.194388
C	-0.434211	4.203975	2.200701
C	-0.119301	5.551493	2.032466
C	-0.306123	5.299827	-0.356464
H	-0.798711	3.328086	-1.064944
H	-0.496290	3.791803	3.202850
H	0.073224	6.170226	2.903805
H	-0.257463	5.721931	-1.355977
H	0.173458	7.156264	0.627114
C	-0.000685	-3.374538	-0.906221
C	1.339736	-5.318716	-2.412355
C	0.388280	-4.580538	-0.317863
C	0.313625	-3.137866	-2.252144
C	0.966034	-4.111854	-3.002442
C	1.064013	-5.543258	-1.066535
H	0.163456	-4.776971	0.725791
H	0.015478	-2.200226	-2.714713
H	1.189847	-3.923802	-4.048249
H	1.364668	-6.474439	-0.596037
H	1.851509	-6.076675	-2.997766
H	1.101810	1.049659	2.769564
H	-2.665605	-4.217356	1.151934
H	1.514004	0.570214	5.154517
H	-2.955739	-5.033709	3.464181
O	-3.219565	-0.491389	0.712536
C	-5.919489	-0.475818	-0.011544
C	-1.030831	1.276734	3.009437
C	-7.161312	-0.229442	-0.871667
H	-7.684859	-1.168385	-1.069575
H	-7.871144	0.417602	-0.349810
H	-6.903701	0.234972	-1.828741
C	-6.350904	-1.126954	1.323974
H	-7.036658	-0.464424	1.862128
H	-6.860616	-2.076898	1.132726
H	-5.489077	-1.325317	1.968245
C	-0.652008	0.381673	-5.251302
H	-0.256879	-0.615420	-5.467662
H	-1.051779	0.800436	-6.179692
H	0.178297	1.009652	-4.913851
C	-2.874837	-0.610506	-4.655779
H	-2.481772	-1.562918	-5.024668
H	-3.580059	-0.827304	-3.850679
H	-3.413182	-0.123950	-5.476020
C	-3.521495	1.599908	-2.884250
H	-3.584984	0.742464	-2.209084
H	-3.649099	2.508776	-2.288546
H	-4.344525	1.537275	-3.602881
C	-2.113680	2.808773	-4.565349
H	-1.105977	2.963489	-4.956897
H	-2.793002	2.631514	-5.405569
H	-2.429311	3.728555	-4.063293
C	4.170011	-0.870984	-0.814353
C	4.797662	0.249385	-1.199164
C	4.842484	-2.196489	-0.953566
H	5.774198	0.115726	-1.672626
B	4.391649	1.740786	-0.982742
C	4.164457	-3.313666	-1.455848
C	6.173323	-2.357152	-0.546531
O	4.680476	2.400082	0.190720
O	3.950458	2.589465	-1.967274
C	4.802990	-4.544769	-1.569643
H	3.131535	-3.222147	-1.775904
C	6.812593	-3.588752	-0.655259
H	6.701330	-1.507801	-0.122499
C	4.217397	3.761668	0.064997
C	4.117376	3.943900	-1.494405
C	6.129658	-4.688613	-1.169688
H	4.257657	-5.394336	-1.970534
H	7.843105	-3.691263	-0.327584
C	5.219686	4.680394	0.750011
C	2.869691	3.852114	0.774764
C	5.406343	4.471059	-2.125110
C	2.929309	4.772895	-1.959760
H	6.625321	-5.651379	-1.251929
H	4.942963	5.729007	0.601281
H	5.223688	4.480913	1.825730
H	6.233155	4.528984	0.372985
H	2.982524	3.495524	1.802978
H	2.506478	4.882250	0.807042
H	2.109773	3.240357	0.284997
H	5.322764	4.402259	-3.213431
H	5.585521	5.516951	-1.859713
H	6.272668	3.878966	-1.814883
H	2.976420	5.784199	-1.542889
H	2.939812	4.853313	-3.050858
H	1.983555	4.318254	-1.659917
H	2.271580	1.197226	0.335073
C	3.021700	-1.273670	1.326944
C	3.570819	-0.344246	2.218423
C	2.724740	-2.552507	1.798155
C	3.776146	-0.676845	3.553977
H	3.851980	0.642587	1.857875
C	2.934760	-2.891708	3.133973
H	2.315756	-3.288751	1.111679
C	3.452099	-1.951803	4.020057
H	4.199202	0.058641	4.232991
H	2.683456	-3.889831	3.481301
H	3.613173	-2.210871	5.062510
C	2.793982	-0.900073	-0.132262
H	2.239531	-1.727557	-0.588619

(*R*)-**TS1-S**

E: -3268.460767

Cu	0.196508	-0.023228	0.543432
P	0.859747	-2.009193	-0.451652
P	1.024401	1.846750	-0.606085
B	1.328233	-0.069356	2.283599
O	1.966984	0.985005	2.921032
O	1.800308	-1.280459	2.765590
C	3.094459	0.454206	3.649559
C	2.651996	-1.024121	3.900421
C	0.302655	1.967574	-5.179331
C	-0.214956	1.117951	-2.982584
C	1.499070	2.793744	-3.253325
C	1.270427	2.805943	-4.624964
C	-0.437512	1.123077	-4.358140
H	2.262868	3.439240	-2.828522
H	1.851752	3.464288	-5.263507
H	0.131543	1.971296	-6.251746
C	0.446423	-2.232631	-2.232603
C	-0.385388	-2.539338	-4.887180
C	1.384023	-2.451419	-3.241490
C	-0.910815	-2.156147	-2.567069
C	-1.327988	-2.323433	-3.881041
C	0.967547	-2.593424	-4.565685
H	-1.647539	-1.955711	-1.794988
H	-2.387441	-2.271994	-4.113313
H	-0.705220	-2.658745	-5.918019
C	2.668253	-2.359684	-0.362637

C	5.446803	-2.644221	-0.071813
C	3.213453	-3.540766	0.145748
C	3.556891	-1.345472	-0.720581
C	4.942866	-1.457823	-0.602391
C	4.589240	-3.677551	0.297127
H	2.560175	-4.351054	0.449508
H	4.996643	-4.596162	0.706912
H	6.516567	-2.772667	0.051904
C	2.854198	2.072289	-0.514038
C	5.651273	2.177138	-0.325961
C	3.495642	3.252842	-0.132901
C	3.655882	0.972504	-0.817467
C	5.047097	0.987762	-0.730608
C	4.883244	3.302334	-0.037764
H	2.909140	4.133936	0.104932
H	5.369731	4.223565	0.266543
H	6.730454	2.234516	-0.234962
C	0.426086	3.448291	0.069788
C	-0.468369	5.813852	1.266457
C	0.520191	3.620039	1.459129
C	-0.127208	4.464728	-0.709449
C	-0.568511	5.644079	-0.110291
C	0.078986	4.798528	2.051132
H	0.954907	2.832850	2.071193
H	-0.230509	4.340495	-1.782758
H	-1.001633	6.425474	-0.726506
H	0.160574	4.923319	3.126999
H	-0.817305	6.732170	1.729140
C	0.131549	-3.529446	0.285182
C	-0.990471	-5.787795	1.492731
C	0.091624	-4.750484	-0.398308
C	-0.395368	-3.450471	1.576283
C	-0.953227	-4.576438	2.178566
C	-0.464246	-5.874445	0.204129
H	0.492269	-4.821370	-1.405561
H	-0.351867	-2.508961	2.111768
H	-1.362553	-4.503233	3.181600
H	-0.492683	-6.816789	-0.334410
H	-1.430561	-6.663775	1.959753
H	-0.796735	0.467748	-2.335398
H	2.441646	-2.518108	-3.004145
H	-1.188132	0.463770	-4.782501
H	1.706155	-2.755701	-5.344957
O	3.013048	-0.180296	-1.205157
C	5.792004	-0.291503	-1.106079
C	0.752243	1.950685	-2.421176
C	7.208676	-0.320808	-0.529726
H	7.725534	-1.237866	-0.824847
H	7.799903	0.511238	-0.921271
H	7.202008	-0.260024	0.563195
C	5.873608	-0.378433	-2.648466
H	6.447089	0.466558	-3.043036
H	6.369883	-1.307371	-2.947689
H	4.879074	-0.358324	-3.103676
C	1.789377	-1.188009	5.151940
H	1.350150	-2.190070	5.151065
H	2.376689	-1.071112	6.067722
H	0.972662	-0.459405	5.167314
C	3.784971	-2.039442	3.900210
H	3.381581	-3.041967	4.072730
H	4.313017	-2.049932	2.944122
H	4.502308	-1.819679	4.698081
C	4.312893	0.563660	2.732959
H	4.193100	-0.063896	1.846209
H	4.417185	1.600639	2.401648
H	5.232912	0.268039	3.246845
C	3.305293	1.284268	4.906426
H	2.391550	1.355277	5.500437
H	4.093949	0.848031	5.528315
H	3.612769	2.298112	4.632174
C	-3.254527	1.373087	-0.461968
C	-3.786428	0.308915	-1.096221
C	-3.317163	2.704249	-1.144139
H	-4.200763	0.527497	-2.083575
B	-4.080399	-1.163224	-0.696392
C	-3.049560	2.819183	-2.514408
C	-3.659785	3.868855	-0.443200
O	-4.376437	-1.644963	0.556385
O	-4.270793	-2.111183	-1.674778
C	-3.110519	4.049134	-3.161750
H	-2.767432	1.936160	-3.075712
C	-3.749419	5.095096	-1.094630
H	-3.871482	3.819794	0.619131
C	-3.468180	5.194841	-2.455080
H	-2.874478	4.108227	-4.220387
H	-4.029842	5.979483	-0.529962
H	-3.523433	6.155703	-2.958127
C	-3.542282	1.733614	2.033749
C	-4.771190	1.087625	2.199982
C	-3.172941	2.706063	2.970682
C	-5.598229	1.397185	3.276377
H	-5.071214	0.323776	1.491051
C	-3.998592	3.017975	4.048803
H	-2.230875	3.234250	2.840909
C	-5.217241	2.362706	4.207092
H	-6.548785	0.882198	3.387334
H	-3.690749	3.778529	4.761072
H	-5.865719	2.604539	5.044086
C	-2.569984	1.376070	0.904135
H	-1.853828	2.203704	0.868666
C	-1.772900	0.121007	1.249656
C	-0.809689	0.218328	2.326492
H	-0.791356	-0.585101	3.063637
H	-0.680474	1.198760	2.785529
H	-2.334909	-0.807488	1.216296
C	-4.582409	-3.366655	-1.025279
C	-5.076887	-2.897631	0.386779
C	-3.295407	-4.181327	-0.957431
H	-3.478010	-5.173170	-0.535121
H	-2.890131	-4.305946	-1.965363
H	-2.540264	-3.678808	-0.348272
C	-5.625791	-4.097810	-1.856309
H	-5.192990	-4.385814	-2.818945
H	-5.951242	-5.009164	-1.344759
H	-6.500509	-3.473360	-2.049484
C	-6.569949	-2.571324	0.426944
H	-7.178740	-3.477510	0.360945
H	-6.798891	-2.072261	1.372971
H	-6.854266	-1.899713	-0.388948
C	-4.711440	-3.824148	1.535472
H	-5.088577	-3.410162	2.475221
H	-5.165430	-4.809740	1.391100
H	-3.630454	-3.946057	1.625542

(R)-**TS2**

E: -3268.484473

Cu	0.549829	-0.203698	-0.726048
C	-2.460204	0.518062	-1.973854
H	-2.788012	1.450999	-2.440907
H	-2.075848	-0.084442	-2.804032
C	-1.321068	0.788790	-0.945635
H	-1.488551	0.055935	-0.132596
P	0.351290	-2.548039	-0.098873
P	2.454045	0.621415	0.543970
B	-3.645692	-0.241848	-1.290556
O	-4.548696	0.279491	-0.396168
O	-3.822952	-1.602354	-1.447639
C	-5.118574	-0.821846	0.332505
C	-4.983225	-1.999435	-0.691798
C	6.337025	-1.627161	-0.618672
C	4.225165	-0.603138	-1.165234
C	4.911705	-0.776686	1.139882

C	6.081632	-1.415730	0.734025
C	5.402170	-1.224763	-1.569536
H	4.730303	-0.619476	2.198036
H	6.795861	-1.749111	1.480893
H	7.255080	-2.117151	-0.928762
C	1.920463	-3.326883	0.482045
C	4.375538	-4.451255	1.231443
C	2.863306	-3.692523	-0.487723
C	2.232348	-3.513801	1.830751
C	3.456807	-4.067205	2.201791
C	4.074491	-4.262893	-0.116596
H	1.523275	-3.241282	2.605686
H	3.684287	-4.204602	3.254599
H	5.326681	-4.887329	1.521039
C	-0.675852	-2.665366	1.431485
C	-1.788303	-2.813406	4.001062
C	-1.447097	-3.767937	1.801936
C	-0.537054	-1.637255	2.362156
C	-1.029507	-1.693357	3.662871
C	-2.017142	-3.826678	3.070756
H	-1.585190	-4.589952	1.106693
H	-2.620451	-4.684587	3.349399
H	-2.207095	-2.906347	4.997379
C	2.221396	0.562522	2.373751
C	1.622798	0.505279	5.118994
C	3.108529	1.110064	3.308501
C	1.044345	0.000360	2.869704
C	0.695935	-0.019757	4.222322
C	2.821299	1.060366	4.667288
H	4.023420	1.583262	2.965196
H	3.524260	1.474710	5.382524
H	1.410504	0.504151	6.182840
C	3.051196	2.357598	0.295694
C	3.757939	5.023600	-0.230452
C	2.548269	3.398926	1.081382
C	3.908632	2.672520	-0.764340
C	4.258932	3.994135	-1.023871
C	2.902953	4.720115	0.823672
H	1.860356	3.188866	1.894196
H	4.310108	1.889842	-1.398446
H	4.926342	4.217293	-1.851007
H	2.485442	5.513298	1.436205
H	4.026545	6.055143	-0.436703
C	-0.396325	-3.850963	-1.159547
C	-1.708695	-5.717062	-2.778890
C	-0.007072	-5.195064	-1.145055
C	-1.450422	-3.454423	-1.986430
C	-2.108876	-4.383246	-2.786997
C	-0.657869	-6.120704	-1.956674
H	0.801965	-5.524213	-0.500614
H	-1.784242	-2.423376	-1.983687
H	-2.933417	-4.058484	-3.414056
H	-0.346247	-7.160746	-1.941638
H	-2.214763	-6.442467	-3.408950
H	3.486613	-0.293330	-1.899694
H	2.642819	-3.542958	-1.541846
H	5.584089	-1.400912	-2.625844
H	4.790632	-4.547966	-0.880776
O	0.183041	-0.543908	1.957400
C	-0.709752	-0.515097	4.582766
C	-0.418083	3.190504	-0.812599
C	3.983222	-0.345494	0.190628
C	0.376229	2.843869	-1.831144
C	-1.703790	0.624834	4.261267
H	-2.730240	0.300937	4.461333
H	-1.485841	1.506001	4.873145
H	-1.633128	0.911093	3.208375
C	-0.830804	-0.890371	6.060393
H	-0.619182	-0.025604	6.694873
H	-1.850958	-1.207086	6.292904
H	-0.144515	-1.698589	6.331228
C	-6.152191	-2.079724	-1.673174
H	-5.894956	-2.777183	-2.475868
H	-7.062975	-2.438931	-1.184893
H	-6.358452	-1.104259	-2.123658
C	-4.723240	-3.363282	-0.069732
H	-4.631636	-4.119519	-0.855250
H	-3.799247	-3.366731	0.511364
H	-5.551861	-3.651123	0.585841
C	-4.259224	-1.021234	1.585238
H	-3.225492	-1.268740	1.322655
H	-4.248660	-0.088696	2.155718
H	-4.653798	-1.816126	2.224879
C	-6.544196	-0.469007	0.726495
H	-7.140982	-0.178842	-0.140725
H	-7.030474	-1.316623	1.220446
H	-6.532481	0.373406	1.424453
B	0.315669	1.457956	-2.574741
O	-0.440796	1.365586	-3.758040
O	1.518893	0.693577	-2.760158
C	0.359611	0.783937	-4.784131
C	1.394176	-0.064878	-3.976496
C	-0.532522	-0.025110	-5.717121
H	0.064778	-0.553743	-6.467691
H	-1.129110	-0.755512	-5.165860
H	-1.219723	0.645274	-6.242297
C	1.017229	1.930938	-5.559090
H	1.594642	1.569908	-6.415761
H	0.234648	2.599538	-5.929446
H	1.680340	2.513129	-4.911776
C	2.764778	-0.180611	-4.632704
H	3.263082	0.789210	-4.695858
H	3.404234	-0.857952	-4.057887
H	2.671042	-0.593132	-5.642849
C	0.869787	-1.456631	-3.632894
H	0.809761	-2.098865	-4.516887
H	1.548726	-1.926730	-2.914017
H	-0.120762	-1.395470	-3.175952
C	-1.349856	2.151760	-0.199348
H	-0.937595	1.938533	0.799559
H	1.097075	3.578550	-2.198252
C	-0.366502	4.543500	-0.192171
C	-0.574229	4.725992	1.182876
C	-0.080315	5.676992	-0.963850
C	-0.466764	5.984094	1.766907
H	-0.809102	3.872800	1.811208
C	0.030482	6.936857	-0.383654
H	0.049360	5.567861	-2.036596
C	-0.158956	7.097490	0.987199
H	-0.624816	6.094891	2.835994
H	0.255295	7.797803	-1.006858
H	-0.078962	8.080766	1.441298
C	-2.732877	2.743102	0.037864
C	-3.389872	3.483113	-0.949770
C	-3.380819	2.571819	1.261746
C	-4.662854	4.002901	-0.732072
H	-2.889619	3.667936	-1.897085
C	-4.648440	3.095344	1.493346
H	-2.882507	2.012536	2.048208
C	-5.301047	3.808875	0.490989
H	-5.153272	4.571994	-1.517270
H	-5.125994	2.948411	2.458766
H	-6.290878	4.221348	0.664768

(R)-**TS3**

E: -3268.500474

Cu	-0.811292	0.257095	0.177364
C	3.756564	-0.290415	1.442616
H	4.161726	0.620337	1.905330
H	3.376186	-0.878779	2.291904
C	2.567535	0.070577	0.527514
H	2.031479	-0.856593	0.310686

P	-1.077981	-2.103367	0.103886
P	-3.007159	1.366821	0.288387
B	4.999076	-1.064118	0.872793
O	6.105502	-1.276086	1.665078
O	5.159391	-1.571533	-0.391725
C	7.184215	-1.701128	0.810469
C	6.424106	-2.266774	-0.444357
C	-6.196007	-0.060267	3.341742
C	-3.928377	-0.048518	2.522334
C	-5.624667	1.235554	1.387412
C	-6.570513	0.799314	2.308138
C	-4.875257	-0.483376	3.448283
H	-5.923114	1.903739	0.584266
H	-7.601296	1.128916	2.219985
H	-6.936598	-0.401288	4.058971
C	-1.817779	-2.724604	1.667360
C	-2.849561	-3.536910	4.134828
C	-1.200067	-2.321420	2.859290
C	-2.955624	-3.530034	1.721257
C	-3.472714	-3.928347	2.953217
C	-1.710065	-2.733152	4.085929
H	-3.444303	-3.849699	0.805701
H	-4.362584	-4.549480	2.986312
H	-3.251580	-3.853222	5.092610
C	-2.102544	-2.878696	-1.225992
C	-3.602791	-3.832241	-3.397276
C	-1.741419	-4.062660	-1.877829
C	-3.254108	-2.227258	-1.670838
C	-4.014491	-2.665733	-2.756582
C	-2.485402	-4.533720	-2.952726
H	-0.861922	-4.609931	-1.557637
H	-2.187438	-5.448723	-3.454192
H	-4.160337	-4.208452	-4.248007
C	-3.967872	1.204718	-1.279770
C	-5.500171	0.669721	-3.565323
C	-4.535462	2.249925	-2.010487
C	-4.196213	-0.095858	-1.729182
C	-4.974210	-0.401477	-2.843810
C	-5.279874	1.982033	-3.155541
H	-4.395619	3.276031	-1.688331
H	-5.706078	2.802484	-3.723729
H	-6.101397	0.484846	-4.448764
C	-2.944298	3.189642	0.532250
C	-2.560154	5.934534	0.949870
C	-2.248058	3.964405	-0.406309
C	-3.445366	3.808458	1.680537
C	-3.251718	5.173259	1.887240
C	-2.063078	5.326963	-0.202262
H	-1.844283	3.501124	-1.302052
H	-3.980025	3.228069	2.425607
H	-3.642693	5.639342	2.786631
H	-1.522297	5.911636	-0.940140
H	-2.407965	6.996761	1.114748
C	0.451160	-3.116366	-0.048805
C	2.716753	-4.681932	-0.518453
C	0.692047	-4.255365	0.722830
C	1.363355	-2.759174	-1.049012
C	2.490939	-3.538526	-1.283091
C	1.823383	-5.033608	0.488672
H	-0.007651	-4.549374	1.498460
H	1.187887	-1.865070	-1.643234
H	3.198125	-3.245454	-2.051734
H	2.000393	-5.920596	1.089406
H	3.591689	-5.296539	-0.703412
H	-2.903490	-0.391648	2.598770
H	-0.331568	-1.667909	2.818477
H	-4.575145	-1.157938	4.243816
H	-1.222362	-2.420969	5.004753
O	-3.632835	-1.102777	-0.984601
C	-5.268795	-1.874084	-3.121706
C	-4.291889	0.816721	1.491097

C	0.549092	1.481035	-0.740012
C	-5.697911	-2.112802	-4.571413
H	-5.935075	-3.167354	-4.734416
H	-6.607143	-1.550915	-4.800632
H	-4.917229	-1.817002	-5.278755
C	-6.413702	-2.317754	-2.179383
H	-7.321619	-1.745027	-2.394576
H	-6.628699	-3.381531	-2.324137
H	-6.152014	-2.160445	-1.128584
C	6.123391	-3.760749	-0.345222
H	5.495512	-4.051926	-1.190940
H	7.040547	-4.355546	-0.382711
H	5.586467	-3.998837	0.577470
C	7.093083	-1.966283	-1.778518
H	6.476381	-2.355614	-2.594444
H	7.224806	-0.894238	-1.933007
H	8.073066	-2.451381	-1.833229
C	8.018734	-0.456524	0.505013
H	7.424952	0.302820	-0.011982
H	8.368544	-0.026333	1.447853
H	8.892561	-0.697125	-0.107819
C	8.031737	-2.723299	1.554877
H	7.429459	-3.556777	1.922301
H	8.818420	-3.118757	0.904332
H	8.511587	-2.245932	2.414350
B	1.550520	0.984657	1.341436
O	1.976623	2.202368	1.867597
O	0.556020	0.435490	2.193949
C	1.084006	2.616705	2.902224
C	0.468858	1.254661	3.376442
C	1.881823	3.352369	3.971169
H	1.246603	3.599434	4.828271
H	2.729429	2.759282	4.321168
H	2.271233	4.288105	3.558556
C	0.061700	3.559734	2.274732
H	-0.635174	3.963573	3.014346
H	0.593039	4.394687	1.808695
H	-0.505226	3.048390	1.496225
C	-0.978647	1.350585	3.830072
H	-1.626128	1.706521	3.025992
H	-1.338609	0.369090	4.152727
H	-1.068751	2.039080	4.676909
C	1.308305	0.560009	4.450326
H	1.256633	1.085024	5.408879
H	0.928754	-0.456167	4.596438
H	2.356606	0.488603	4.146124
C	1.742724	1.487781	-1.368863
C	2.939066	0.683940	-0.845493
H	3.138697	-0.144877	-1.539051
H	-0.131475	2.195556	-1.226619
C	1.975249	2.326172	-2.582034
C	1.594945	3.674878	-2.603452
C	2.561633	1.791236	-3.737190
C	1.777220	4.456242	-3.740760
H	1.162859	4.111338	-1.706591
C	2.740857	2.568455	-4.878154
H	2.870980	0.749512	-3.744684
C	2.349755	3.905961	-4.886042
H	1.479616	5.501248	-3.729458
H	3.187779	2.127425	-5.764897
H	2.494707	4.514380	-5.773912
C	4.202521	1.538912	-0.869405
C	5.231316	1.236246	-1.765579
C	4.352250	2.669142	-0.058242
C	6.372108	2.030408	-1.857769
H	5.137448	0.358570	-2.397871
C	5.496569	3.459527	-0.136343
H	3.564139	2.921324	0.643881
C	6.512087	3.147515	-1.037951
H	7.152812	1.773107	-2.568726
H	5.592086	4.328952	0.508685

H 7.400987 3.768506 -1.101948

(*R*)-**TS1-R**$_A$
E: -3268.454277
Cu -0.169186 0.011478 -0.158346
C 1.358021 -0.379407 -1.501069
H 1.069737 -0.280879 -2.545426
H 2.037755 -1.215093 -1.330533
C 1.645992 0.835942 -0.769314
P -0.979992 -0.474829 1.932164
P -1.723344 1.457377 -1.259825
B -0.279044 -1.740619 -1.284239
O -0.934907 -1.862021 -2.502172
O -0.033719 -2.994935 -0.743559
C -1.396471 -3.218545 -2.643163
C -0.417076 -4.002474 -1.705737
C -2.917009 5.298239 1.036456
C -1.410321 3.457755 0.639073
C -3.440637 3.653440 -0.651071
C -3.771435 4.810022 0.048646
C -1.734817 4.623288 1.328185
H -4.106726 3.282087 -1.424870
H -4.695419 5.333108 -0.179358
H -3.176360 6.201603 1.580597
C -1.551034 0.929548 2.982622
C -2.254047 3.140541 4.550472
C -2.891168 1.198577 3.262862
C -0.564386 1.782972 3.491286
C -0.912585 2.871942 4.280809
C -3.239989 2.305781 4.034872
H 0.482666 1.591138 3.274548
H -0.132952 3.517156 4.674314
H -2.527800 3.998159 5.157640
C -2.434182 -1.613519 1.881226
C -4.623119 -3.335655 1.529632
C -2.479168 -2.846568 2.535452
C -3.513040 -1.283714 1.059074
C -4.626314 -2.103874 0.876520
C -3.559561 -3.703763 2.350102
H -1.658575 -3.148685 3.177227
H -3.575747 -4.664640 2.854240
H -5.457287 -4.017849 1.407751
C -3.323090 0.727296 -1.799601
C -5.665024 -0.684350 -2.417430
C -3.876955 0.805108 -3.078267
C -3.995151 -0.043617 -0.851417
C -5.152354 -0.768582 -1.122954
C -5.039638 0.102201 -3.382464
H -3.389451 1.400247 -3.843538
H -5.460199 0.164016 -4.381100
H -6.562418 -1.232504 -2.682867
C -0.993378 2.120548 -2.813021
C 0.379711 2.985917 -5.097143
C -0.651245 1.211101 -3.824364
C -0.635586 3.463433 -2.957201
C 0.047400 3.892236 -4.094257
C 0.025212 1.643935 -4.960271
H -0.906477 0.159831 -3.710284
H -0.881773 4.181048 -2.180794
H 0.318359 4.939147 -4.192741
H 0.282782 0.929824 -5.736828
H 0.913685 3.321556 -5.981094
C 0.164933 -1.353724 3.070703
C 1.940781 -2.751496 4.714504
C 0.016452 -1.320417 4.461929
C 1.205669 -2.097035 2.508993
C 2.085970 -2.798929 3.330829
C 0.904366 -2.011384 5.279892
H -0.792947 -0.751742 4.909207
H 1.312021 -2.129622 1.427503
H 2.889073 -3.382002 2.892377
H 0.784582 -1.974185 6.358340
H 2.634161 -3.291808 5.351997
H -0.499958 2.915240 0.878808
H -3.675135 0.547431 2.891923
H -1.070682 4.990343 2.104361
H -4.287312 2.506906 4.238970
O -3.447494 -0.076777 0.408511
C -5.775215 -1.568457 0.020321
C -2.256062 2.969366 -0.359587
C -6.675074 -2.693644 -0.495368
H -7.128016 -3.237599 0.337664
H -7.499576 -2.287129 -1.086742
H -6.117934 -3.403334 -1.115221
C -6.616710 -0.604518 0.889327
H -7.434050 -0.180037 0.297417
H -7.044720 -1.140545 1.742662
H -6.009063 0.221154 1.272045
C 0.855580 -4.460307 -2.417281
H 1.542617 -4.885895 -1.680829
H 0.644729 -5.229538 -3.166153
H 1.356766 -3.620565 -2.909243
C -1.046753 -5.170866 -0.960586
H -0.295138 -5.650170 -0.325923
H -1.868771 -4.842741 -0.320605
H -1.424932 -5.920416 -1.663775
C -2.846436 -3.251325 -2.160967
H -2.914617 -2.980520 -1.104180
H -3.426119 -2.523218 -2.734917
H -3.296321 -4.239660 -2.298031
C -1.329480 -3.606331 -4.113280
H -0.333763 -3.438131 -4.529252
H -1.593431 -4.660244 -4.249596
H -2.042316 -3.002501 -4.683340
C 4.108720 1.325117 -0.719893
C 5.121790 0.510895 -1.049492
C 4.126850 2.749715 -1.173973
H 5.934151 0.945158 -1.636766
B 5.220715 -1.011322 -0.731463
C 4.815865 3.718242 -0.436119
C 3.461543 3.141709 -2.341596
O 4.524125 -1.962542 -1.435046
O 6.042744 -1.553210 0.223059
C 4.832305 5.048254 -0.847341
H 5.332785 3.423153 0.472500
C 3.475342 4.473119 -2.751775
H 2.933489 2.399905 -2.933285
C 4.710065 -3.223954 -0.754166
C 6.050379 -2.987256 0.025197
C 4.157231 5.430828 -2.004840
H 5.370908 5.786955 -0.260850
H 2.953785 4.758089 -3.660727
H 4.166882 6.468523 -2.325154
H 1.434384 1.761415 -1.300303
C 2.773675 1.697328 1.363761
C 2.204528 2.976027 1.351040
C 3.280698 1.209676 2.570539
C 2.162210 3.750438 2.506362
H 1.797989 3.371739 0.425224
C 3.226970 1.975228 3.735040
H 3.725350 0.217336 2.597155
C 2.676825 3.254227 3.704184
H 1.725037 4.744383 2.471339
H 3.619394 1.571656 4.663995
H 2.642916 3.857555 4.606726
C 2.891430 0.864733 0.098812
H 3.092990 -0.165906 0.417924
C 3.509926 -3.387969 0.172640
H 2.583636 -3.322803 -0.403816
H 3.526544 -4.350127 0.693161
H 3.498180 -2.585748 0.915179
C 6.126550 -3.670586 1.382247

H	6.030761	-4.755266	1.270493
H	7.093863	-3.461512	1.848285
H	5.342929	-3.316681	2.055700
C	7.293142	-3.314941	-0.801383
H	8.177687	-2.940984	-0.278280
H	7.406520	-4.393847	-0.940643
H	7.254100	-2.838816	-1.785804
C	4.753723	-4.343046	-1.781543
H	5.518956	-4.166928	-2.540210
H	4.957602	-5.301507	-1.293528
H	3.786266	-4.416843	-2.285588

(*R*)-**TS1-R$_B$**

E: -3268.453052

Cu	0.390032	0.250529	-0.153837
C	-0.582655	1.460025	-1.528665
H	-0.520124	1.145333	-2.570464
H	-0.531768	2.544171	-1.414599
C	-1.547791	0.754899	-0.700312
P	1.405104	0.675632	1.848838
P	0.635513	-1.874391	-1.073443
B	1.514013	1.444787	-1.454577
O	2.100267	1.013619	-2.635304
O	2.085640	2.642828	-1.046810
C	3.263045	1.823321	-2.890802
C	2.922889	3.135188	-2.112121
C	-1.451073	-5.341942	1.192990
C	-0.471343	-3.138677	1.137604
C	-0.559514	-4.468045	-0.874492
C	-1.187612	-5.490124	-0.169239
C	-1.085152	-4.169010	1.847336
H	-0.357728	-4.590828	-1.935010
H	-1.470054	-6.404750	-0.681898
H	-1.939857	-6.141964	1.740930
C	1.139091	-0.461963	3.279335
C	0.543496	-2.268280	5.339505
C	2.106295	-1.360920	3.736880
C	-0.128506	-0.471511	3.874063
C	-0.421600	-1.363996	4.899810
C	1.807119	-2.262526	4.756267
H	-0.893451	0.219938	3.533296
H	-1.410128	-1.354207	5.348167
H	0.312274	-2.969896	6.135319
C	3.238499	0.672521	1.667511
C	6.003930	0.523950	1.234478
C	4.072326	1.718295	2.063476
C	3.830277	-0.426254	1.040282
C	5.204920	-0.550422	0.844083
C	5.442783	1.651527	1.829302
H	3.648236	2.595401	2.541077
H	6.080663	2.477960	2.125796
H	7.076940	0.486695	1.082024
C	2.338545	-2.521082	-1.370222
C	5.030388	-3.132343	-1.862758
C	2.700217	-3.315491	-2.462087
C	3.357557	-2.113213	-0.504883
C	4.708752	-2.377507	-0.736392
C	4.034250	-3.618027	-2.706980
H	1.935476	-3.669955	-3.145659
H	4.302817	-4.225067	-3.565576
H	6.067321	-3.361576	-2.082847
C	-0.126142	-1.815703	-2.747522
C	-1.382477	-1.315689	-5.206568
C	0.613111	-1.370453	-3.850149
C	-1.509018	-1.986228	-2.888539
C	-2.129994	-1.735232	-4.109610
C	-0.008741	-1.132584	-5.071758
H	1.672702	-1.172569	-3.746435
H	-2.114492	-2.285394	-2.037526
H	-3.204484	-1.857448	-4.200911
H	0.583088	-0.790371	-5.915635
H	-1.870281	-1.121890	-6.157198
C	1.035232	2.327218	2.567977
C	0.404252	4.868926	3.552095
C	1.201169	2.620821	3.926276
C	0.556849	3.320778	1.707794
C	0.246112	4.586888	2.197682
C	0.884445	3.885042	4.415104
H	1.573610	1.859793	4.605652
H	0.448404	3.106392	0.650201
H	-0.124978	5.347565	1.517310
H	1.012400	4.101526	5.471373
H	0.154652	5.853466	3.936345
H	-0.208899	-2.214090	1.643725
H	3.103541	-1.363747	3.310267
H	-1.278015	-4.046531	2.908975
H	2.568876	-2.957547	5.096521
O	2.987868	-1.427164	0.625032
C	5.726690	-1.873974	0.286445
C	-0.203339	-3.279547	-0.226639
C	7.123172	-1.726578	-0.322564
H	7.839394	-1.392558	0.432779
H	7.487962	-2.688376	-0.691790
H	7.127523	-1.010288	-1.150298
C	5.787061	-2.891025	1.450950
H	6.135350	-3.862551	1.085555
H	6.477894	-2.539660	2.224367
H	4.802596	-3.032063	1.907707
C	2.074868	4.112257	-2.927593
H	1.699951	4.896666	-2.263393
H	2.655082	4.585346	-3.725650
H	1.212520	3.608514	-3.375521
C	4.121450	3.849644	-1.506519
H	3.788626	4.751164	-0.982935
H	4.643025	3.214175	-0.787130
H	4.827166	4.151931	-2.287545
C	4.465821	1.079274	-2.309893
H	4.375559	0.981426	-1.225676
H	4.504112	0.073058	-2.738565
H	5.407286	1.589280	-2.536976
C	3.424255	1.991355	-4.394424
H	2.509261	2.367236	-4.857280
H	4.242308	2.683195	-4.621117
H	3.663183	1.025236	-4.850060
C	-3.532115	2.223936	-0.534616
C	-4.657103	1.608898	-0.949323
C	-3.261699	3.635152	-0.935273
H	-5.367833	2.230810	-1.497829
B	-5.116037	0.134366	-0.757205
C	-3.446746	4.042482	-2.262558
C	-2.788664	4.577931	-0.011819
O	-6.435913	-0.172820	-0.528159
O	-4.333049	-0.986214	-0.885703
C	-3.175436	5.350706	-2.654354
H	-3.784618	3.315065	-2.994987
C	-2.529251	5.889083	-0.398992
H	-2.630971	4.288872	1.023700
C	-6.512034	-1.580863	-0.217209
C	-5.190828	-2.146979	-0.855851
C	-2.717473	6.280113	-1.723458
H	-3.313938	5.641419	-3.691603
H	-2.178377	6.607509	0.336430
C	-7.797350	-2.143870	-0.806845
C	-6.543578	-1.691830	1.306355
C	-5.370880	-2.611003	-2.300657
C	-4.502406	-3.242154	-0.051537
H	-2.503613	7.300206	-2.028011
H	-7.851445	-3.225427	-0.646769
H	-8.659102	-1.684930	-0.313398
H	-7.873470	-1.944277	-1.877729
H	-7.402553	-1.130442	1.685049
H	-6.642177	-2.732046	1.630759

H	-5.639394	-1.269650	1.752439
H	-4.396905	-2.893160	-2.708169
H	-6.024117	-3.486121	-2.359214
H	-5.790984	-1.817941	-2.926561
H	-5.138034	-4.131887	0.005542
H	-3.566004	-3.525832	-0.542764
H	-4.265081	-2.914548	0.960460
H	-2.081119	-0.052301	-1.194612
C	-2.924661	0.804601	1.485652
C	-2.766191	-0.569077	1.661295
C	-3.549845	1.532751	2.505498
C	-3.214399	-1.198128	2.821503
H	-2.284555	-1.145906	0.882393
C	-3.993913	0.912042	3.669197
H	-3.688709	2.605033	2.383395
C	-3.827777	-0.463206	3.832027
H	-3.071749	-2.269280	2.935706
H	-4.468869	1.501027	4.448685
H	-4.173911	-0.954297	4.737037
C	-2.409345	1.549600	0.262133
H	-1.779897	2.351660	0.661070

(*R*)-**TS1-R$_C$**

E: -3268.455708

Cu	0.778893	-0.633500	0.196173
C	-0.248085	-2.175800	1.107308
H	0.322026	-2.842362	1.754282
H	-1.016965	-2.714904	0.550867
C	-0.640289	-0.896519	1.683541
P	2.771335	0.259634	1.006455
P	0.161714	0.450387	-1.762231
B	1.053875	-2.626551	-0.426740
O	2.093927	-3.458154	-0.033643
O	0.425030	-3.147172	-1.551649
C	2.261960	-4.492318	-1.027034
C	0.862949	-4.508794	-1.724320
C	3.601711	0.211580	-4.871437
C	1.821739	-0.803822	-3.597459
C	2.278882	1.552043	-3.357124
C	3.319114	1.437999	-4.279406
C	2.848388	-0.910663	-4.527874
H	2.063135	2.521314	-2.919869
H	3.905370	2.315345	-4.535529
H	4.409574	0.127209	-5.592102
C	4.119417	0.435938	-0.233340
C	6.155154	0.498070	-2.146546
C	4.911519	1.576335	-0.379265
C	4.347810	-0.668280	-1.058950
C	5.367839	-0.643265	-2.002110
C	5.922811	1.607191	-1.337887
H	3.719759	-1.548073	-0.952710
H	5.535827	-1.508621	-2.636340
H	6.944003	0.524406	-2.892413
C	2.634794	1.955434	1.708898
C	2.162557	4.565211	2.620350
C	3.015240	2.331868	2.997720
C	2.026744	2.921651	0.908069
C	1.783546	4.229374	1.320506
C	2.771577	3.626158	3.449723
H	3.488624	1.609421	3.654729
H	3.061307	3.907029	4.457132
H	1.985388	5.567431	2.995449
C	-0.320049	2.222889	-1.604341
C	-1.065074	4.859721	-0.984552
C	-1.518551	2.754788	-2.082736
C	0.482025	3.058601	-0.826676
C	0.151324	4.374706	-0.507845
C	-1.890229	4.057854	-1.769259
H	-2.174136	2.138672	-2.686137
H	-2.832560	4.451107	-2.137702
H	-1.375467	5.871921	-0.749201
C	-1.208066	-0.294101	-2.738565
C	-3.224635	-1.570037	-4.197859
C	-1.981586	-1.293838	-2.150584
C	-1.452849	0.063070	-4.071568
C	-2.459849	-0.566052	-4.794855
C	-2.983976	-1.932416	-2.876563
H	-1.783696	-1.592298	-1.129333
H	-0.846265	0.829405	-4.546704
H	-2.642196	-0.282226	-5.826861
H	-3.570644	-2.715792	-2.406038
H	-4.004961	-2.068304	-4.765641
C	3.588463	-0.671009	2.366335
C	4.721309	-2.107320	4.482119
C	4.963855	-0.599288	2.612219
C	2.788837	-1.471464	3.187524
C	3.351492	-2.183462	4.243578
C	5.526493	-1.315747	3.664571
H	5.597716	0.015611	1.979747
H	1.722911	-1.540745	2.998024
H	2.718748	-2.801226	4.873506
H	6.595395	-1.256250	3.846216
H	5.163080	-2.666537	5.301417
H	1.248034	-1.683806	-3.317166
H	4.747335	2.442433	0.255002
H	3.065122	-1.873601	-4.981695
H	6.531424	2.499744	-1.448661
O	1.655485	2.522267	-0.352887
C	1.167100	5.184035	0.298935
C	1.521004	0.433556	-3.011059
C	0.534474	6.410856	0.957950
H	1.284433	6.974872	1.518987
H	0.134286	7.088958	0.199310
H	-0.276904	6.135903	1.638884
C	2.287822	5.652415	-0.660067
H	1.872247	6.302680	-1.436602
H	3.047997	6.212354	-0.105661
H	2.777428	4.804303	-1.148365
C	-0.156259	-5.395262	-1.005995
H	-1.149500	-5.196397	-1.419633
H	0.065704	-6.458342	-1.138214
H	-0.186248	-5.177785	0.066444
C	0.883947	-4.834565	-3.209869
H	-0.133515	-4.789752	-3.609826
H	1.498165	-4.125945	-3.770028
H	1.273417	-5.843696	-3.379761
C	3.390232	-4.059290	-1.964313
H	3.134603	-3.136309	-2.493424
H	4.293852	-3.877377	-1.374842
H	3.615072	-4.834692	-2.702977
C	2.640892	-5.788159	-0.324601
H	1.927631	-6.042230	0.462269
H	2.684132	-6.616546	-1.039488
H	3.628862	-5.684422	0.134079
C	-3.087123	-1.452381	1.998594
C	-4.265897	-1.675823	1.380993
C	-2.710325	-2.281313	3.179453
H	-4.897104	-2.458942	1.804569
B	-4.940916	-0.810723	0.283791
C	-2.216257	-1.688897	4.349213
C	-2.833113	-3.674356	3.138420
O	-6.285171	-0.954003	0.031407
O	-4.369444	0.196297	-0.452625
C	-1.870728	-2.467485	5.448647
H	-2.109524	-0.609556	4.399211
C	-2.478608	-4.456143	4.235584
H	-3.192221	-4.148285	2.229025
C	-6.618503	-0.082420	-1.069788
C	-5.445337	0.957655	-1.044102
C	-1.997191	-3.855210	5.395651
H	-1.498091	-1.988765	6.349543
H	-2.573762	-5.536690	4.180017

C	-7.997683	0.513752	-0.829765
C	-6.631072	-0.942726	-2.333051
C	-5.701032	2.143165	-0.113112
C	-5.021694	1.456030	-2.416652
H	-1.719199	-4.462533	6.251876
H	-8.236839	1.247404	-1.606285
H	-8.753798	-0.276114	-0.865400
H	-8.061829	1.002649	0.144435
H	-7.335753	-1.767670	-2.194366
H	-6.944381	-0.366248	-3.208169
H	-5.642743	-1.364988	-2.531882
H	-4.764429	2.691892	0.020104
H	-6.448998	2.824353	-0.529892
H	-6.040083	1.812957	0.873284
H	-5.881300	1.873734	-2.950869
H	-4.280758	2.250503	-2.305388
H	-4.584945	0.656070	-3.018561
H	-0.229586	-0.701590	2.673135
C	-2.294833	0.978584	2.135897
C	-3.501306	1.347700	2.738881
C	-1.287885	1.946675	2.043157
C	-3.699589	2.638218	3.226696
H	-4.300179	0.618830	2.831316
C	-1.483996	3.236952	2.524102
H	-0.344375	1.671348	1.583459
C	-2.692342	3.592628	3.119058
H	-4.647740	2.894522	3.691485
H	-0.680806	3.962475	2.438311
H	-2.844200	4.599290	3.497867
C	-2.074957	-0.405576	1.520338
H	-2.288288	-0.266244	0.456303

(*R*)-**TS1-R$_D$**

E: -3268.439562

Cu	-0.450350	-0.635241	-0.288327
C	0.259189	-1.878920	-1.817462
H	-0.564064	-2.248260	-2.425846
H	1.041515	-2.623617	-1.667121
C	0.675823	-0.520677	-2.080330
P	-2.684975	-0.113159	-1.171502
P	-0.181333	0.294524	1.840445
B	-0.566051	-2.761544	-0.170467
O	-1.553482	-3.611726	-0.649653
O	0.251036	-3.440576	0.728605
C	-3.164136	-1.661321	4.825341
C	-1.395589	-1.826594	3.189922
C	-2.266760	0.334832	3.802589
C	-3.163949	-0.278439	4.675846
C	-2.269599	-2.433771	4.085883
H	-2.268249	1.416640	3.720678
H	-3.857712	0.332113	5.245741
H	-3.857347	-2.136331	5.512846
C	-4.148858	-0.815422	-0.291194
C	-6.304362	-1.890541	1.130180
C	-5.464123	-0.544367	-0.688883
C	-3.928043	-1.629379	0.818026
C	-5.000451	-2.163804	1.530226
C	-6.535115	-1.081743	0.016160
H	-2.909624	-1.846450	1.118220
H	-4.809605	-2.791546	2.395534
H	-7.142585	-2.304623	1.682841
C	-3.254442	1.631862	-1.363522
C	-4.039270	4.325170	-1.373798
C	-3.917538	2.173370	-2.466951
C	-3.023235	2.473762	-0.277184
C	-3.402894	3.814251	-0.244186
C	-4.298456	3.511209	-2.473867
H	-4.132854	1.551354	-3.328957
H	-4.805948	3.922297	-3.340730
H	-4.348294	5.364513	-1.399422
C	-0.437559	2.112281	2.098097
C	-0.931150	4.866870	2.348487
C	0.414150	2.925391	2.851830
C	-1.547135	2.722836	1.510152
C	-1.833428	4.083853	1.631827
C	0.180845	4.291873	2.957080
H	1.275509	2.496436	3.349561
H	0.863978	4.910821	3.529589
H	-1.100673	5.932763	2.454278
C	1.447281	0.048326	2.683267
C	3.993388	-0.262669	3.821298
C	2.565834	0.676343	2.120968
C	1.622652	-0.733887	3.827395
C	2.890055	-0.895508	4.385733
C	3.826462	0.532243	2.689248
H	2.455951	1.279845	1.223443
H	0.776154	-1.221664	4.297398
H	3.007586	-1.512580	5.271494
H	4.669499	1.037988	2.230062
H	4.978573	-0.385598	4.261590
C	-2.842504	-0.810108	-2.865668
C	-2.769865	-2.018587	-5.394208
C	-2.238178	-0.159508	-3.949750
C	-3.399674	-2.077703	-3.062636
C	-3.363337	-2.676416	-4.319516
C	-2.208652	-0.757122	-5.206086
H	-1.785943	0.819245	-3.812060
H	-3.845700	-2.609462	-2.228786
H	-3.798536	-3.661888	-4.456510
H	-1.740742	-0.237223	-6.036798
H	-2.742664	-2.487160	-6.373366
H	-0.724970	-2.437446	2.589889
H	-5.652601	0.090658	-1.550207
H	-2.261157	-3.514370	4.196774
H	-7.551792	-0.867423	-0.299654
O	-2.399635	1.910923	0.810571
C	-3.148355	4.593070	1.043294
C	-1.378450	-0.432491	3.047341
C	-3.128973	6.104883	0.806916
H	-4.088355	6.442531	0.406584
H	-2.978552	6.641428	1.747353
H	-2.339225	6.398168	0.107861
C	-4.280480	4.255886	2.043365
H	-4.107979	4.768563	2.995378
H	-5.246309	4.579080	1.641750
H	-4.334643	3.180359	2.237211
C	2.376196	1.324135	-1.786637
C	3.444616	1.848794	-1.155849
C	1.278458	2.270504	-2.165542
H	3.346119	2.914606	-0.932304
B	4.866284	1.355454	-0.770383
C	0.808469	2.325786	-3.484025
C	0.727825	3.149572	-1.227681
O	5.682036	0.497413	-1.456171
O	5.523842	1.984209	0.269501
C	-0.183334	3.230420	-3.851797
H	1.223001	1.655747	-4.232150
C	-0.260948	4.057603	-1.595570
H	1.066358	3.110410	-0.196126
C	6.917294	0.378227	-0.713072
C	6.932315	1.701377	0.124920
C	-0.725645	4.099116	-2.907053
H	-0.531367	3.257175	-4.880550
H	-0.675364	4.725780	-0.847370
C	8.065235	0.231561	-1.700121
C	6.810892	-0.871474	0.156521
C	7.545029	2.883697	-0.626566
C	7.561907	1.578904	1.504463
H	-1.506206	4.798891	-3.188601
H	9.024552	0.222569	-1.172879
H	7.965302	-0.714286	-2.240603
H	8.075708	1.041855	-2.431951

H	6.614949	-1.733567	-0.483913
H	7.738980	-1.050101	0.707543
H	5.985904	-0.792224	0.870908
H	7.342535	3.802989	-0.069780
H	8.628547	2.774838	-0.728780
H	7.109849	2.989042	-1.625012
H	8.617912	1.303526	1.419047
H	7.502872	2.540232	2.023377
H	7.057392	0.828881	2.117258
H	0.043023	-0.008142	-2.795950
C	3.058057	-1.179918	-1.632392
C	2.972863	-1.510234	-0.279352
C	3.860603	-1.974018	-2.454356
C	3.665006	-2.596634	0.244668
H	2.311705	-0.940341	0.358520
C	4.547467	-3.072340	-1.942545
H	3.933249	-1.741335	-3.513994
C	4.451115	-3.389722	-0.588589
H	3.569267	-2.832058	1.300593
H	5.156138	-3.683547	-2.603171
H	4.983683	-4.247575	-0.187836
C	2.155193	-0.121809	-2.232405
H	2.384178	-0.115337	-3.312411
C	-1.474810	-4.872920	0.038498
C	-0.015897	-4.853351	0.609732
C	-2.551991	-4.875697	1.122256
H	-3.525299	-4.698713	0.656221
H	-2.590072	-5.833942	1.649445
H	-2.381173	-4.080682	1.851590
C	-1.753226	-5.988992	-0.960035
H	-1.618337	-6.970534	-0.493573
H	-2.788184	-5.919327	-1.308787
H	-1.098843	-5.922748	-1.831612
C	0.146030	-5.498357	1.978049
H	-0.115200	-6.561042	1.940029
H	1.187818	-5.416328	2.302564
H	-0.481666	-5.012869	2.729277
C	1.020104	-5.419782	-0.362365
H	2.021247	-5.193975	0.014928
H	0.924869	-6.504651	-0.468663
H	0.927500	-4.965065	-1.353421

(*R*)-**TS1-R$_E$**

E: -3268.444925

Cu	-0.342842	0.055081	-0.684316
C	0.085127	0.625761	-2.632490
H	-0.729147	1.072053	-3.205916
H	0.664390	-0.094618	-3.208199
C	0.813214	1.487866	-1.725325
P	0.000540	-1.579576	0.909005
P	-2.067169	1.678703	0.016486
B	-1.415766	-0.872098	-2.200992
O	-2.745177	-0.519242	-2.405813
O	-1.132539	-2.049703	-2.879520
C	-3.430224	-1.648239	-2.982014
C	-2.265042	-2.389893	-3.708921
C	-1.558036	3.775874	4.123654
C	-0.834074	2.191887	2.458442
C	-2.766871	3.568274	2.043315
C	-2.602112	4.171750	3.285175
C	-0.676121	2.781739	3.711332
H	-3.590770	3.869964	1.401918
H	-3.293146	4.945719	3.605599
H	-1.438202	4.242114	5.097223
C	0.763348	-1.029792	2.491956
C	2.026979	-0.077622	4.797608
C	0.343046	-1.460448	3.752045
C	1.818327	-0.115806	2.395456
C	2.455919	0.348406	3.540924
C	0.967956	-0.977172	4.900416
H	2.135675	0.246839	1.421725
H	3.282196	1.046503	3.446279
H	2.514440	0.292876	5.694517
C	-1.466062	-2.577684	1.451715
C	-3.761917	-4.060500	2.104319
C	-1.401468	-3.965907	1.621778
C	-2.704677	-1.966013	1.662859
C	-3.864844	-2.677066	1.984557
C	-2.537544	-4.700180	1.936961
H	-0.458449	-4.483166	1.489781
H	-2.468286	-5.776948	2.052411
H	-4.639198	-4.650292	2.346138
C	-3.788706	1.059510	0.239662
C	-6.281125	-0.129952	0.719224
C	-4.950418	1.612034	-0.299554
C	-3.921572	-0.082544	1.027047
C	-5.143900	-0.692843	1.298517
C	-6.185564	1.012120	-0.071954
H	-4.891134	2.504371	-0.913670
H	-7.082281	1.443053	-0.506020
H	-7.255095	-0.574816	0.891109
C	-2.255894	3.013677	-1.235644
C	-2.341259	4.928692	-3.282899
C	-2.785076	2.689564	-2.493726
C	-1.748183	4.298844	-1.027111
C	-1.793322	5.249682	-2.044790
C	-2.834668	3.643846	-3.504862
H	-3.142754	1.680390	-2.678937
H	-1.299424	4.562034	-0.074865
H	-1.391744	6.242257	-1.864468
H	-3.254009	3.379807	-4.471436
H	-2.377373	5.671810	-4.073963
C	1.109465	-2.955937	0.371464
C	2.564713	-5.198975	-0.458053
C	2.184291	-3.418946	1.132915
C	0.786275	-3.607986	-0.825607
C	1.502759	-4.729056	-1.229968
C	2.910575	-4.534467	0.715241
H	2.455268	-2.921981	2.058840
H	-0.030409	-3.240223	-1.440443
H	1.235576	-5.230801	-2.155260
H	3.742920	-4.887567	1.316458
H	3.125118	-6.073760	-0.773947
H	-0.140492	1.428346	2.126857
H	-0.474836	-2.169069	3.843125
H	0.138776	2.463098	4.354871
H	0.627927	-1.308439	5.876972
O	-2.760046	-0.602964	1.550563
C	-5.145309	-1.886379	2.249010
C	-1.875817	2.575920	1.616722
C	-6.398237	-2.749789	2.082452
H	-6.394266	-3.580288	2.793046
H	-7.297852	-2.165718	2.293149
H	-6.476659	-3.157365	1.069693
C	-5.091849	-1.350716	3.699517
H	-5.981253	-0.748688	3.912146
H	-5.056274	-2.184682	4.408181
H	-4.209193	-0.724864	3.861552
C	-1.980471	-1.832394	-5.103108
H	-1.035482	-2.246251	-5.466611
H	-2.769142	-2.099413	-5.813007
H	-1.886520	-0.742148	-5.079754
C	-2.397481	-3.904075	-3.758408
H	-1.535004	-4.334440	-4.276628
H	-2.441252	-4.335430	-2.755845
H	-3.300858	-4.195148	-4.304479
C	-4.022263	-2.454408	-1.824161
H	-3.236000	-2.826931	-1.159726
H	-4.677817	-1.803003	-1.239845
H	-4.610508	-3.304503	-2.183281
C	-4.540439	-1.136195	-3.886356
H	-4.160200	-0.435544	-4.633134

H	-5.033978	-1.965560	-4.403626
H	-5.291000	-0.616352	-3.282656
C	2.850354	2.366922	-0.562527
C	3.849213	1.973284	0.242275
C	2.164218	3.656193	-0.227863
H	4.054635	2.664278	1.067478
B	4.877352	0.801759	0.287125
C	2.040759	4.685697	-1.169492
C	1.669696	3.875935	1.061951
O	6.186379	0.994966	-0.073187
O	4.676158	-0.387830	0.937814
C	1.474037	5.907432	-0.818821
H	2.411287	4.541912	-2.180459
C	1.084029	5.090186	1.410249
H	1.737149	3.077371	1.794157
C	6.913134	-0.221303	0.209973
C	5.969687	-0.952943	1.234384
C	0.993131	6.116138	0.472862
H	1.406420	6.699164	-1.559306
H	0.689403	5.226842	2.413021
C	8.283107	0.147077	0.762661
C	7.068034	-0.965511	-1.112969
C	6.266604	-0.602902	2.693076
C	5.896679	-2.462764	1.064966
H	0.542278	7.066701	0.742270
H	8.830282	-0.753044	1.060559
H	8.865879	0.656926	-0.010084
H	8.208386	0.812228	1.625348
H	7.575037	-0.313669	-1.830236
H	7.663856	-1.875263	-0.993178
H	6.095712	-1.233793	-1.529708
H	5.461275	-0.995834	3.320384
H	7.211260	-1.040158	3.029183
H	6.312073	0.480548	2.840994
H	6.887033	-2.912180	1.190487
H	5.233668	-2.883691	1.826026
H	5.504416	-2.736375	0.083588
H	0.325090	2.444678	-1.555191
C	3.082106	0.441827	-2.266997
C	2.975076	-0.768988	-1.581426
C	3.831036	0.467096	-3.448534
C	3.575619	-1.925047	-2.070164
H	2.404238	-0.805718	-0.661988
C	4.431391	-0.686349	-3.946889
H	3.925758	1.401573	-3.996689
C	4.294904	-1.892221	-3.262607
H	3.480068	-2.851817	-1.514960
H	4.998375	-0.643953	-4.872407
H	4.756546	-2.796755	-3.647919
C	2.334472	1.680106	-1.823125
H	2.510922	2.416464	-2.625892

(*R*)-**TS1-R$_F$**

E: -3268.453560

Cu	-0.452705	0.378912	-0.053788
C	0.512514	1.874918	-1.101260
H	-0.001896	2.828695	-1.205165
H	1.027465	1.575235	-2.015119
C	1.206535	1.625276	0.141012
P	-0.466302	-1.911522	-0.319615
P	-1.957592	1.100961	1.579397
B	-1.284865	0.994123	-1.865687
O	-2.290489	1.942433	-2.000753
O	-1.116238	0.304829	-3.056149
C	-2.958149	1.735877	-3.261339
C	-1.867203	0.977718	-4.087505
C	-1.155829	-0.621241	5.806130
C	-0.412213	0.491190	3.799489
C	-2.721000	-0.174729	4.020624
C	-2.441754	-0.704467	5.280024
C	-0.140036	-0.017929	5.063974
H	-3.730884	-0.234476	3.625440
H	-3.234686	-1.177950	5.851098
H	-0.942515	-1.029963	6.789341
C	-0.552052	-3.050308	1.126638
C	-0.690436	-4.688274	3.391633
C	-1.061975	-4.350944	1.030072
C	-0.128495	-2.577965	2.369299
C	-0.193615	-3.392297	3.497278
C	-1.126394	-5.165930	2.156256
H	0.239940	-1.562882	2.456104
H	0.132363	-3.003849	4.457180
H	-0.747063	-5.323848	4.270335
C	-1.973304	-2.438084	-1.237954
C	-4.396434	-3.050196	-2.501815
C	-1.980550	-3.148199	-2.436774
C	-3.202659	-2.051271	-0.701726
C	-4.427000	-2.352599	-1.293218
C	-3.186434	-3.442944	-3.068636
H	-1.042642	-3.457957	-2.886600
H	-3.183722	-3.986823	-4.007859
H	-5.322621	-3.296013	-3.009715
C	-3.743460	0.837131	1.220524
C	-6.351968	0.429402	0.279852
C	-4.722303	1.818142	1.396832
C	-4.125869	-0.355469	0.600141
C	-5.410790	-0.584703	0.108052
C	-6.016368	1.614656	0.930165
H	-4.462877	2.755492	1.878412
H	-6.766785	2.387037	1.064723
H	-7.362553	0.298926	-0.091182
C	-1.841035	2.924478	1.833401
C	-1.478636	5.695705	2.038780
C	-1.872965	3.733286	0.688849
C	-1.642508	3.521988	3.080855
C	-1.459334	4.900388	3.180871
C	-1.694390	5.108747	0.792867
H	-2.044114	3.281665	-0.284738
H	-1.622028	2.917324	3.981641
H	-1.300972	5.350227	4.156519
H	-1.714197	5.721423	-0.103597
H	-1.328738	6.768342	2.118504
C	0.909155	-2.554850	-1.359576
C	2.983022	-3.378930	-3.048567
C	1.468175	-3.828803	-1.221285
C	1.410424	-1.691520	-2.342316
C	2.439052	-2.103484	-3.184326
C	2.498065	-4.238174	-2.064781
H	1.103768	-4.507247	-0.456497
H	0.971141	-0.706164	-2.461980
H	2.817475	-1.425524	-3.943513
H	2.925574	-5.229555	-1.948765
H	3.787556	-3.700980	-3.703236
H	0.389373	0.929509	3.209260
H	-1.416875	-4.727074	0.074671
H	0.869456	0.042592	5.459211
H	-1.522671	-6.173253	2.070261
O	-3.167864	-1.330813	0.468967
C	-5.696125	-1.934934	-0.550538
C	-1.708968	0.425519	3.270926
C	-6.907429	-1.869093	-1.483691
H	-7.103514	-2.848020	-1.929054
H	-7.808223	-1.593278	-0.929107
H	-6.757435	-1.142624	-2.288806
C	-5.970185	-2.974229	0.561867
H	-6.855662	-2.685757	1.137642
H	-6.146310	-3.960788	0.120890
H	-5.124005	-3.053883	1.250823
C	-0.894281	1.918029	-4.800435
H	-0.055044	1.332603	-5.187713
H	-1.370133	2.433306	-5.640184
H	-0.493648	2.669702	-4.112835

C	-2.407628	-0.060435	-5.058910
H	-1.576856	-0.544825	-5.581212
H	-2.977086	-0.834680	-4.540225
H	-3.052182	0.410069	-5.808908
C	-4.197231	0.886872	-2.980157
H	-3.916726	-0.096382	-2.595720
H	-4.806475	1.386974	-2.221476
H	-4.805551	0.750022	-3.879820
C	-3.366348	3.088271	-3.826730
H	-2.518000	3.773865	-3.882192
H	-3.792352	2.975595	-4.829154
H	-4.127802	3.541060	-3.184261
C	3.630433	2.061190	-0.596892
C	4.831314	1.647647	-1.051344
C	3.207984	3.470448	-0.850766
H	5.453133	2.397033	-1.543447
B	5.474670	0.248101	-0.878258
C	2.719038	4.297904	0.169400
C	3.269575	3.988128	-2.149608
O	6.780582	0.015264	-1.244745
O	4.879367	-0.858428	-0.326415
C	2.309805	5.598938	-0.101492
H	2.654677	3.922518	1.185055
C	2.854091	5.289262	-2.425033
H	3.627674	3.352737	-2.954803
C	7.025956	-1.398799	-1.065320
C	5.939615	-1.784987	-0.008218
C	2.370096	6.099182	-1.401683
H	1.933921	6.220830	0.705560
H	2.902708	5.665516	-3.442761
C	8.462132	-1.599344	-0.608179
C	6.799055	-2.070143	-2.420107
C	6.370619	-1.489480	1.429518
C	5.409706	-3.204034	-0.115767
H	2.039843	7.111678	-1.614017
H	8.642497	-2.652052	-0.367805
H	9.150832	-1.311452	-1.408083
H	8.691712	-0.995583	0.272185
H	7.444998	-1.597436	-3.165476
H	7.040338	-3.136536	-2.382837
H	5.760251	-1.959399	-2.746552
H	5.500199	-1.576704	2.084758
H	7.134688	-2.194654	1.769735
H	6.764961	-0.473414	1.527553
H	6.212402	-3.926329	0.065180
H	4.629739	-3.362370	0.634999
H	4.975316	-3.396615	-1.097203
H	1.016712	2.355141	0.925582
C	3.072952	0.670074	1.485530
C	3.788144	1.493041	2.354510
C	2.694959	-0.597839	1.938672
C	4.095005	1.072305	3.650838
H	4.130447	2.467725	2.018478
C	2.991682	-1.022570	3.227214
H	2.167416	-1.256907	1.255866
C	3.693372	-0.182889	4.095712
H	4.652812	1.731226	4.310385
H	2.680741	-2.011839	3.552419
H	3.931907	-0.510814	5.103379
C	2.634247	1.104007	0.095783
H	2.645567	0.198604	-0.521836

(*R*)-**TS1-S$_A$**
E: -3268.451320

Cu	-0.589084	0.316489	0.541045
P	-2.017137	1.606492	-0.676240
P	-0.002881	-1.743198	-0.471172
B	-1.771385	-0.241800	2.177467
O	-1.971329	-1.476671	2.778813
O	-2.783960	0.634640	2.547243
C	-3.295392	-1.500658	3.349502
C	-3.570957	0.019291	3.587468
C	2.381905	-1.749010	-4.450398
C	1.920824	-0.858572	-2.258992
C	0.616383	-2.701233	-3.101189
C	1.352226	-2.673243	-4.284133
C	2.668666	-0.842943	-3.431326
H	-0.176980	-3.432863	-2.980420
H	1.122267	-3.379355	-5.076343
H	2.955378	-1.733315	-5.372421
C	-1.673945	1.748503	-2.478609
C	-1.032472	1.862066	-5.198355
C	-2.663139	2.036385	-3.425968
C	-0.370368	1.502908	-2.909215
C	-0.047226	1.559966	-4.262165
C	-2.341017	2.098654	-4.778697
H	0.385142	1.251060	-2.172804
H	0.969523	1.351752	-4.581643
H	-0.785888	1.901999	-6.255181
C	-3.732022	0.943066	-0.705992
C	-6.224266	-0.331944	-0.720430
C	-4.878066	1.637497	-0.324520
C	-3.874880	-0.390888	-1.092312
C	-5.099300	-1.052653	-1.125553
C	-6.115789	0.998464	-0.324165
H	-4.801831	2.674087	-0.011909
H	-7.003109	1.542040	-0.015687
H	-7.199830	-0.805469	-0.717520
C	-1.425592	-2.895216	-0.717699
C	-3.714093	-4.512024	-0.863684
C	-1.352458	-4.263614	-0.438729
C	-2.665375	-2.384537	-1.113890
C	-3.825206	-3.160164	-1.182918
C	-2.485898	-5.064362	-0.509252
H	-0.405001	-4.700624	-0.141760
H	-2.414871	-6.122941	-0.281044
H	-4.588867	-5.151913	-0.900322
C	1.087568	-2.737527	0.644605
C	2.657603	-4.156644	2.486839
C	0.594150	-3.041395	1.922089
C	2.377971	-3.147596	0.303450
C	3.151738	-3.859208	1.221585
C	1.373089	-3.742967	2.835126
H	-0.405561	-2.725607	2.204507
H	2.790556	-2.910231	-0.671365
H	4.149375	-4.186471	0.946740
H	0.973382	-3.965890	3.820047
H	3.268539	-4.707267	3.195835
C	-2.231366	3.330924	-0.077136
C	-2.571459	5.886720	1.006387
C	-2.613935	4.398684	-0.894610
C	-2.005070	3.557946	1.284690
C	-2.180880	4.829000	1.824171
C	-2.784294	5.670288	-0.353467
H	-2.776750	4.243480	-1.956620
H	-1.715839	2.728732	1.922533
H	-2.005139	4.993070	2.883113
H	-3.080591	6.493537	-0.996668
H	-2.703774	6.879603	1.425987
H	2.140904	-0.135682	-1.480976
H	-3.689477	2.201066	-3.109515
H	3.463953	-0.112088	-3.548264
H	-3.114507	2.322887	-5.507071
O	-2.724204	-1.055206	-1.445728
C	-5.116965	-2.487262	-1.649930
C	0.894479	-1.790970	-2.078120
C	-6.359209	-3.249112	-1.181159
H	-7.268838	-2.761396	-1.541107
H	-6.369796	-4.262878	-1.589316
H	-6.405088	-3.311858	-0.089241
C	-5.107552	-2.436540	-3.196057
H	-5.084501	-3.451366	-3.606461

H	-6.007374	-1.931560	-3.562332
H	-4.234374	-1.894799	-3.571849
C	-3.022908	0.525476	4.921927
H	-3.064526	1.618729	4.930452
H	-3.608130	0.153114	5.768074
H	-1.980079	0.224011	5.062423
C	-5.023119	0.438363	3.418081
H	-5.118813	1.515135	3.588650
H	-5.386857	0.223028	2.411221
H	-5.661165	-0.078495	4.142820
C	-4.221494	-2.122130	2.303634
H	-4.271941	-1.498533	1.407968
H	-3.826592	-3.100513	2.014045
H	-5.235910	-2.257437	2.691668
C	-3.273612	-2.355924	4.607207
H	-2.499641	-2.028181	5.304502
H	-4.242440	-2.317591	5.115944
H	-3.072468	-3.398203	4.341004
C	2.868879	2.034447	0.078075
C	3.933227	1.828972	-0.721834
C	2.146603	3.336323	-0.065747
H	4.205880	2.659898	-1.374545
B	4.922736	0.634911	-0.770170
C	1.673274	3.721883	-1.324620
C	1.949161	4.210897	1.010821
O	4.635522	-0.693529	-0.596598
O	6.241837	0.861654	-1.079994
C	0.989835	4.921359	-1.500742
H	1.828364	3.060842	-2.170911
C	1.279395	5.417643	0.834720
H	2.314066	3.942315	1.997545
C	5.892261	-1.410049	-0.591941
C	6.851180	-0.418977	-1.340871
C	0.787458	5.773254	-0.419287
H	0.609148	5.182867	-2.483779
H	1.133514	6.078918	1.683757
C	5.717886	-2.742477	-1.305372
C	6.263948	-1.626879	0.873712
C	6.845506	-0.603762	-2.859464
C	8.280637	-0.407032	-0.820873
H	0.247532	6.705811	-0.549790
H	6.690855	-3.221441	-1.455069
H	5.104775	-3.418546	-0.707623
H	5.235040	-2.616458	-2.276707
H	5.447627	-2.159906	1.367661
H	7.177877	-2.220232	0.971753
H	6.403064	-0.674624	1.394783
H	7.366304	0.240635	-3.319661
H	7.355040	-1.524628	-3.156405
H	5.824576	-0.628576	-3.253101
H	8.736287	-1.396193	-0.931467
H	8.877441	0.307250	-1.395704
H	8.323375	-0.117494	0.230982
C	3.387459	0.829434	2.268494
C	4.235334	1.842410	2.724017
C	3.449633	-0.416140	2.906731
C	5.100532	1.626244	3.796271
H	4.243445	2.806482	2.223660
C	4.309523	-0.636924	3.977434
H	2.829249	-1.230581	2.541765
C	5.139909	0.387212	4.430756
H	5.751974	2.429311	4.129716
H	4.341206	-1.616337	4.447005
H	5.817960	0.217937	5.262049
C	2.398209	0.995989	1.111261
H	2.433408	0.028254	0.599732
C	0.960998	1.270091	1.557967
C	0.202447	0.409886	2.446014
H	-0.280065	0.900094	3.295145
H	0.635284	-0.549535	2.725061
H	0.734891	2.320876	1.697296

(*R*)-**TS1-S$_B$**

E: -3268.440570

Cu	-0.415460	0.158268	-0.336419
P	-1.072992	-1.843540	-1.258817
P	-1.111993	0.536463	1.888133
B	-1.624906	1.510220	-1.351120
O	-2.229635	2.691355	-0.945707
O	-2.145002	1.112461	-2.577845
C	-3.353435	2.955565	-1.811604
C	-2.954498	2.181110	-3.107857
C	-0.753701	-2.527621	5.356012
C	-0.539356	-1.948442	3.024637
C	-1.252027	-0.284763	4.616404
C	-1.153329	-1.221323	5.639630
C	-0.454748	-2.892414	4.046799
H	-1.578661	0.726315	4.843525
H	-1.395203	-0.934759	6.658676
H	-0.681289	-3.258758	6.155730
C	-0.554197	-3.330881	-0.308728
C	0.378503	-5.434500	1.283049
C	0.815660	-3.467372	-0.042598
C	-1.448358	-4.259316	0.224674
C	-0.982467	-5.303889	1.022986
C	1.279560	-4.515370	0.743465
H	-2.512333	-4.170654	0.026298
H	-1.687816	-6.016114	1.440295
H	0.738348	-6.248101	1.905608
C	-2.888304	-2.044859	-1.479463
C	-5.676197	-2.027472	-1.766971
C	-3.481334	-2.487120	-2.663330
C	-3.731725	-1.614818	-0.452046
C	-5.122795	-1.603922	-0.558940
C	-4.864375	-2.469462	-2.808612
H	-2.857649	-2.823184	-3.485114
H	-5.313679	-2.802396	-3.738665
H	-6.752416	-2.024838	-1.900138
C	-2.940117	0.790052	1.991301
C	-5.736894	0.989304	2.050385
C	-3.563167	1.830136	2.683347
C	-3.759378	-0.148450	1.360747
C	-5.152485	-0.077463	1.369379
C	-4.950253	1.932032	2.707042
H	-2.960865	2.574381	3.193742
H	-5.422205	2.750714	3.240911
H	-6.816238	1.089592	2.078339
C	-0.421048	2.135059	2.485941
C	0.783420	4.584649	3.132795
C	-0.771321	3.305086	1.796834
C	0.545391	2.207486	3.492049
C	1.147537	3.423654	3.808308
C	-0.181639	4.520542	2.128774
H	-1.497025	3.261478	0.989615
H	0.850809	1.313639	4.023934
H	1.905371	3.456608	4.585291
H	-0.471787	5.418655	1.591419
H	1.251111	5.532409	3.382618
C	-0.400289	-2.183877	-2.937143
C	0.637809	-2.585734	-5.503336
C	-0.110943	-3.476129	-3.388111
C	-0.168048	-1.094577	-3.784544
C	0.345995	-1.296928	-5.062384
C	0.406970	-3.674726	-4.665356
H	-0.288679	-4.330974	-2.742320
H	-0.413714	-0.093146	-3.445556
H	0.523478	-0.444608	-5.711484
H	0.630328	-4.681591	-5.005081
H	1.045241	-2.741881	-6.497742
H	-0.291993	-2.230563	2.007284
H	1.516934	-2.738900	-0.443583
H	-0.149591	-3.907794	3.813558

H	2.342560	-4.610958	0.943303
O	-3.137048	-1.189052	0.710097
C	-5.928228	-1.194440	0.673392
C	-0.929919	-0.637910	3.300601
C	-7.351483	-0.764151	0.310520
H	-7.892395	-1.585222	-0.167375
H	-7.916044	-0.501770	1.209028
H	-7.353293	0.096234	-0.366203
C	-5.997453	-2.408463	1.630005
H	-6.541816	-2.139539	2.541042
H	-6.516977	-3.241532	1.145226
H	-4.997732	-2.747759	1.917410
C	-2.064868	2.997975	-4.045220
H	-1.656421	2.335353	-4.814298
H	-2.625708	3.795156	-4.542328
H	-1.226644	3.449972	-3.505526
C	-4.118061	1.574055	-3.877407
H	-3.741544	1.051357	-4.762023
H	-4.666792	0.851889	-3.268689
H	-4.810714	2.353452	-4.212225
C	-4.591369	2.375998	-1.129482
H	-4.515556	1.289587	-1.038477
H	-4.670787	2.794091	-0.122279
H	-5.506136	2.617647	-1.679620
C	-3.499881	4.460286	-1.979309
H	-2.568019	4.920644	-2.314665
H	-4.286811	4.692583	-2.704450
H	-3.777789	4.912632	-1.022286
C	3.792462	1.766814	-0.966344
C	5.061246	1.439078	-1.291425
C	3.315291	3.159175	-1.211952
H	5.695593	2.236380	-1.681279
B	5.754829	0.084552	-0.974343
C	2.645589	3.901172	-0.229020
C	3.545016	3.762154	-2.454780
O	5.171580	-1.159642	-1.057116
O	7.050633	0.025661	-0.525132
C	2.244337	5.209992	-0.476047
H	2.426506	3.454549	0.736055
C	3.130051	5.066899	-2.708074
H	4.040792	3.191499	-3.234971
C	6.012063	-2.069564	-0.313657
C	7.411443	-1.370788	-0.412800
C	2.482172	5.798885	-1.716310
H	1.737176	5.767650	0.305293
H	3.311016	5.509679	-3.683224
C	5.934826	-3.447405	-0.951546
C	5.458085	-2.115107	1.111450
C	8.177218	-1.738936	-1.682924
C	8.297727	-1.552884	0.809694
H	2.160396	6.817954	-1.909341
H	6.629460	-4.135861	-0.459926
H	4.922887	-3.848516	-0.840026
H	6.170475	-3.413638	-2.017072
H	4.410960	-2.427868	1.073761
H	6.008135	-2.828429	1.732085
H	5.494420	-1.131358	1.590398
H	9.037220	-1.071207	-1.785833
H	8.544426	-2.768521	-1.643527
H	7.549983	-1.625699	-2.572195
H	8.508543	-2.614530	0.973633
H	9.250553	-1.037478	0.657294
H	7.830069	-1.146024	1.708595
C	2.937418	0.678813	1.181922
C	3.799957	1.497087	1.917472
C	2.326696	-0.386804	1.855089
C	4.044166	1.263503	3.271491
H	4.321807	2.313387	1.428983
C	2.582191	-0.637980	3.198521
H	1.660742	-1.039437	1.298289
C	3.445849	0.187573	3.917705
H	4.719180	1.920461	3.812855
H	2.104365	-1.482815	3.685563
H	3.647536	-0.008198	4.966715
C	2.841470	0.735029	-0.348045
H	3.294803	-0.221482	-0.613754
C	1.497214	0.670533	-1.076940
C	0.537103	1.738245	-1.237252
H	0.306536	2.066066	-2.253369
H	0.562059	2.563289	-0.530434
H	1.625297	0.094599	-1.995118

(*R*)-**TS1-S$_C$**

E: -3268.455224

Cu	-0.832826	-0.676298	0.405735
P	0.159883	-0.287700	-1.705202
P	-2.676403	0.722940	0.647837
B	-1.631549	-2.646057	0.447109
O	-1.306677	-3.668477	-0.433828
O	-2.798625	-2.964373	1.137714
C	-2.378698	-4.635390	-0.446141
C	-3.085088	-4.362118	0.921933
C	-6.048520	-0.204922	-2.407474
C	-4.655297	-0.849572	-0.544060
C	-4.430374	1.348210	-1.509541
C	-5.421430	1.036667	-2.438900
C	-5.664070	-1.146917	-1.454310
H	-3.966426	2.328845	-1.539685
H	-5.705740	1.772732	-3.184682
H	-6.827268	-0.441271	-3.126312
C	-1.105847	-0.237898	-3.049374
C	-3.100609	-0.349354	-5.008344
C	-1.137270	0.707547	-4.076521
C	-2.080101	-1.238813	-3.014201
C	-3.067186	-1.301010	-3.991361
C	-2.135683	0.654255	-5.047372
H	-2.059308	-1.968339	-2.211258
H	-3.818115	-2.084268	-3.947543
H	-3.878641	-0.386616	-5.765041
C	1.016864	1.327862	-1.890758
C	2.104011	3.911506	-1.950894
C	2.359118	1.518749	-2.214166
C	0.260022	2.460309	-1.593795
C	0.750512	3.762191	-1.644919
C	2.900415	2.801874	-2.225247
H	2.987965	0.660090	-2.422987
H	3.952907	2.938260	-2.449863
H	2.546656	4.901067	-1.985869
C	-2.491600	2.561352	0.572420
C	-1.941255	5.308562	0.471393
C	-3.151426	3.447309	1.427468
C	-1.600318	3.104333	-0.350889
C	-1.269043	4.457902	-0.404749
C	-2.888101	4.811122	1.365127
H	-3.861478	3.068378	2.155041
H	-3.407275	5.491149	2.032722
H	-1.731709	6.372765	0.461843
C	-3.438631	0.524334	2.311460
C	-4.385532	0.272557	4.932770
C	-2.561775	0.636720	3.397800
C	-4.795202	0.297414	2.550730
C	-5.263201	0.164561	3.857315
C	-3.032955	0.517463	4.700219
H	-1.509013	0.836935	3.219278
H	-5.493706	0.222496	1.723529
H	-6.319299	-0.017892	4.032307
H	-2.341220	0.614250	5.531680
H	-4.754553	0.170216	5.948899
C	1.351451	-1.506211	-2.399237
C	3.027386	-3.496131	-3.425376
C	1.844788	-1.411432	-3.706668
C	1.704448	-2.606296	-1.616948

C	2.537625	-3.599681	-2.127286
C	2.683132	-2.397872	-4.214537
H	1.563087	-0.568913	-4.332693
H	1.304493	-2.693851	-0.613881
H	2.799990	-4.453553	-1.509866
H	3.060145	-2.316601	-5.229530
H	3.676723	-4.269104	-3.825460
H	-4.335661	-1.598586	0.175223
H	-0.388178	1.492899	-4.122598
H	-6.141752	-2.122367	-1.426704
H	-2.156926	1.399558	-5.837143
O	-1.045811	2.230861	-1.247426
C	-0.244999	4.907413	-1.448008
C	-4.041854	0.410330	-0.550930
C	0.454356	6.205174	-1.035439
H	1.166433	6.519425	-1.803098
H	-0.268384	7.018311	-0.929649
H	0.991265	6.090405	-0.088622
C	-0.984662	5.131522	-2.788217
H	-1.726564	5.929530	-2.680075
H	-0.273856	5.418654	-3.569984
H	-1.503736	4.224130	-3.112550
C	-2.455225	-5.130348	2.084753
H	-2.860150	-4.745544	3.025292
H	-2.674350	-6.200672	2.027861
H	-1.368710	-4.999975	2.105575
C	-4.592854	-4.564076	0.919072
H	-4.998468	-4.325789	1.906890
H	-5.086303	-3.921542	0.186233
H	-4.842193	-5.605646	0.691506
C	-3.269971	-4.329363	-1.650358
H	-3.733901	-3.342828	-1.560870
H	-2.659881	-4.339707	-2.558427
H	-4.060835	-5.077437	-1.760842
C	-1.777271	-6.026175	-0.589967
H	-1.019064	-6.217414	0.172289
H	-2.554236	-6.793685	-0.511105
H	-1.303735	-6.122893	-1.571622
C	2.272696	0.932905	1.767341
C	3.278603	1.510022	1.080744
C	1.428107	1.796504	2.646868
H	3.410473	2.584274	1.215084
B	4.381726	0.804162	0.251523
C	0.889923	2.986951	2.146701
C	1.184756	1.476453	3.990700
O	4.369100	-0.491050	-0.203919
O	5.562532	1.449978	-0.032653
C	0.134704	3.829466	2.957017
H	1.058229	3.243115	1.104918
C	0.431549	2.319047	4.802967
H	1.587225	0.559871	4.409461
C	5.728793	-0.837012	-0.543463
C	6.368811	0.564834	-0.838924
C	-0.102556	3.498085	4.287996
H	-0.277370	4.742340	2.540702
H	0.262267	2.050592	5.841817
C	5.714284	-1.793579	-1.724187
C	6.326705	-1.525593	0.683919
C	6.218534	1.003004	-2.295446
C	7.822685	0.701174	-0.408929
H	-0.698676	4.151516	4.917888
H	6.734462	-1.995197	-2.066723
H	5.262352	-2.741891	-1.422164
H	5.133722	-1.395553	-2.558961
H	5.712871	-2.393766	0.935924
H	7.349107	-1.865129	0.493748
H	6.333312	-0.860495	1.552696
H	6.494307	2.058095	-2.380028
H	6.873610	0.424261	-2.953132
H	5.190616	0.887679	-2.648220
H	8.448553	-0.025975	-0.936105
H	8.187921	1.703109	-0.653403
H	7.940158	0.549693	0.665850
C	2.979368	-1.402019	2.513495
C	3.751965	-0.881902	3.555502
C	3.147274	-2.757216	2.197320
C	4.628678	-1.690000	4.280089
H	3.694539	0.175324	3.795386
C	4.014988	-3.570204	2.918418
H	2.608711	-3.175722	1.352028
C	4.759837	-3.040061	3.971518
H	5.215567	-1.255303	5.084494
H	4.123756	-4.616550	2.646473
H	5.443012	-3.669624	4.533831
C	1.986048	-0.570643	1.689423
H	2.184383	-0.856693	0.652139
C	0.518948	-0.867239	1.992710
C	-0.101087	-2.144264	1.691528
H	-0.746229	-2.537004	2.477401
H	0.515564	-2.925620	1.250998
H	0.153067	-0.427154	2.912887

(*R*)-**TS1-R-PH$_2$**

E: -2344.471662

Cu	0.156451	-0.225430	-0.481886
C	-1.112247	0.731577	0.829428
H	-1.039099	1.816443	0.850447
H	-1.262506	0.306345	1.822783
C	-1.874267	0.129264	-0.242675
B	0.972390	0.631747	1.236354
O	1.535686	1.895252	1.319725
O	1.326415	-0.138945	2.331534
C	2.523824	1.869773	2.370991
C	2.003151	0.711077	3.281817
C	2.652127	-2.577679	0.244123
C	5.303011	-2.476593	1.140939
C	3.039599	-3.293411	1.377819
C	3.626174	-1.836479	-0.421499
C	4.955543	-1.763442	-0.006427
C	4.356615	-3.239010	1.823360
H	2.303853	-3.873890	1.926064
H	4.648457	-3.789020	2.712161
H	6.322087	-2.445531	1.510489
C	2.981157	1.138117	-2.129620
C	5.679483	1.559693	-1.516507
C	3.550008	2.402707	-2.291502
C	3.802548	0.106878	-1.679131
C	5.146650	0.281369	-1.354131
C	4.892624	2.608558	-1.988613
H	2.937469	3.229909	-2.637398
H	5.328227	3.594911	-2.111438
H	6.718814	1.748035	-1.270950
O	3.218457	-1.130299	-1.529911
C	5.923242	-0.938524	-0.857114
C	7.173007	-0.535823	-0.071043
H	7.720776	-1.421709	0.261023
H	7.857566	0.036016	-0.703225
H	6.921097	0.068387	0.806259
C	6.347473	-1.785634	-2.079366
H	7.015345	-1.206087	-2.724844
H	6.875366	-2.686085	-1.749176
H	5.481669	-2.094335	-2.672354
C	0.954330	1.173066	4.293812
H	0.487831	0.294855	4.749718
H	1.398971	1.777619	5.090110
H	0.169335	1.760667	3.807471
C	3.091486	-0.094540	3.975194
H	2.637561	-0.880576	4.586526
H	3.757673	-0.572047	3.253240
H	3.686307	0.546405	4.634587
C	3.868922	1.552486	1.716462
H	3.861188	0.554188	1.270941

H	4.056761	2.277251	0.919317
H	4.691641	1.606630	2.436399
C	2.572632	3.236855	3.036333
H	1.582307	3.560327	3.364118
H	3.240866	3.220366	3.903709
H	2.955660	3.978542	2.328705
C	-4.158770	-0.646564	0.609974
C	-4.634958	0.607707	0.620039
C	-4.998942	-1.779674	1.098654
H	-5.632766	0.735309	1.048856
B	-3.973163	1.937165	0.125877
C	-4.492882	-2.725486	1.999482
C	-6.315467	-1.931288	0.646536
O	-4.061718	2.398527	-1.169349
O	-3.451478	2.878873	0.980877
C	-5.284972	-3.778796	2.447464
H	-3.476385	-2.628566	2.370484
C	-7.107335	-2.987071	1.089115
H	-6.710615	-1.219529	-0.072767
C	-3.346279	3.651512	-1.243903
C	-3.330955	4.121299	0.255994
C	-6.595001	-3.914977	1.992887
H	-4.877645	-4.494742	3.155478
H	-8.124007	-3.088967	0.720719
C	-4.092177	4.578794	-2.193661
C	-1.951094	3.364684	-1.805124
C	-4.546667	4.968265	0.633863
C	-2.052481	4.824456	0.690255
H	-7.210360	-4.740642	2.337531
H	-3.631371	5.571660	-2.202294
H	-4.048433	4.176325	-3.210079
H	-5.142651	4.682782	-1.915146
H	-2.054497	2.830982	-2.754672
H	-1.404898	4.294517	-1.990175
H	-1.353773	2.745139	-1.132661
H	-4.562341	5.101612	1.719249
H	-4.509921	5.956510	0.166604
H	-5.479383	4.477572	0.339886
H	-1.901841	5.739795	0.108971
H	-2.125985	5.101937	1.745894
H	-1.174355	4.186326	0.569349
H	-2.202438	0.773170	-1.051252
C	-2.986501	-1.917684	-1.170319
C	-3.624207	-1.403520	-2.303701
C	-2.532481	-3.237115	-1.200523
C	-3.778887	-2.181787	-3.446696
H	-3.999129	-0.382638	-2.285300
C	-2.686790	-4.021518	-2.342741
H	-2.056095	-3.656784	-0.317146
C	-3.307539	-3.494627	-3.472138
H	-4.273206	-1.765269	-4.319910
H	-2.325809	-5.046178	-2.346790
H	-3.430907	-4.102909	-4.363284
C	-2.783906	-1.039702	0.056070
H	-2.294139	-1.662553	0.816464
P	0.907430	-2.415172	-0.306711
H	0.880197	-3.301280	-1.414598
H	0.301536	-3.282016	0.634103
P	1.185011	0.795874	-2.306441
H	0.759513	2.077620	-2.732508
H	1.154943	0.165164	-3.576943

(*R*)-**TS1-S-PH$_2$**

E: -2344.470239

Cu	-0.029667	-0.350440	0.188275
P	0.902855	-2.391987	-0.447685
P	0.486242	1.219880	-1.465960
B	1.171056	0.121951	1.830695
O	1.698071	1.378116	2.090386
O	1.808105	-0.845227	2.590072
C	2.916623	1.200585	2.842367
C	2.679012	-0.182841	3.531450
C	2.737047	-2.467731	-0.367084
C	5.520896	-2.279925	-0.124655
C	3.439264	-3.388267	0.412233
C	3.467887	-1.479440	-1.021317
C	4.853524	-1.354438	-0.926811
C	4.822062	-3.290680	0.533548
H	2.900827	-4.167913	0.942553
H	5.360805	-4.003075	1.149997
H	6.597734	-2.220851	-0.009724
C	2.244352	1.721563	-1.638056
C	4.982925	2.301660	-1.600665
C	2.682047	3.045963	-1.574631
C	3.209574	0.720249	-1.705333
C	4.580595	0.968645	-1.674385
C	4.044157	3.331203	-1.560339
H	1.956044	3.851615	-1.518490
H	4.378756	4.362178	-1.506924
H	6.038721	2.547501	-1.570701
O	2.751863	-0.576024	-1.772277
C	5.523005	-0.234699	-1.726262
C	6.910426	0.105618	-1.177648
H	7.570193	-0.764237	-1.234906
H	7.377862	0.893494	-1.774231
H	6.861904	0.439167	-0.136217
C	5.659951	-0.694568	-3.196236
H	6.106486	0.102261	-3.799733
H	6.302091	-1.579276	-3.255848
H	4.687536	-0.947339	-3.628708
C	1.909876	-0.071360	4.848078
H	1.607151	-1.072229	5.169617
H	2.522384	0.372498	5.638670
H	1.005885	0.533614	4.726738
C	3.932011	-1.024766	3.720204
H	3.672444	-1.971860	4.203240
H	4.408884	-1.253279	2.764525
H	4.653716	-0.505192	4.359473
C	4.066476	1.174131	1.834816
H	3.978576	0.315269	1.164444
H	4.028633	2.082923	1.227531
H	5.039987	1.129048	2.333132
C	3.084379	2.372325	3.797929
H	2.199327	2.513271	4.422114
H	3.950969	2.217561	4.449278
H	3.249285	3.292334	3.228802
C	-3.127060	1.169753	-1.001341
C	-3.571238	0.092102	-1.672009
C	-3.021746	2.480742	-1.713881
H	-3.859271	0.263962	-2.711718
B	-3.755125	-1.390888	-1.237416
C	-2.619096	2.529756	-3.057145
C	-3.274836	3.696475	-1.062271
O	-4.477495	-1.821044	-0.150847
O	-3.331895	-2.428659	-2.031272
C	-2.487709	3.741777	-3.727065
H	-2.374027	1.606510	-3.573931
C	-3.149740	4.909697	-1.733364
H	-3.588174	3.698445	-0.023600
C	-2.754915	4.939812	-3.068523
H	-2.163280	3.749331	-4.763553
H	-3.362890	5.835738	-1.207317
H	-2.649463	5.887218	-3.588430
C	-3.772053	1.576453	1.421351
C	-5.044278	0.997630	1.376043
C	-3.506266	2.514059	2.426253
C	-6.015855	1.341044	2.312399
H	-5.268619	0.260379	0.612420
C	-4.476476	2.859635	3.364435
H	-2.526520	2.984737	2.467851
C	-5.738528	2.273366	3.310876
H	-6.997853	0.878873	2.259575

H	-4.246601	3.591739	4.133526
H	-6.499795	2.542303	4.037208
C	-2.656812	1.171124	0.454195
H	-1.904447	1.967589	0.519738
C	-1.960948	-0.098527	0.937080
C	-0.968314	0.053585	1.980597
H	-0.888010	-0.756606	2.704004
H	-0.861025	1.038527	2.437443
H	-2.583701	-0.984865	1.003831
C	-3.582607	-3.656477	-1.309172
C	-4.718637	-3.236091	-0.313062
C	-2.280666	-4.033318	-0.604342
H	-2.375759	-4.978767	-0.062825
H	-1.495227	-4.148229	-1.356824
H	-1.974520	-3.252325	0.098833
C	-6.121548	-3.378713	-0.902405
H	-6.407640	-4.429461	-1.003803
H	-6.837867	-2.890072	-0.235977
H	-6.191681	-2.901368	-1.884444
C	-4.651820	-3.903818	1.051896
H	-5.474305	-3.545405	1.677616
H	-4.748191	-4.989655	0.952663
H	-3.713946	-3.679779	1.564177
H	0.152841	0.938703	-2.815866
H	-0.099036	2.506291	-1.402549
H	0.613039	-3.561961	0.294648
H	0.674351	-2.937988	-1.737713
C	-3.974580	-4.741468	-2.300558
H	-3.126694	-4.969210	-2.953181
H	-4.251893	-5.659065	-1.772012
H	-4.813095	-4.432276	-2.927770

(*R*)-**1a**$_1$

E: -1067.803408

C	-0.520435	0.564481	3.146738
H	-1.446650	0.915740	3.593527
H	0.126612	-0.047872	3.772037
C	-0.197167	0.871638	1.893200
C	0.958193	-0.827576	0.352546
C	-0.176021	-1.327459	-0.164109
C	2.266050	-1.480068	0.040011
H	-0.057469	-2.256290	-0.726914
B	-1.647276	-0.814185	-0.129467
C	3.167371	-1.845227	1.047514
C	2.615614	-1.731254	-1.292050
O	-2.055248	0.429962	-0.546063
O	-2.697735	-1.640785	0.173105
C	4.375893	-2.459076	0.731517
H	2.916258	-1.672351	2.090334
C	3.827856	-2.337165	-1.609686
H	1.933733	-1.430385	-2.082118
C	-3.480562	0.524506	-0.305986
C	-3.908615	-0.984236	-0.271228
C	4.711879	-2.704649	-0.598188
H	5.056020	-2.748061	1.527280
H	4.084096	-2.516058	-2.649722
C	-4.109894	1.342273	-1.422903
C	-3.671492	1.231770	1.035108
C	-4.233339	-1.548128	-1.654439
C	-5.032272	-1.305871	0.701815
H	5.658182	-3.177040	-0.843919
H	-5.199352	1.355249	-1.318855
H	-3.751869	2.374646	-1.370874
H	-3.856929	0.943521	-2.407241
H	-3.183907	2.210324	0.997671
H	-4.732175	1.388271	1.250654
H	-3.228917	0.659195	1.854978
H	-4.329327	-2.634969	-1.581294
H	-5.174532	-1.145092	-2.038906
H	-3.439033	-1.325122	-2.373239
H	-5.938031	-0.752109	0.435365
H	-5.262905	-2.374232	0.658266
H	-4.760099	-1.058535	1.729713
H	-0.869287	1.485630	1.301338
C	1.696720	1.543624	0.390136
C	1.001436	2.075813	-0.700742
C	2.965755	2.039478	0.689280
C	1.563707	3.091183	-1.468005
H	0.015023	1.690024	-0.947978
C	3.532350	3.053484	-0.081360
H	3.517141	1.625783	1.529720
C	2.831969	3.583214	-1.161407
H	1.012300	3.497469	-2.311049
H	4.522122	3.427579	0.163552
H	3.271515	4.373249	-1.762966
C	1.079354	0.423567	1.222614
H	1.791638	0.183013	2.019389

(*R*)-**1a**$_2$

E: -1067.802631

C	1.092739	-0.821022	0.138332
C	-0.027409	-1.311421	-0.420069
C	2.401271	-1.497582	-0.115777
H	0.110210	-2.188859	-1.055779
B	-1.515982	-0.864517	-0.318034
C	2.477683	-2.896905	-0.153175
C	3.581662	-0.766521	-0.310634
O	-1.989222	0.396479	-0.582057
O	-2.515127	-1.762405	-0.048522
C	3.684891	-3.543483	-0.399224
H	1.584002	-3.484287	0.035884
C	4.788475	-1.413141	-0.561578
H	3.558264	0.317922	-0.286189
C	4.846238	-2.803801	-0.609000
H	3.718620	-4.628856	-0.416777
H	5.686956	-0.824841	-0.723147
H	5.789313	-3.306925	-0.800007
C	1.499876	1.690123	0.364654
C	0.874912	2.057210	-0.829477
C	2.426013	2.566906	0.937043
C	1.175271	3.273009	-1.438993
H	0.142876	1.393847	-1.278686
C	2.730430	3.780946	0.326772
H	2.915172	2.291567	1.868235
C	2.105228	4.137663	-0.865975
H	0.681157	3.543890	-2.367582
H	3.456833	4.447141	0.782925
H	2.341560	5.082666	-1.345891
C	1.156763	0.384601	1.082441
H	1.976101	0.180289	1.780788
C	-0.089649	0.586738	1.917062
C	-0.181533	0.257351	3.202932
H	-1.092055	0.436815	3.768340
H	0.647616	-0.202446	3.737216
H	-0.933396	1.054777	1.416265
C	-3.735716	-1.004228	0.130146
C	-3.432328	0.312106	-0.666718
C	-3.894936	-0.775467	1.632940
H	-4.826552	-0.250244	1.861195
H	-3.912933	-1.744618	2.139016
H	-3.058150	-0.199238	2.039548
C	-4.905218	-1.815935	-0.404524
H	-5.042369	-2.712713	0.206486
H	-5.828304	-1.229485	-0.358913
H	-4.740958	-2.129197	-1.437424
C	-3.779403	0.213709	-2.151447
H	-4.861607	0.208838	-2.308637
H	-3.360310	1.079313	-2.671976
H	-3.357633	-0.690298	-2.600878
C	-4.032387	1.574414	-0.067732
H	-3.757304	2.439133	-0.678420
H	-5.124544	1.505539	-0.048033

H -3.675107 1.748653 0.949255

References

1- a) Rivera-Chao, E.; Mitxelena, M.; Varela, J. A.; Fañanás-Mastral, M. *Angew. Chem., Int. Ed.* **2019**, *58*, 18230– 18234; b) Barbeira-Arán, S.; Sanchez-Sordo, I.; Fañanás-Mastral, M. *Org. Lett.* **2024**, *26,* 3784–3789.

2- Dominguez-Molano, P.; Solé-Daura, A.; Carbó, J. J. E. Fernández, *Adv. Sci.* **2024**, *11*, e2309779.

3- Davenport, E.; Fernández, E. *Chem. Commun.,* **2018**, *54*, 10104-10107.

4- Gaussian 16, Revision C.01, M. J. Frisch, G. W. Trucks, H. B. Schlegel, G. E. Scuseria, M. A. Robb, J. R. Cheeseman, G. Scalmani, V. Barone, G. A. Petersson, H. Nakatsuji, X. Li, M. Caricato, A. V. Marenich, J. Bloino, B. G. Janesko, R. Gomperts, B. Mennucci, H. P. Hratchian, J. V. Ortiz, A. F. Izmaylov, J. L. Sonnenberg, D. Williams-Young, F. Ding, F. Lipparini, F. Egidi, J. Goings, B. Peng, A. Petrone, T. Henderson, D. Ranasinghe, V. G. Zakrzewski, J. Gao, N. Rega, G. Zheng, W. Liang, M. Hada, M. Ehara, K. Toyota, R. Fukuda, J. Hasegawa, M. Ishida, T. Nakajima, Y. Honda, O. Kitao, H. Nakai, T. Vreven, K. Throssell, J. A. Montgomery, Jr., J. E. Peralta, F. Ogliaro, M. J. Bearpark, J. J. Heyd, E. N. Brothers, K. N. Kudin, V. N. Staroverov, T. A. Keith, R. Kobayashi, J. Normand, K. Raghavachari, A. P. Rendell, J. C. Burant, S. S. Iyengar, J. Tomasi, M. Cossi, J. M. Millam, M. Klene, C. Adamo, R. Cammi, J. W. Ochterski, R. L. Martin, K. Morokuma, O. Farkas, J. B. Foresman, and D. J. Fox, Gaussian, Inc., Wallingford CT, **2016**.

5- R. G. Parr, W. Yang, Oxford University Press: Oxford, U.K., **1989**.

6- J. D. Chai, M. Head-Gordon, *Phys. Chem. Chem. Phys.,* **2008**, *10*, 6615.

7- a) P. J. Hay, W. R. Wadt, *J. Chem. Phys.*, **1985**, *82,* 270 (b) P. J. Hay, W. R. Wadt, *J. Chem. Phys.*, **1985**, *82*, 284, (c) P. J. Hay, W. R. Wadt, *J. Chem. Phys.*, **1985**, *82*, 299.

8- a) A. Höllwarth, M. Böhme, S. Dapprich, A. W. Ehlers, A. Gobbi, V. Jonas, K. F. Köler, R. Stegmann, A. Veldkamp, G. Frenking, *Chem. Phys. Lett.* **1993**, *208*, 237; b) A. Höllwarth, M. Böhme, S. Dapprich, A. W. Ehlers, A. Gobbi, V. Jonas, K. F. Köler, R. Stegmann, A. Veldkamp, G. Frenking, *Chem. Phys. Lett.* **1993**, 111.

9- a) M. S. Gordon, *Chem. Phys. Lett.*, **1980**, *76*, 163; b) R. C. J. Binning, L. A. Curtiss, *Comput. Chem.* **1990**, *11*, 1206; c) A. D. McLean, G. S. Chandler, *J. Phys. Chem.*, **1980**, *72*, 5639.

10- A. V. Marenich, C. J. Cramer, D. G. J. Truhlar, *Phys. Chem. B*, **2009**, *113*, 6378.

11- a) S. Aguado-Ullate, S. Saureu, L. Guasch, J. J. Carbó, *Chem. Eur. J.* **2012**, *18*, 995, b) S. Aguado-Ullate, M. Urbano-Cuadrado, I. Villaba, E. Pires, J. I. García, C. Bo, J. J. Carbó, *Chem. Eur. J.* **2012**, *18*, 14026; (c) MolQuO application (accessed Feb 2026): http://rodi.urv.es/~carbo/quadrants/index.html
12- T. Lu, Q. Chen, *J. Comput. Chem.* **2022**, *43* (8), 539–555; Erratum: *J. Comput. Chem.* **2022**, *43* (13), 951.
13- T. Lu, Q. Chen, In *Comprehensive Computational Chemistry*, Elsevier: Oxford, **2024**, 2, 240–264.
14- T. Lu and F. Chen, *J. Comput. Chem*. **2012**, *33*, 580-592.
15- T. Lu, *J. Chem. Phys.,* **2024**, *161*, 082503.
16- M. Álvarez-Moreno, C. de Graaf, N. López, F. Maseras, J. M. Poblet, C. Bo, *J. Chem. Inf. Model*. **2015**, *55*(1), 95–103.
17- C. Bo, F. Maseras, N. López*, Nat. Catal.* **2018**, *1*, 809–810.
